# Inherently chiral calixarenes by a catalytic enantioselective desymmetrizing cross-dehydrogenative coupling†

**DOI:** 10.1039/d2sc06234h

**Published:** 2022-12-06

**Authors:** Xin Zhang, Shuo Tong, Jieping Zhu, Mei-Xiang Wang

**Affiliations:** a Key Laboratory of Bioorganic Phosphorus Chemistry and Chemical Biology (Ministry of Education), Department of Chemistry, Tsinghua University Beijing 100084 China tongshuo@mail.tsinghua.edu.cn http://mascl.group; b Laboratory of Synthesis and Natural Products (LSPN), Institute of Chemical Sciences and Engineering, Ecole Polytechnique Fédérale de Lausanne, EPFL-SB-ISIC-LSPN BCH5304 CH-1015 Lausanne Switzerland

## Abstract

Under the catalysis of PdBr_2_ and a chiral phosphoramidite ligand, the upper-rim mono (2-bromoaroyl)-substituted calix[4]arene derivatives underwent a facile enantioselective desymmetrization reaction to afford 9*H*-fluorene-embedded inherently chiral calixarenes in good yields with excellent enantioselectivities. The transannular dehydrogenative arene–arene coupling reaction proceeded most probably through an oxidative addition of the C_aryl_–Br bond to a ligated palladium catalyst followed by a sequence of an enantioselective 1,5-palladium migration and an intramolecular C–H arylation sequence. This new family of inherently chiral calixarenes possesses unique chiroptical properties thanks to their highly rigid structure induced by the 9*H*-fluorene segment.

## Abstract 2

Reported here is the catalytic enantioselective desymmetrization of calixarene skeletons for the construction of inherently chiral calixarenes through a transannular arene–arene dehydrogenative coupling reaction.

## Graphical abstract

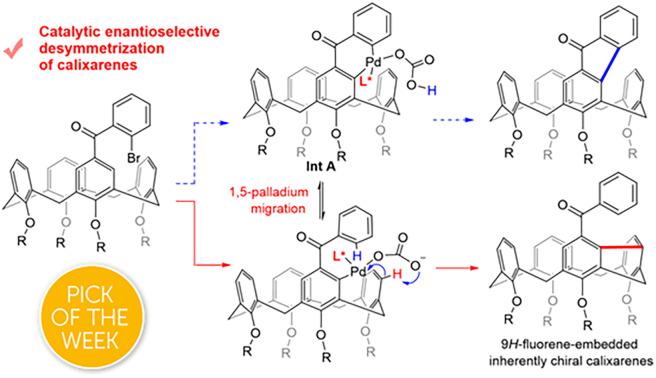

## Introduction

**Scheme 1:**
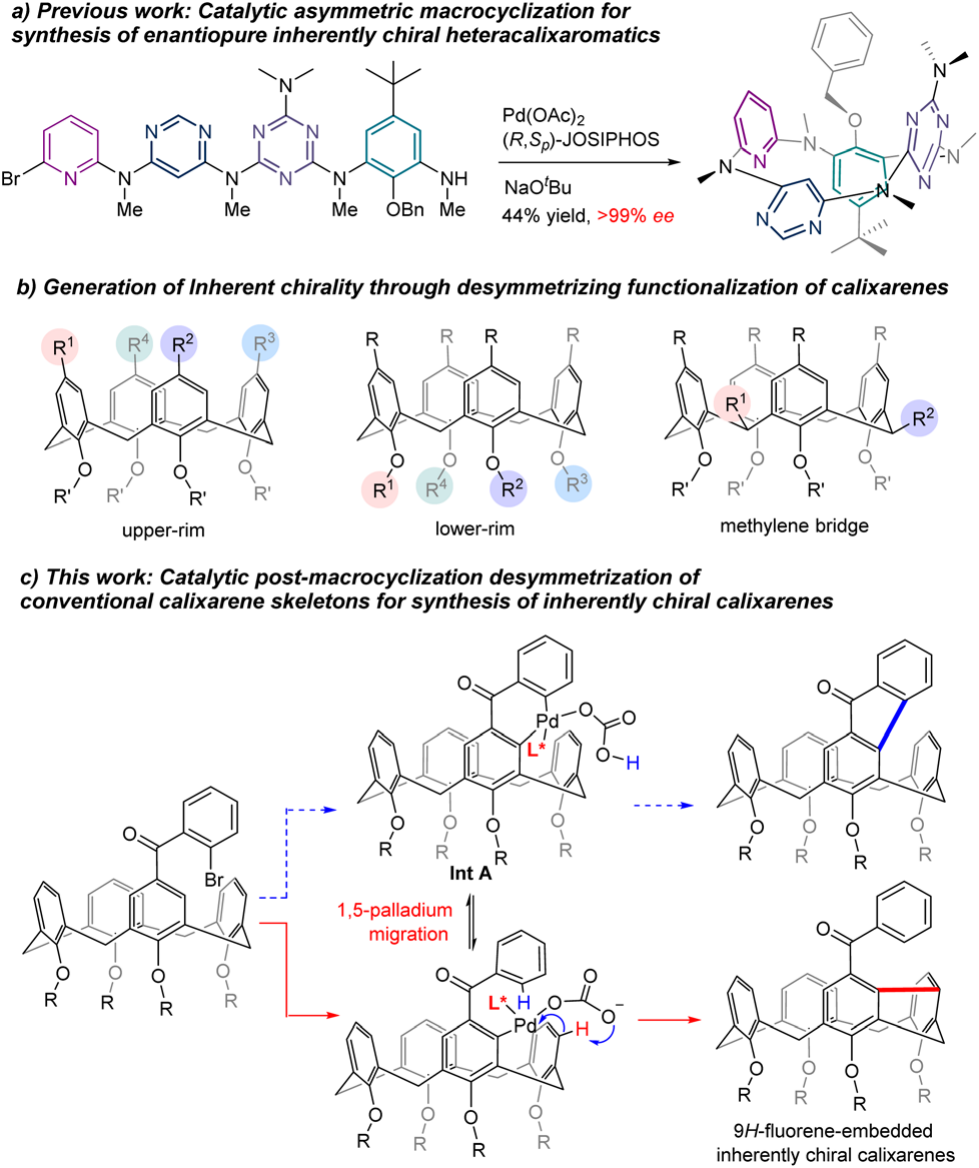
Strategies for the construction of inherently chiral macrocycles.

Inherently chiral macrocycles, coined by Böhmer in 1994, are a unique type of chiral chemical entity.^1^ Different from chiral molecules bearing a central, an axial and a planar chirality and helicity, the inherent chirality results from the three-dimensional curvature architecture of macrocycles.^2–4^ Calix[*n*]arenes are prototypical inherently chiral molecules from which the term originated. With their unique three-dimensional bowl-shaped structures, controllable cavity sizes and shapes, easy preparation and post-chemical modifications, calix[*n*]arenes and their aza- as well as oxa-analogues have become privileged scaffolds in catalysis, molecular recognition, sensing, nanotechnology, biotechnology, *etc.*^5–7^ The inherently chiral calixarenes and analogues have not been the subject of intensive investigation due mainly to the inaccessibility of enantioenriched chiral calix[n]arenes. Indeed, most of the enantioenriched calixarenes and resorcinarenes documented in the literature are synthesized by separation of enantiomers using chiral HPLC columns.^2–4,8–12^ Kinetic resolution of racemates has been reported to give chiral calixarenes with either low efficiency or modest enantioselectivity while diastereoselective synthesis using chiral auxiliary requires inevitably multiple reaction steps and sometimes tedious separation of diastereomers.^13–18^ Catalytic enantioselective synthesis of an intrinsically chiral tetraazacalix[4]arene was attempted by Tsue in 2009.^19^ However, under their optimized conditions, Pd-catalyzed C_aryl_–N bond-forming macrocyclization afforded the product with only 35% ee and only one example was documented in this paper. Very recently, we reported a general synthesis of highly enantioenriched ABCD-type heteracalixaromatics *via* a Pd-catalyzed intramolecular Buchwald–Hartwig reaction.^20^ Linear precursors containing benzene, pyridine, pyrimidine and triazine rings underwent efficient cyclization to furnish nitrogen-bridged calixarenes with ee up to >99% (Scheme 1a). These chiral macrocycles exhibit excellent and intriguing proton-triggered switchable CPL properties.

In view of the easy accessibility of calixarenes, desymmetrization is, without doubt, an obvious option to pursue in order to access enantioenriched calixarenes. In principle, selective functionalization of the upper rim,^3^ the lower rim^11^ and the methylene bridge^21^ could break the symmetry of calixarenes, generating therefore the inherent chirality (Scheme 1b). All these three routes have indeed been exploited using a chiral auxiliary approach with limited success. On the other hand, catalytic enantioselective desymmetrization of calixarenes remains, to the best of our knowledge, unknown. Aiming at creating new inherently chiral macrocyclic systems and searching for novel chiroptical organic molecules,^22^ we set out to investigate the catalytic enantioselective desymmetrization of conventional calix[4]arenes. Our initial design was to construct a 9*H*-fluoren-9-one moiety within the skeleton of calix[4]arenes taking advantage of the Pd-catalyzed intramolecular C–H arylation process (Scheme 1c). We hypothesized that, in the presence of an appropriate chiral ligand, the ArPdXL* species generated *in situ via* oxidative addition would be able to discriminate the two enantiotopic *ortho* C–H bonds leading, after reductive elimination of the 6-membered palladacycle Int A (Scheme 1c), to chiral calix[4]arene.^23,24^ Surprisingly, a more complex reaction sequence occurred affording chiral *meta*–*meta* bridged calix[4]arenes in good yields with high enantioselectivities (Scheme 1c). Although one example of this type of calix[4]arenes is reported in the literature,^25,26^ no enantioselective synthesis is known to date. We disclose herein the results of our study.

## Results and discussion

### Optimization of reaction conditions

**Scheme 2:**
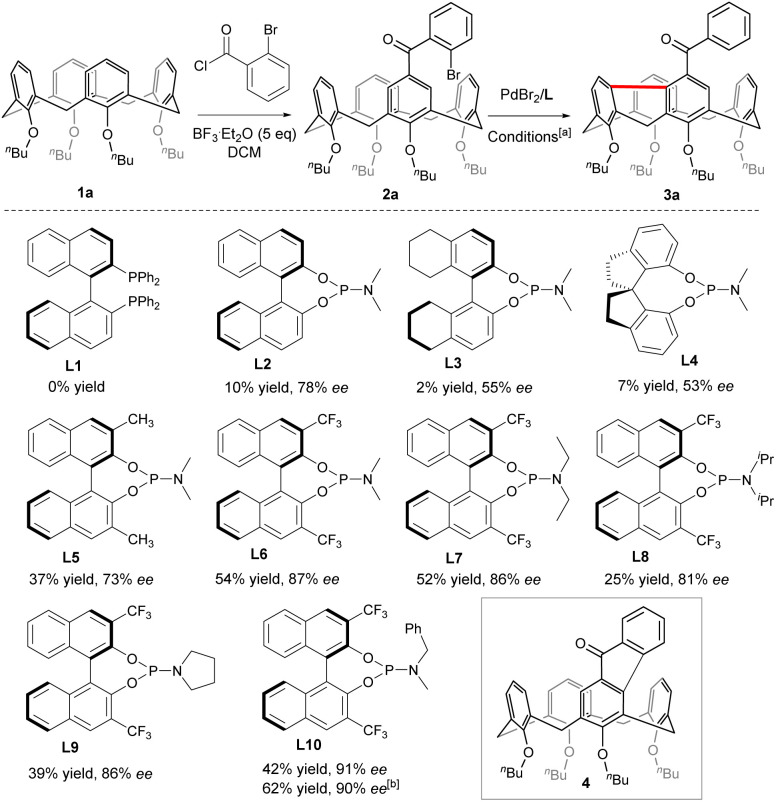
Structures of ligands. [a] Sealed tube, inert atmosphere, 2a (0.05 mmol), PdBr_2_ (0.1 eq.), L (0.2 eq.), Cs_2_CO_3_ (2 eq.), THF (1.5 mL), 110 °C; [b] PdBr_2_ (0.1 eq.), L10 (0.2 eq.), Rb_2_CO_3_ (3 eq.), under otherwise standard conditions.

To begin with, Friedel–Crafts acylation between symmetric calix[4]arene 1a and 2-bromobenzoyl chloride afforded our starting reagent 2a (Scheme 2). The catalytic enantioselective desymmetrizing reaction of 2a was initially investigated by varying the Pd-sauces, the ligands, the solvents, and the temperature (ESI†). PdBr_2_ stood out as the best Pd source. The nature of chiral ligands was found to impact not only the enantioselectivity of the reaction but also the product yield (Scheme 2). While a bidentate phosphine ligand such as *R*-BINAP (L1) failed to promote the reaction, chiral phosphoramidite derived from *R*-BINOL (L2) was able to affect the enantioselective transformation of 2a to afford 3a with 78% ee, albeit in a very low yield. Since either the enantioselectivity or the efficiency of the reaction was not improved when phosphoramidites prepared from *R*-[H_8_]BINOL (L3) and *S*-2,2′,3,3′-tetrahydro-1,1′-spirobi[indene]-7,7′-diol (L4) were employed as ligands, we focused on the chiral BINOL scaffold by introducing substituents on 3,3′-positions. In contrast to 3,3′-diaryl-substituted phosphoramidites which did not perform well due to most probably the steric hindrance (ESI†), 3,3′-dimethylated ligand L5 was able to increase the yield considerably while maintaining the same enantioselectivity. Both the chemical yield and enantiomeric excess value of 3a were improved remarkably when 3,3′-bis(trifluoromethyl)-substituted phosphoramidite (L6) was used as a ligand in combination with Cs_2_CO_3_ (54% yield, 87% ee). The amino part of the ligand was subsequently varied and phosphoramidite bearing an *N*-benzyl-*N*-methylamino group (L10) gave the best results. Finally, replacing CsCO_3_ with Rb_2_CO_3_ further increased the reaction efficiency. Overall, under optimized conditions [PdBr_2_ (10 mol%), L10 (20 mol%), Rb_2_CO_3_ (3.0 equiv.), THF, 110 °C], compound 2a was converted to 3a in 62% yield with 90% ee. A sequence of oxidative addition, enantioselective C–H activation/1,5-palladium migration^27,28^ followed by the second C–H activation/reductive elimination could account for the reaction outcome. The exclusive formation of 3a at the expense of 4 indicated that the 1,5-palladium migration and subsequent transannular arylation proceed much faster than the Csp^2^–Csp^2^ bond-forming reductive elimination from intermediate Int A (Scheme 1c). The facile transannular C_aryl_–H and C_aryl_–H cross-coupling reaction is most likely facilitated by the preferential cone conformation of the calixarene backbone.

### Scope of the reaction

**Scheme 3:**
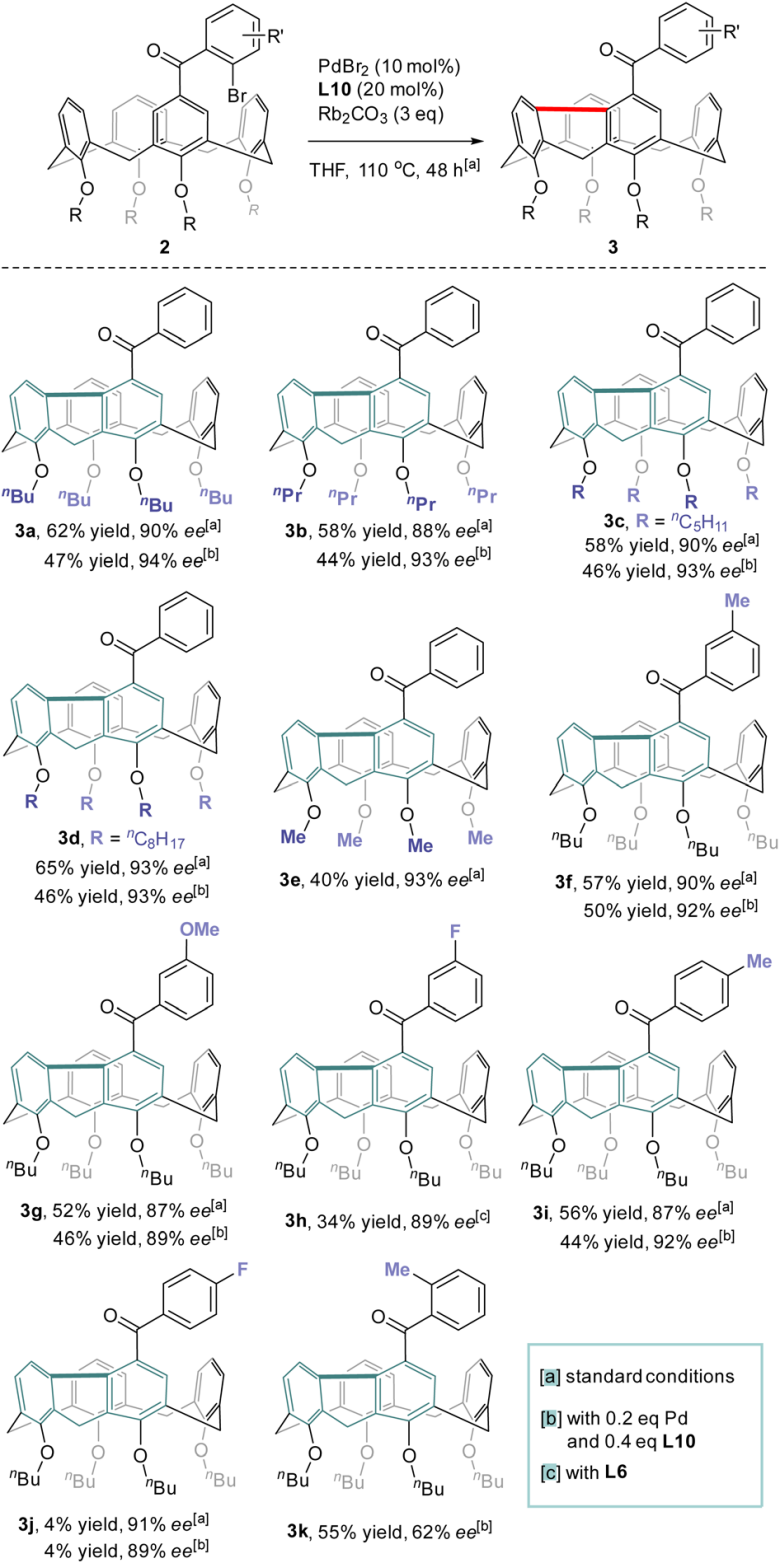
Scope of the reaction.

With the optimized conditions in hand, the reaction scope with regard to the structure of the lower-rim substituents of calix[4]arene was first examined (Scheme 3). Satisfyingly, substrates that contain alkoxy groups with different lengths of the alkyl chain such as *n*-Pr (3b), *n*-amyl (3c) and *n*-octyl (3d) were well accepted affording the desired products in good yields and enantiopurities. Noteworthily, when the alkyl substituent on each phenolic oxygen was replaced by methyl, the inherently chiral macrocycle 3e was obtained similarly with 93% ee. No racemization took place at an elevated temperature such as 150 °C (ESI†). Since an *O*-alkyl group larger than ethyl is generally required to stabilize the conformational structure of tetraethers of conventional calix[4]arenes,^29^ the isolation of highly enantioenriched compound 3e which contains only methoxy substituents at the lower-rim suggests that the rigidification of the macrocycle stemmed from the chemical bonding between proximal benzene rings. The formation of the 9*H*-fluorene unit also led to the contraction of the macrocyclic ring size which could prevent the methoxybenzene moieties from rotating or flipping around the macrocyclic annulus, the processes accounting for racemization. Substituents on 2-bromobenzoyl and substitution patterns subtly affected the outcomes of catalytic enantioselective desymmetrization. Reactants 2f–2h which bear respectively methyl, methoxy and fluorine groups on the *para*-position of bromine of 2-bromoaroyl acted as good substrates and their conversion to 3f–3h proceeded with the same high level of efficiency and enantiocontrol. Excellent enantioselectivity along with 44% yield was achieved for the transformation of 2i which has a methyl moiety *para* to the carbonyl. Surprisingly, substrate 2j having a fluorine *para* to the carbonyl appeared virtually inert to chiral catalysis with a large amount of starting material remaining intact. Moving methyl to the *ortho* position of the carbonyl, however, had a detrimental effect on enantioselectivity as the ee value of 3k dropped to 62%. A similarly high level of enantiocontrol of 1,5-Pd migration observed for most of the substrates 2a–2j except *ortho*-methyl-bearing reactant 2k implies the steric interaction between substrates and chiral catalyst may be crucial in chirality expression from a chiral catalyst to inherently chiral macrocycles.

### Structural elucidation

**Fig. 1:**
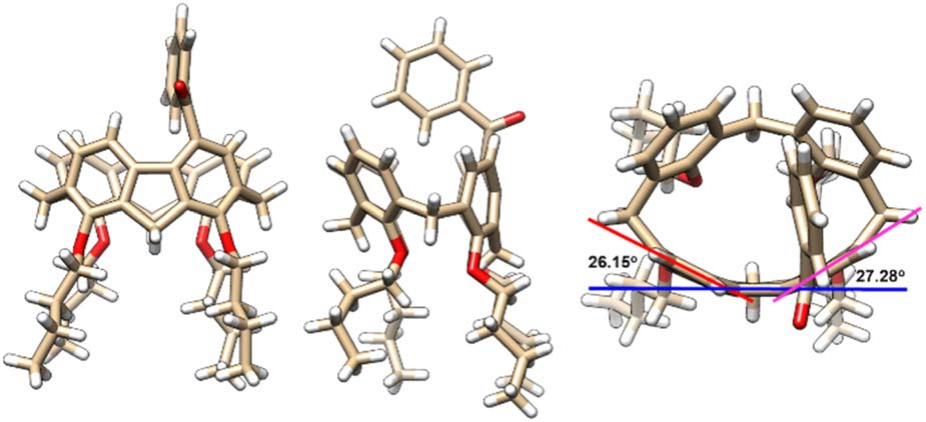
X-ray molecular structures of *P*-3a with side (left and middle) and top (right) views.

Molecular structures and their absolute configuration were determined unambiguously by crystallographic analysis of *P*-3a (Fig. 1). Similar to its calix[4]arene precursor, compound 3a adopts a cone conformation, indicating the stability of the conformational structure of the macrocycle during the course of palladation, 1,5-metal migration and transannular arylation. Noticeably, the newly formed 9*H*-fluorene fragment deviates severely from planarity.^30^ The dihedral angles between the five-membered ring and fused benzene rings are in the range of 26.15° and 27.38°, reflecting the ring strain of the macrocycle. The curved structure of 9*H*-fluorene results in the generation of an oval-shaped cavity.

### Derivatization of inherently chiral calixarenes

**Scheme 4:**
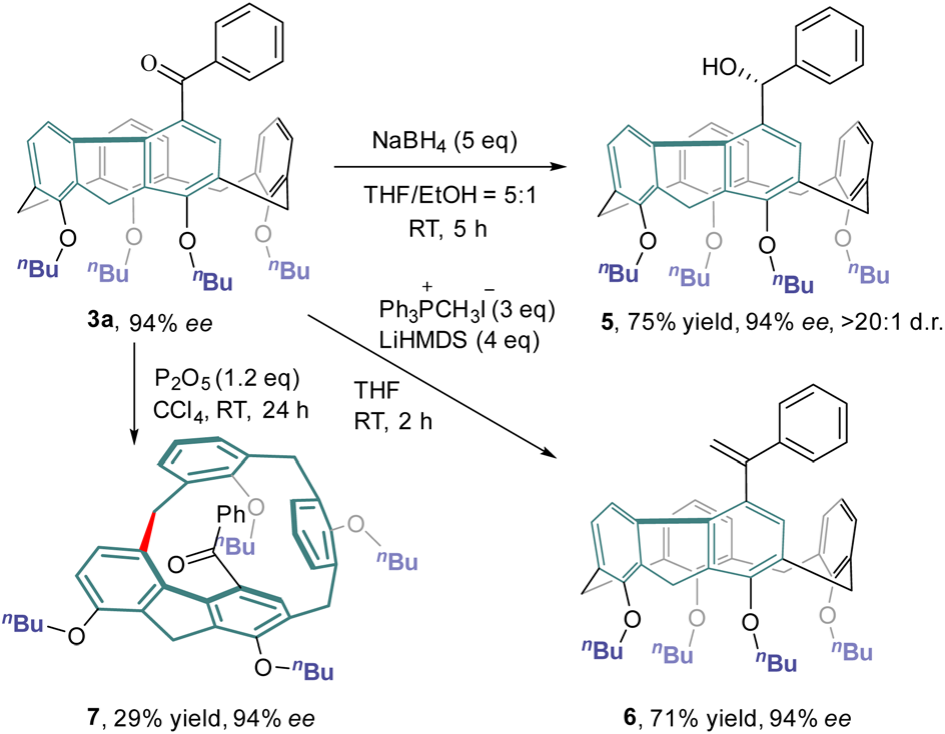
Synthetic transformation of inherently chiral calixarene 3a.

The chemical transformations of 3a taking advantage of the carbonyl group are depicted in Scheme 4. Treatment of 3a with an excess amount of NaBH_4_ at ambient temperature in a mixture of THF and ethanol produced alcohol 5 in 75% yield with a diastereomeric ratio of >20:1. The Wittig reaction of 3a with methylenetriphenylphosphorane provided olefin 6 in 71% yield. Both hydroxy and olefin functionalities would provide versatile handles for further elaboration of this new class of inherently chiral calixarenes. Interestingly, simply stirring a CCl_4_ solution of 3a at room temperature in the presence of P_2_O_5_ furnished inherently chiral macrocycle 7.^31^ No erosion of enantiopurity was observed in this transformation, indicating a probable concerted mechanism of macrocycle-to-macrocycle rearrangement.^25*b*,*c*^

### Chiroptical properties

**Fig. 2:**
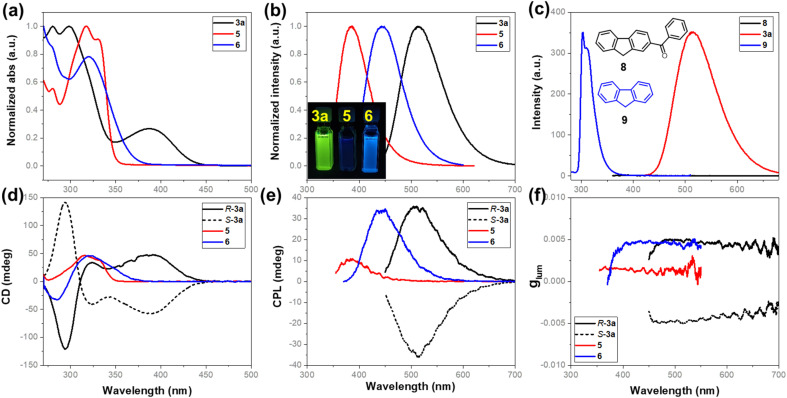
(a) UV/vis and (b) fluorescence spectra of 3a, 5, and 6 in CH_3_CN; (c) fluorescence spectra of 3a (2 × 10^−5^ M), 8 (2 × 10^−5^ M), and 9 (5 × 10^−6^ M) in CH_3_CN; (d) ECD and (e) CPL spectra of *P*-3a, *M*-3a, 5, and 6 in CH_3_CN (5 × 10^−5^ M); (f) luminescence dissymmetry factors *g*_lum_ of *P*-3a, *M*-3a, 5, and 6.

The acquired enantioenriched inherently chiral macrocycles 3 and their derivatives 5 and 6 exhibited promising chiroptical properties (Fig. 2 and ESI†). The UV–vis spectra of 3a and its analogues 3b–3k in acetonitrile show the lowest-energy absorption maximum at *ca.* 390 nm. When the carbonyl group was converted into hydroxyl (5) or a vinyl group (6), the absorption bands blue-shifted to 330 nm and 321 nm, respectively, indicating the fine-tuning of the properties by the substituents on the inherently chiral macrocycle skeleton. Relating to the UV–vis spectrum was the ECD spectrum which shows two positive Cotton effects at 391 and 325 nm and one negative effect at 285 nm for *P*-3a. Enantiomer *M*-3a gives the expected mirror-imaged ECD spectrum. For compounds 5 and 6, the positive Cotton effects at 390 nm disappear, while the other two (one positive and one negative) remain constant (Fig. 2d). Compound 3a was found to be fluorescent, giving a broad emission band (*ϕ*_F_ = 13.0%) centered at 514 nm upon excitation at 388 nm. Similarly, the hypsochromic shift of the emission band to 383 nm for 5 and 443 nm for 6 was observed (Fig. 2b). In addition, 3a showed pronounced positive solvatochromism in polar solvent (in toluene, *λ*_em_ = 476 nm; in CH_3_CN, *λ*_em_ = 514 nm, ESI†), indicating an excited state intramolecular charge transfer (ESICT) process. Notably, *P*-3a and *M*-3a appear strongly CPL active, giving emission maxima consistent with those observed in their fluorescence spectra. Under UV irradiation at 388 nm, for instance, *P*-3a and *M*-3a in acetonitrile gave complementary CPL spectra with large luminescence dissymmetry values of |*g*_lum_| = 5 × 10^−3^ at 515 nm. Interestingly, despite having a different conjugation system, the vinyl-bearing compound 6 has the same luminescence dissymmetry factor (|*g*_lum_| = 4.9 × 10^−3^) as ketone compound 3a. However, in the case of 5 in which conjugation was interrupted due to the reduction of carbonyl, the luminescence dissymmetry factor decreased sharply (|*g*_lum_| = 1.7 × 10^−3^) with the concomitant hypsochromic shift from 515 nm to 479 nm (Fig. 2e and f). These indicated that the post-modification on the inherently chiral calixarene skeleton, especially the tuning of conjugation of chromophores, could effectively regulate the chiroptical properties. It is worth addressing that benzoyl-substituted 9*H*-fluorene 8 and fluorene 9 show strikingly different photophysical properties. The former compound is not fluorescent at all due to probably a very fast intercrossing process while the latter emits fluorescence at 303 nm. The photophysical properties of compounds 3, 5 and 6 therefore resulted from the deformed 9*H*-fluorene chromophore, suggesting that the regulation of conformation of chromophores by means of forming strained macrocycles would provide a new method to fine-tune the photophysical properties of conventional aromatic chromophores.

## Conclusions

In conclusion, we have developed a new type of inherently chiral calixarene by the catalytic enantioselective transannular arene–arene dehydrogenative coupling reaction of calixarene derivatives. We have demonstrated that the 9*H*-fluorene-embedded inherently chiral calixarenes are useful scaffolds for the fabrication of CPL materials. The outcomes opened new opportunities for the design and synthesis of novel CPL materials based on the calixarene skeleton. The acknowledged versatile molecular recognition properties and the above-mentioned post-modification nature of calixarenes would render the resulting inherently chiral macrocycles a unique and lively chiroptical system.

## Data availability

The authors declare that the data supporting the findings of this study are available within the paper and the ESI,† as well as from the authors upon request.

## Author contributions

X. Z., S. T., and M. X. W. conceived and designed the experiments. X. Z. carried out the experiments. S. T., J. Z., and M. X. W. interpreted the results and co-wrote the manuscript.

## Conflicts of interest

There are no conflicts to declare.

## Supplementary Material

SC-014-D2SC06234H-s001

SC-014-D2SC06234H-s002
