# Supplementary material for: Inherently chiral calixarenes by a catalytic enantioselective desymmetrizing cross-dehydrogenative coupling
Source: Chem Sci. 2022 Dec 6;14(4):827–32. doi: 10.1039/d2sc06234h (PMC9890570; doi:10.1039/d2sc06234h)

Electronic Supplementary Material (ESI) for Chemical Science.
This journal is © The Royal Society of Chemistry 2022

Supporting information

Inherently Chiral Calixarenes by a Catalytic Enantioselective Desymmetrizing Cross-Dehydrogenative Coupling

Xin Zhang, Shuo Tong*, Jieping Zhu, Mei-Xiang Wang

Correspondence to: tongshuo@mail.tsinghua.edu.cn

Table of Contents

1. **General information**……S2
2. **Syntheis of chiral phosphoramidite ligands L6 and L10**……S4
3. **Possible reaction mechanism for the synthesis of 3a**……S6
4. **Optimization of reaction conditions for catalytic enantioselective synthesis of 3a**……S7
5. **General procedure for the synthesis of 2a-2k**……S10
6. **General procedure for the synthesis of 3a-3k**……S19
7. **Synthesis of 5**……S31
8. **Synthesis of 6**……S32
9. **Synthesis of 7**……S34
10. **Copies of HPLC chromatograms**……S36
11. **Inversion barrier test of 3e**……S51
12. **Crystallographic data of *P*-3a**……S52
13. **Crystallographic data of *rac*-7**……S54
14. **Copies of UV-vis spectra of 3a-3k, 5, 6, and 7**……S56
15. **Copies of fluorescence spectra of 3a-3k, 5, 6, and 7**……S79
16. **Copies of CD spectra of 3a-3k, 5, 6, and 7**……S102
17. **Copies of CPL spectra of 3a-3k, 5, 6, and 7**……S125
18. **Summary of Optical properties**……S169
19. **References**……S171
20. **Copies of ^{1}H and ^{13}C NMR spectra**……S172

1. General information

NMR spectra were recorded on a JEOL ECX-400 400 MHz NMR spectrometers. ^{1}H NMR chemical shifts were reported relative to residual $CDCl_3$ (7.26 ppm). ^{13}C NMR chemical shifts were reported relative to the central line of $CDCl_3$ (77.16 ppm). Abbreviations are used in the description of NMR data as follows: chemical shift (δ, ppm), multiplicity (s = singlet, d = doublet, t = triplet, q = quartet, dd = doublet of doublets, m = multiplet, and br = broad), coupling constant (J, Hz). The high resolution mass spectra (HRMS) were recorded on a GCT-MS Micromass UK spectrometer or a microTOF-Q spectrometer. Infrared spectra were recorded using a PerkinElmer Spectrum 100 FT-IR spectrometer with KBr pellets in the 4000-400 cm^{-1} region. All yields reported were isolated yields. Enantiomeric excesses were determined by HPLC using Daicel chiral stationary phase columns by comparing the samples with the appropriate racemic samples at 25 °C, column and elution details specified in each entry. The optical rotation was determined by Rudolph Autopol VI Automatic polarimeter. Crystallographic data were collected on a Rigaku XtaLAB Synergy (Cu) X-ray single crystal diffractometer. Melting points were uncorrected. Melting points were uncorrected.

Unless otherwise stated, reagents and solvents were purchased from commercial sources and preserved under argon. More sensitive compounds were stored in a desiccator or glove-box if required. Reagents were used without further purification unless otherwise noted. All reactions were performed under nitrogen and stirring unless otherwise noted. When needed oven dried glassware was used (T°>100 °C) or under vacuum with a heat gun (T°>200 °C). Anhydrous solvents were purified and dried following standard procedures. Reactions were monitored by thin layer chromatography (TLC) using Merck TLC silica gel 60 F254. Compounds were visualized by UV-light 254 nm and by dipping the plates in an ethanolic phosphomolybdic acid solution followed by heating. Flash column chromatography was performed over silica gel (230-400 mesh).

Optical properties were recorded in chromatographically pure grade solvent (acetonitrile, DCM, and toluene). UV-vis absorption spectra were recorded using an Agilent® Cary-5000 UV/Vis spectrophotometer at room temperature. Electronic circular dichroism (ECD) spectra were recorded on a JASCO J-815 spectropolarimeter at room temperature in a 1 cm-cuvette. Fluorescence spectra were recorded using an Agilent® Eclipse fluorescence spectrophotometer. Fluorescence quantum yields $\emptyset$ were measured in diluted solution with an optical density lower then 0.05 using the following equation:

$$\frac{\emptyset_x}{\emptyset_r} = \frac{A_r(\lambda)}{A_x(\lambda)} \times \frac{n_x^2}{n_r^2} \times \frac{D_x}{D_r}$$

Where A is the absorbance at the excitation wavelength (λ), n the refractive index and D the integrated intensity. r and x stand for reference and sample respectively. The fluorescence quantum yields were measured relative to quinine sulfatee, $\phi = 0.577$ in 0.1 M H_2SO_4, $\lambda_{ex} = 350$ nm. Excitation of reference and sample compounds was performed at the same wavelength. The circularly polarized luminescence (CPL) spectra were recorded in solution with a JASCO CPL-200 spectrometer at room temperature.

2. Synthesis of chiral phosphoramidite ligands L6 and L10.

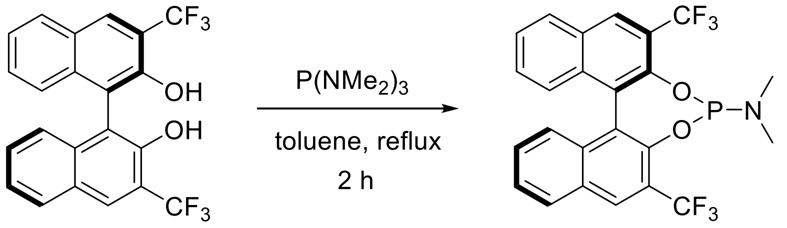

L6

L6 was prepared according to literature procedures[1].

To a solution of *R*-3,3'-bis(trifluoromethyl)-[1,1'-binaphthalene]-2,2'-diol[2] (127 mg, 0.3 mmol, 1 equiv) in toluene (2 mL) was added $P(NMe_2)_3$ (81 µL, 0.45 mmol, 1.5 equiv). After reflux for 2 h, the mixture was concentrated under reduced pressure. Purification by flash column chromatography (PE : CH_2Cl_2 = 10 : 1) provided **L6** (140 mg, 94% yield) as a foamy solid, **m.p.** = 90-92 °C. **^{1}H NMR** (400 MHz, $CDCl_3$, 298 K) δ 8.33 (s, 1H), 8.30 (s, 1H), 7.99 (d, *J* = 8.2 Hz, 2H), 7.53-7.48 (m, 2H), 7.38 (t, *J* = 8.4 Hz, 1H), 7.33 (t, *J* = 8.6 Hz, 1H), 7.26 (d, *J* = 8.5 Hz, 1H), 7.13 (d, *J* = 8.6 Hz, 1H), 2.50 (br s, 6H). **IR** (KBr) ν 3649, 2929, 2851, 1626, 1452, 1324, 1233, 1134, 847, 750 cm^{-1}. **HRMS** (ESI) m/z calcd. for $C_{24}H_{17}F_6NO_2P^+$ $[M+H]^+$ 496.08956; Found: 496.08884. **$[\alpha]_D^{27}$** = - 466 (*c* = 1.0, $CHCl_3$).

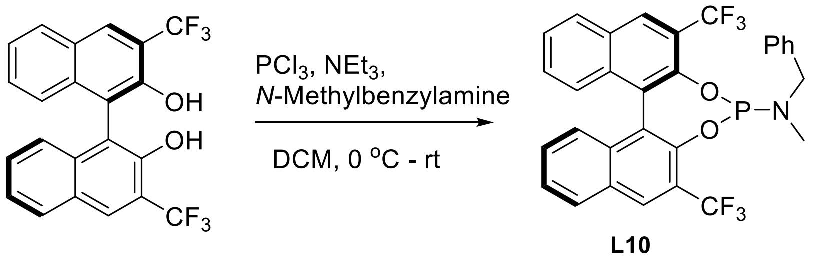

L10

L10 was prepared according to literature procedures[3].

Et_3N (208 µL, 1.5 mmol, 5 equiv) and *N*-Methylbenzylamine (39 µL, 0.3 mmol, 1 equiv) was added to a solution of PCl_3 (27 µL, 0.3 mmol, 1 equiv) in CH_2Cl_2 (1.5 mL) at 0 °C. The mixture was stirred for 5 h at rt, and a solution of *R*- 3,3'-bis(trifluoromethyl)-[1,1'-binaphthalene]-2,2'-diol (127 mg, 0.3 mmol, 1 equiv) in CH_2Cl_2 (1.5 mL) was added to

it. When the starting material was consumed by TLC analysis, the mixture was concentrated under reduced pressure. Purification by flash chromatography (PE : CH_2Cl_2 = 10 : 1) provided **L10** (110 mg, 64% yield) as a foamy solid, m.p. = 38-40 ^oC. **^{1}H NMR** (400 MHz, $CDCl_3$, 298 K) δ 8.38 (s, 1H), 8.35 (s, 1H), 8.01 (t, J = 7.2 Hz, 2H), 7.54-7.48 (m, 2H), 7.38-7.23 (m, 8H), 7.18 (d, J = 8.4 Hz, 1H), 4.04 (br s, 2H), 2.37 (br s, 3H). **IR** (KBr) v 3064, 2908, 1623, 1453, 1324, 1293, 1234, 1136, 1015, 847, 752 cm^{-1}. **HRMS** (ESI) m/z calcd. for $C_{30}H_{21}F_6NO_2P^+$ $[M+H]^+$ 572.12086; Found: 572.12006. $[\alpha]_D^{27}$ = - 412 (c = 1.0, $CHCl_3$).

3. Possible reaction mechanism for the synthesis of 3a

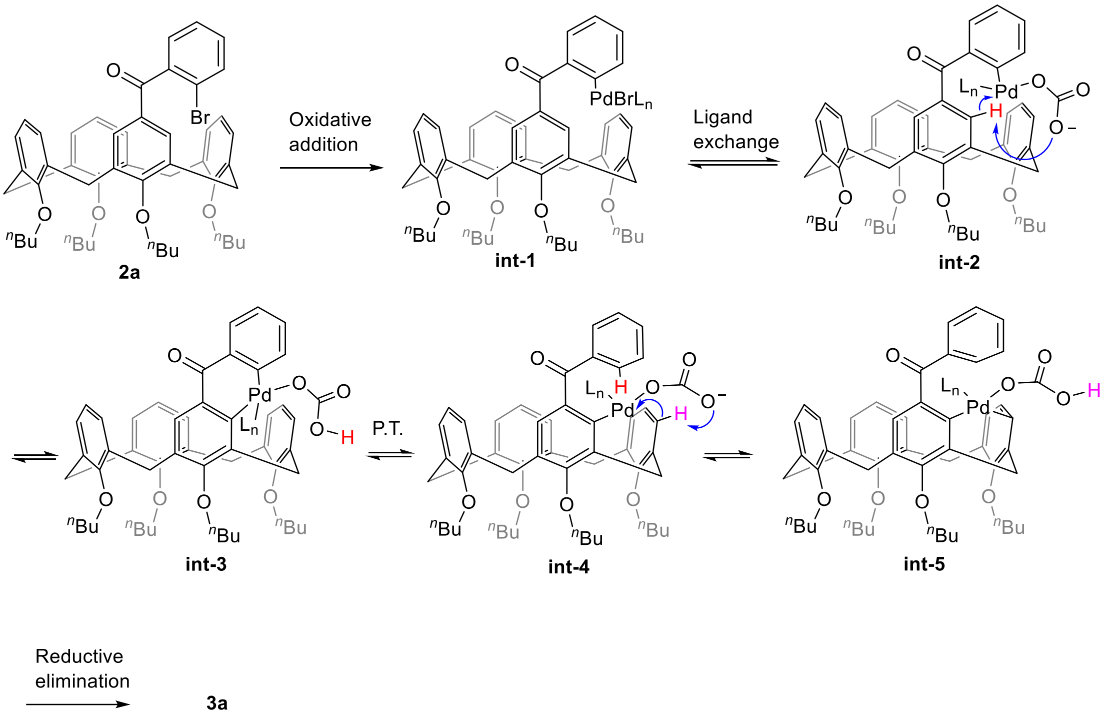

4. Optimization of reaction conditions for catalytic enantioselective synthesis of 3a

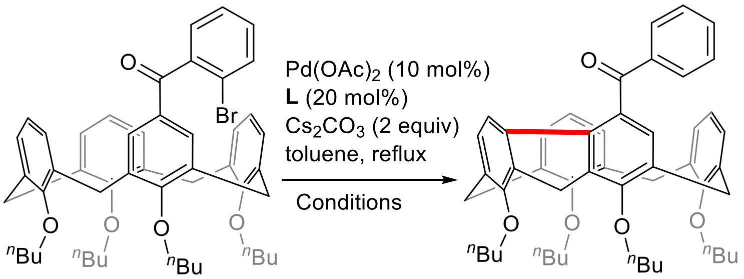

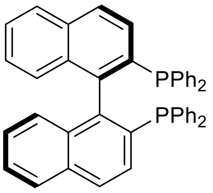

L1
14% yield, 0% *ee*[a]

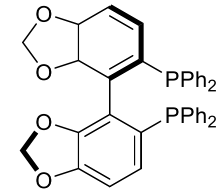

L11
5% yield, 0% *ee*[a]

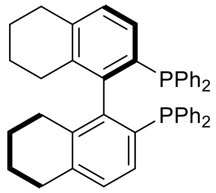

L12
12% yield, 0% *ee*[a]

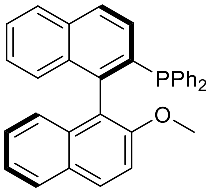

L13
3% yield, 0% *ee*[a]

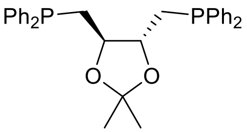

L14
0% yield[a]

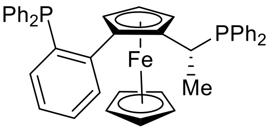

L15
0% yield[a]

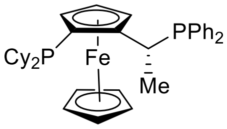

L16
0% yield[a]

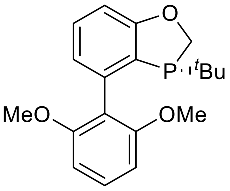

L17
4% yield, 0% *ee*[a]

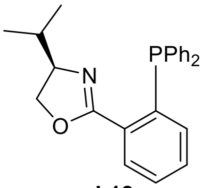

L18
3% yield, 0% *ee*[a]

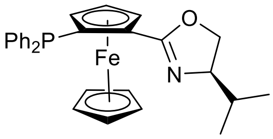

L19
1% yield, 9% *ee*[a]

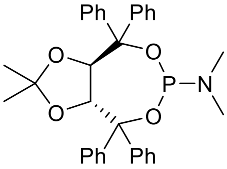

L20
0% yield[a]

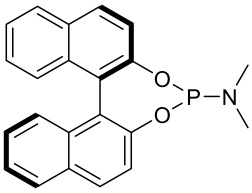

L2
4% yield, 47% *ee*[a]
47% yield, 47% *ee*[b]

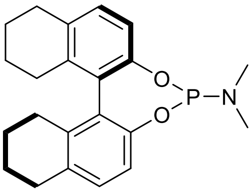

L3
10% yield, 11% *ee*[a]

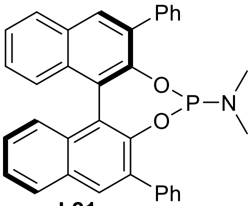

L21
14% yield, 13% *ee*[b]

Conditions: [a] Schlenk tube, inert atmosphere, toluene, reflux. [b] Sealed tube, inert atmosphere, toluene, 130 ^{o}C.

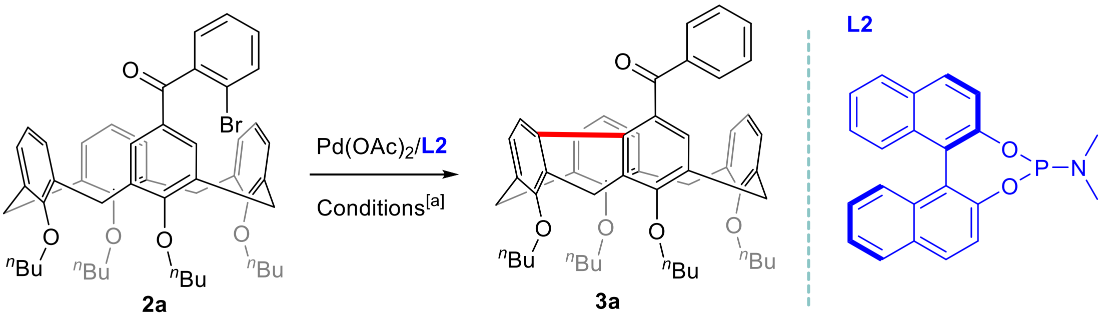

entry	catalyst (mol%)	Ligand (mol%)	base (eq)	solvent	temp. (°C)	yield (%)	*ee* (%)
1	$Pd(OAc)_2$ (10)	**L2** (20)	Cs_2CO_3 (2)	toluene	110	13	42
2	$Pd(OAc)_2$ (10)	**L2** (20)	Cs_2CO_3 (2)	toluene	120	19	35
3	$Pd(OAc)_2$ (10)	**L2** (20)	Cs_2CO_3 (2)	toluene	130	47	47
4	$Pd(OAc)_2$ (10)	**L2** (20)	Cs_2CO_3 (2)	toluene	140	49	31
5	$Pd(OAc)_2$ (20)	**L2** (40)	Cs_2CO_3 (2)	toluene	130	46	32
6	$Pd(OAc)_2$ (5)	**L2** (10)	Cs_2CO_3 (2)	toluene	130	31	37
7	$Pd(OAc)_2$ (10)	**L2** (20)	Cs_2CO_3 (1)	toluene	130	50	45
8	$Pd(OAc)_2$ (10)	**L2** (20)	Cs_2CO_3 (3)	toluene	130	51	45
9	$Pd(OAc)_2$ (10)	**L2** (20)	Cs_2CO_3 (2)	THF	130	42	62
10	$Pd(OAc)_2$ (10)	**L2** (20)	Cs_2CO_3 (2)	DMF	130	29	23
11	$Pd(OAc)_2$ (10)	**L2** (20)	Cs_2CO_3 (2)	dioxane	130	7	47
12	$Pd(OAc)_2$ (10)	**L2** (20)	Cs_2CO_3 (2)	xylenes	130	40	16
13	$Pd(OAc)_2$ (10)	**L2** (20)	Cs_2CO_3 (2)	^{t}amylOH	130	41	49
14	$Pd(OAc)_2$ (10)	**L2** (20)	Cs_2CO_3 (2)	^{t}BuOH	130	19	44
15	$Pd(OAc)_2$ (10)	**L2** (20)	K_2CO_3 (2)	THF	130	13	35
16	$Pd(OAc)_2$ (10)	**L2** (20)	K_3PO_4 (2)	THF	130	72	0
17	$Pd(OAc)_2$ (10)	**L2** (20)	KO^{t}Bu (2)	THF	130	21	25
18	$Pd(OAc)_2$ (10)	**L2** (20)	NaO^{t}Bu (2)	THF	130	25	24
19	$Pd(OAc)_2$ (10)	**L2** (20)	Cs_2CO_3 (2)	THF	140	52	15
20	$Pd(OAc)_2$ (10)	**L2** (20)	Cs_2CO_3 (2)	THF	120	12	67
21	$Pd_2(dba)_3$ (5)	**L2** (20)	Cs_2CO_3 (2)	THF	120	trace	60
22	$PdCl_2$ (10)	**L2** (20)	Cs_2CO_3 (2)	THF	120	12	47
23	$PdBr_2$ (10)	**L2** (20)	Cs_2CO_3 (2)	THF	120	17	75
24	$Pd(TFA)_2$ (10)	**L2** (20)	Cs_2CO_3 (2)	THF	120	14	66
25	$PdCl_2(MeCN)_2$ (10)	**L2** (20)	Cs_2CO_3 (2)	THF	120	6	70

Conditions: [a] Sealed tube, inert atmosphere, **2a** (0.05 mmol), $Pd(OAc)_2$, **L2**, solvent.

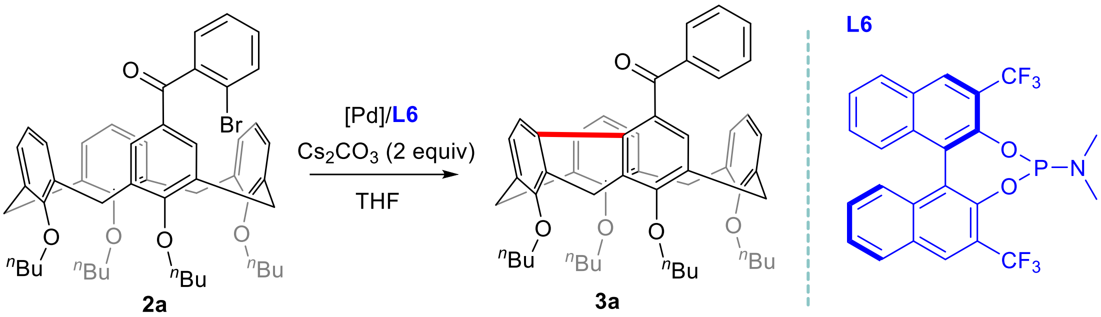

entry	catalyst (mol%)	Ligand (mol%)	temp. (°C)	yield (%)	*ee* (%)
1	$PdBr_2$ (10)	**L6** (20)	90	5	89
2	$PdBr_2$ (10)	**L6** (20)	100	26	87
3	$PdBr_2$ (10)	**L6** (20)	110	54	87
4	$PdBr_2$ (10)	**L6** (20)	120	38	86
5	$Pd_2(dba)_3$ (5)	**L6** (20)	110	22	75
6	$PdCl_2(MeCN)_2$ (10)	**L6** (20)	110	28	89
7	$PdBr_2$ (10)	**L6** (15)	110	31	84
8	$PdBr_2$ (20)	**L6** (30)	110	39	70
9	$PdBr_2$ (20)	**L6** (40)	110	50	89
10	$PdBr_2$ (30)	**L6** (60)	110	37	88

5. General procedure for the synthesis of 2a-2k

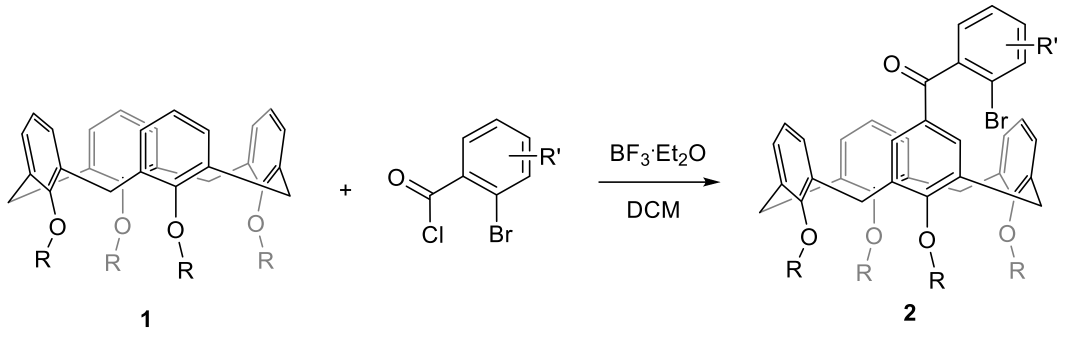

$BF_3·Et_2O$ (1.24 mL, 10 mmol, 5 equiv) and *o*-bromobenzoyl chloride (4 mmol, 2 equiv) was simultaneously added to a solution of calix[4]arenes (2 mmol, 1 equiv) in CH_2Cl_2 (20 mL) at room temperature. After stirred at 30 °C for 24 h, The reaction mixture was quenched with saturated $NaHCO_3$ aqueous solution and extracted with CH_2Cl_2. The combined organic layers were washed with brine (3 × 50 mL) and dried over anhydrous Na_2SO_4. Solvents were removed in *vacuo* and the residue was purified by flash column chromatography on silica gel (PE : EA = 40 : 1) to give the pure product **2**.

(2-bromophenyl)(1^2,3^2,5^2,7^2-tetrabutoxy-1,3,5,7(1,3)-tetrabenzenacyclooctaphane-1^5-yl)methanone (**2a**)

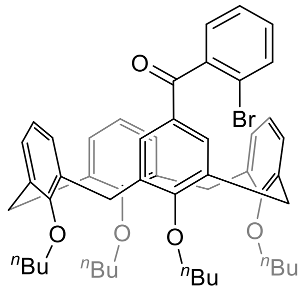

Chemical Formula: $C_{51}H_{59}BrO_5$; Molecular Weight: 831.9320.

710 mg, 43% yield, white foamy solid. **m.p.** = 56-58 °C. **^{1}H NMR** (400 MHz, $CDCl_3$, 298 K) δ 7.66-7.57 (m, 1H), 7.34-7.27 (m, 2H), 7.11 (s, 2H), 6.97-6.90 (m, 1H), 6.75-6.50 (m, 9H), 4.46 (d, J = 13.3 Hz, 4H), 4.01-3.83 (m, 8H), 3.18 (d, J = 13.3 Hz, 2H), 3.17 (d, J = 13.4 Hz, 2H), 1.98-1.85 (m, 8H), 1.53-1.38 (m, 8H), 1.01 (t, J = 7.4 Hz, 12H). **^{13}C NMR** (100 MHz, $CDCl_3$, 298 K) δ 194.8, 161.9, 156.6, 140.7, 135.6, 135.5, 135.1, 134.6, 133.3, 131.2, 130.8, 130.1, 129.7, 128.7, 128.34, 128.30, 126.7, 122.5,

122.1, 120.1, 75.3, 75.2, 75.0, 32.53, 32.50, 32.4, 31.10, 31.05, 19.53, 19.47, 14.22, 14.15. **IR** (KBr) *v* 3359, 2958, 2922, 2870, 1665, 1588, 1456, 1288, 1206, 1114, 761 cm^{-1}. **HRMS** (ESI) m/z calcd. for $C_{51}H_{60}BrO_5^+$ $[M+H]^+$ 831.36186; Found: 831.36328.

(2-bromophenyl)(1^2,3^2,5^2,7^2-tetrapropoxy-1,3,5,7(1,3)-tetrabenzenacyclooctaphane-1^5-yl)methanone (**2b**)

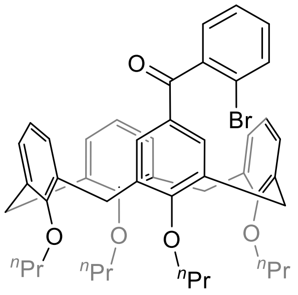

Chemical Formula: $C_{47}H_{51}BrO_5$; Molecular Weight: 775.8240.

620 mg, 40% yield, white foamy solid. **m.p.** = 59-62 °C. **^{1}H NMR** (400 MHz, $CDCl_3$, 298 K) δ 7.63-7.57 (m, 1H), 7.34-7.27 (m, 2H), 7.10 (s, 2H), 6.95-6.91 (m, 1H), 6.71-6.50 (m, 9H), 4.45 (d, J = 13.4 Hz, 4H), 3.96-3.78 (m, 8H), 3.17 (d, J = 13.3 Hz, 2H), 3.16 (d, J = 13.4 Hz, 2H), 2.00-1.86 (m, 8H), 1.04-0.95 (m, 12H). **^{13}C NMR** (100 MHz, $CDCl_3$, 298 K) δ 194.8, 161.9, 156.6, 140.6, 135.6, 135.4, 135.07, 134.6, 133.3, 131.2, 130.8, 130.0, 129.7, 128.7, 128.32, 128.27, 126.7, 122.5, 122.1, 120.1, 31.1, 31.0, 23.5, 23.4, 23.3, 10.50, 10.47, 10.4. **IR** (KBr) *v* 3359, 2963, 2932, 2875, 1652, 1555, 1453, 1273, 1212, 1090, 755 cm^{-1}. **HRMS** (ESI) m/z calcd. for $C_{47}H_{52}BrO_5^+$ $[M+H]^+$ 775.29926; Found: 775.30042.

(2-bromophenyl)(1^2,3^2,5^2,7^2-tetrakis(pentyloxy)-1,3,5,7(1,3)-tetrabenzenacyclooctaphane-1^5-yl)methanone (**2c**)

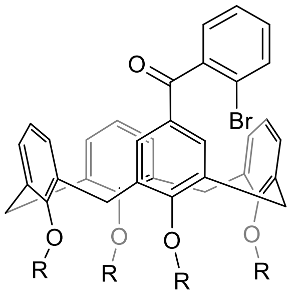

Chemical Formula: $C_{55}H_{67}BrO_5$; Molecular Weight: 888.0400.

650 mg, 37% yield, colourless solid. **m.p.** = 26-28 °C. **^{1}H NMR** (400 MHz, $CDCl_3$, 298 K) δ 7.62-7.60 (m, 1H), 7.32-7.27 (m, 2H), 7.11 (s, 2H), 6.96-6.93 (m, 1H), 6.69-6.64 (m, 4H), 6.59-6.52 (m, 5H), 4.45 (d, *J* = 13.3 Hz, 4H), 3.97-3.84 (m, 8H), 3.17 (d, *J* = 13.6 Hz, 2H), 3.16 (d, *J* = 13.6 Hz, 2H), 1.96-1.90 (m, 8H), 1.41-1.39 (m, 16H), 0.95 (t, *J* = 6.7 Hz, 12H). **^{13}C NMR** (100 MHz, $CDCl_3$, 298 K) δ 194.8, 161.9, 156.7, 156.6, 140.7, 135.7, 135.5, 135.1, 134.6, 133.3, 131.2, 130.8, 130.0, 129.7, 128.7, 128.32, 128.29, 126.7, 122.5, 122.1, 120.1, 75.6, 75.5, 75.3, 31.13, 31.09, 30.2, 30.15, 30.05, 28.6, 28.52, 28.46, 23.0, 22.9, 14.35, 14.31, 14.28. **IR** (KBr) ν 3360, 2956, 2928, 2861, 1666, 1588, 1457, 1288, 1200, 1114, 1007, 760 cm^{-1}. **HRMS** (ESI) m/z calcd. for $C_{55}H_{68}BrO_5^+$ $[M+H]^+$ 887.42446; Found: 887.42572.

(2-bromophenyl)(1^2,3^2,5^2,7^2-tetrakis(octyloxy)-1,3,5,7(1,3)-tetrabenzenacyclooctaphane-1^5-yl)methanone (**2d**)

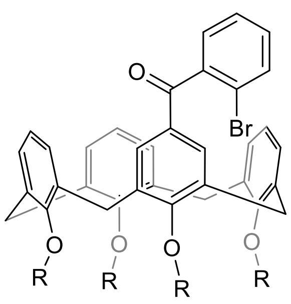

Chemical Formula: $C_{67}H_{91}BrO_5$; Molecular Weight: 1056.3640.

1010 mg, 48% yield, colorless oil. **^{1}H NMR** (400 MHz, $CDCl_3$, 298 K) δ 7.62-7.60 (m, 1H), 7.32-7.27 (m, 2H), 7.10 (s, 2H), 6.95-6.92 (m, 1H), 6.69-6.63 (m, 4H), 6.59-6.52

(m, 5H), 4.44 (d, J = 13.3 Hz, 4H), 3.96-3.84 (m, 8H), 3.17 (d, J = 13.6 Hz, 2H), 3.16 (d, J = 13.6 Hz, 2H), 1.93-1.89 (m, 8H), 1.37-1.30 (m, 40H), 0.91-0.88 (m, 12H). **^{13}C NMR** (100 MHz, $CDCl_3$, 298 K) δ 194.8, 161.9, 156.7, 156.6, 140.8, 135.7, 135.5, 135.1, 134.6, 133.3, 131.2, 130.8, 130.1, 129.7, 128.7, 128.4, 128.3, 126.7, 122.5, 122.1, 120.1, 75.6, 75.5, 75.4, 32.1, 31.2, 31.1, 30.59, 30.55, 30.5, 30.07, 30.05, 30.0, 29.8, 29.74, 29.70, 26.54, 26.50, 26.45, 22.9, 14.2. **IR** (KBr) v 3057, 2955, 2924, 2854, 1667, 1588, 1458, 1288, 1208, 1115, 1010, 761 cm^{-1}. **HRMS** (ESI) m/z calcd. for $C_{67}H_{92}BrO_5^+$ [M+H]$^+$ 1055.61226; Found: 1055.61389.

(2-bromophenyl)(1^2,3^2,5^2,7^2-tetramethoxy-1,3,5,7(1,3)-tetrabenzenacyclooctaphane-1^5-yl)methanone (**2e**)

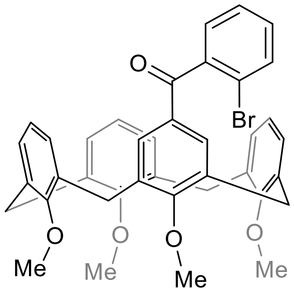

Chemical Formula: $C_{39}H_{35}BrO_5$; Molecular Weight: 663.6080.

440 mg, 33% yield, white foamy solid. **m.p.** = 60-63 ^{o}C. **^{1}H NMR** (400 MHz, $CDCl_3$, 298 K) δ 7.79-7.55 (m, 2H), 7.38-7.21 (m, 3H), 7.06-6.90 (m, 6H), 6.82-6.77 (m, 1H), 6.73-6.63 (m, 1H), 6.53-6.46 (m, 2H), 4.37 (dd, J = 13.1, 4.0 Hz, 1H), 4.10-4.07 (m, 2H), 3.87-3.58 (m, 12H), 3.24-3.02 (m, 5H). **^{13}C NMR** (100 MHz, $CDCl_3$, 298 K), recorded as conformational isomers, δ 193.0, 158.1, 157.7, 140.6, 136.8, 135.8, 135.2, 135.0, 134.5, 134.0, 133.9, 133.4, 133.2, 132.9, 132.3, 132.1, 131.1, 131.0, 130.5, 129.4, 128.7, 128.5, 128.1, 126.6, 126.3, 122.9, 122.3, 121.9, 62.1, 61.4, 61.2, 60.9, 59.8, 58.9, 36.1, 35.8, 30.7. **IR** (KBr) v 3315, 2927, 2821, 1664, 1588, 1466, 1426, 1292, 1207, 1115, 764 cm^{-1}. **HRMS** (ESI) m/z calcd. for $C_{39}H_{36}BrO_5^+$ [M+H]$^+$ 663.17406; Found: 663.17578.

(2-bromo-5-methylphenyl)(1^2,3^2,5^2,7^2-tetrabutoxy-1,3,5,7(1,3)-tetrabenzenacyclooctaphane-1^5-yl)methanone (**2f**)

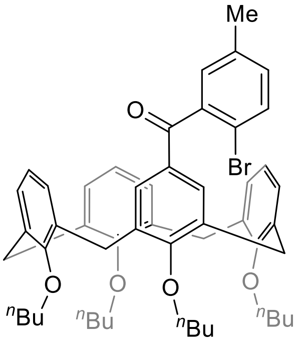

Chemical Formula: $C_{52}H_{61}BrO_5$; Molecular Weight: 845.9590.

560 mg, 33% yield, white foamy solid. **m.p.** = 85-88 °C. **^{1}H NMR** (400 MHz, $CDCl_3$, 298 K) δ 7.48-7.46 (m, 1H), 7.14 (s, 2H), 7.11 (d, J = 8.2 Hz, 1H), 6.84 (s, 1H), 6.70-6.55 (m, 9H), 4.46 (d, J = 13.2 Hz, 4H), 3.99-3.87 (m, 8H), 3.18 (d, J = 13.2 Hz, 4H), 2.34 (s, 3H), 1.96-1.87 (m, 8H), 1.53-1.40 (m, 8H), 1.01 (t, J = 7.4 Hz, 12H). **^{13}C NMR** (100 MHz, $CDCl_3$, 298 K) δ 195.1, 162.0, 156.64, 156.57, 140.8, 136.8, 135.7, 135.5, 135.2, 134.6, 132.9, 131.6, 131.2, 130.0, 129.5, 128.6, 128.4, 128.3, 122.4, 121.9, 116.5, 75.3, 75.1, 75.0, 32.53, 32.48, 32.4, 31.1, 31.0, 21.1, 19.52, 19.47, 19.45, 14.23, 14.16. **IR** (KBr) ν 3358, 2958, 2923, 2871, 1663, 1590, 1457, 1245, 1114, 1022, 761 cm^{-1}. **HRMS** (ESI) m/z calcd. for $C_{52}H_{62}BrO_5^+$ $[M+H]^+$ 845.37751; Found: 845.37915.

(2-bromo-5-methoxyphenyl)(1^2,3^2,5^2,7^2-tetrabutoxy-1,3,5,7(1,3)-tetrabenzenacyclooctaphane-1^5-yl)methanone (**2g**)

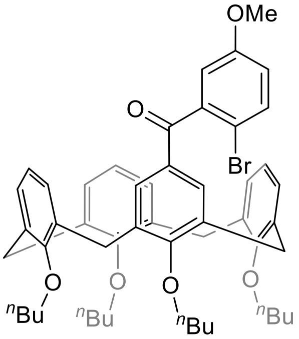

Chemical Formula: $C_{52}H_{61}BrO_6$; Molecular Weight: 861.9580.

610 mg, 35% yield, white foamy solid. **m.p.** = 61-64 °C. **^{1}H NMR** (400 MHz, $CDCl_3$, 298 K) δ 7.48 (dd, J = 8.8, 0.7 Hz, 1H), 7.22 (s, 2H), 6.87 (ddd, J = 8.8, 3.0, 0.8 Hz, 1H), 6.73 (d, J = 7.4 Hz, 2H), 6.65-6.57 (m, 4H), 6.55-6.52 (m, 4H), 4.45 (d, J = 13.4

Hz, 4H), 4.01 (t, *J* = 7.4 Hz, 2H), 3.93-3.84 (m, 6H), 3.82 (s, 3H), 3.18 (d, *J* = 13.6 Hz, 2H), 3.17 (d, *J* = 13.6 Hz, 2H), 1.95-1.87 (m, 8H), 1.52-1.40 (m, 8H), 1.01 (t, *J* = 7.4 Hz, 12H). **^{13}C NMR** (100 MHz, $CDCl_3$, 298 K) δ 194.9, 162.3, 158.5, 156.8, 156.3, 141.8, 136.0, 135.4, 135.1, 134.1, 133.8, 131.2, 129.7, 128.52, 128.48, 128.1, 122.4, 122.0, 116.6, 114.6, 110.0, 75.3, 75.1, 75.0, 55.7, 32.5, 32.42, 32.40, 31.04, 30.99, 19.50, 19.45, 19.4, 14.24, 14.22, 14.18. **IR** (KBr) ν 3058, 2958, 2930, 2871, 1667, 1589, 1458, 1288, 1196, 1112, 1025, 761 cm^{-1}. **HRMS** (ESI) m/z calcd. for $C_{52}H_{62}BrO_6^+$ $[M+H]^+$ 861.37243; Found: 861.37432.

(2-bromo-5-fluorophenyl)(1^2,3^2,5^2,7^2-tetrabutoxy-1,3,5,7(1,3)-tetrabenzenacyclooctaphane-1^5-yl)methanone (**2h**)

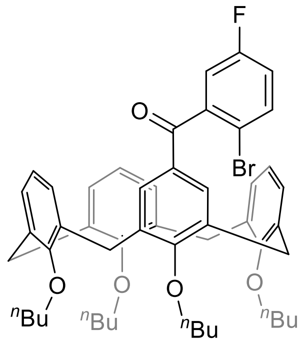

Chemical Formula: $C_{51}H_{58}BrFO_5$; Molecular Weight: 849.9224.

200 mg, 12% yield, white foamy solid. **m.p.** = 55-58 °C. **^{1}H NMR** (400 MHz, $CDCl_3$, 298 K) δ 7.55 (dd, *J* = 8.8, 4.9 Hz, 1H), 7.05-7.00 (m, 3H), 6.79 (dd, *J* = 6.8, 2.0 Hz, 2H), 6.68-6.60 (m, 7H), 6.51-6.48 (m, 1H), 4.45 (d, *J* = 13.2 Hz, 2H), 4.44 (d, *J* = 13.2 Hz, 2H), 4.02-3.88 (m, 6H), 3.84 (t, *J* = 7.2 Hz, 2H), 3.18 (d, *J* = 13.2 Hz, 2H), 3.17 (d, *J* = 13.2 Hz, 2H), 1.97-1.85 (m, 8H), 1.53-1.37 (m, 8H), 1.02-0.98 (m, 12H). **^{13}C NMR** (100 MHz, $CDCl_3$, 298 K) δ 193.3, 161.9, 161.2 (d, *J* = 249.5 Hz),156.7, 156.3, 142.2 (d, *J* = 6.2 Hz), 135.8, 135.6, 134.8, 134.73, 134.65, 131.1, 129.4, 128.9, 128.4, 128.3, 122.5, 122.0, 118.0 (d, *J* = 22.5 Hz), 116.7 (d, *J* = 23.8 Hz), 114.2 (d, *J* = 3.0 Hz), 77.5, 77.2, 76.8, 75.4, 75.2, 75.0, 32.5, 32.3, 31.1, 31.0, 19.6, 19.5, 19.4, 14.3, 14.2, 14.1.. **IR** (KBr) ν 3332, 3061, 2958, 2930, 2871, 1669, 1589, 1458, 1246, 1195, 1021, 762 cm^{-1}. **HRMS** (ESI) m/z calcd. for $C_{51}H_{59}BrFO_5^+$ $[M+H]^+$ 849.35244; Found: 849.35321.

(2-bromo-4-methylphenyl)(1^2,3^2,5^2,7^2-tetrabutoxy-1,3,5,7(1,3)-tetrabenzenacyclooctaphane-1^5-yl)methanone (**2i**)

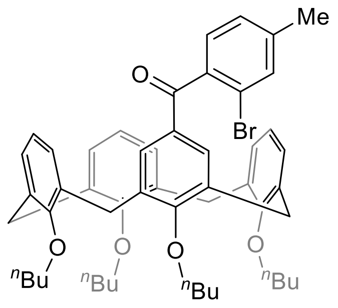

Chemical Formula: $C_{52}H_{61}BrO_5$; Molecular Weight: 845.9590.

420 mg, 25% yield, white foamy solid. **m.p.** = 67-69 ^oC. **^{1}H NMR** (400 MHz, $CDCl_3$, 298 K) δ 7.44 (s, 1H), 7.13 (s, 2H), 7.07 (d, *J* = 8.6 Hz, 1H), 6.83 (d, *J* = 7.7 Hz, 1H), 6.68-6.65 (m, 4H), 6.60-6.52 (m, 5H), 4.45 (d, *J* = 13.2 Hz, 2H), 4.44 (d, *J* = 13.2 Hz, 2H), 3.98-3.85 (m, 8H), 3.17 (d, *J* = 13.2 Hz, 2H), 3.16 (d, *J* = 13.2 Hz, 2H), 1.95-1.86 (m, 8H), 1.50-1.40 (m, 8H), 1.00 (t, *J* = 7.4 Hz, 12H). **^{13}C NMR** (100 MHz, $CDCl_3$, 298 K) δ 194.9, 161.8, 156.7, 156.5, 141.4, 137.6, 135.6, 135.4, 135.2, 134.6, 133.9, 131.2, 130.4, 129.9, 128.6, 128.4, 128.3, 127.4, 122.4, 122.1, 120.1, 75.3, 75.2, 75.0, 32.52, 32.49, 32.4, 31.10, 31.06, 21.2, 19.52, 19.49, 19.46, 14.22, 14.15. **IR** (KBr) ν 3361, 2929, 2871, 1664, 1597, 1456, 1308, 1288, 1206, 1117, 761 cm^{-1}. **HRMS** (ESI) m/z calcd. for $C_{52}H_{62}BrO_5^+$ $[M+H]^+$ 845.37751; Found: 845.37891.

(2-bromo-4-fluorophenyl)(1^2,3^2,5^2,7^2-tetrabutoxy-1,3,5,7(1,3)-tetrabenzenacyclooctaphane-1^5-yl)methanone (**2j**)

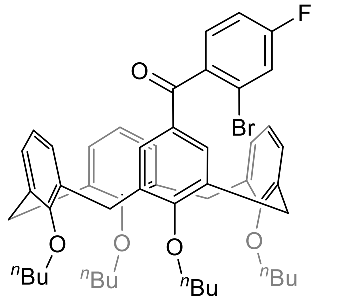

Chemical Formula: $C_{51}H_{58}BrFO_5$; Molecular Weight: 849.9224.

340 mg, 20% yield, white foamy solid. **m.p.** = 60-62 °C. ^{1}**H NMR** (400 MHz, $CDCl_3$, 298 K) δ 7.36 (dd, J = 8.4, 2.4 Hz, 1H), 7.02 (s, 2H), 6.96 (td, J = 8.3, 2.4 Hz, 1H), 6.84 (dd, J = 8.5, 6.0 Hz, 1H), 6.79 (dd, J = 6.9, 1.8 Hz, 2H), 6.70-6.64 (m, 4H), 6.58 (d, J = 7.5 Hz, 2H), 6.48-6.44 (m, 1H), 4.46 (d, J = 13.2 Hz, 4H), 4.03-3.89 (m, 6H), 3.85 (t, J = 7.2 Hz, 2H), 3.19 (d, J = 13.2 Hz, 2H), 3.18 (d, J = 13.2 Hz, 2H), 1.98-1.86 (m, 8H), 1.54-1.38 (m, 8H), 1.01 (t, J = 7.4 Hz, 12H). ^{13}**C NMR** (100 MHz, $CDCl_3$, 298 K) δ 193.7, 162.8 (d, J = 253.8 Hz), 161.7, 156.7, 156.4, 136.5 (d, J = 3.6 Hz), 135.8, 135.4, 134.9, 134.8, 131.5 (d, J = 8.8 Hz), 131.1, 130.0, 128.8, 128.5, 128.1, 122.5, 122.0, 121.2 (d, J = 9.7 Hz), 120.8 (d, J = 24.4 Hz), 113.8 (d, J = 21.2 Hz), 75.4, 75.2, 75.0, 32.5, 32.3, 31.1, 31.0, 19.6, 19.5, 19.4, 14.24, 14.19, 14.1. **IR** (KBr) ν 3359, 3059, 2958, 2929, 2871, 1667, 1594, 1457, 1210, 1114, 762 cm^{-1}. **HRMS** (ESI) m/z calcd. for $C_{51}H_{59}BrFO_5^+$ $[M+H]^+$ 849.35244; Found: 849.35382.

(2-bromo-6-methylphenyl)(1^{2},3^{2},5^{2},7^{2}-tetrabutoxy-1,3,5,7(1,3)-tetrabenzenacyclooctaphane-1^{5}-yl)methanone (**2k**)

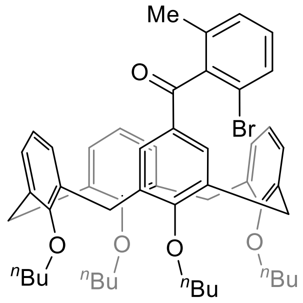

Chemical Formula: $C_{52}H_{61}BrO_5$; Molecular Weight: 845.9590.

760 mg, 45% yield, white foamy solid. **m.p.** = 68-71 °C. ^{1}**H NMR** (400 MHz, $CDCl_3$, 298 K) δ 7.42 (d, J = 7.3 Hz, 1H), 7.35 (br s, 1H), 7.20-7.07 (m, 3H), 6.73 (br d, J = 6.9 Hz, 2H), 6.61-6.46 (m, 6H), 6.38 (br s, 1H), 4.43 (d, J = 13.2 Hz, 4H), 4.02-3.98 (m, 2H), 3.92-3.81 (m, 6H), 3.16 (d, J = 13.2 Hz, 4H), 1.96-1.86 (m, 8H), 1.52-1.39 (m, 8H), 1.02-0.97 (m, 12H). ^{13}**C NMR** (100 MHz, $CDCl_3$, 298 K) δ 196.3, 162.4, 156.8, 156.2, 141.1, 137.2, 136.3, 135.4, 135.0, 134.1, 131.2, 130.0, 129.9, 129.8, 129.0, 128.6, 128.5, 127.9, 122.5, 122.1, 119.1, 75.4, 75.2, 75.0, 32.6, 32.44, 32.41, 31.1, 31.0, 20.0, 19.54, 19.46, 19.4, 14.24, 14.21, 14.18. **IR** (KBr) ν 3430, 3057, 2958, 2930, 2871, 1667,

1589, 1456, 1245, 1206, 1110, 761 cm^{-1}. **HRMS** (ESI) m/z calcd. for $C_{52}H_{62}BrO_5^+$ $[M+H]^+$ 845.37751; Found: 845.37946.

6. General procedure for the synthesis of 3a-3k

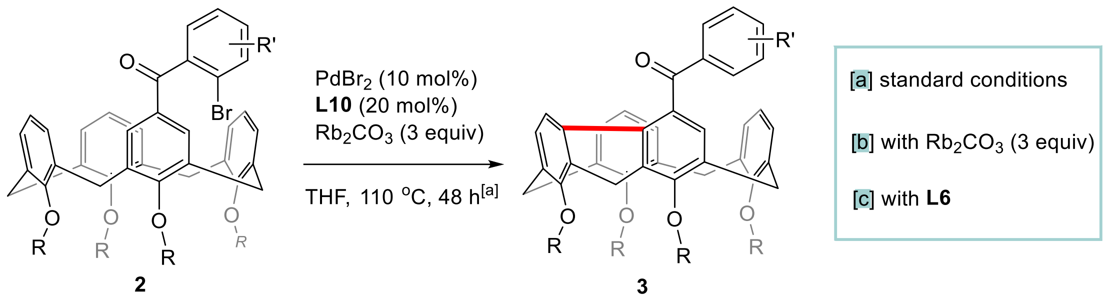

General procedure A: To a 10 mL Schlenk-type sealed tube was added **2** (0.05 mmol), $PdBr_2$ (1.4 mg, 0.005 mmol, 10 mol%), **L10** (5.7 mg, 0.01 mmol, 20 mol%), Rb_2CO_3 (34.7 mg, 0.15 mmol, 3 equiv), and dry THF (1.5 mL). The reaction tube was purged with N_2, sealed with a Teflon cap and stirred at 110 ^oC for 48 h. The reaction was cooled to room temperature. The reaction mixture was filtered through Celite and the filtrate was concentrated in *vacuo*. The residue was purified by preparative TLC to afford desired product.

General procedure B: To a 10 mL Schlenk-type sealed tube was added **2** (0.05 mmol), $PdBr_2$ (2.7 mg, 0.01 mmol, 20 mol%), **L10** (11.4 mg, 0.02 mmol, 40 mol%), Rb_2CO_3 (34.7 mg, 0.15 mmol, 3 equiv), and dry THF (1.5 mL). The reaction tube was purged with N_2, sealed with a Teflon cap and stirred at 110 ^oC for 24 h. The reaction was cooled to room temperature. The reaction mixture was filtered through Celite and the filtrate was concentrated in *vacuo*. The residue was purified by preparative TLC to afford desired product.

General procedure C: To a 10 mL Schlenk-type sealed tube was added **2** (0.05 mmol), $PdBr_2$ (1.4 mg, 0.005 mmol, 10 mol%), **L6** (4.9 mg, 0.01 mmol, 20 mol%), Cs_2CO_3 (32.5 mg, 0.1 mmol, 2 equiv), and dry THF (1.5 mL). The reaction tube was purged with N_2, sealed with a Teflon cap and stirred at 110 ^oC for 48 h. The reaction was cooled to room temperature. The reaction mixture was filtered through Celite and the filtrate was concentrated in *vacuo*. The residue was purified by preparative TLC to afford desired product.

phenyl(1^1,1^8,3^2,5^2-tetrabutoxy-1^9H-1(2,7)-fluorena-3,5(1,3)-dibenzenacyclohexaphane-1^4-yl)methanone (**3a**)

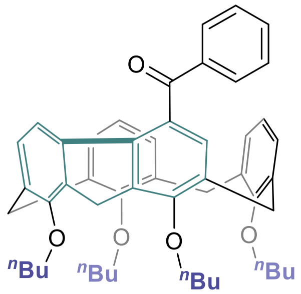

Chemical Formula: $C_{51}H_{58}O_5$; Molecular Weight: 750.4284.

General procedure A: 23.3 mg, 62% yield, 90% *ee*; **General procedure B**: 17.7 mg, 47% yield, 94% *ee*. Yellow solid. **m.p.** = 143-146 °C. **^{1}H NMR** (400 MHz, $CDCl_3$, 298 K) δ 7.47-7.40 (m, 1H), 7.26-7.22 (m, 1H), 7.20 (d, *J* = 7.4 Hz, 1H), 7.18 (d, *J* = 7.3 Hz, 1H), 7.09 (dd, *J* = 7.8, 1.7 Hz, 1H), 6.95-6.89 (m, 2H), 6.88-6.82 (m, 2H), 6.79 (dd, *J* = 7.4, 1.6 Hz, 1H), 6.72 (s, 1H), 6.63 (t, *J* = 7.6 Hz, 1H), 6.45 (d, *J* = 8.4 Hz, 1H), 6.33 (d, *J* = 8.4 Hz, 1H), 4.41 (d, *J* = 12.9 Hz, 1H), 4.35 (d, *J* = 12.5 Hz, 1H), 4.33-4.27 (m, 1H), 4.23 (d, *J* = 12.9 Hz, 1H), 4.14 (dt, *J* = 9.6, 6.5 Hz, 1H), 4.06 (dt, *J* = 9.7, 6.4 Hz, 1H), 4.02-3.92 (m, 3H), 3.91 (d, *J* = 19.3 Hz, 1H), 3.88 (d, *J* = 19.3 Hz, 1H), 3.56-3.46 (m, 2H), 3.26 (d, *J* = 13.0 Hz, 1H), 3.24 (d, *J* = 12.6 Hz, 1H) , 3.01 (d, *J* = 13.0 Hz, 1H), 2.15-1.77 (m, 8H), 1.73-1.57 (m, 4H), 1.47-1.31 (m, 4H), 1.10-0.98 (m, 12H). **^{13}C NMR** (100 MHz, $CDCl_3$, 298 K) δ 197.0, 159.9, 156.9, 156.3, 156.0, 145.2, 143.5, 138.7, 138.4, 138.3, 135.2, 134.1, 133.0, 132.1, 130.5, 130.4, 129.7, 129.5, 129.2, 128.8, 128.2, 127.9, 127.2, 123.3, 122.9, 117.3, 75.3, 75.2, 73.9, 73.6, 34.5, 33.4, 33.1, 32.74, 32.69, 32.3, 32.2, 26.2, 19.7, 19.6, 19.3, 14.4, 14.2, 14.1. **IR** (KBr) *v* 3060, 2958, 2931, 2872, 1653, 1555, 1453, 1274, 1211, 1090, 758 cm^{-1}. **HRMS** (ESI) m/z calcd. for $C_{51}H_{59}O_5^+$ $[M+H]^+$ 751.43570; Found: 751.43719.

HPLC: ID column, Hexane : *i*-PrOH= 80 : 20 , 24 °C, 0.5 mL/min flow rate, detection at 365 nm, t_1 = 19.3 min (minor), t_2 = 24.5 min (major). **$[\alpha]_D^{27}$** = +475 (*c* = 2.0, $CHCl_3$) for 94% *ee* for *P*-**3a**. **$[\alpha]_D^{27}$** = -465 (*c* = 2.0, $CHCl_3$) for 91% *ee* *M*-**3a**.

phenyl(1[1],1[8],3[2],5[2]-tetrapropoxy-1[9]H-1(2,7)-fluorena-3,5(1,3)-dibenzenacyclohexaphane-1[4]-yl)methanone (**3b**)

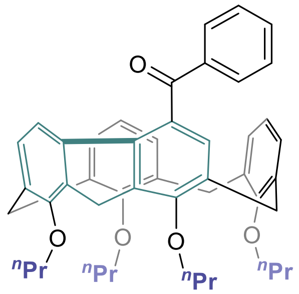

Chemical Formula: $C_{47}H_{50}O_5$; Molecular Weight: 694.3658.

General procedure A: 20.1 mg, 58% yield, 88% *ee*; **General procedure B:** 15.3 mg, 44% yield, 93% *ee*. Yellow solid. **m.p.** = 158-160 °C. **^{1}H NMR** (400 MHz, $CDCl_3$, 298 K) δ 7.46-7.41 (m, 1H), 7.26-7.22 (m, 1H), 7.20 (d, *J* = 7.4 Hz, 1H), 7.18 (d, *J* = 7.5 Hz, 1H), 7.09 (dd, *J* = 7.9, 1.3 Hz, 1H), 6.95-6.89 (m, 2H), 6.88-6.83 (m, 2H), 6.79 (dd, *J* = 7.5, 1.5 Hz, 1H), 6.72 (s, 1H), 6.63 (t, *J* = 7.6 Hz, 1H), 6.45 (d, *J* = 8.4 Hz, 1H), 6.33 (d, *J* = 8.4 Hz, 1H), 4.42 (d, *J* = 13.0 Hz, 1H), 4.36 (d, *J* = 12.6 Hz, 1H),.4.27 (dt, *J* = 9.5, 6.5 Hz, 1H). 4.25 (d, *J* = 13.0 Hz, 1H)**,** 4.11 (dt, *J* = 9.6, 6.6 Hz, 1H), 4.02 (dt, *J* = 9.6, 6.5 Hz, 1H), 3.99-3.93 (m, 3H), 3.91 (d, *J* = 19.3 Hz, 1H), 3.88 (d, *J* = 19.3 Hz, 1H), 3.54-3.42 (m, 2H), 3.26 (d, *J* = 13.1 Hz, 1H), 3.24 (d, *J* = 12.7 Hz, 1H), 3.01 (d, *J* = 13.0 Hz, 1H), 2.17-1.79 (m, 8H), 1.15 (t, *J* = 7.4 Hz, 6H), 1.00-0.92 (m, 6H). **^{13}C NMR** (100 MHz, $CDCl_3$, 298 K) δ 197.0, 159.8, 156.9, 156.3, 156.0, 145.2, 143.5, 138.7, 138.44, 138.40, 138.3, 135.2, 134.1, 133.0, 132.1, 130.5, 130.4, 129.7, 129.5, 129.2, 128.8, 128.2, 127.9, 127.2, 123.3, 122.9, 117.3, 75.9, 75.5, 34.5, 33.5, 33.2, 26.1, 23.84, 23.81, 23.28, 23.25, 11.0, 10.9, 10.2, 10.1. **IR** (KBr) *v* 3359, 2963, 2932, 2875, 1652, 1555, 1453, 1273, 1212, 1090, 755 cm^{-1}. **HRMS** (ESI) m/z calcd. for $C_{47}H_{51}O_5^+$ $[M+H]^+$ 695.37310; Found: 695.37347.

HPLC: ID column, Hexane : *i*-PrOH= 80 : 20 , 24 °C, 0.5 mL/min flow rate, detection at 365 nm, t_1 = 21.6 min (minor), t_2 = 26.3 min (major). **$[\alpha]_D$**^{27} = +536 (*c* = 2.0, $CHCl_3$) for 93% *ee*.

phenyl(1[1],1[8],3[2],5[2]-tetrakis(pentyloxy)-1[9]H-1(2,7)-fluorena-3,5(1,3)-dibenzenacyclohexaphane-1[4]-yl)methanone (**3c**)

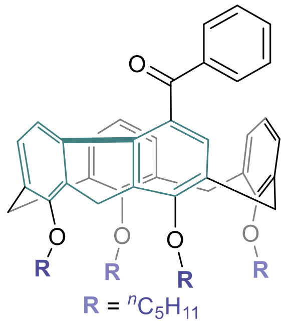

Chemical Formula: $C_{55}H_{66}O_5$; Molecular Weight: 806.4910.

General procedure A: 23.4 mg, 58% yield, 90% *ee*; **General procedure B:** 18.6 mg, 46% yield, 93% *ee*. Yellow solid. **m.p.** = 82-85 °C. **^{1}H NMR** (400 MHz, $CDCl_3$, 298 K) δ 7.47-7.41 (m, 1H), 7.25-7.22 (m, 1H), 7.20 (d, J = 7.4 Hz, 1H), 7.18 (d, J = 7.5 Hz, 1H), 7.09 (dd, J = 7.7, 1.4 Hz, 1H), 6.94-6.89 (m, 2H), 6.88-6.83 (m, 2H), 6.79 (dd, J = 7.4, 1.5 Hz, 1H) , 6.72 (s, 1H), 6.63 (t, J = 7.6 Hz, 1H), 6.45 (d, J = 8.4 Hz, 1H), 6.33 (d, J = 8.4 Hz, 1H), 4.41 (d, J = 13.0 Hz, 1H), 4.35 (d, J = 12.7 Hz, 1H), 4.32-4.26 (m, 1H), 4.23 (d, J = 13.0 Hz, 1H), 4.13 (dt, J = 9.6, 6.6 Hz, 1H), 4.05 (dt, J = 9.6, 6.5 Hz, 1H), 4.01-3.93 (m, 3H), 3.92 (d, J = 19.6 Hz, 1H), 3.91 (d, J = 19.6 Hz, 1H), 3.88 (d, J = 19.6 Hz, 1H), 3.56-3.46 (m, 2H), 3.26 (d, J = 12.6 Hz, 1H), 3.24 (d, J = 12.7 Hz, 1H), 3.00 (d, J = 13.0 Hz, 1H), 2.17-1.79 (m, 8H), 1.66-1.51 (m, 4H), 1.54-1.35 (m, 8H), 1.40-1.22 (m, 4H), 1.08-0.92 (m, 12H). **^{13}C NMR** (100 MHz, $CDCl_3$, 298 K) δ 197.0, 159.9, 157.0, 156.3, 155.9, 145.2, 143.5, 138.7, 138.43, 138.41, 138.3, 135.2, 134.1, 133.0, 132.1, 130.5, 130.4, 129.7, 129.5, 129.1, 128.8, 128.2, 127.9, 127.1, 123.3, 122.9, 117.3, 75.44, 75.37, 74.3, 73.9, 34.5, 33.5, 33.2, 30.31, 30.29, 29.87, 29.85, 28.7, 28.6, 28.2, 26.2, 23.1, 23.0, 22.8, 22.7, 14.4, 14.3. **IR** (KBr) ν 3060, 2955, 2922, 2870, 1653, 1555, 1453, 1273, 1212, 1090, 1078, 757 cm^{-1}. **HRMS** (ESI) m/z calcd. for $C_{55}H_{67}O_5^+$ $[M+H]^+$ 807.49830; Found: 807.49939.

HPLC: ID column, Hexane : *i*-PrOH= 80 : 20 , 24 °C, 0.5 mL/min flow rate, detection at 365 nm, t_1 = 18.5 min (minor), t_2 = 23.1 min (major). **$[\alpha]_D^{27}$** = +446 (c = 2.0, $CHCl_3$) for 93% *ee*.

phenyl(1^1,1^8,3^2,5^2-tetrakis(octyloxy)-1^{9}H-1(2,7)-fluorena-3,5(1,3)-dibenzenacyclohexaphane-1^4-yl)methanone (**3d**)

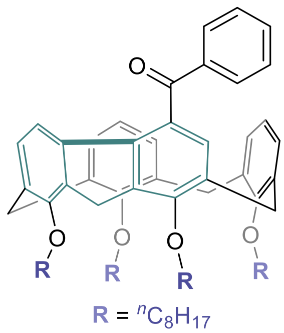

Chemical Formula: $C_{67}H_{90}O_5$; Molecular Weight: 974.6788.

General procedure A: 31.7 mg, 65% yield, 93% *ee*; **General procedure B:** 22.4 mg, 46% yield, 93% *ee*. Yellow solid. **m.p.** = 63-64 °C. **^{1}H NMR** (400 MHz, $CDCl_3$, 298 K) δ 7.48-7.38 (m, 1H), 7.25-7.22 (m, 1H), 7.22-7.15 (m, 2H), 7.11-7.06 (m, 1H), 6.96-6.89 (m, 2H), 6.88-6.83 (m, 2H), 6.82-6.76(m, 1H), 6.72 (s, 1H), 6.63 (t, J = 7.5 Hz, 1H), 6.45 (d, J = 8.4 Hz, 1H), 6.33 (d, J = 8.4 Hz, 1H), 4.40 (d, J = 13.0 Hz, 1H), 4.34 (d, J = 12.4 Hz, 1H), 4.32-4.26 (m, 1H), 4.22 (d, J = 13.0 Hz, 1H), 4.18-4.09 (m, 1H), 4.09-4.01 (m, 1H), 4.00-4.92 (m, 3H), 3.91 (d, J = 19.9 Hz, 1H), 3.87 (d, J = 19.9 Hz, 1H), 3.57-3.44 (m, 2H), 3.25 (d, J = 13.0 Hz, 1H), 3.23 (d, J = 12.6 Hz, 1H), 3.00 (d, J = 12.7 Hz, 1H), 2.18-1.77 (m, 8H), 1.28-1.27 (m, 40H), 0.99-0.83 (m, 12H). **^{13}C NMR** (100 MHz, $CDCl_3$, 298 K) δ 197.0, 159.9, 157.0, 156.3, 156.0, 145.2, 143.5, 138.7, 138.5, 138.43, 138.39, 138.3, 135.2, 134.2, 133.0, 132.1, 130.5, 130.4, 129.7, 129.5, 129.1, 128.8, 128.2, 127.9, 127.1, 123.3, 122.9, 117.3, 75.5, 75.4, 74.3, 73.9, 34.6, 33.5, 33.2, 32.2, 32.14, 32.11, 32.07, 30.7, 30.6, 30.3, 30.24, 30.15, 30.1, 29.80, 29.76, 29.7, 29.62, 29.55, 26.6, 26.5, 26.2, 22.89, 22.87, 22.85, 14.3. **IR** (KBr) ν 3361, 2954, 2923, 2854, 1654, 1454, 1379, 1273, 1212, 1090, 757 cm^{-1}. **HRMS** (ESI) m/z calcd. for $C_{67}H_{91}O_5^+$ $[M+H]^+$ 975.68610; Found: 975.68768.

HPLC: ID column, Hexane : *i*-PrOH= 80 : 20 , 24 °C, 0.5 mL/min flow rate, detection at 365 nm, t_1 = 14.5 min (minor), t_2 = 16.9 min (major). **$[\alpha]_D^{27}$** = +427 (c = 2.0, $CHCl_3$) for 93% *ee*.

phenyl(1^1,1^8,3^2,5^2-tetramethoxy-1^9H-1(2,7)-fluorena-3,5(1,3)-dibenzenacyclohexaphane-1^4-yl)methanone (**3e**)

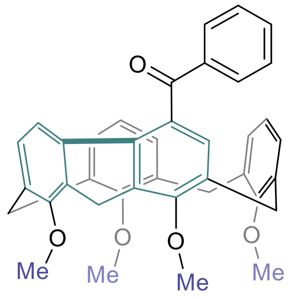

Chemical Formula: $C_{39}H_{34}O_5$; Molecular Weight: 582.2406.

General procedure A: 13.4 mg, 46% yield, 84% *ee*; **General procedure B:** 11.6 mg, 40% yield, 93% *ee*. Yellow solid. **m.p.** = 60-63 °C. **^{1}H NMR** (400 MHz, $CDCl_3$, 298 K) δ 8.29-8.15 (m, 1H), 7.66-6.62 (m, 12H), 6.49-6.27 (m, 1H), 4.43-4.24 (m, 1H), 4.15-3.44 (m, 15H), 3.34-3.15 (m, 1H), 3.12-2.85(m, 3H). **^{13}C NMR** (100 MH, $CDCl_3$, 298 K), recorded as conformational isomers δ 196.7, 161.6, 160.7, 160.0, 157.5, 157.3, 157.1, 157.0, 147.5, 145.7, 145.5, 143.8, 139.3, 138.4, 138.2, 138.1, 138.0, 137.8, 137.7, 136.9, 135.3, 135.1, 134.9, 134.4, 134.2, 134.0, 133.1, 132.6, 132.4, 132.1, 131.3, 131.0, 130.9, 130.7, 130.5, 129.8, 129.6, 129.4, 129.1, 129.0, 128.8, 128.5, 128.2, 128.1, 128.0, 127.2, 126.4, 123.7, 123.4, 122.4, 122.3, 117.8, 117.6, 61.5, 61.5, 61.0, 60.4, 60.0, 59.7, 37.4, 36.6, 34.7, 33.1, 32.9, 26.9, 26.8. **IR** (KBr) *v* 3059, 2932, 2821, 1647, 1579, 1461, 1268, 1219, 1089, 1018, 766, 757 cm^{-1}. **HRMS** (ESI) m/z calcd. for $C_{39}H_{35}O_5^+$ $[M+H]^+$ 583.24790; Found: 583.24884.

HPLC: ID column, Hexane : *i*-PrOH= 80 : 20 , 24 °C, 0.5 mL/min flow rate, detection at 365 nm, t_1 = 38.7 min (minor), t_2 = 41.7 min (major). $[\alpha]_D^{27}$ = +718 (*c* = 2.0, $CHCl_3$) for 93% *ee.*

(1^1,1^8,3^2,5^2-tetrabutoxy-1^9H-1(2,7)-fluorena-3,5(1,3)-dibenzenacyclohexaphane-1^4-yl)(m-tolyl)methanone (**3f**)

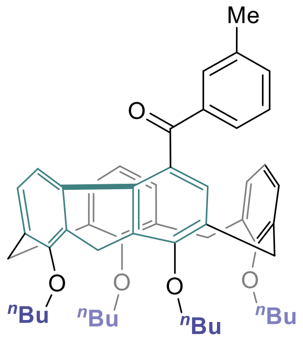

Chemical Formula: $C_{52}H_{60}O_5$; Molecular Weight: 765.0470.

General procedure A: 21.8 mg, 57% yield, 90% *ee*; **General procedure B:**19.1 mg, 50% yield, 92% *ee.* Yellow solid. **m.p.** = 34-36 °C. **^{1}H NMR** (400 MHz, $CDCl_3$, 298 K) δ 7.34-7.31 (m, 1H), 7.26-7.20 (m, 2H), 7.09-7.05 (m, 1H), 6.99 (t, J = 7.6 Hz, 1H), 6.88-6.77 (m, 3H), 6.74 (s, 1H), 6.63 (t, J = 7.5 Hz, 1H), 6.47 (d, J = 8.3 Hz, 1H), 6.35 (d, J = 8.4 Hz, 1H), 6.24 (d, J = 7.5 Hz, 1H), 4.41 (d, J = 13.0 Hz, 1H), 4.35 (d, J = 12.6 Hz, 1H), 4.32-4.27 (m, 1H), 4.22 (d, J = 13.0 Hz, 1H), 4.14 (dt, J = 9.4, 6.4 Hz, 1H), 4.05 (dt, J = 10.0, 6.5 Hz, 1H), 4.02-3.93 (m, 3H), 3.91 (d, J = 19.3 Hz, 1H), 3.87 (d, J = 19.3 Hz, 1H), 3.56-3.45 (m, 2H), 3.24 (d, J = 13.0 Hz, 1H). 3.23 (d, J = 12.6 Hz, 1H), 3.00 (d, J = 13.0 Hz, 1H), 2.33 (s, 3H), 2.14-1.76 (m, 8H), 1.72-1.58 (m, 4H), 1.46-1.32 (m, 4H), 1.10-0.96 (m, 12H). **^{13}C NMR** (100 MHz, $CDCl_3$, 298 K) δ 197.1, 159.9, 156.9, 156.3, 156.0, 145.4, 143.6, 138.7, 138.6, 138.5, 138.33, 138.27, 137.9, 135.2, 134.1, 133.0, 132.9, 130.4, 130.0, 129.7, 129.6, 129.3, 128.8, 128.6, 128.0, 127.9, 127.1, 123.3, 122.9, 117.4, 75.3, 75.2, 73.9, 73.6, 34.5, 33.4, 33.1, 32.74, 32.69, 32.3, 32.2, 26.2, 21.5, 19.7, 19.6, 19.3, 14.4, 14.2, 14.1. **IR** (KBr) v 3361, 3060, 2958, 2930, 2871, 1651, 1584, 1454, 1274, 1206, 1090, 763 cm^{-1}. **HRMS** (ESI) m/z calcd. for $C_{52}H_{61}O_5^+$ $[M+H]^+$ 765.45135; Found: 765.45148.

HPLC: ID column, Hexane:*i*-PrOH= 80 : 20 , 24 °C, 0.5 mL/min flow rate, detection at 365 nm, t_1 = 18.9 min (minor), t_2 = 26.2 min (major). **$[\alpha]_D^{27}$** = +540 (c = 2.0, $CHCl_3$) for 92% *ee.*

(3-methoxyphenyl)(1^1,1^8,3^2,5^2-tetrabutoxy-1^{9}H-1(2,7)-fluorena-3,5(1,3)-dibenzenacyclohexaphane-1^4-yl)methanone (3g)

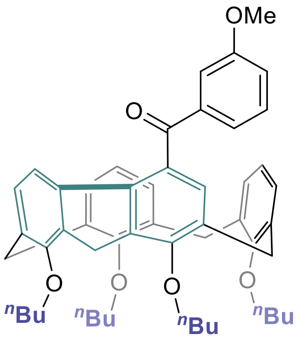

Chemical Formula: $C_{52}H_{60}O_6$; Molecular Weight: 781.0460.

General procedure A: 20.3 mg, 52% yield, 87% *ee*; **General procedure B:** 18 mg, 46% yield, 89% *ee*. Yellow solid. **m.p.** = 42-45 °C. **^{1}H NMR** (400 MHz, $CDCl_3$, 298 K) δ 7.32-7.29 (m, 1H), 7.21 (dd, J = 7.3, 2.0 Hz, 1H), 7.07 (dd, J = 7.8, 1.3 Hz, 1H), 7.02-6.92 (m, 2H), 6.90-6.76 (m, 4H), 6.64 (t, J = 7.6 Hz, 1H), 6.43 (d, J = 8.3 Hz, 1H), 6.34 (d, J = 8.4 Hz, 1H), 4.41 (d, J = 12.9 Hz, 1H), 4.35 (d, J = 12.6 Hz, 1H), 4.31 (dt, J = 9.7, 6.4 Hz, 1H), 4.22 (d, J = 13.0 Hz, 1H), 4.15 (dt, J = 9.6, 6.5 Hz, 1H), 4.06 (dt, J = 9.7, 6.4 Hz, 1H), 4.02-3.93 (m, 3H), 3.92 (d, J = 19.2 Hz, 1H), 3.88 (d, J = 19.2 Hz, 1H), 3.82 (s, 3H), 3.55-4.55 (m, 2H), 3.27 (d, J = 12.3 Hz, 1H). 3.24 (d, J = 11.9 Hz, 1H), 3.00 (d, J = 13.0 Hz, 1H), 2.17-1.77 (m, 8H), 1.71-1.59 (m, 4H), 1.46 -1.32 (m, 4H), 1.08-0.98 (m, 12H). **^{13}C NMR** (100 MHz, $CDCl_3$, 298 K) δ 196.5, 159.9, 159.6, 157.0, 156.2, 156.0, 145.2, 143.5, 139.8, 138.6, 138.4, 138.3, 138.2, 135.0, 134.1, 133.0, 130.4, 129.7, 129.5, 129.1, 129.0, 128.9, 127.90, 127.85, 127.2, 124.5, 123.3, 122.9, 119.4, 117.6, 112.8, 75.22, 75.18, 73.9, 73.6, 55.5, 34.5, 33.4, 33.1, 32.73, 32.68, 32.2, 26.2, 19.7, 19.6, 19.3, 14.4, 14.4, 14.2, 14.1. **IR** (KBr) ν 3362, 3057, 2957, 2931, 2872, 1651, 1556, 1463, 1454, 1252, 1206, 1090, 765 cm^{-1}. **HRMS** (ESI) m/z calcd. for $C_{52}H_{61}O_6^+$ $[M+H]^+$ 781.44627; Found: 781.44473.

HPLC: ID column, Hexane : *i*-PrOH= 80 : 20 , 24 °C, 0.5 mL/min flow rate, detection at 365 nm, t_1 = 22.6 min (minor), t_2 = 28.3 min (major). **$[\alpha]_D^{27}$** = +530 (c = 1.0, $CHCl_3$) for 89% *ee*.

(3-fluorophenyl)(1^1,1^8,3^2,5^2-tetrabutoxy-1^{9}H-1(2,7)-fluorena-3,5(1,3)-dibenzenacyclohexaphane-1^4-yl)methanone (**3h**)

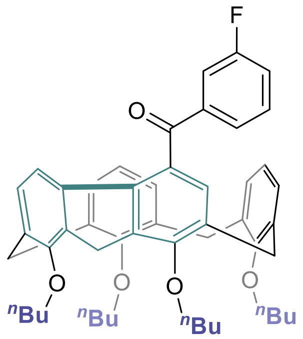

Chemical Formula: $C_{51}H_{57}FO_5$; Molecular Weight: 769.0104.

General procedure A: 10 mg, 26% yield, 89% *ee*; **General procedure C:** 13.4 mg, 34% yield, 89% *ee*. Yellow solid. **m.p.** = 30-33 °C. **^{1}H NMR** (400 MHz, $CDCl_3$, 298 K) δ 7.21 (dd, J = 6.4, 2.6 Hz, 1H), 7.17-7.05 (m, 3H), 7.09-7.05 (m, 1H), 7.01-6.96 (m, 2H), 6.89-6.78 (m, 4H), 6.75 (s, 1H), 6.65 (t, J = 7.6 Hz, 1H), 6.51-6.43 (m, 1H), 6.42 (d, J = 8.4 Hz, 1H), 6.35 (d, J = 8.3 Hz, 1H), 4.40 (d, J = 13.0 Hz, 1H), 4.37-4.28 (m, 2H), 4.20 (d, J = 13.0 Hz, 1H), 4.14 (dt, J = 9.5, 6.5 Hz, 1H), 4.06 (dt, J = 9.6, 6.3 Hz, 1H), 4.02-3.92 (m, 3H), 3.91 (d, J = 19.1 Hz, 1H), 3.87 (d, J = 19.1 Hz, 1H), 3.56-3.44 (m, 2H), 3.26 (d, J = 13.0 Hz, 1H), 3.24 (d, J = 12.6 Hz, 1H), 2.99 (d, J = 13.0 Hz, 1H), 2.15-1.76 (m, 8H), 1.72-1.59 (m, 4H), 1.46-1.31 (m, 4H), 1.14-0.96 (m, 12H). **^{13}C NMR** (100 MHz, $CDCl_3$, 298 K) δ 195.2, 162.4 (d, J = 248.7 Hz), 160.2, 157.0, 156.2, 156.0, 145.4, 143.3, 140.7 (d, J = 6.1 Hz), 138.7, 138.5, 138.3, 138.2, 135.1, 134.1, 133.0, 130.5, 129.9, 129.8 (d, J = 7.6 Hz), 129.7, 129.0, 128.3, 127.9, 127.8, 127.3, 126.6 (d, J = 2.7 Hz), 123.5, 122.9, 119.1 (d, J = 21.3 Hz), 117.4, 116.5 (d, J = 21.8 Hz), 75.3, 75.2, 74.0, 73.6, 34.6, 33.4, 33.1, 32.73, 32.69, 32.3, 32.2, 26.1, 19.7, 19.6, 19.3, 14.38, 14.36, 14.2, 14.1. **IR** (KBr) ν 3361, 3065, 2958, 2929, 2872, 1728, 1656, 1585, 1454, 1272, 1071, 766 cm^{-1}. **HRMS** (ESI) m/z calcd. for $C_{51}H_{58}O_5F^+$ $[M+H]^+$ 769.42628; Found: 769.42084.

HPLC: ID column, Hexane : *i*-PrOH= 80 : 20 , 24 °C, 0.5 mL/min flow rate, detection at 365 nm, t_1 = 16.3 min (minor), t_2 = 22.9 min (major). **$[\alpha]_D^{27}$** = +312 (c = 1.0, $CHCl_3$) for 89% *ee*.

(1^1,1^8,3^2,5^2-tetrabutoxy-1^9H-1(2,7)-fluorena-3,5(1,3)-dibenzenacyclohexaphane-1^4-yl)(p-tolyl)methanone (**3i**)

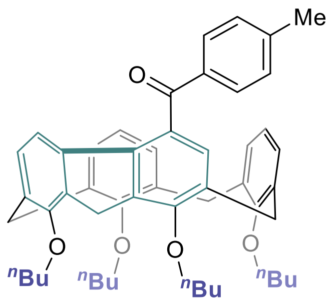

Chemical Formula: $C_{52}H_{60}O_5$; Molecular Weight: 765.0470.

General procedure A: 21.4 mg, 56% yield, 87% *ee*; **General procedure B:** 16.8 mg, 44% yield, 92% *ee.* Yellow solid. **m.p.** = 38-40 °C. **^{1}H NMR** (400 MHz, $CDCl_3$, 298 K) δ 7.24 (dd, J = 6.4, 2.9 Hz, 1H), 7.09 (dd, J = 7.7, 1.2 Hz, 1H), 7.03-6.97 (m, 2H), 6.91-6.84 (m, 4H), 6.79 (dd, J = 7.4, 1.5 Hz, 1H), 6.74 (s, 1H), 6.64 (t, J = 7.6 Hz, 1H), 6.42 (d, J = 8.3 Hz, 1H), 6.32 (d, J = 8.4 Hz, 1H), 4.41 (d, J = 12.9 Hz, 1H), 4.35 (d, J = 12.8 Hz, 1H), 4.32-4.26 (m, 1H), 4.22 (d, J = 12.9 Hz, 1H), 4.14 (dt, J = 9.6, 6.5 Hz, 1H), 4.05 (dt, J = 9.7, 6.3 Hz, 1H), 4.01-3.93 (m, 3H), 3.91 (d, J = 19.2 Hz, 1H), 3.87 (d, J = 19.2 Hz, 1H), 3.57-3.44 (m, 2H), 3.26 (d, J = 12.8 Hz, 1H), 3.23 (d, J = 12.4 Hz, 1H), 3.00 (d, J = 13.0 Hz, 1H), 2.39 (s, 3H), 2.15-1.77 (m, 8H), 1.72-1.60 (m, 4H), 1.47-1.31 (m, 4H), 1.11-0.97 (m, 12H). **^{13}C NMR** (100 MHz, $CDCl_3$, 298 K) δ 196.5, 159.7, 157.0, 156.3, 156.0, 145.1, 143.6, 142.7, 138.7, 138.44, 138.39, 138.3, 135.8, 135.1, 134.1, 132.8, 130.7, 130.4, 129.6, 129.5, 129.3, 128.9, 128.8, 127.92, 127.87, 127.2, 123.2, 122.9, 117.5, 75.2, 75.2, 73.9, 73.6, 34.5, 33.4, 33.2, 32.8, 32.7, 32.3, 32.2, 26.1, 21.8, 19.72, 19.65, 19.3, 14.4, 14.2, 14.1. **IR** (KBr) ν 3360, 2957, 2928, 2871, 1649, 1453, 1273, 1208, 1090, 1076, 766 cm^{-1}. **HRMS** (ESI) m/z calcd. for $C_{52}H_{61}O_5^+$ $[M+H]^+$ 765.45135; Found: 765.45142.

HPLC: ID column, Hexane : *i*-PrOH= 80 : 20 , 24 °C, 0.5 mL/min flow rate, detection at 365 nm, t_1 = 22.0 min (minor), t_2 = 27.1 min (major). **$[\alpha]_D^{27}$** = +495 (c = 2.0, $CHCl_3$) for 92% *ee.*

(4-fluorophenyl)(1^1,1^8,3^2,5^2-tetrabutoxy-1^9H-1(2,7)-fluorena-3,5(1,3)-dibenzenacyclohexaphane-1^4-yl)methanone (**3j**)

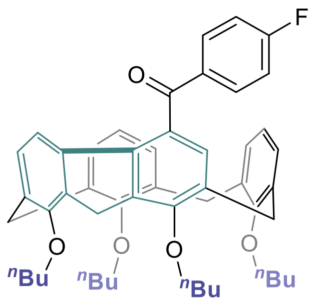

Chemical Formula: $C_{51}H_{57}FO_5$; Molecular Weight: 769.0104

General procedure A: 1.6 mg, 4% yield, 91% *ee*; **General procedure B:** 1.6 mg, 4% yield, 89% *ee.* Yellow solid. **m.p.** = 120-123 °C. **^{1}H NMR** (400 MHz, $CDCl_3$, 298 K) δ 7.26-7.21 (m, 1H), 7.11-7.05 (m, 1H), 6.92-6.77 (m, 7H), 6.68-6.60 (m, 2H), 6.41-6.30 (m, 2H), 4.41 (d, J = 13.0 Hz, 1H), 4.35 (d, J = 12.5 Hz, 1H), 4.31 (dt, J = 9.6, 6.4 Hz, 1H), 4.22 (d, J = 12.9 Hz, 1H), 4.14 (dt, J = 9.5, 6.5 Hz, 1H), 4.05 (dt, J = 9.6, 6.4 Hz, 1H), 4.05-3.90 (m, 3H), 3.91 (d, J = 19.3 Hz, 1H), 3.87 (d, J = 19.3 Hz, 1H), 3.57-3.44 (m, 2H), 3.26 (d, J = 13.1 Hz, 1H), 3.24 (d, J = 12.6 Hz, 1H), 3.01 (d, J = 13.0 Hz, 1H), 2.16-1.78 (m, 8H), 1.72-1.59 (m, 4H), 1.48-1.29 (m, 4H), 1.10-0.96 (m, 12H). **^{13}C NMR** (100 MHz, $CDCl_3$, 298 K) δ 195.7, 165.2 (d, J = 253.2 Hz), 159.9, 156.9, 156.3, 155.9, 144.8, 143.2, 138.7, 138.54, 138.45, 138.3, 135.3, 134.6 (d, J = 2.7 Hz), 134.1, 133.1, 133.0, 132.6, 130.4, 129.8, 129.5, 128.9, 128.7, 127.89, 127.88, 127.2, 123.3, 122.9, 117.1, 115.3 (d, J = 21.6 Hz), 75.3, 75.2, 73.9, 73.6, 34.5, 33.4, 33.1, 32.72, 32.66, 32.3, 32.2, 26.1, 19.7, 19.6, 19.3, 14.38, 14.36, 14.2, 14.1. **IR** (KBr) ν 3360, 2958, 2930, 2872, 1657, 1596, 1454, 1274, 1210, 1090, 771 cm^{-1}. **HRMS** (ESI) m/z calcd. for $C_{51}H_{58}O_5F^+$ $[M+H]^+$ 769.42628; Found: 769.42786.

HPLC: ID column, Hexane : *i*-PrOH= 80 : 20 , 24 °C, 0.5 mL/min flow rate, detection at 365 nm, t_1 = 15.8 min (minor), t_2 = 19.0 min (major). **$[\alpha]_D^{27}$** = +448 (c = 0.25, $CHCl_3$) for 91% *ee.*

(1^1,1^8,3^2,5^2-tetrabutoxy-1^9H-1(2,7)-fluorena-3,5(1,3)-dibenzenacyclohexaphane-1^4-yl)(o-tolyl)methanone (**3k**)

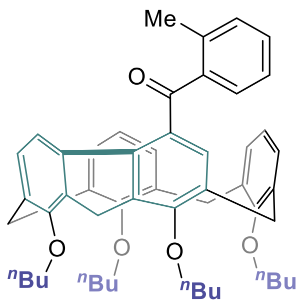

Chemical Formula: $C_{52}H_{60}O_5$; Molecular Weight: 765.0470

General procedure B: 21 mg, 55% yield, 62% *ee.* Yellow solid. **m.p.** = 165-168 °C. **^{1}H NMR** (400 MHz, $CDCl_3$, 298 K) δ 7.30-7.24 (m, 1H), 7.20 (dd, J = 7.7, 1.4 Hz, 1H), 7.09-7.00 (m, 2H), 6.99-6.92 (m, 2H), 6.85 (dd, J = 7.4, 1.4 Hz, 1H), 6.83-6.78 (m, 1H), 6.76 (t, J = 7.5 Hz, 1H), 6.70-6.64 (m, 2H), 6.52-6.45 (m, 2H), 4.39 (d, J = 12.4 Hz, 1H), 4.36-4.28 (m, 2H), 4.19 (d, J = 12.9 Hz, 1H), 4.17-4.11 (m, 1H), 4.07-3.90 (m, 4H), 3.91 (d, J = 19.2 Hz, 1H), 3.88 (d, J = 19.2 Hz, 1H), 3.59-3.41 (m, 2H), 3.30 (d, J = 12.5 Hz, 1H), 3.13 (d, J = 13.2 Hz, 1H), 2.98 (d, J = 13.0 Hz, 1H), 2.17-1.76 (m, 8H), 1.73-1.57 (m, 4H), 1.47-1.30 (m, 4H), 1.10-0.95 (m, 12H), 0.91 (s, 3H). **^{13}C NMR** (100 MHz, $CDCl_3$, 298 K) δ 199.1, 160.8, 156.5, 155.9, 145.6, 143.7, 140.1, 139.04, 138.95, 138.3, 138.2, 137.5, 135.1, 134.4, 133.7, 130.7, 130.6, 130.4, 130.1, 130.0, 129.2, 128.8, 127.9, 127.8, 127.2, 125.3, 123.2, 122.8, 116.8, 75.3, 75.1, 74.0, 73.5, 34.6, 33.5, 32.8, 32.7, 32.3, 32.2, 26.3, 19.72, 19.70, 19.6, 19.30, 19.27, 14.4, 14.3, 14.2, 14.1. **IR** (KBr) v 3428, 3061, 2957, 2930, 2871, 1651, 1550, 1454, 1272, 1213, 1090, 1075, 758 cm^{-1}. **HRMS** (ESI) m/z calcd. for $C_{52}H_{61}O_5^+$ $[M+H]^+$ 765.45135; Found: 765.44962.

HPLC: ID column, Hexane : *i*-PrOH= 80 : 20 , 24 °C, 0.5 mL/min flow rate, detection at 365 nm, t_1 = 21.9 min (minor), t_2 = 38.5 min (major). **$[\alpha]_D^{27}$** = +292 (c = 1.0, $CHCl_3$) for 62% *ee.*

7. Synthesis of 5

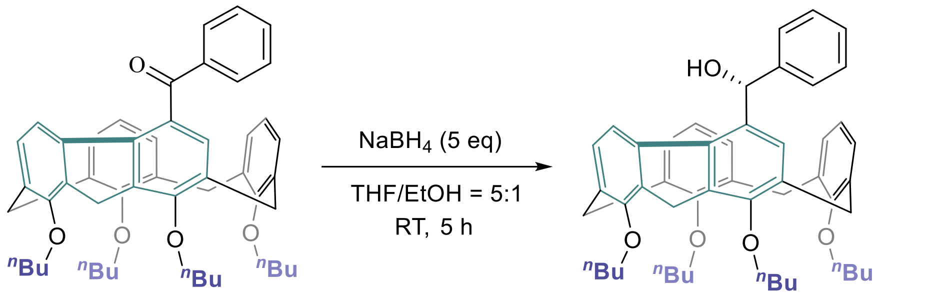

3a, 94% *ee* **5**, 75% yield, 94% *ee*, >20:1 d.r.

To a solution of **3a** (20 mg, 0.026mmol, 1 equiv, 94% *ee*) in THF/EtOH (6 mL, 5/1, v/v) was added $NaBH_4$ (5 mg, 0.13 mmol, 5 equiv) at room temperature. After stirred for 5 h, starting material was completely consumed. The reaction was quenched by H_2O, concentrated in *vacuo*. The residue was dissolved and extracted with DCM (3 × 20 mL). The combined organic layers were washed with brine and dried over Na_2SO_4. Solvents were removed in *vacuo* and the residue was purified by preparative TLC (PE : EA = 10 : 1) to give the pure product **5** (15 mg, 75% yield, 94% *ee*), colourless solid, **m.p.** = 49-51 ^{o}C. **^{1}H NMR** (400 MHz, $CDCl_3$, 298 K) δ 7.17-7.04 (m, 5H), 6.88-6.83 (m, 4H), 6.78 (t, *J* = 7.5 Hz, 1H), 6.74 (d, *J* = 8.3 Hz, 1H), 6.70 (t, *J* = 7.5 Hz, 1H), 6.63 (s, 1H), 6.36 (d, *J* = 8.3 Hz, 1H), 5.99 (s, 1H), 4.37 (d, *J* = 13.2 Hz, 1H), 4.36 (d, *J* = 13.2 Hz, 1H), 4.19 (d, *J* = 13.0 Hz, 1H), 4.15-4.11 (m, 2H), 4.01-3.93 (m, 5H), 3.94 (d, *J* = 19.4 Hz, 1H), 3.55-3.41 (m, 2H), 3.28 (d, *J* = 13.2 Hz, 1H), 3.27 (d, *J* = 13.2 Hz, 1H), 2.97 (d, *J* = 13.2 Hz, 1H), 2.17-1.78 (m, 8H), 1.72-1.60 (m, 4H), 1.43-1.32 (m, 4H), 1.09-0.99 (m, 12H). **^{13}C NMR** (100 MHz, $CDCl_3$, 298 K) δ 157.4, 156.4, 156.0, 143.4, 143.0, 141.7, 138.8, 138.42, 138.35, 138.2, 134.6, 134.4, 132.9, 129.7, 129.4, 128.94, 128.92, 128.8, 128.7, 128.2, 127.9, 127.8, 126.6, 123.1, 119.0, 75.3, 75.1, 73.9, 73.8, 73.09, 73.07, 34.0, 33.6, 33.4, 32.8, 32.7, 32.22, 32.15, 26.1, 19.7, 19.3, 14.4, 14.3, 14.2. **IR** (KBr) *v* 3552, 3060, 2957, 2930, 2871, 1589, 1560, 1463, 1452, 1379, 1200, 1091, 763 cm^{-1}. **HRMS** (ESI) m/z calcd. for $C_{51}H_{59}O_5^-$ [M-H]$^-$ 751.43680; Found: 751.43860. **HPLC**: IB column, Hexane : *i*-PrOH= 95 : 5 , 24 ^{o}C, 0.5 mL/min flow rate, detection at 330 nm, t_1 = 12.2 min (minor), t_2 = 23.1 min (major). **$[\alpha]_D^{27}$** = +180 (*c* = 2.0, $CHCl_3$) for 94% *ee*.

8. Synthesis of 6

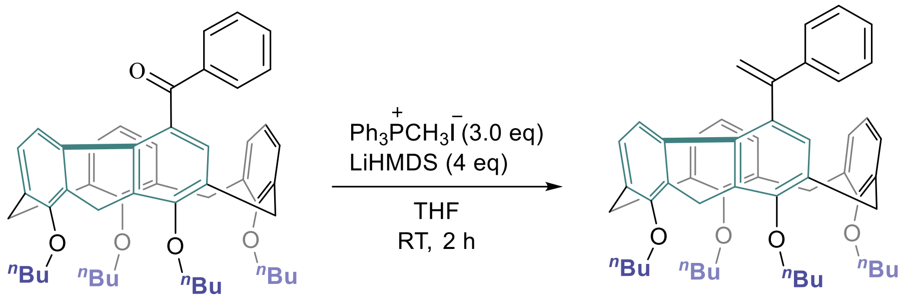

3a, 94% *ee* **6**, 71% yield, 94% *ee*

To a dry Schlenk tube was sequentially added CH_3PPh_3I (73 mg, 0.18 mmol, 3 equiv), dry THF (1.2mL), and LiHMDS (0.12 ml, 2 M in THF, 4 equiv) under N_2. After stirred for 0.5 h at room temperature, a solution of **3a** (44 mg, 0.06 mmol, 1 equiv, 94% *ee*) in THF (1.2 mL) was added. When the **3a** was consumed by TLC analysis, the reaction was quenched by H_2O and extracted with DCM (3 × 20 mL). The combined organic layers were washed with brine and dried over Na_2SO_4. Solvents were removed in *vacuo* and the residue was purified by preparative TLC (PE : EA = 40 : 1) to give the pure product **6** (31.4 mg, 71% yield, 94% *ee*), colourless solid, **m.p.** = 30-31 °C. **^{1}H NMR** (400 MHz, $CDCl_3$, 298 K) δ 7.19-7.13 (m, 3H), 6.99 (s, 1H), 7.01-6.96 (m, 2H), 6.82 (dd, *J* = 7.4, 1.4 Hz, 1H), 6.71-6.66 (m, 3H), 6.38 (br d, *J* = 7.0 Hz, 2H), 6.32 (d, *J* = 8.3 Hz, 1H), 6.30 (s, 1H), 5.52 (d, *J* = 1.6 Hz, 1H), 5.32 (s, 1H), 4.37 (d, *J* = 12.3 Hz, 1H), 4.33 (d, *J* = 12.3 Hz, 1H), 4.22 (d, *J* = 13.0 Hz, 1H), 4.23-4.14 (m, 2H), 4.04-3.93 (m, 4H), 3.92 (d, *J* = 19.3 Hz, 1H), 3.86 (d, *J* = 19.3 Hz, 1H), 3.54-3.45 (m, 2H), 3.26 (d, *J* = 12.7 Hz, 1H), 3.17 (d, *J* = 12.8 Hz, 1H), 2.99 (d, *J* = 12.9 Hz, 1H), 2.14-1.79 (m, 8H), 1.71-1.62 (m, 8H), 1.42-1.33 (m, 8H), 1.08-0.99 (m, 12H). **^{13}C NMR** (100 MHz, $CDCl_3$, 298 K) δ 157.1, 156.2, 156.1, 147.7, 144.7, 143.3, 140.9, 138.8, 138.5, 138.1, 134.8, 134.4, 132.9, 131.1, 130.1, 129.3, 129.0, 128.7, 128.2, 127.9, 127.8, 127.0, 123.3, 122.8, 116.6, 116.0, 75.2, 75.1, 73.8, 34.0, 33.4, 33.2, 32.8, 32.24, 32.19, 26.1, 19.7, 19.3, 14.4, 14.2. **IR** (KBr) ν 3189, 3058, 2957, 2926, 2871, 1659, 1463, 1454, 1266, 1090, 996, 763 cm^{-1}. **HRMS** (ESI) m/z calcd. for $C_{52}H_{61}O_4^+$ $[M+H]^+$ 749.45644; Found: 749.45056. **HPLC**: ID column, Hexane : *i*-PrOH= 98 : 2 , 24 °C, 0.5 mL/min flow rate,

detection at 330 nm, t_1 = 8.3 min (minor), t_2 = 8.8 min (major). **$[\alpha]_D$**^{27} = +183 (*c* = 2.0, $CHCl_3$) for 94% *ee*.

9. Synthesis of 7

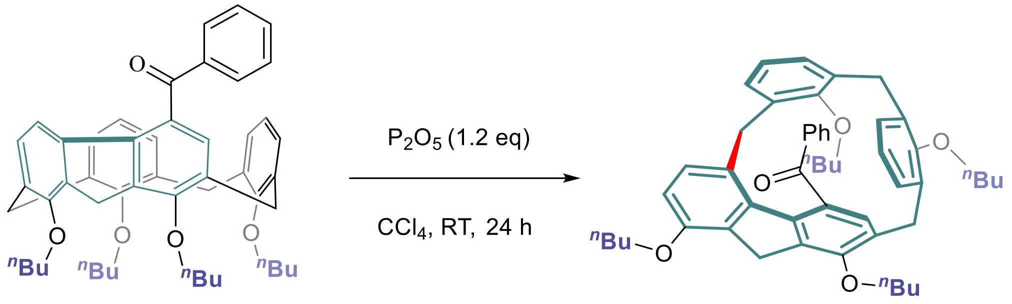

3a, 94% *ee*

7, 29% yield, 94% *ee*

To a dry round bottom flask was added **3a** (20 mg, 0.026 mmol, 1 equiv, 94% *ee*), P_2O_5 (4.4 mg, 0.031mmol, 1.2 equiv), and CCl_4 (6.5 mL). The reaction mixture was stirred at room temperature for 24 h before a saturated solution of $NaHCO_3$ was added. The reaction mixture was extracted with DCM (3 × 20 mL). The combined organic layers were washed with brine and dried over Na_2SO_4. Solvents were removed in *vacuo* and the crude product was purified by preparative TLC (PE : EA = 30 : 1) to give the pure product **7** (5.8 mg, 29% yield, 94% *ee*), white solid, **m.p.** = 125-127 °C. **^{1}H NMR** (400 MHz, $CDCl_3$, 298 K) δ 7.35 (dd, J = 7.4, 1.8 Hz, 1H), 7.28 (t, J = 7.5 Hz, 1H), 7.13-7.09 (m, 2H), 7.02-6.92 (m, 4H), 6.74 (d, J = 8.2 Hz, 1H), 6.53 (s, 1H), 6.34-6.26 (m, 4H), 4.41 (d, J = 19.3 Hz, 1H), 4.29 (d, J = 14.4 Hz, 1H), 4.22-4.03 (m, 6H), 3.93 (d, J = 22.3 Hz, 1H), 3.85 (d, J = 22.3 Hz, 1H), 3.73 (td, J = 9.5, 5.7 Hz, 1H), 3.54 (td, J = 9.5, 3.9 Hz, 1H), 3.36 (d, J = 14.4 Hz, 1H), 3.14 (d, J = 11.9 Hz, 1H), 2.87-2.81 (m, 1H), 2.52-2.46 (m, 1H), 1.91-1.79 (m, 4H), 1.69-1.51 (m, 5H), 1.30-1.21 (m, 3H), 1.08-1.00 (m, 6H), 0.90 (t, J = 7.4 Hz, 3H), 0.89-0.79 (m, 3H), 0.70 (t, J = 7.4 Hz, 3H), 0.29-0.17 (m, 1H). **^{13}C NMR** (100 MHz, $CDCl_3$, 298 K) δ 193.7, 158.1, 157.0, 156.4, 154.0, 144.8, 144.0, 139.9, 136.8, 136.7, 136.6, 136.4, 136.0, 132.2, 131.7, 131.6, 130.9, 130.8, 130.5, 130.2, 129.0, 128.4, 128.3, 128.2, 128.0, 127.2, 124.0, 122.7, 108.7, 75.2, 74.6, 74.4, 68.0, 41.7, 32.8, 32.7, 32.4, 32.1, 31.7, 31.0, 29.6, 19.6, 19.5, 19.2, 18.9, 14.2, 14.12, 14.11, 14.0. **IR** (KBr) v 3359, 2957, 2922, 2852, 1642, 1556, 1455, 1262, 1207, 1091, 1021, 799 cm^{-1}. **HRMS** (ESI) m/z calcd. for $C_{51}H_{59}O_5^+$ $[M+H]^+$ 751.43570; Found: 751.43707. **HPLC**: IB column, Hexane : *i*-PrOH= 95 : 5 , 24 °C, 0.5 mL/min

flow rate, detection at 330 nm, t_1 = 16.5 min (major), t_2 = 20.7 min (minor). **$[\alpha]_D^{27}$** = +606 (c = 0.5, $CHCl_3$) for 94% *ee*.

10. Copies of HPLC chromatograms

rac-**3a**

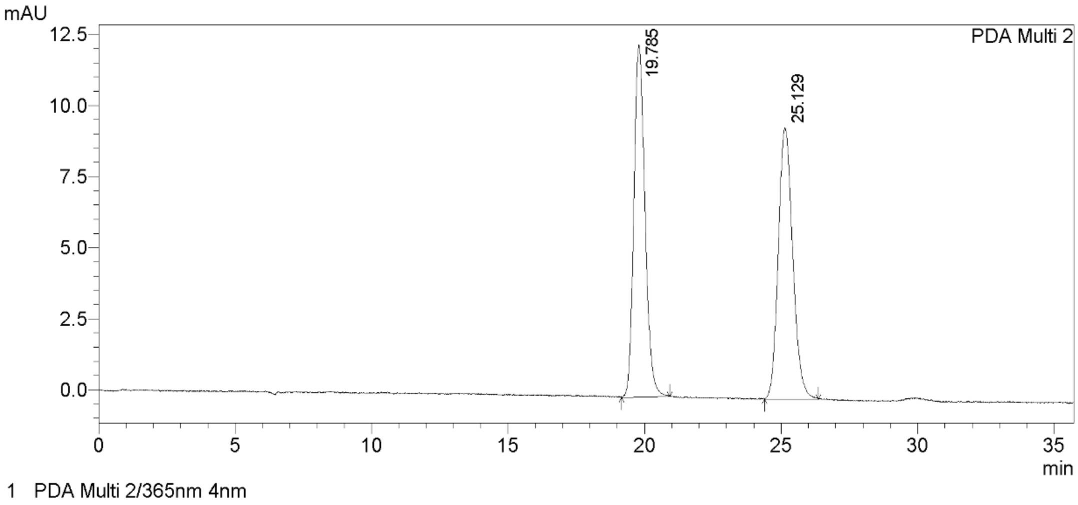

PeakTable

PDA Ch2 365nm 4nm

Peak#	Ret. Time	Area	Height	Area %	Height %
1	19.785	351300	12385	50.019	56.480
2	25.129	351035	9543	49.981	43.520
Total		702335	21928	100.000	100.000

P-**3a**

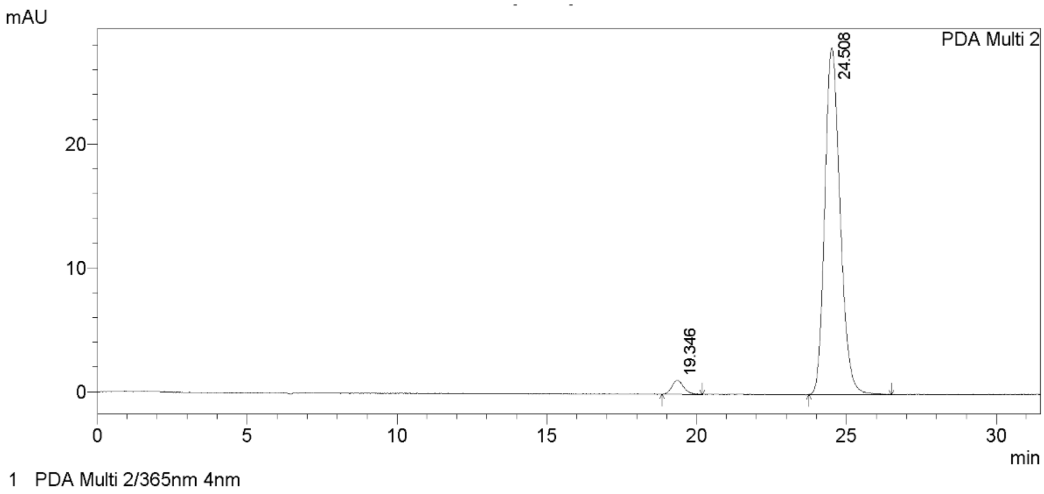

PeakTable

PDA Ch2 365nm 4nm

Peak#	Ret. Time	Area	Height	Area %	Height %
1	19.346	30829	1140	2.992	3.912
2	24.508	999610	28005	97.008	96.088
Total		1030440	29145	100.000	100.000

M-**3a**

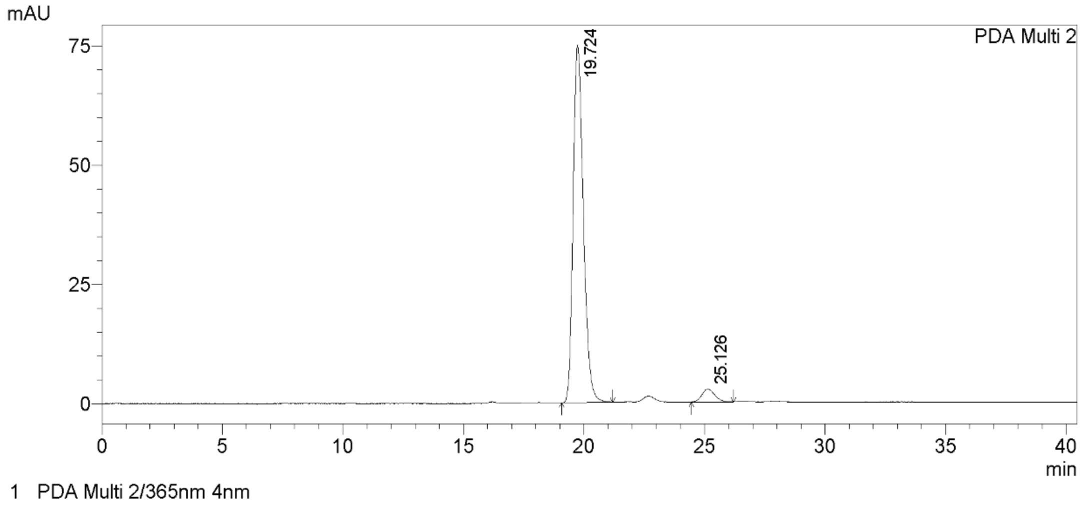

PeakTable

PDA Ch2 365nm 4nm

Peak#	Ret. Time	Area	Height	Area %	Height %
1	19.724	2215359	74995	95.511	96.465
2	25.126	104122	2748	4.489	3.535
Total		2319482	77743	100.000	100.000

3b

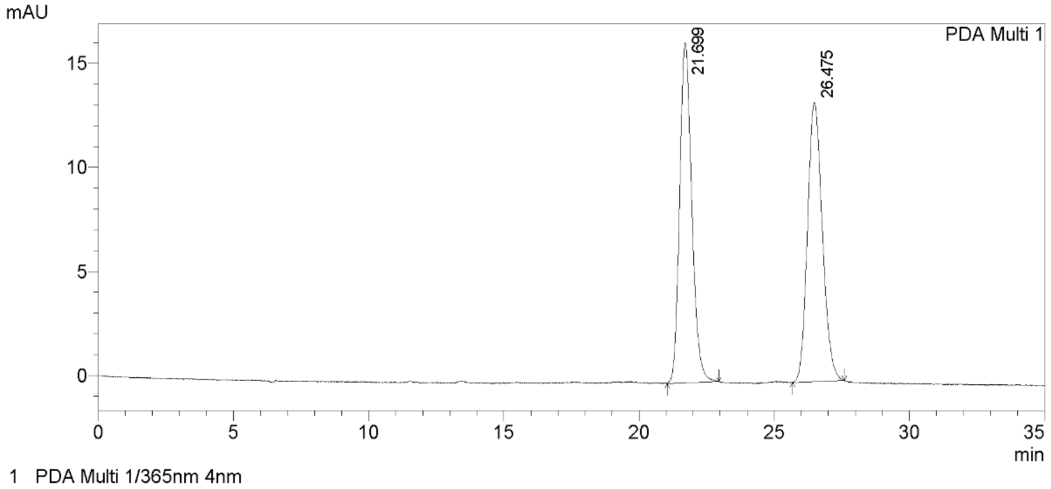

PeakTable

PDA Ch1 365nm 4nm

Peak#	Ret. Time	Area	Height	Area %	Height %
1	21.699	511149	16337	49.934	54.894
2	26.475	512494	13424	50.066	45.106
Total		1023643	29760	100.000	100.000

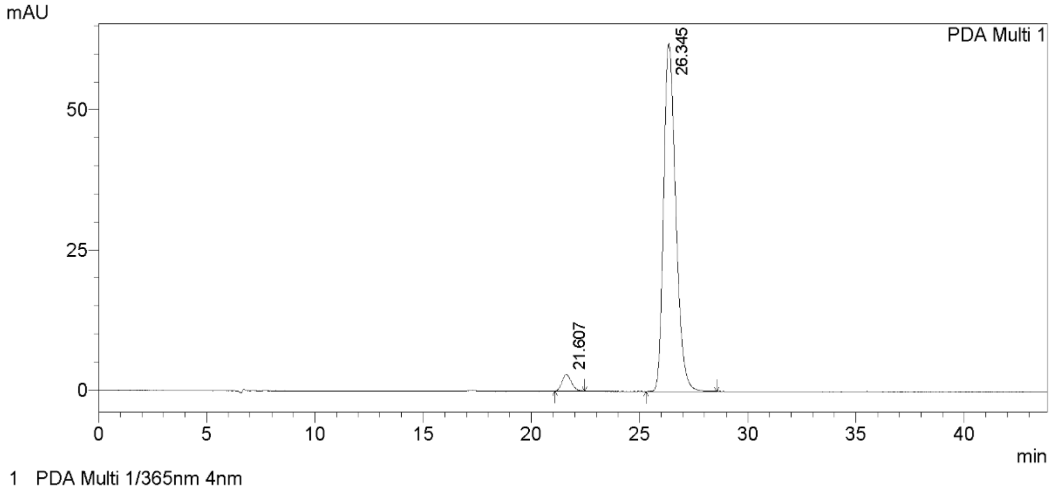

PeakTable

PDA Ch1 365nm 4nm

Peak#	Ret. Time	Area	Height	Area %	Height %
1	21.607	89800	2975	3.576	4.570
2	26.345	2421154	62127	96.424	95.430
Total		2510954	65103	100.000	100.000

3c

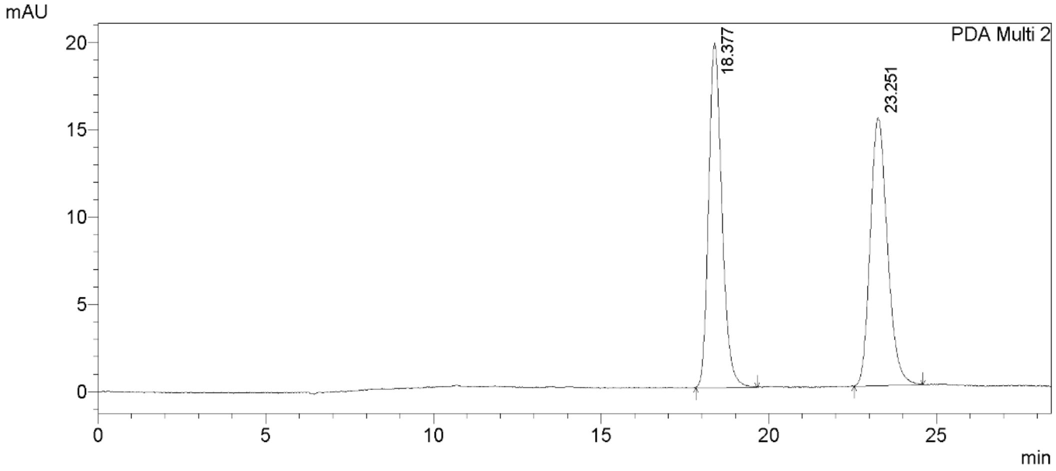

1 PDA Multi 2/365nm 4nm

PeakTable

PDA Ch2 365nm 4nm

Peak#	Ret. Time	Area	Height	Area %	Height %
1	18.377	546222	19758	49.964	56.296
2	23.251	547003	15339	50.036	43.704
Total		1093225	35096	100.000	100.000

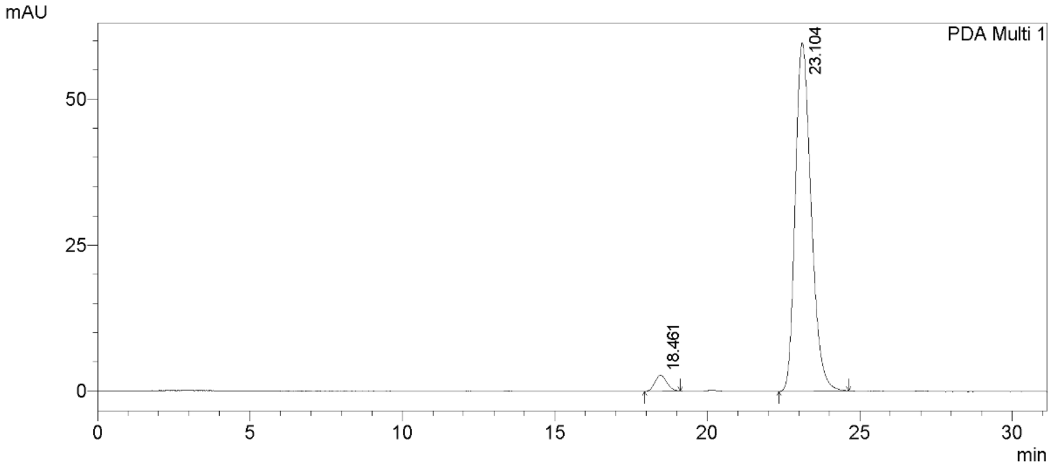

1 PDA Multi 1/365nm 4nm

PeakTable

PDA Ch1 365nm 4nm

Peak#	Ret. Time	Area	Height	Area %	Height %
1	18.461	75440	2784	3.368	4.450
2	23.104	2164232	59769	96.632	95.550
Total		2239672	62553	100.000	100.000

3d

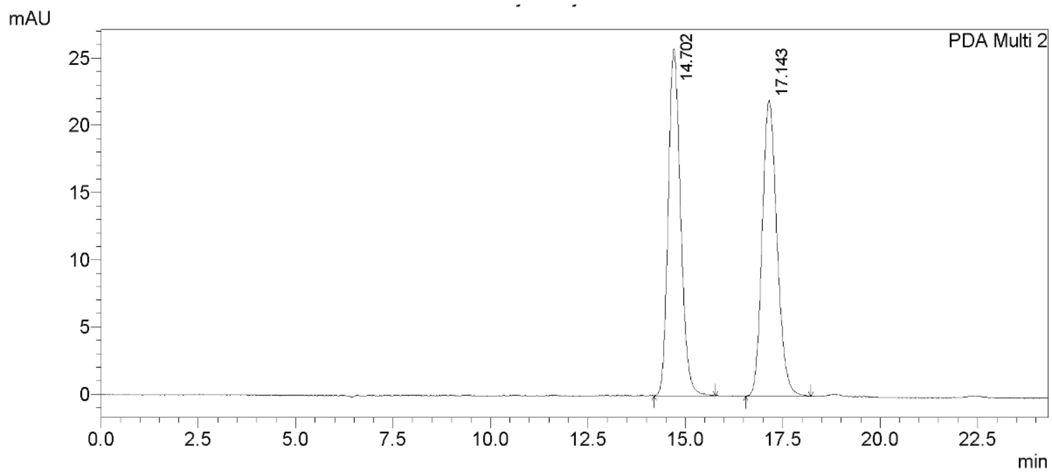

1 PDA Multi 2/365nm 4nm

PeakTable

PDA Ch2 365nm 4nm

Peak#	Ret. Time	Area	Height	Area %	Height %
1	14.702	570765	25765	49.451	53.936
2	17.143	583441	22004	50.549	46.064
Total		1154205	47769	100.000	100.000

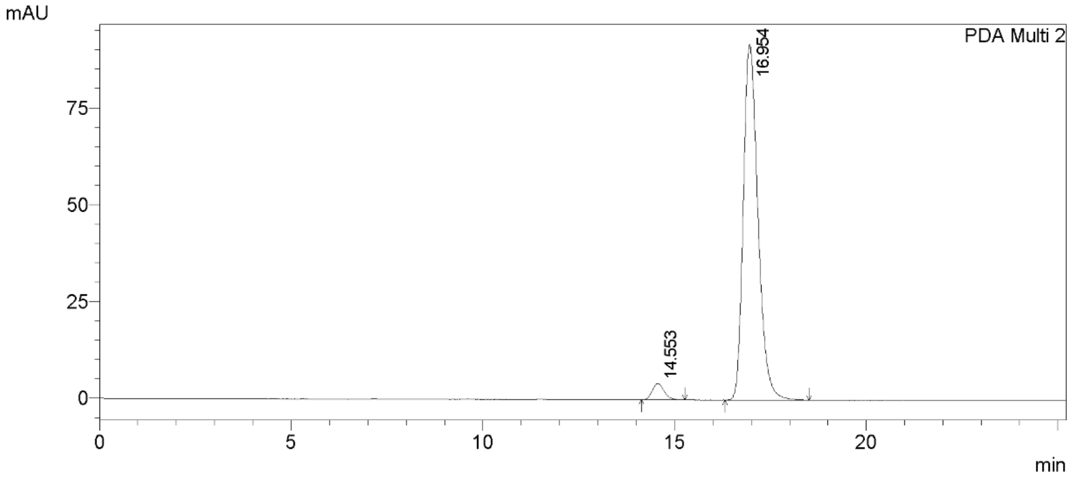

1 PDA Multi 2/365nm 4nm

PeakTable

PDA Ch2 365nm 4nm

Peak#	Ret. Time	Area	Height	Area %	Height %
1	14.553	89870	4223	3.561	4.397
2	16.954	2434138	91817	96.439	95.603
Total		2524008	96040	100.000	100.000

3e

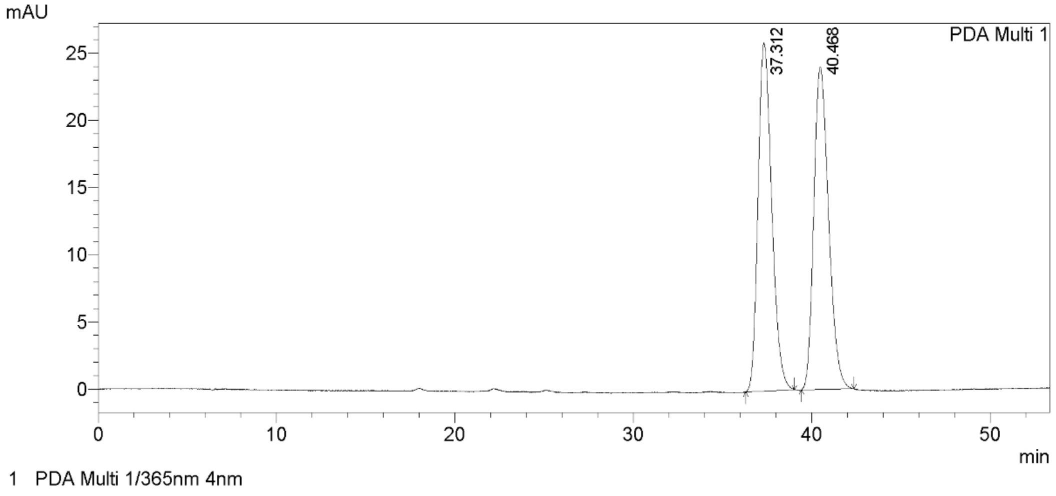

PeakTable

PDA Ch1 365nm 4nm

Peak#	Ret. Time	Area	Height	Area %	Height %
1	37.312	1388184	25909	50.091	51.910
2	40.468	1383150	24002	49.909	48.090
Total		2771334	49911	100.000	100.000

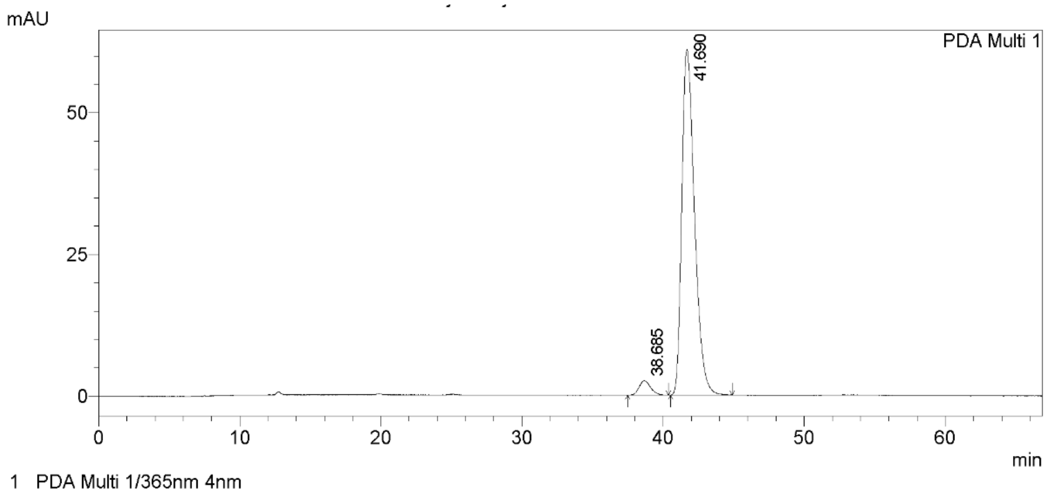

PeakTable

PDA Ch1 365nm 4nm

Peak#	Ret. Time	Area	Height	Area %	Height %
1	38.685	144824	2562	3.650	4.033
2	41.690	3823113	60959	96.350	95.967
Total		3967937	63520	100.000	100.000

3f

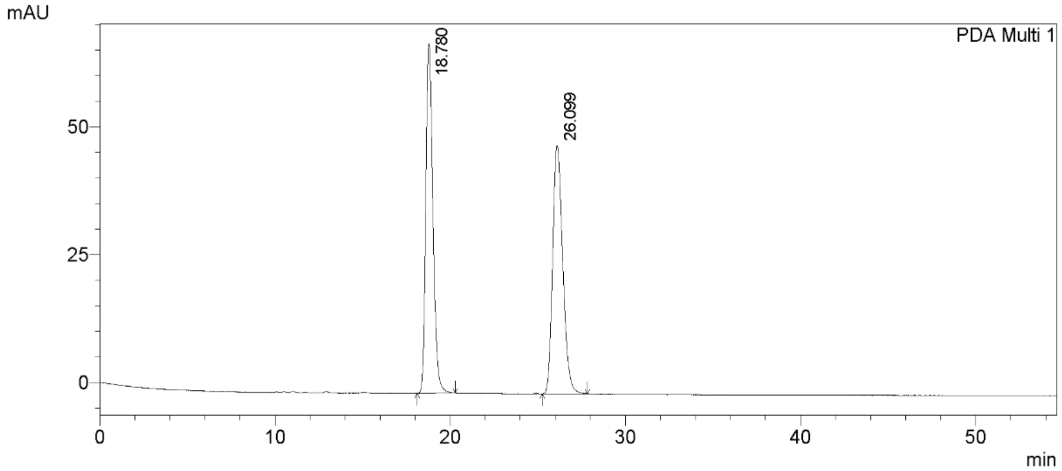

1 PDA Multi 1/365nm 4nm

PeakTable

PDA Ch1 365nm 4nm

Peak#	Ret. Time	Area	Height	Area %	Height %
1	18.780	1932070	68248	49.866	58.462
2	26.099	1942420	48490	50.134	41.538
Total		3874490	116738	100.000	100.000

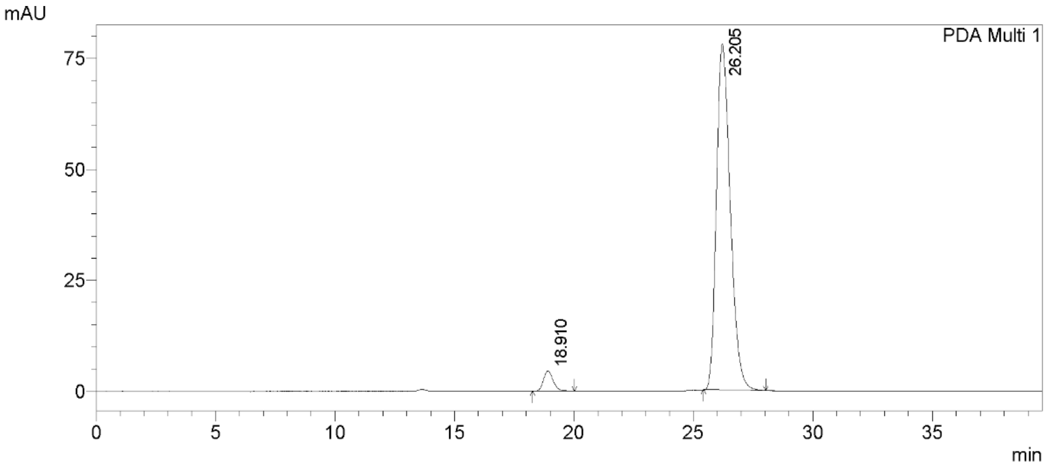

1 PDA Multi 1/365nm 4nm

PeakTable

PDA Ch1 365nm 4nm

Peak#	Ret. Time	Area	Height	Area %	Height %
1	18.910	127592	4555	3.906	5.527
2	26.205	3138818	77858	96.094	94.473
Total		3266410	82413	100.000	100.000

3g

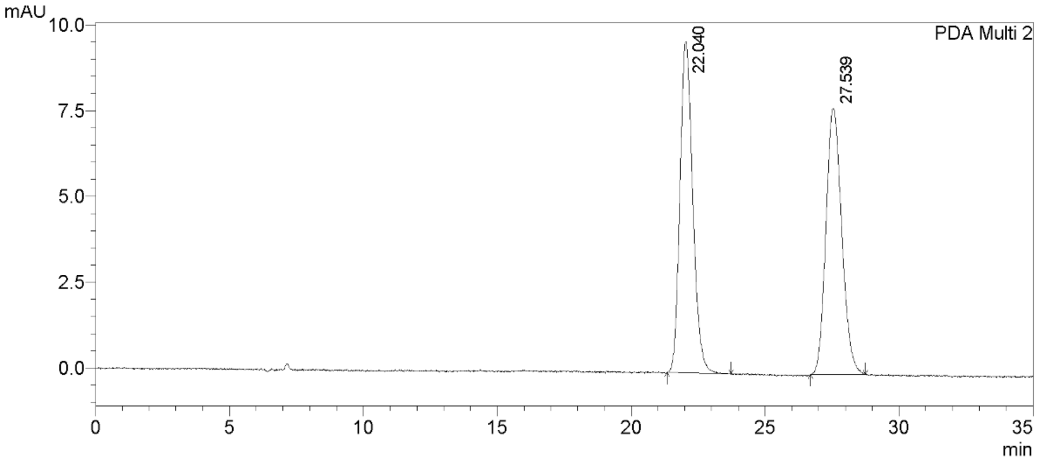

1 PDA Multi 2/365nm 4nm

PeakTable

PDA Ch2 365nm 4nm

Peak#	Ret. Time	Area	Height	Area %	Height %
1	22.040	321299	9647	50.148	55.404
2	27.539	319409	7765	49.852	44.596
Total		640708	17412	100.000	100.000

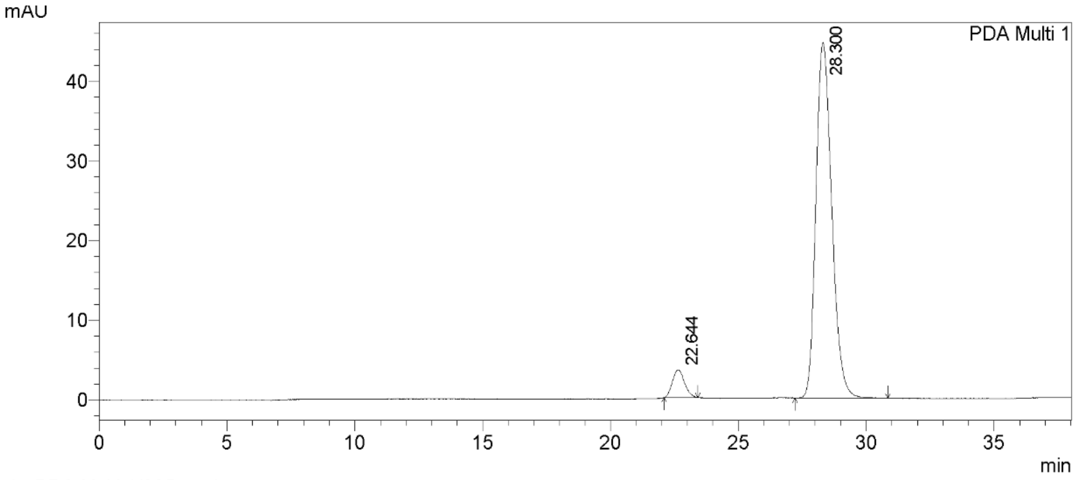

1 PDA Multi 1/365nm 4nm

PeakTable

PDA Ch1 365nm 4nm

Peak#	Ret. Time	Area	Height	Area %	Height %
1	22.644	114512	3466	5.631	7.207
2	28.300	1919263	44635	94.369	92.793
Total		2033776	48101	100.000	100.000

3h

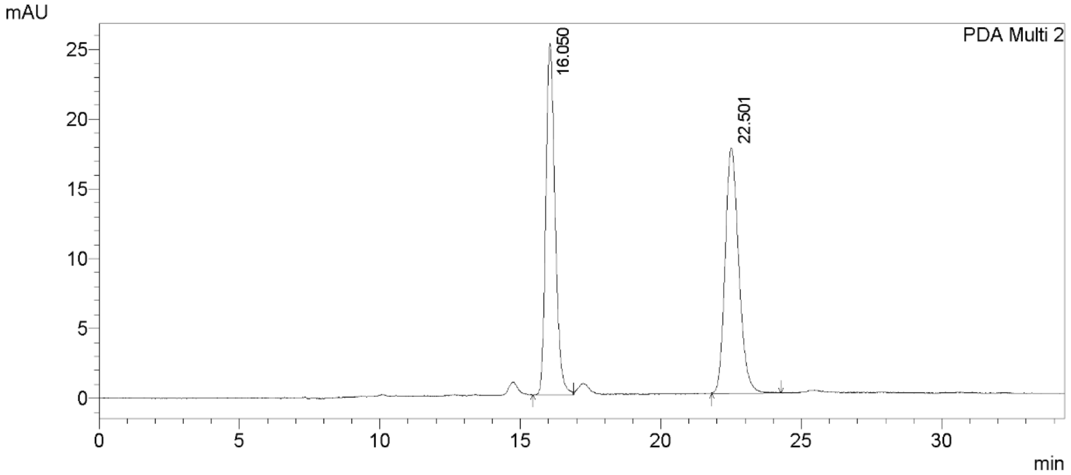

1 PDA Multi 2/365nm 4nm

PeakTable

PDA Ch2 365nm 4nm

Peak#	Ret. Time	Area	Height	Area %	Height %
1	16.050	574870	25153	49.169	58.822
2	22.501	594296	17608	50.831	41.178
Total		1169166	42761	100.000	100.000

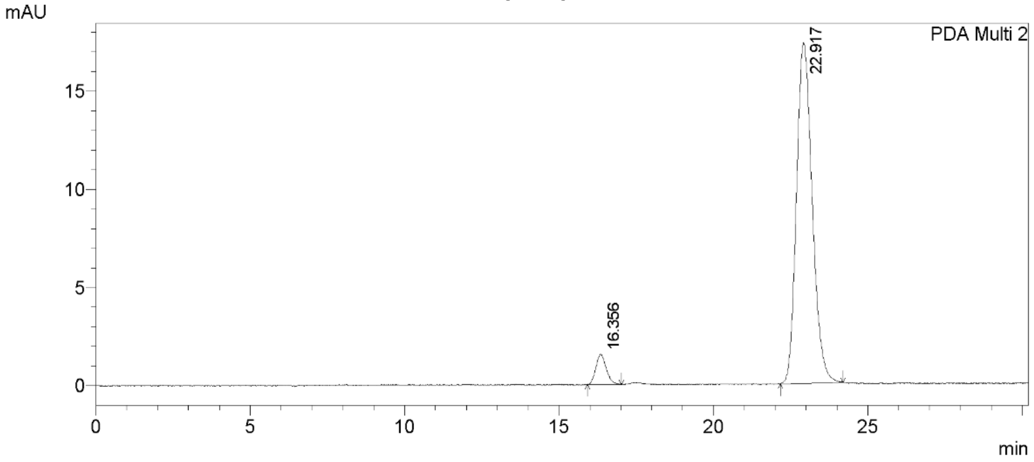

1 PDA Multi 2/365nm 4nm

PeakTable

PDA Ch2 365nm 4nm

Peak#	Ret. Time	Area	Height	Area %	Height %
1	16.356	36433	1546	5.579	8.175
2	22.917	616559	17369	94.421	91.825
Total		652991	18915	100.000	100.000

3i

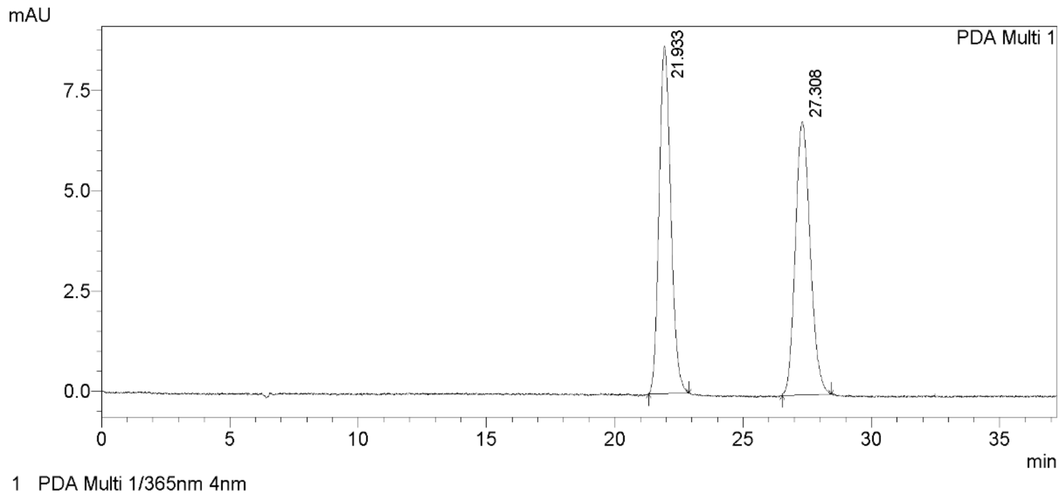

PeakTable

PDA Ch1 365nm 4nm

Peak#	Ret. Time	Area	Height	Area %	Height %
1	21.933	276727	8662	50.043	55.965
2	27.308	276251	6816	49.957	44.035
Total		552978	15478	100.000	100.000

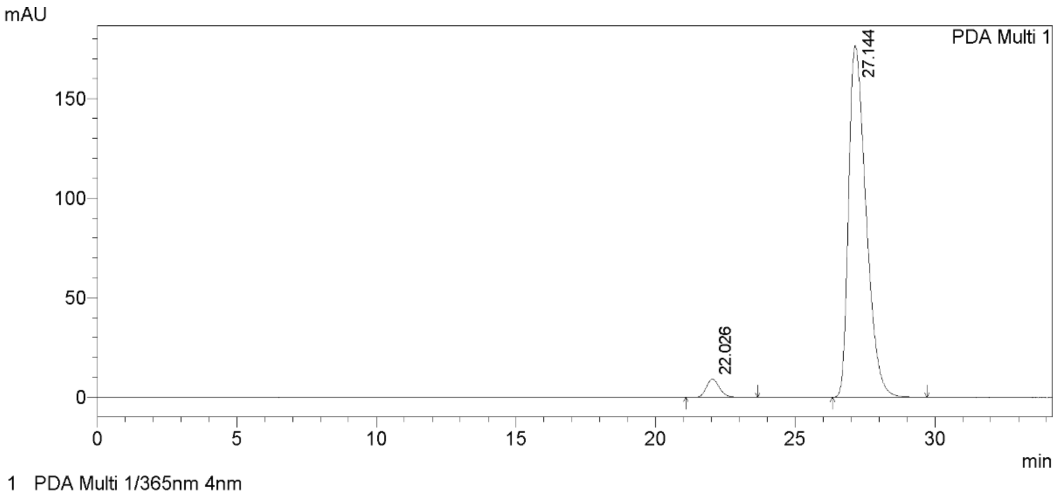

PeakTable

PDA Ch1 365nm 4nm

Peak#	Ret. Time	Area	Height	Area %	Height %
1	22.026	302631	9207	3.785	4.954
2	27.144	7693299	176630	96.215	95.046
Total		7995930	185836	100.000	100.000

3j

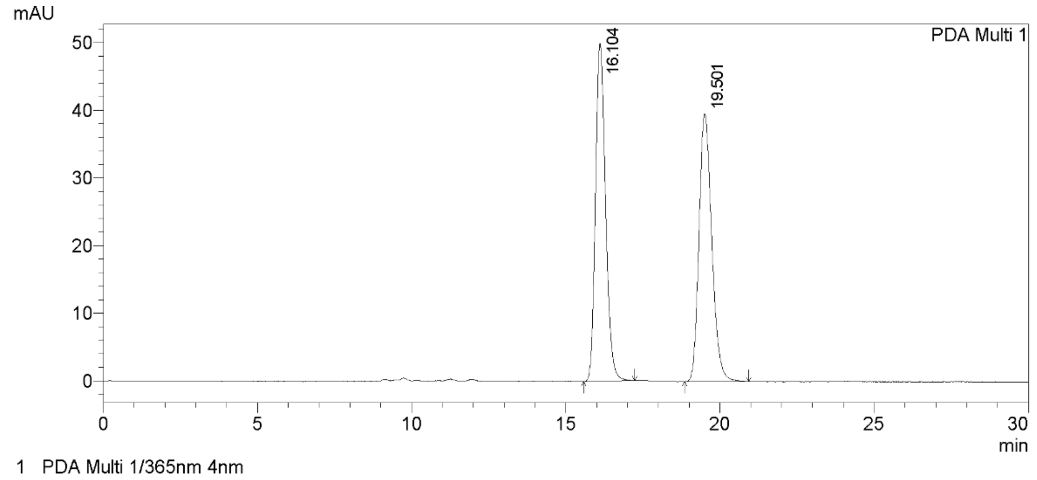

PeakTable

PDA Ch1 365nm 4nm

Peak#	Ret. Time	Area	Height	Area %	Height %
1	16.104	1140161	49957	50.007	55.757
2	19.501	1139864	39640	49.993	44.243
Total		2280025	89597	100.000	100.000

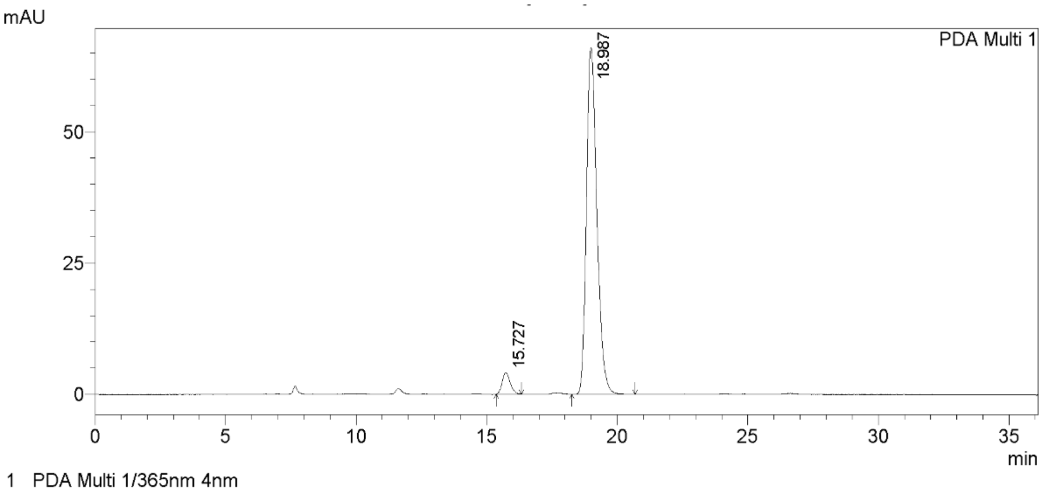

PeakTable

PDA Ch1 365nm 4nm

Peak#	Ret. Time	Area	Height	Area %	Height %
1	15.727	87813	4091	4.549	5.838
2	18.987	1842619	65977	95.451	94.162
Total		1930432	70068	100.000	100.000

3k

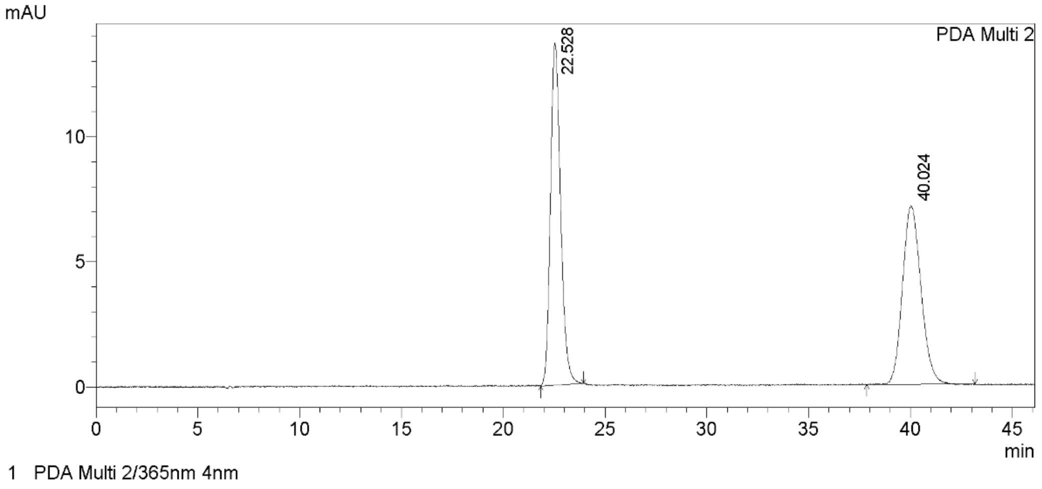

PeakTable

PDA Ch2 365nm 4nm

Peak#	Ret. Time	Area	Height	Area %	Height %
1	22.528	473309	13666	50.927	65.703
2	40.024	456073	7134	49.073	34.297
Total		929382	20800	100.000	100.000

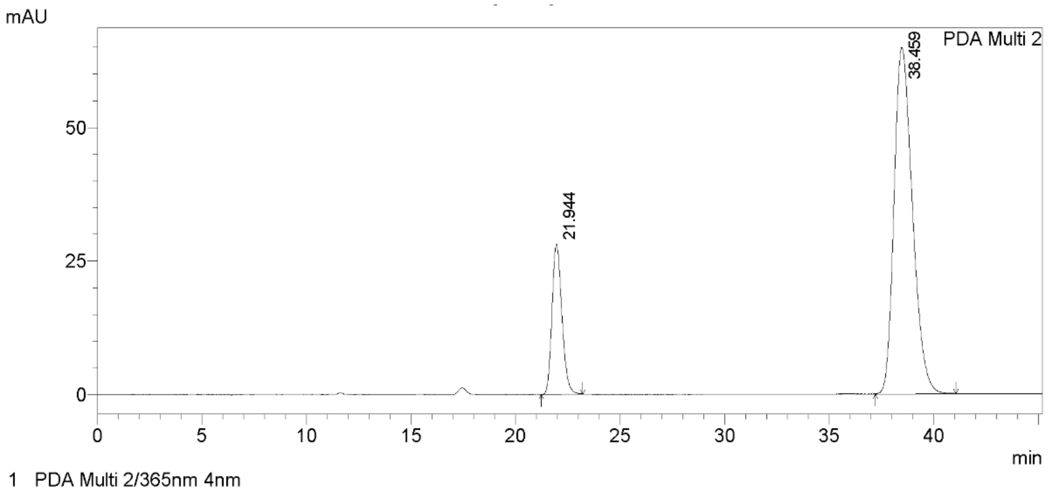

PeakTable

PDA Ch2 365nm 4nm

Peak#	Ret. Time	Area	Height	Area %	Height %
1	21.944	922057	28110	18.837	30.242
2	38.459	3972928	64840	81.163	69.758
Total		4894985	92950	100.000	100.000

5

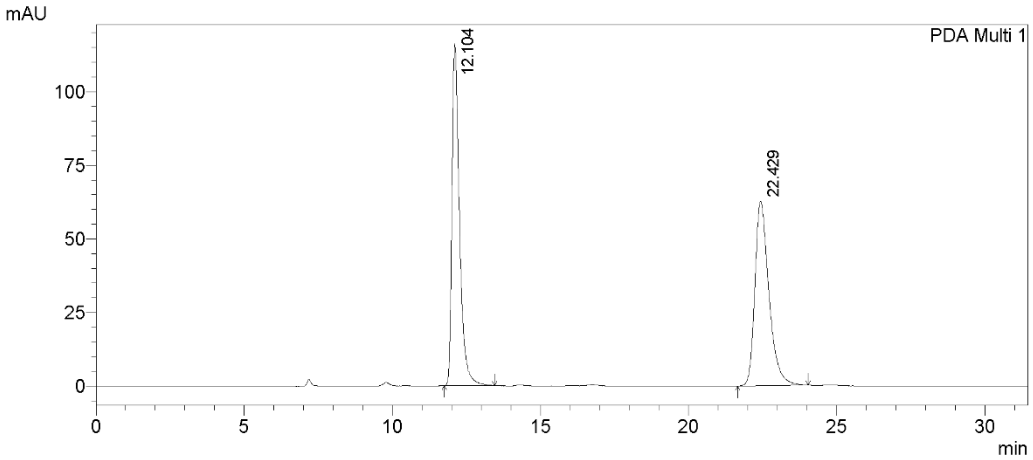

1 PDA Multi 1/330nm 4nm

PeakTable

PDA Ch1 330nm 4nm

Peak#	Ret. Time	Area	Height	Area %	Height %
1	12.104	2065894	116005	50.016	64.927
2	22.429	2064611	62663	49.984	35.073
Total		4130505	178668	100.000	100.000

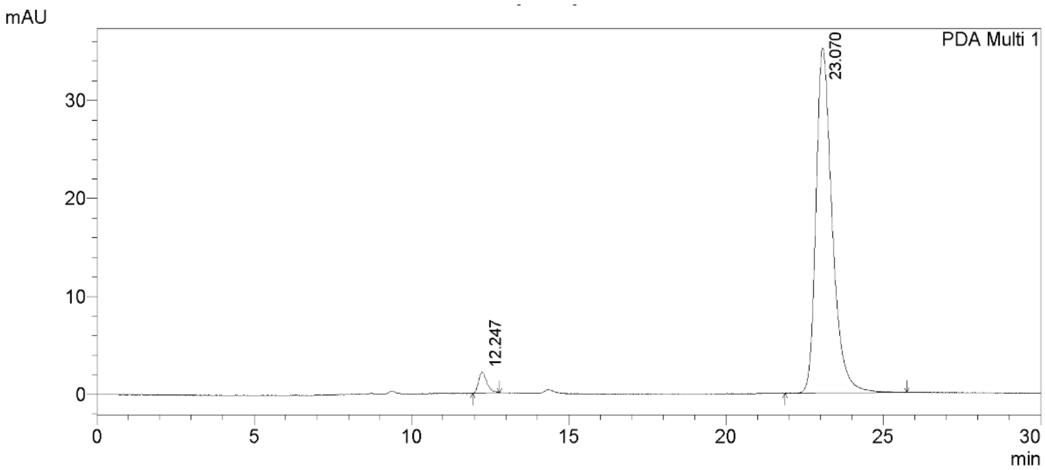

1 PDA Multi 1/330nm 4nm

PeakTable

PDA Ch1 330nm 4nm

Peak#	Ret. Time	Area	Height	Area %	Height %
1	12.247	39616	2164	3.057	5.788
2	23.070	1256502	35226	96.943	94.212
Total		1296118	37390	100.000	100.000

6

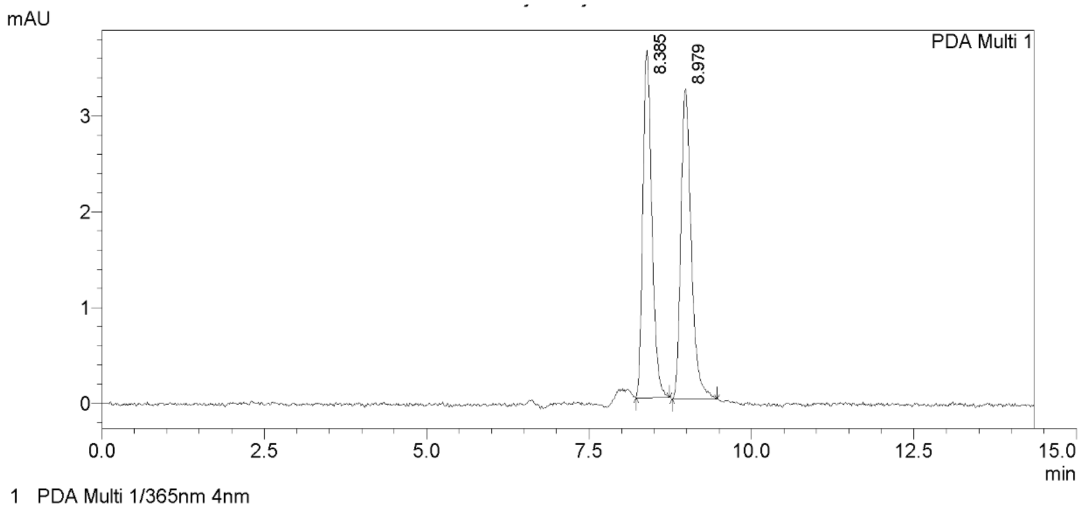

PeakTable

PDA Ch1 365nm 4nm

Peak#	Ret. Time	Area	Height	Area %	Height %
1	8.385	35031	3628	49.180	52.802
2	8.979	36199	3243	50.820	47.198
Total		71230	6872	100.000	100.000

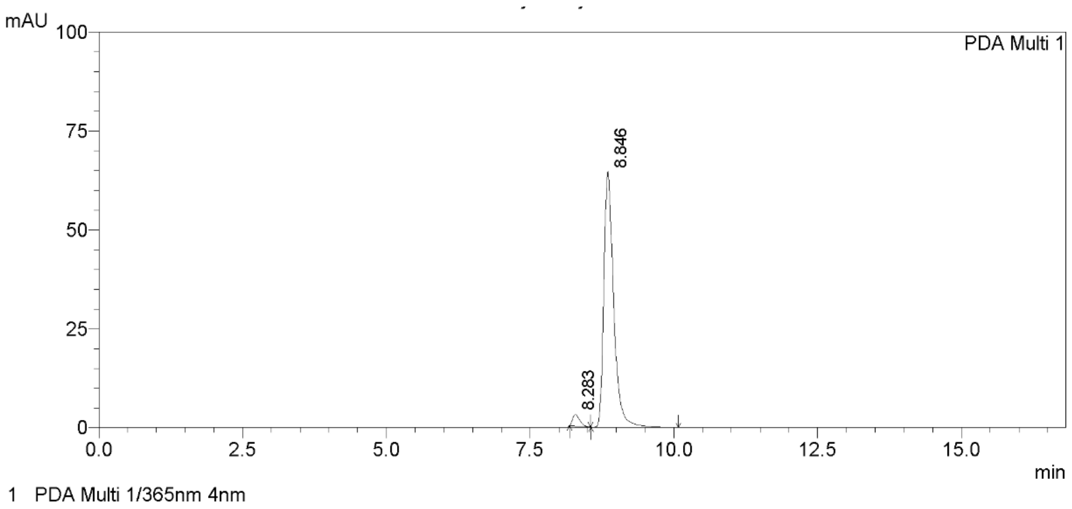

PeakTable

PDA Ch1 365nm 4nm

Peak#	Ret. Time	Area	Height	Area %	Height %
1	8.283	25367	2876	3.174	4.246
2	8.846	773717	64859	96.826	95.754
Total		799084	67736	100.000	100.000

7

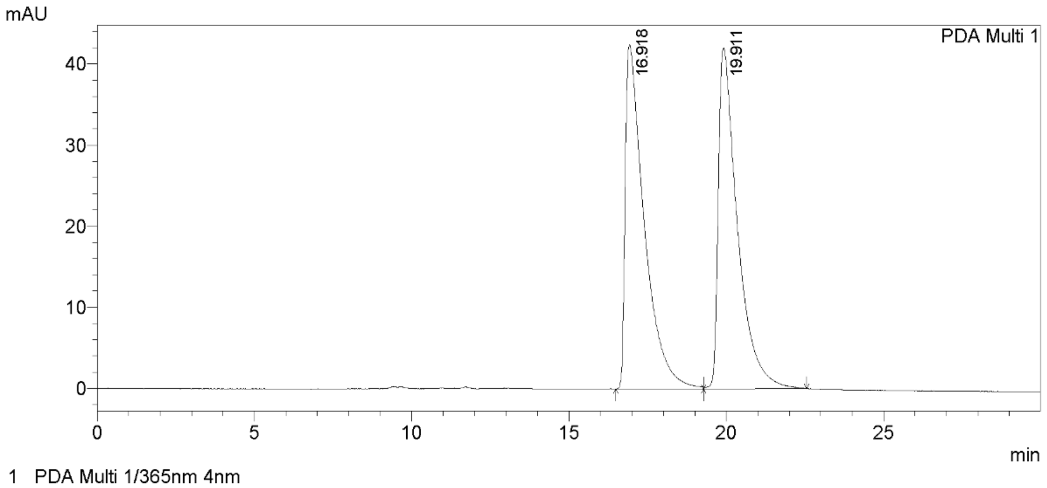

PeakTable

PDA Ch1 365nm 4nm

Peak#	Ret. Time	Area	Height	Area %	Height %
1	16.918	1786223	42458	50.136	50.216
2	19.911	1776506	42093	49.864	49.784
Total		3562729	84551	100.000	100.000

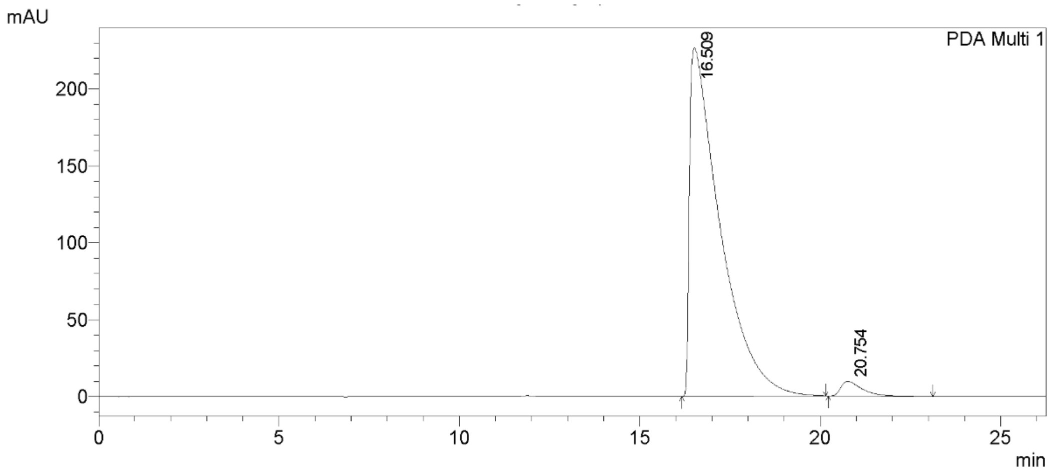

PeakTable

PDA Ch1 365nm 4nm

Peak#	Ret. Time	Area	Height	Area %	Height %
1	16.509	13355763	226798	97.050	95.913
2	20.754	405938	9664	2.950	4.087
Total		13761701	236462	100.000	100.000

11. Inversion barrier test of 3e

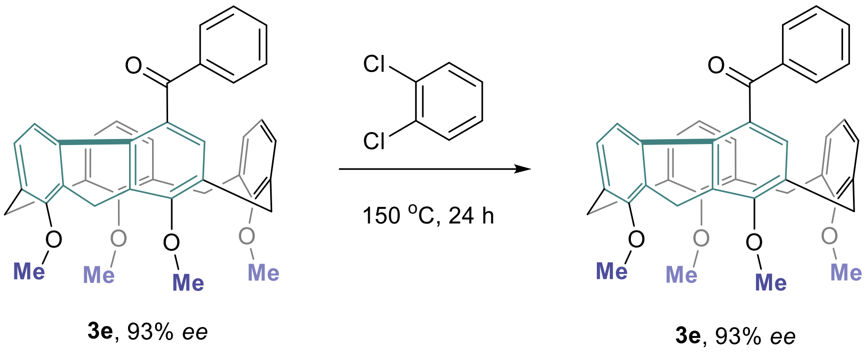

0 h

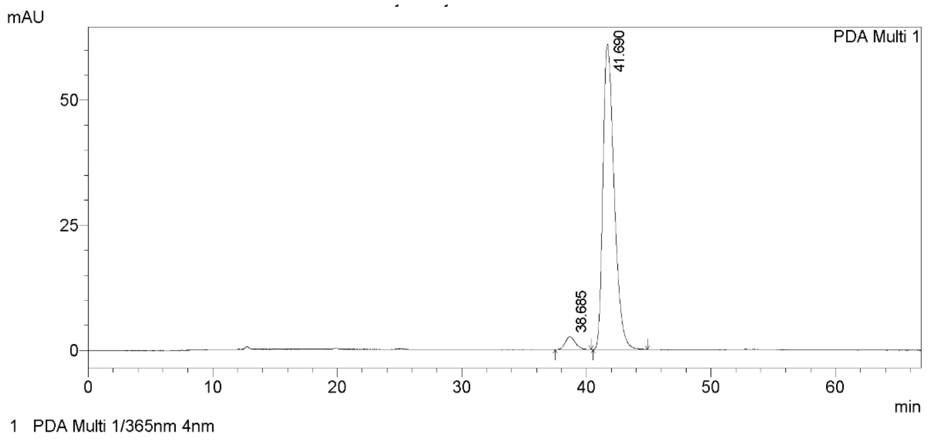

PeakTable

PDA Ch1 365nm 4nm

Peak#	Ret. Time	Area	Height	Area %	Height %
1	38.685	144824	2562	3.650	4.033
2	41.690	3823113	60959	96.350	95.967
Total		3967937	63520	100.000	100.000

24 h

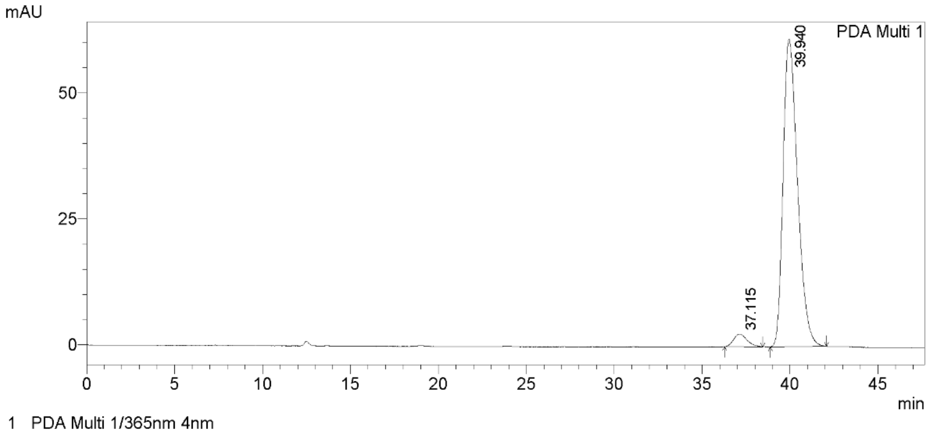

PeakTable

PDA Ch1 365nm 4nm

Peak#	Ret. Time	Area	Height	Area %	Height %
1	37.115	133229	2507	3.682	3.947
2	39.940	3485108	60994	96.318	96.053
Total		3618337	63501	100.000	100.000

12. Crystallographic data of *P*-3a

Crystal data and X-ray molecular structures with their CCDC numbers are reported as follows. CIFs and CheckCIFs are provided in separated files as Supplementary Information. Following each Table of crystal data and structure refinement for compounds.

Table S1. Crystal data and structure refinement for *P*-**3a**.

Identification code	exp_3578
Empirical formula	$C_{51}H_{58}O_5$
Formula weight	750.97
Temperature/K	100.2(5)
Crystal system	triclinic
Space group	P1
a/Å	10.6821(2)
b/Å	12.08420(10)
c/Å	17.0570(2)
α/°	93.1420(10)
β/°	105.2290(10)
γ/°	97.6850(10)
Volume/Å^3	2096.06(5)
Z	2
ρ_{calc}g/cm^3	1.190
μ/mm^{-1}	0.586
F(000)	808.0
Crystal size/mm^3	0.5 × 0.2 × 0.05
Radiation	CuKα (λ = 1.54184)
2Θ range for data collection/°	7.414 to 133.118
Index ranges	$-12 \leq h \leq 12$, $-14 \leq k \leq 14$, $-20 \leq l \leq 20$
Reflections collected	36384
Independent reflections	12156 [R_{int} = 0.0397, R_{sigma} = 0.0416]
Data/restraints/parameters	12156/45/1044
Goodness-of-fit on F^2	1.085
Final R indexes [I>=2σ (I)]	R_1 = 0.0541, wR_2 = 0.1401
Final R indexes [all data]	R_1 = 0.0630, wR_2 = 0.1667
Largest diff. peak/hole / e Å^{-3}	0.70/-0.56
Flack parameter	-0.01(10)
CCDC	2216266

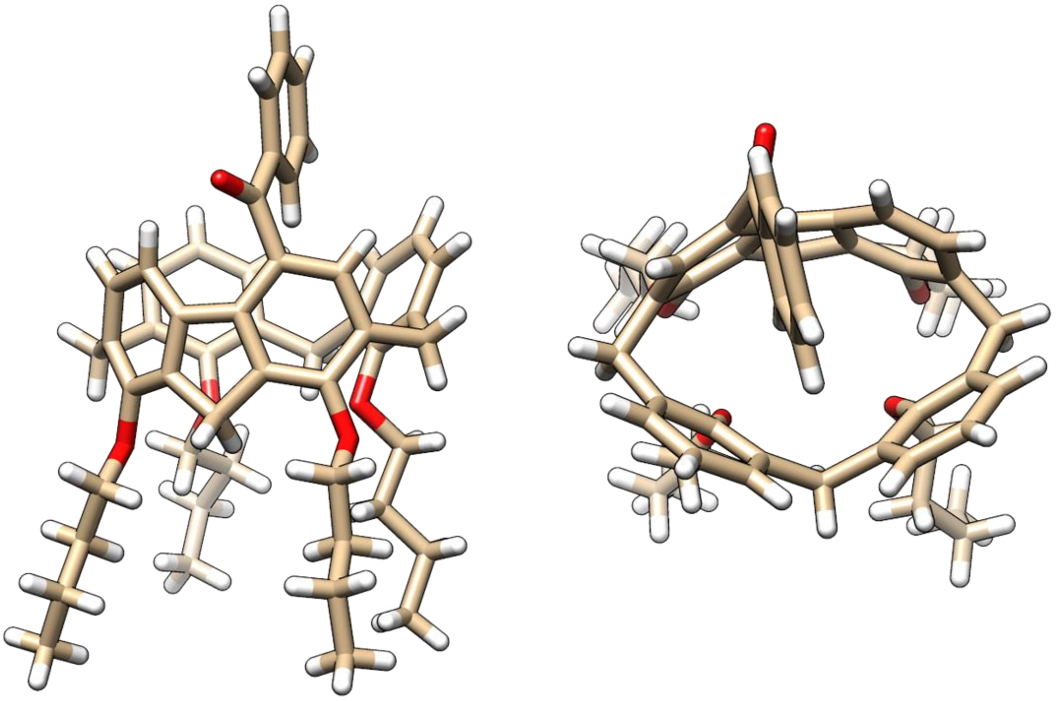

Datablock exp_3578 - ellipsoid plot

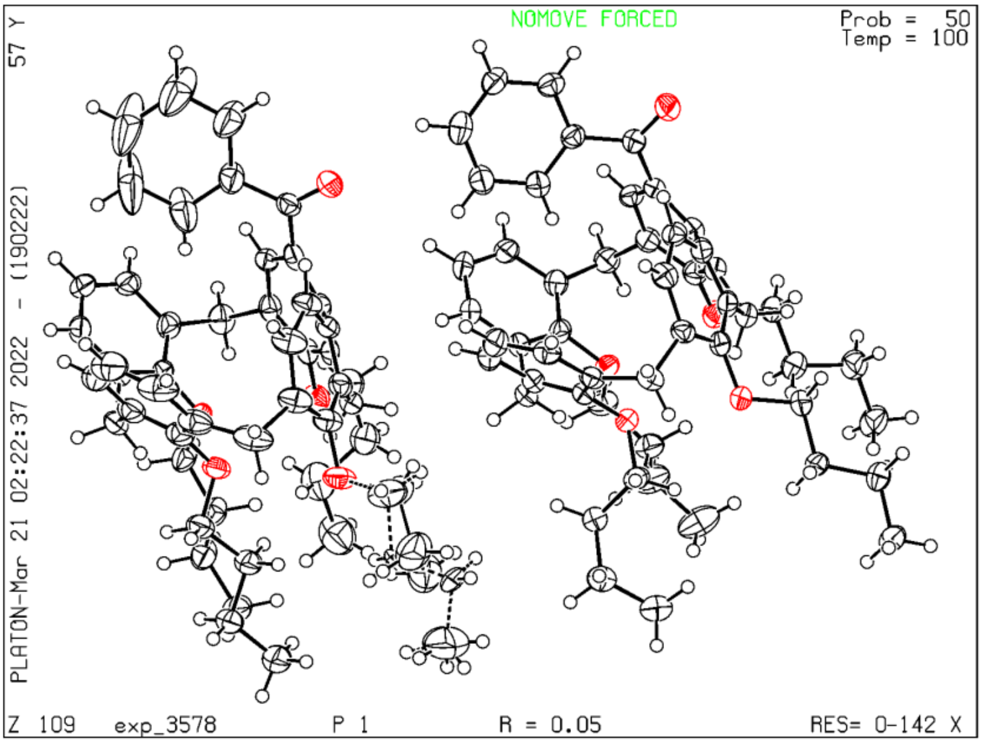

Figure S1. X-ray molecular structures of *P*-**3a.** Oak ridge thermal ellipsoid plot (ORTEP) diagram of *P*-**3a**. Thermal ellipsoids are shown at the 50% probability level.

13. Crystallographic data of *rac*-7

14. Table S2. Crystal data and structure refinement for *rac*-7.

Identification code	zx-cp-rac_auto
Empirical formula	$C_{51}H_{56}O_5$
Formula weight	748.95
Temperature/K	172.99(10)
Crystal system	triclinic
Space group	P-1
a/Å	9.35900(10)
b/Å	12.70520(10)
c/Å	18.77490(10)
α/°	80.2660(10)
β/°	79.8290(10)
γ/°	76.8500(10)
Volume/Å^3	2120.34(3)
Z	2
ρ_{calc}g/cm^3	1.173
μ/mm^{-1}	0.579
F(000)	804.0
Crystal size/mm^3	0.2 × 0.15 × 0.1
Radiation	Cu Kα (λ = 1.54184)
2Θ range for data collection/°	4.826 to 155.582
Index ranges	$-11 \le h \le 11, -15 \le k \le 16, -23 \le l \le 23$
Reflections collected	75676
Independent reflections	8594 [R_{int} = 0.0357, R_{sigma} = 0.0147]
Data/restraints/parameters	8594/0/528
Goodness-of-fit on F^2	1.078
Final R indexes [I>=2σ (I)]	R_1 = 0.0620, wR_2 = 0.1623
Final R indexes [all data]	R_1 = 0.0683, wR_2 = 0.1663
Largest diff. peak/hole / e Å^{-3}	0.79/-0.60
CCDC	2216267

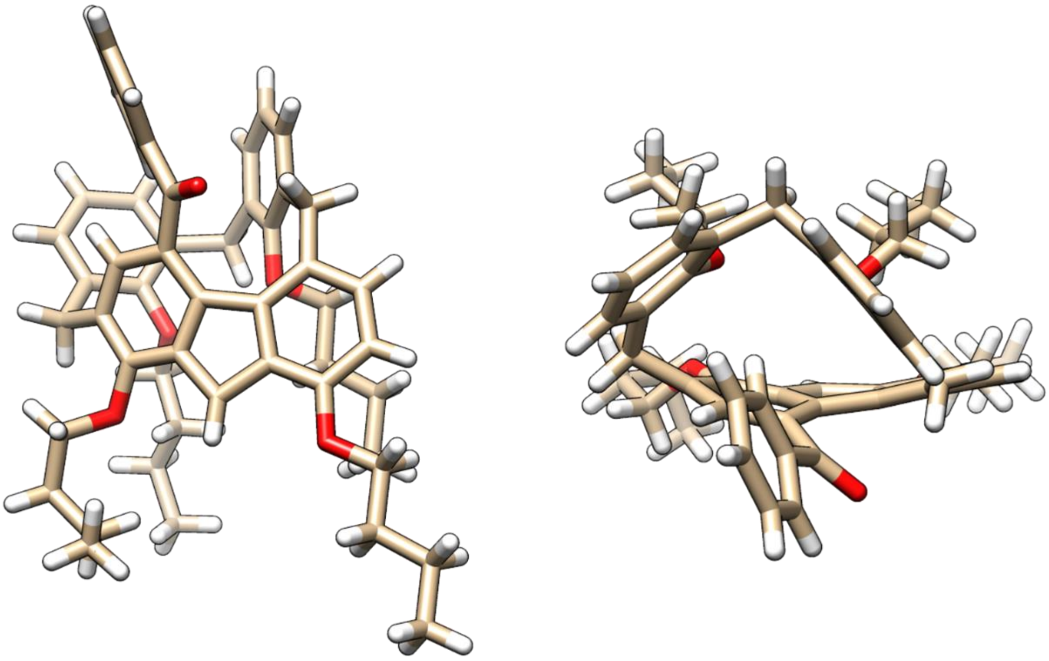

Datablock zx-cp-rac_auto - ellipsoid plot

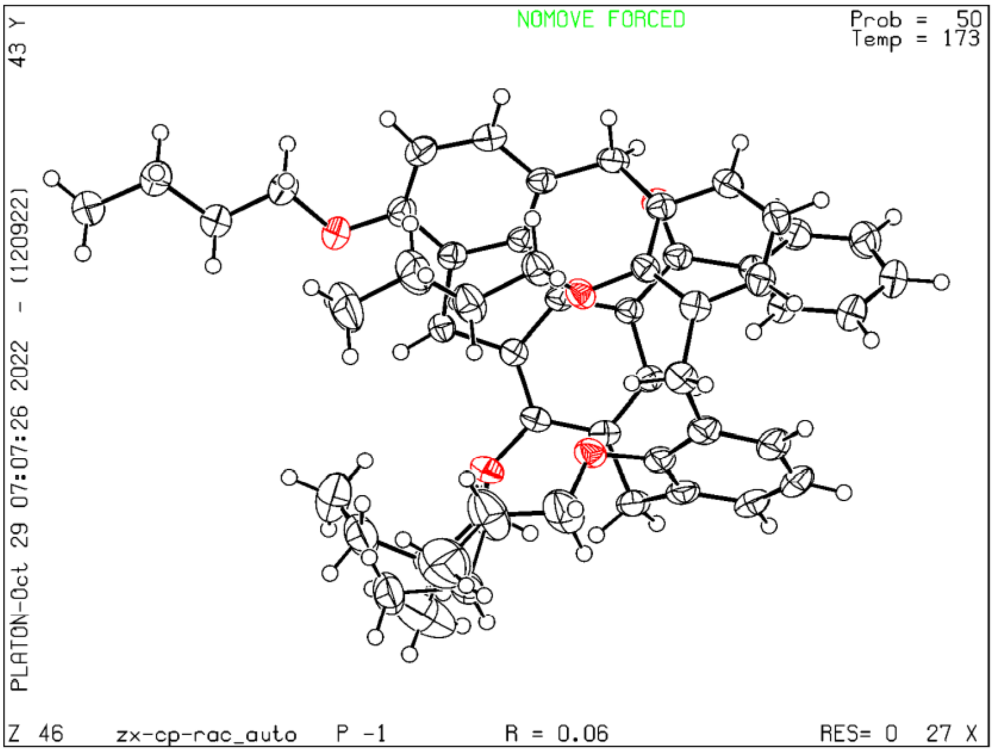

Figure S2. X-ray molecular structures of *rac*-**7.** Oak ridge thermal ellipsoid plot (ORTEP) diagram of *rac*-**7**. Thermal ellipsoids are shown at the 50% probability level.

15. Copies of UV-vis spectra of 3a-3k, 5, 6, and 7 in different solvents (CH_3CN, DCM, and toluene)

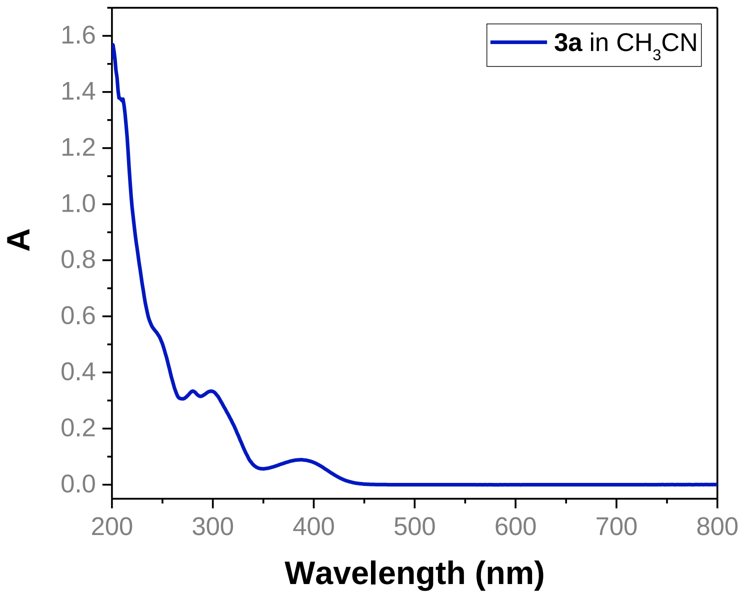

Figure S3. UV-Vis spectrum of **3a** in CH_3CN at 25 °C, the concentration was ca. $3{\times}10^{-5}$ M

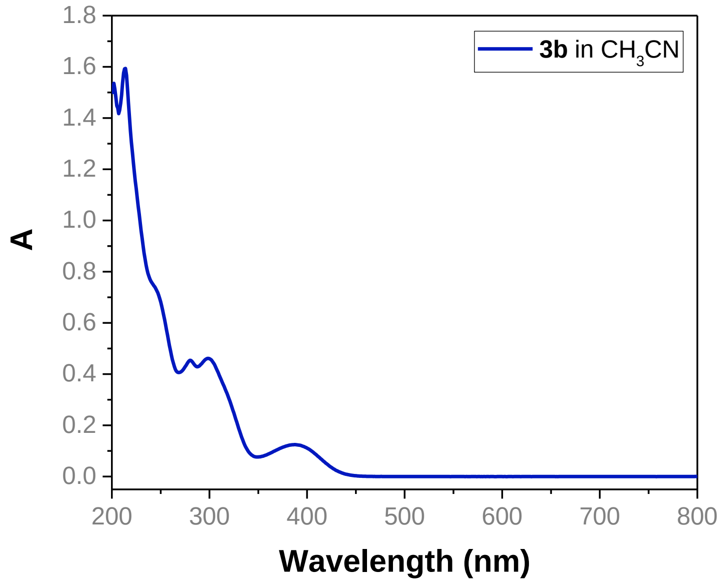

Figure S4. UV-Vis spectrum of **3b** in CH_3CN at 25 °C, the concentration was ca. $3{\times}10^{-5}$ M

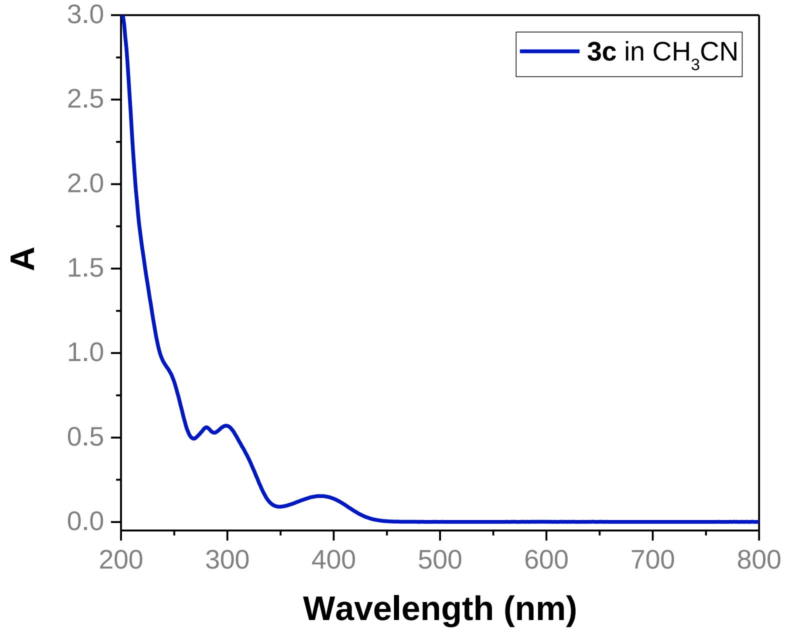

Figure S5. UV-Vis spectrum of **3c** in CH_3CN at 25 °C, the concentration was ca. 3×10^{-5} M

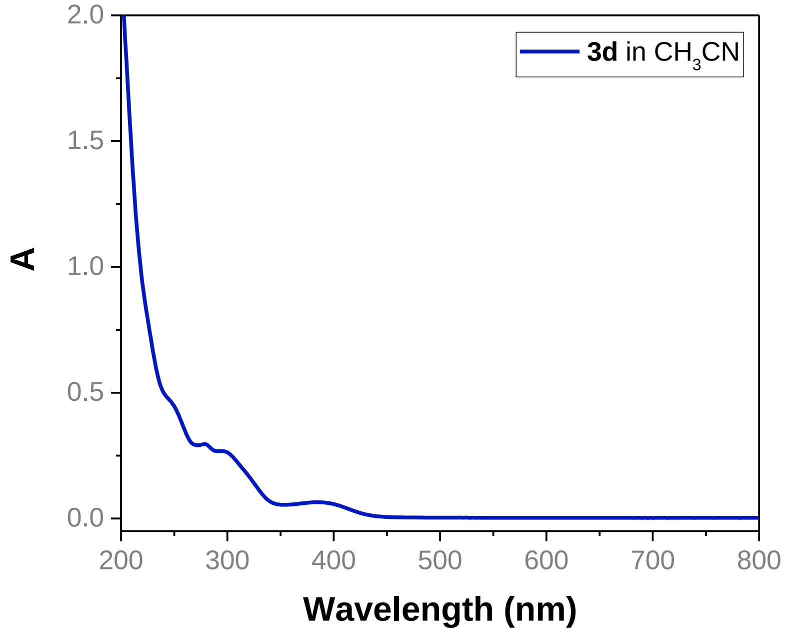

Figure S6. UV-Vis spectrum of **3d** in CH_3CN at 25 °C, the concentration was ca. 3×10^{-5} M

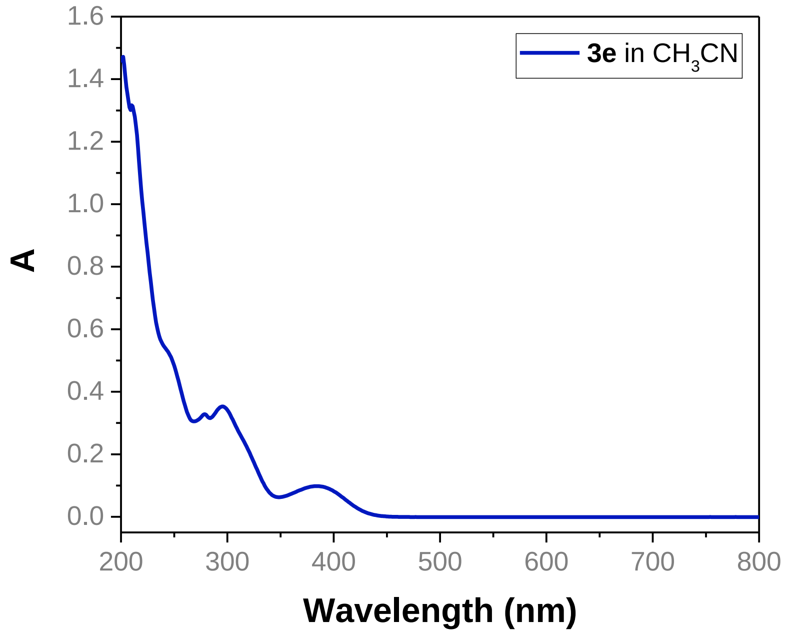

Figure S7. UV-Vis spectrum of **3e** in CH_3CN at 25 ºC, the concentration was ca. $3{\times}10^{-5}$ M

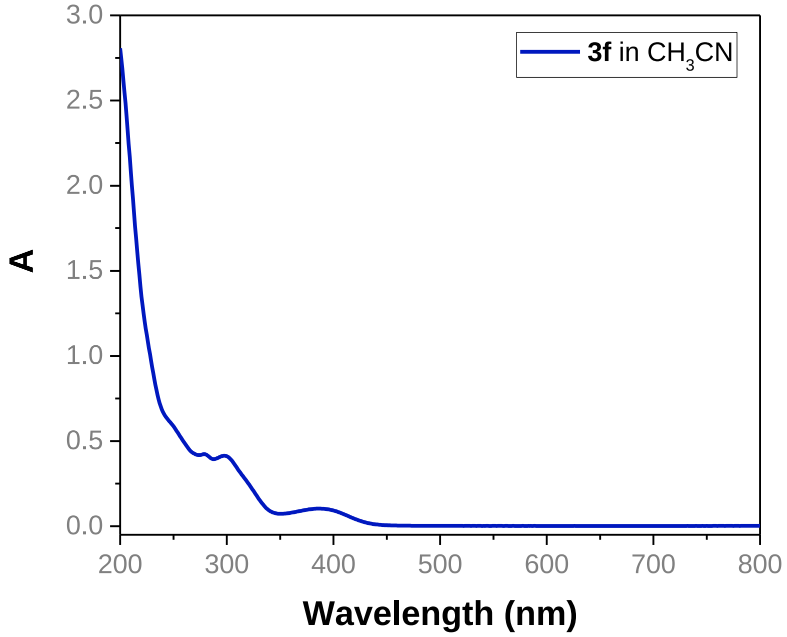

Figure S8. UV-Vis spectrum of **3f** in CH_3CN at 25 ºC, the concentration was ca. $3{\times}10^{-5}$ M

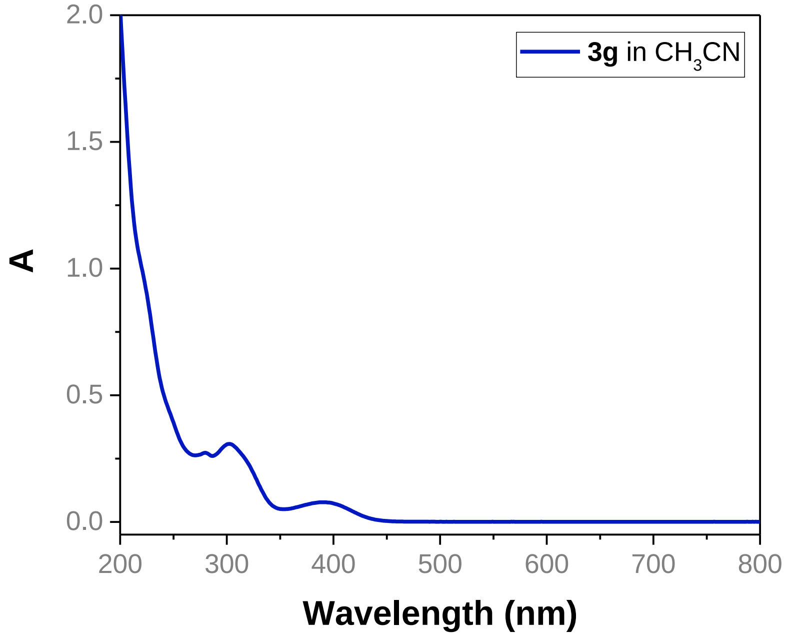

Figure S9. UV-Vis spectrum of **3g** in CH_3CN at 25 ºC, the concentration was ca. 3×10^{-5} M

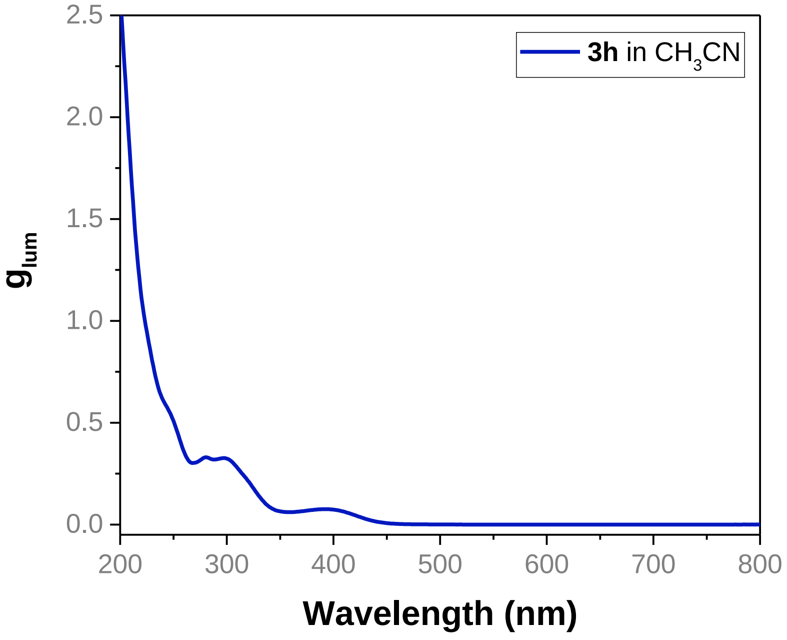

Figure S10. UV-Vis spectrum of **3h** in CH_3CN at 25 ºC, the concentration was ca. 3×10^{-5} M

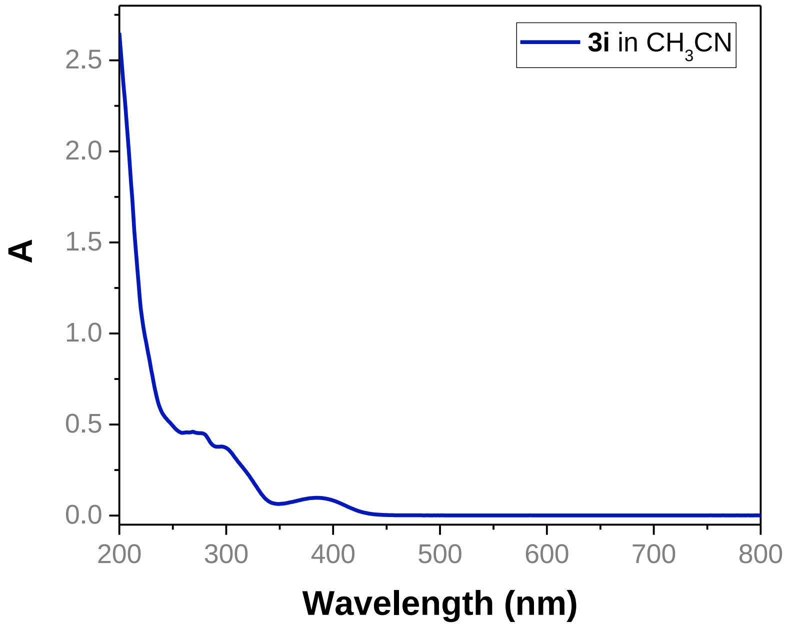

Figure S11. UV-Vis spectrum of **3i** in CH_3CN at 25 °C, the concentration was ca. 3×10^{-5} M

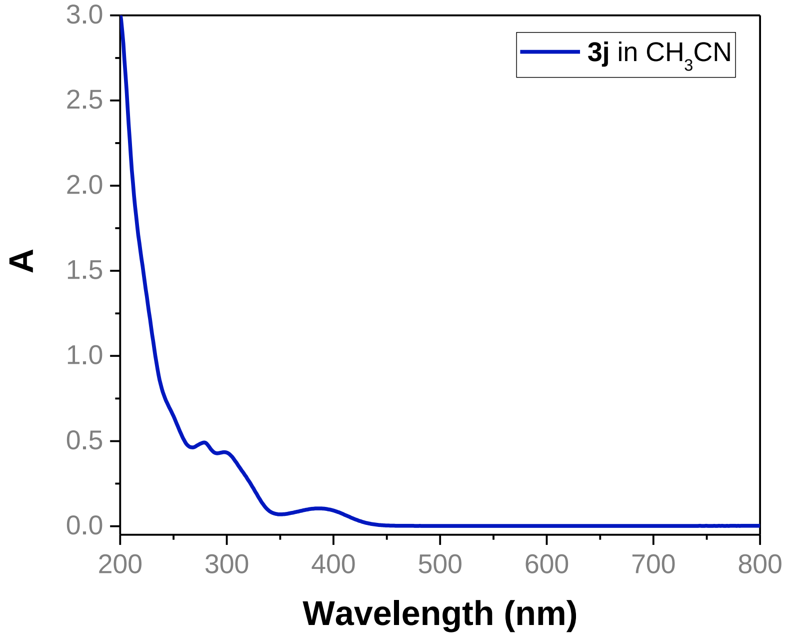

Figure S12. UV-Vis spectrum of **3j** in CH_3CN at 25 °C, the concentration was ca. 3×10^{-5} M

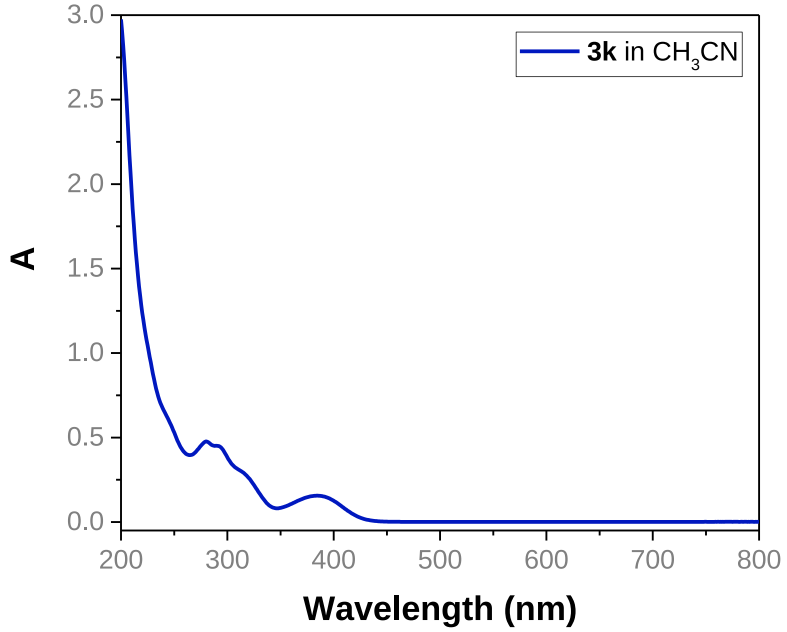

Figure S13. UV-Vis spectrum of **3k** in CH_3CN at 25 °C, the concentration was ca. 3×10^{-5} M

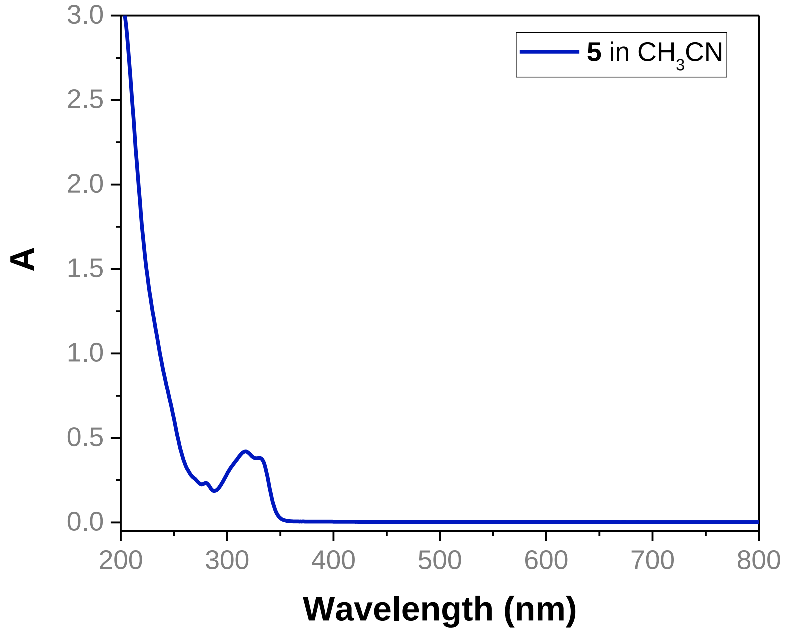

Figure S14. UV-Vis spectrum of **5** in CH_3CN at 25 °C, the concentration was ca. 3×10^{-5} M

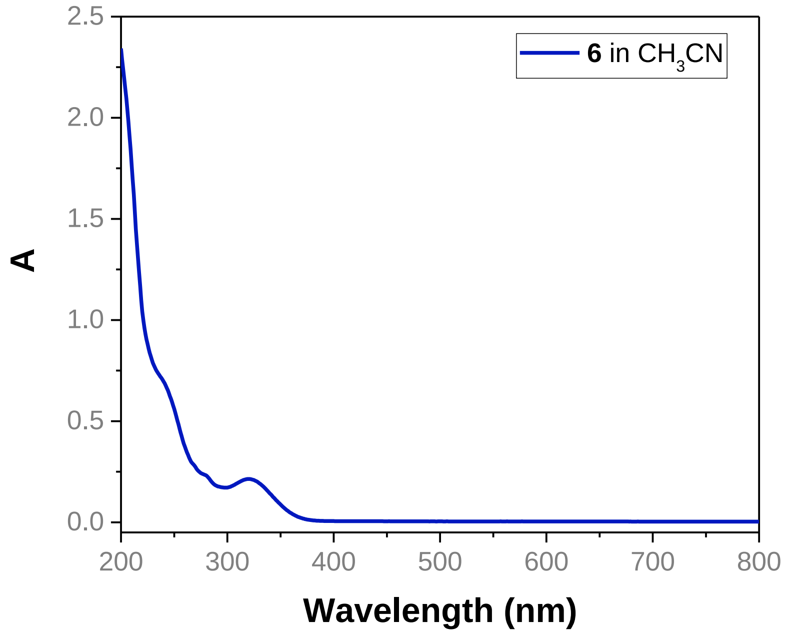

Figure S15. UV-Vis spectrum of **6** in CH_3CN at 25 ºC, the concentration was ca. 3×10^{-5} M

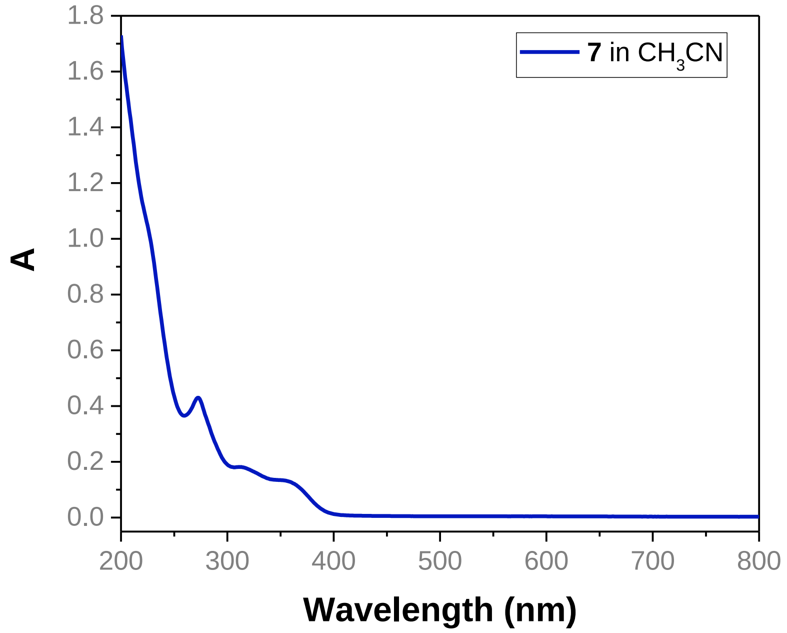

Figure S16. UV-Vis spectrum of **7** in CH_3CN at 25 ºC, the concentration was ca. 3×10^{-5} M

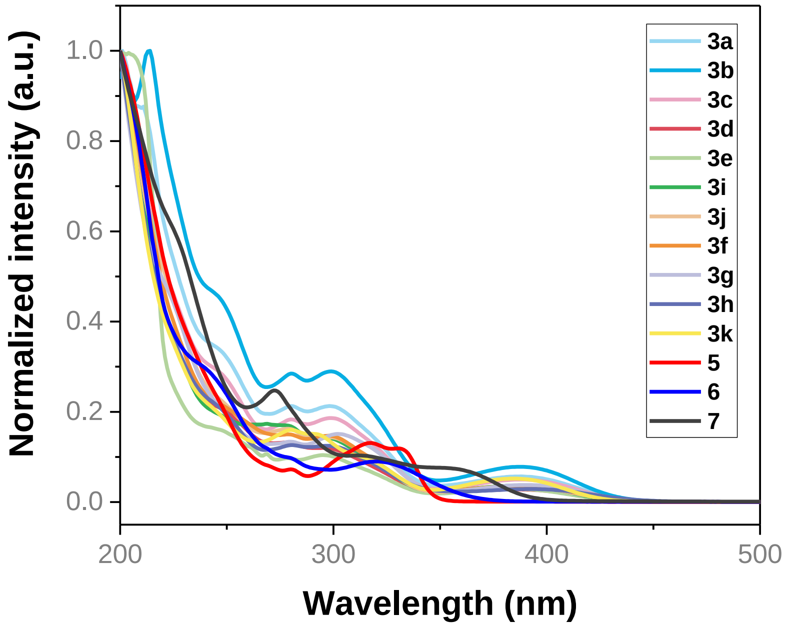

Figure S17. Normalized UV-Vis spectra of **3a-3k**, **5**, **6**, and **7** in CH_3CN at 25 ºC

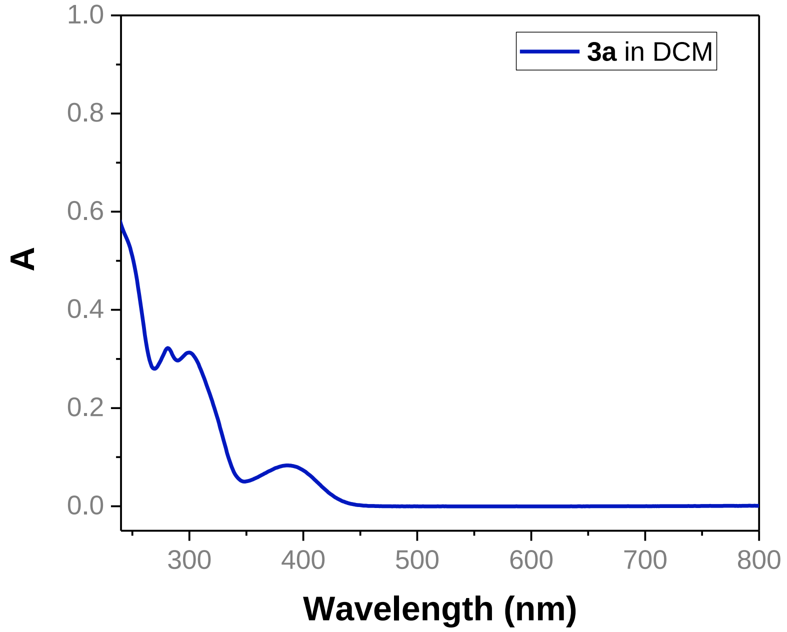

Figure S18. UV-Vis spectrum of **3a** in DCM at 25 ºC, the concentration was ca. 3×10^{-5} M

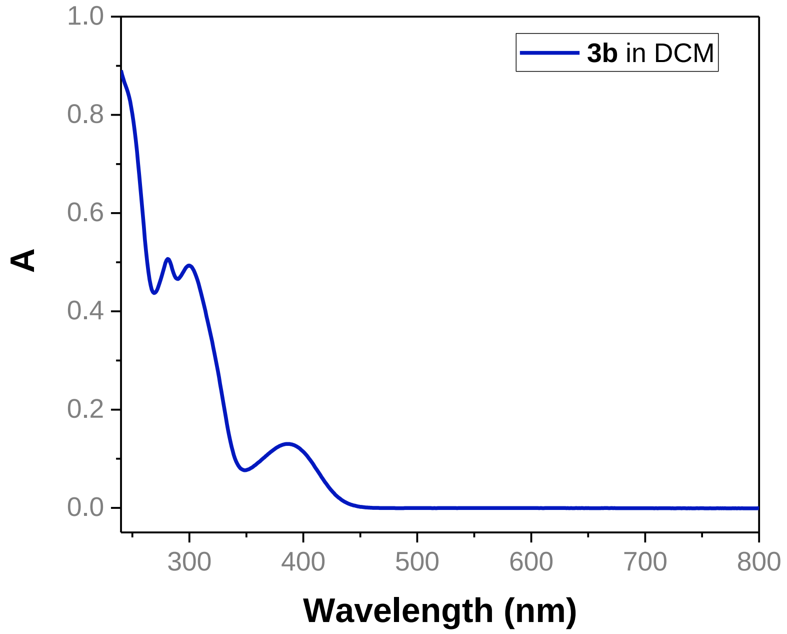

Figure S19. UV-Vis spectrum of **3b** in DCM at 25 ºC, the concentration was ca. 3×10^{-5} M

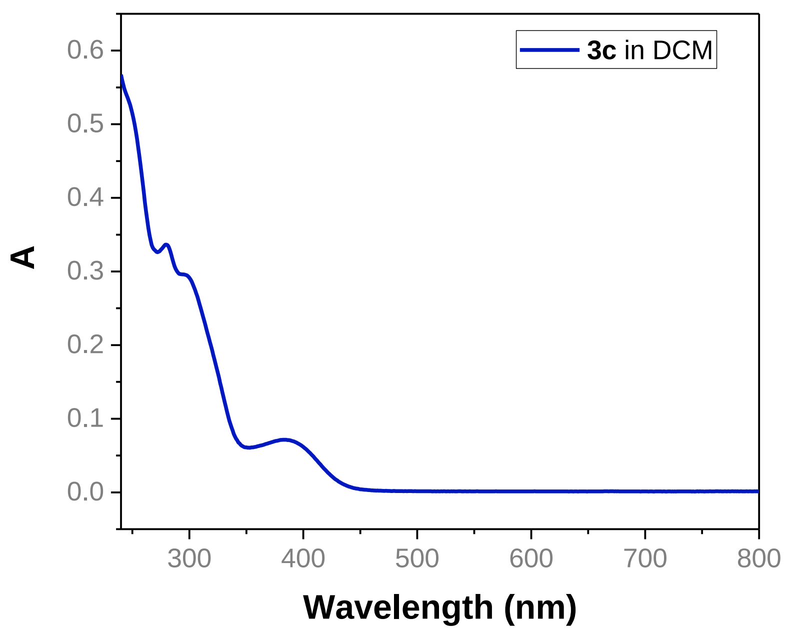

Figure S20. UV-Vis spectrum of **3c** in DCM at 25 ºC, the concentration was ca. 3×10^{-5} M

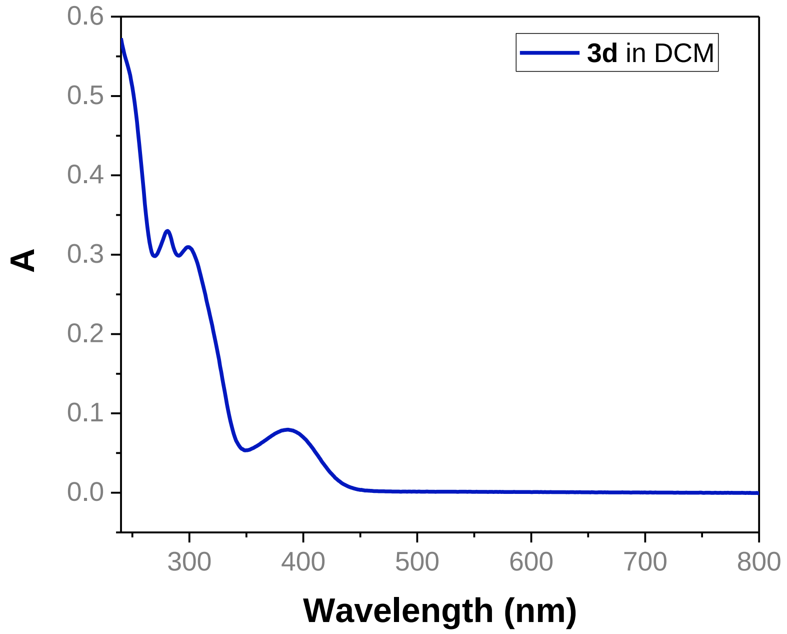

Figure S21. UV-Vis spectrum of **3d** in DCM at 25 ºC, the concentration was ca. 3×10^{-5} M

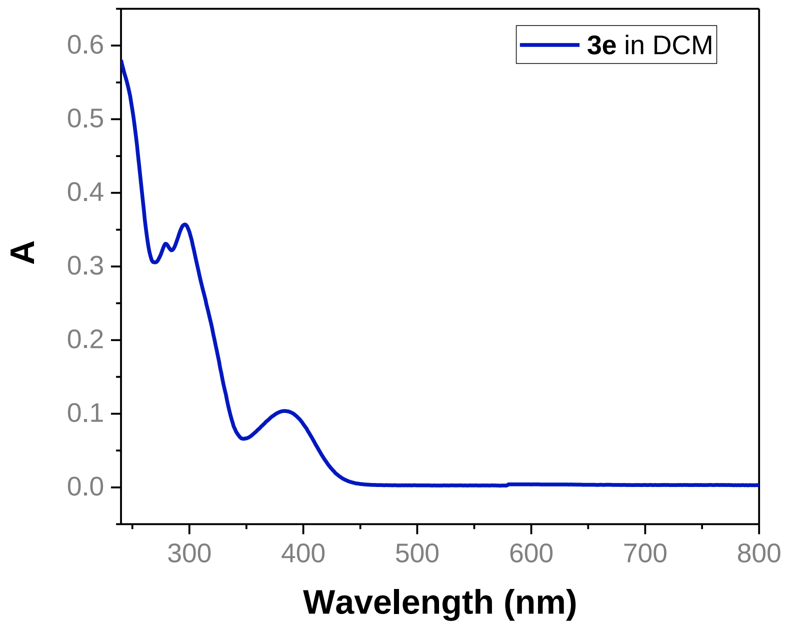

Figure S22. UV-Vis spectrum of **3e** in DCM at 25 ºC, the concentration was ca. 3×10^{-5} M

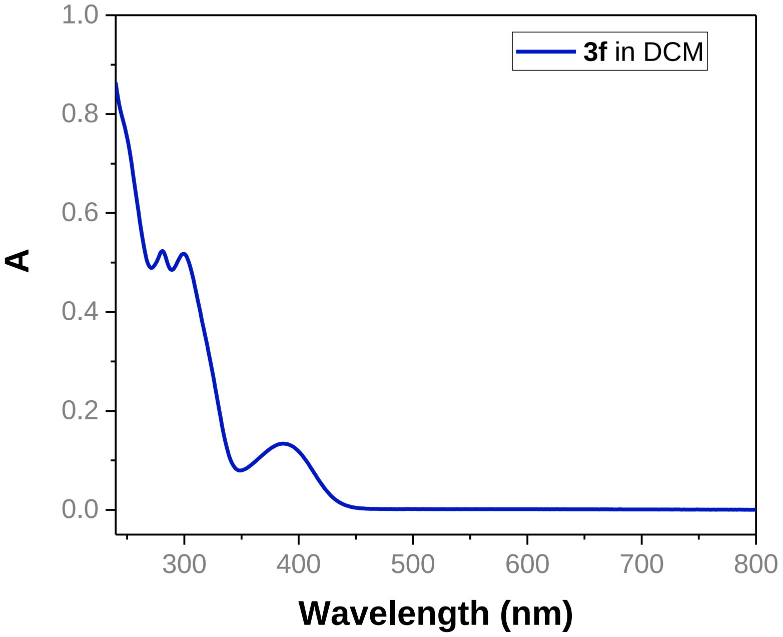

Figure S23. UV-Vis spectrum of **3f** in DCM at 25 ºC, the concentration was ca. 3×10^{-5} M

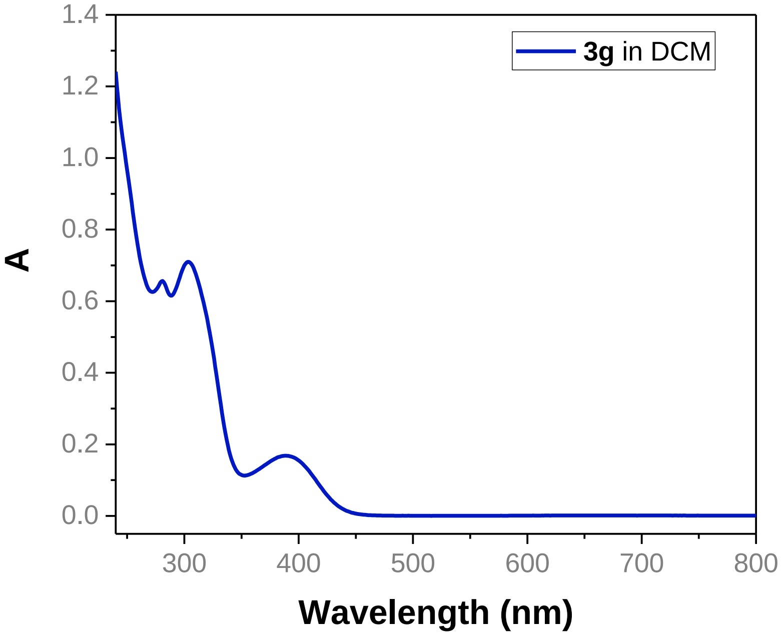

Figure S24. UV-Vis spectrum of **3g** in DCM at 25 ºC, the concentration was ca. 3×10^{-5} M

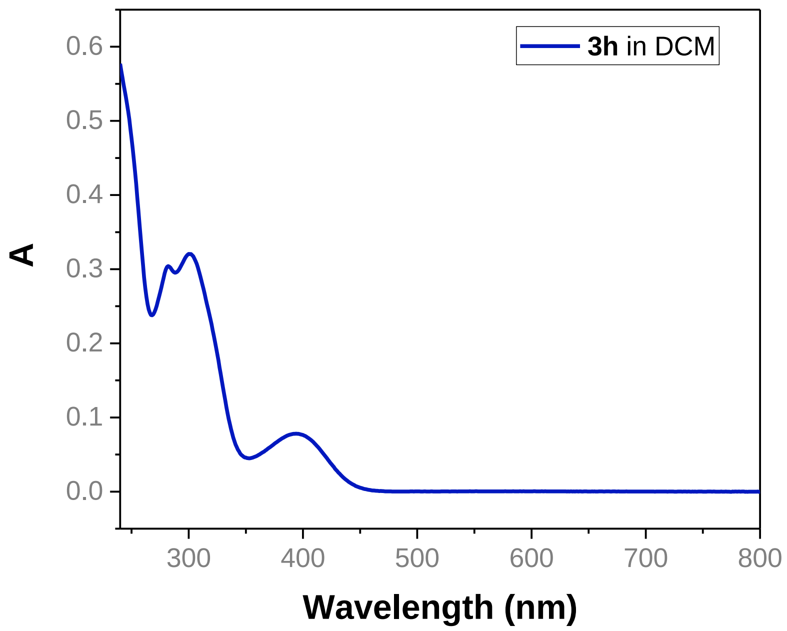

Figure S25. UV-Vis spectrum of **3h** in DCM at 25 °C, the concentration was ca. 3×10^{-5} M

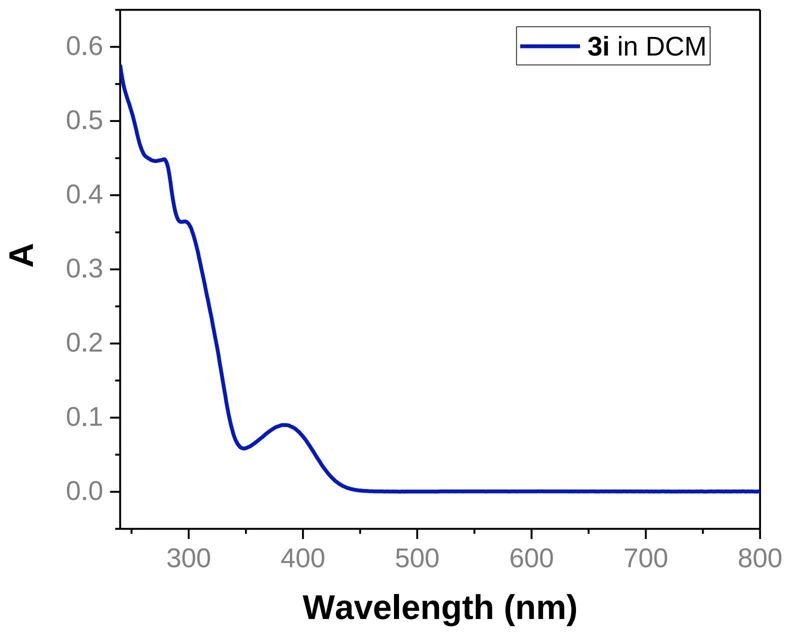

Figure S26. UV-Vis spectrum of **3i** in DCM at 25 °C, the concentration was ca. 3×10^{-5} M

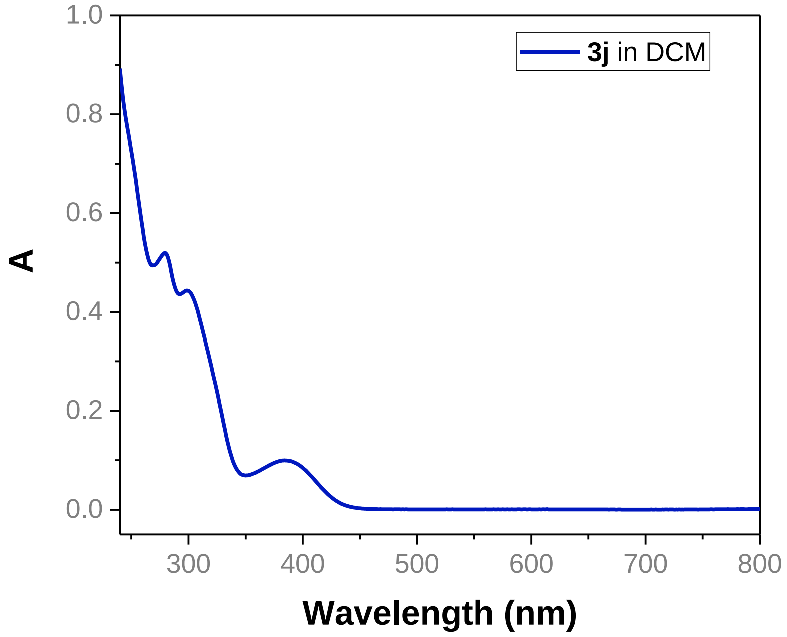

Figure S27. UV-Vis spectrum of **3j** in DCM at 25 ºC, the concentration was ca. 3×10^{-5} M

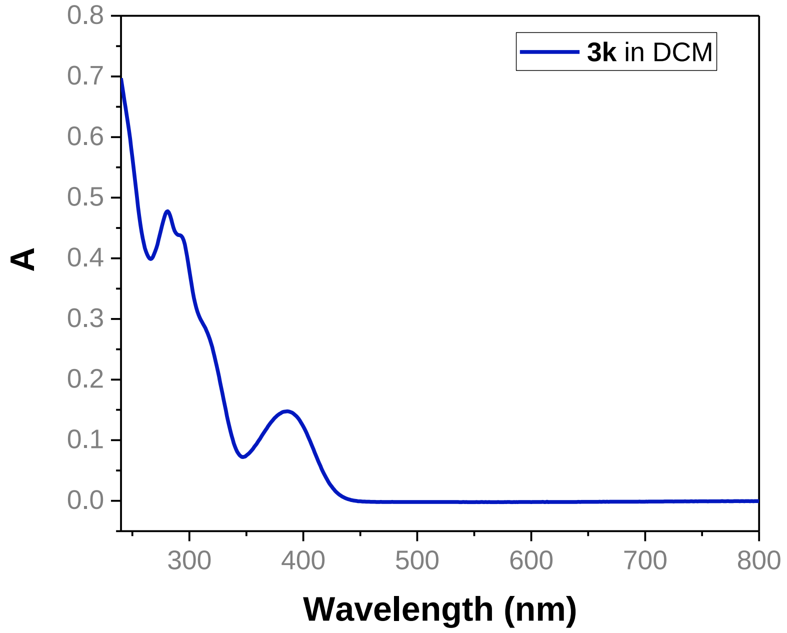

Figure S28. UV-Vis spectrum of **3k** in DCM at 25 ºC, the concentration was ca. 3×10^{-5} M

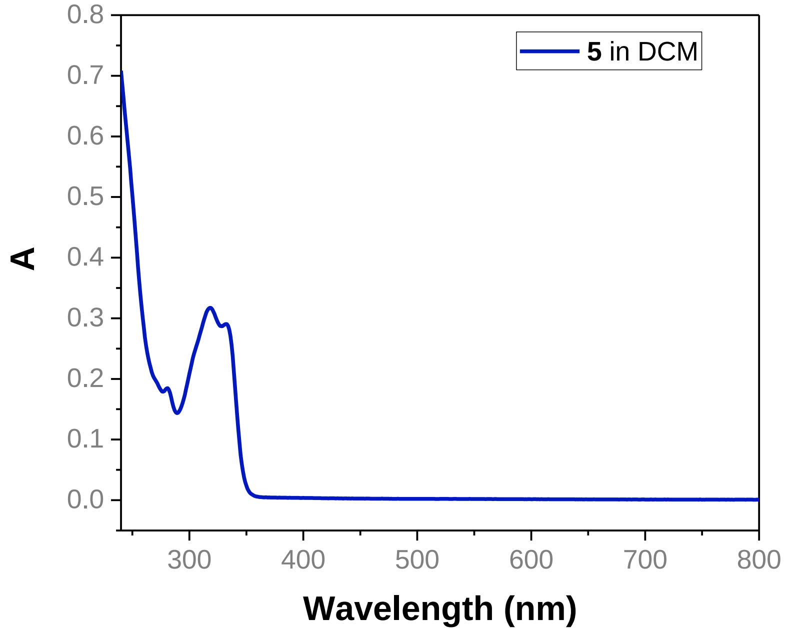

Figure S29. UV-Vis spectrum of **5** in DCM at 25 ºC, the concentration was ca. 3×10^{-5} M

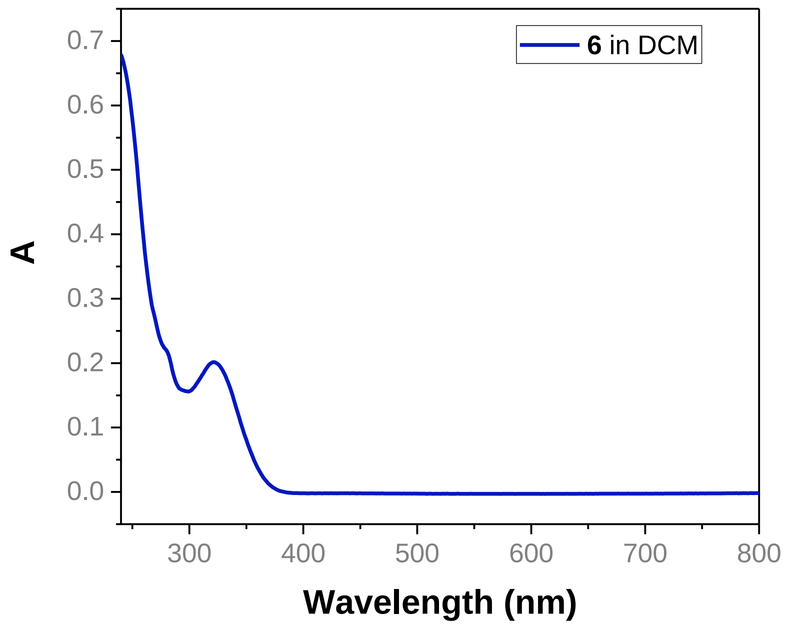

Figure S30. UV-Vis spectrum of **6** in DCM at 25 ºC, the concentration was ca. 3×10^{-5} M

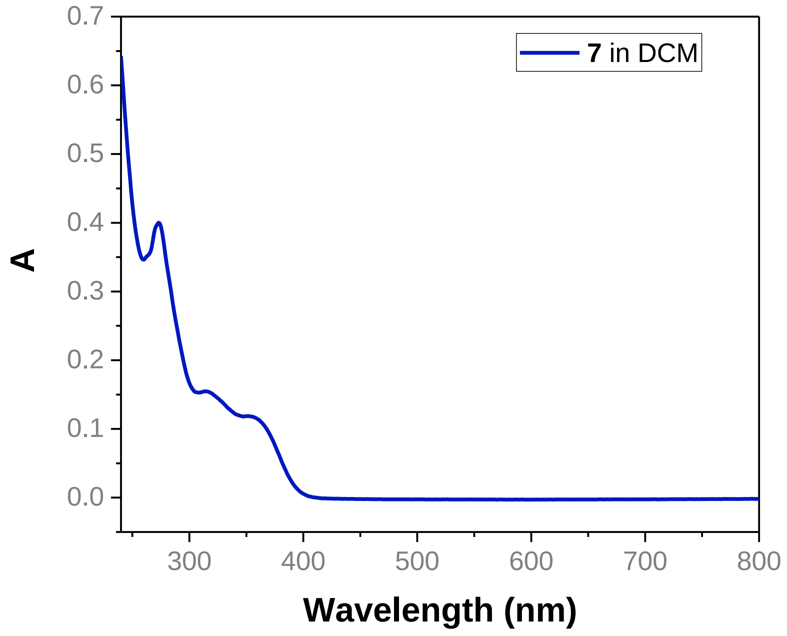

Figure S31. UV-Vis spectrum of **7** in DCM at 25 ºC, the concentration was ca. 3×10^{-5} M

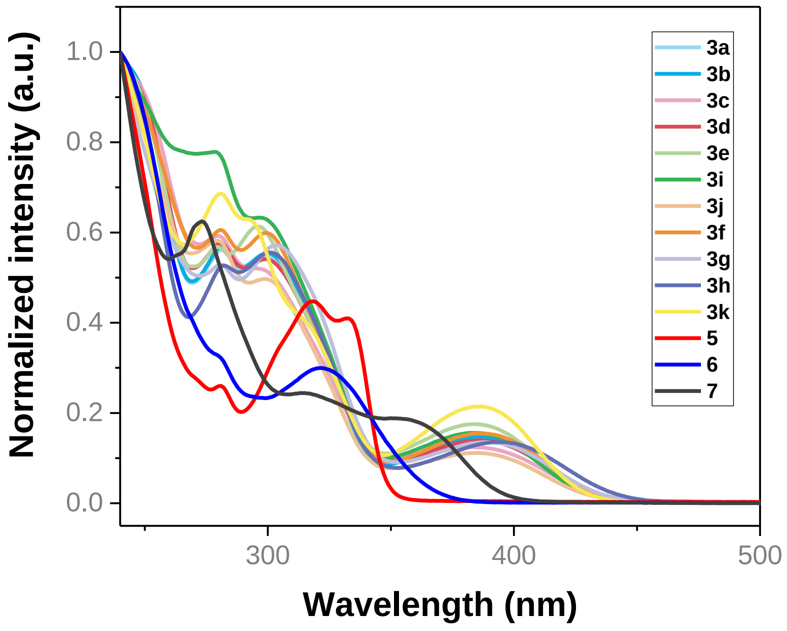

Figure S32. Normalized UV-Vis spectra of **3a-3k**, **5**, **6**, and **7** in DCM at 25 ºC

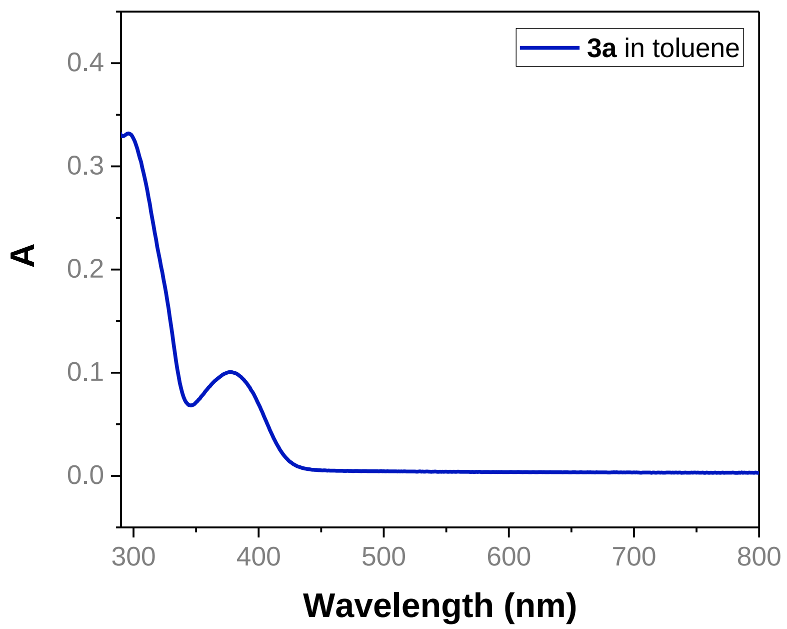

Figure S33. UV-Vis spectrum of **3a** in toluene at 25 °C, the concentration was ca. 3×10^{-5} M

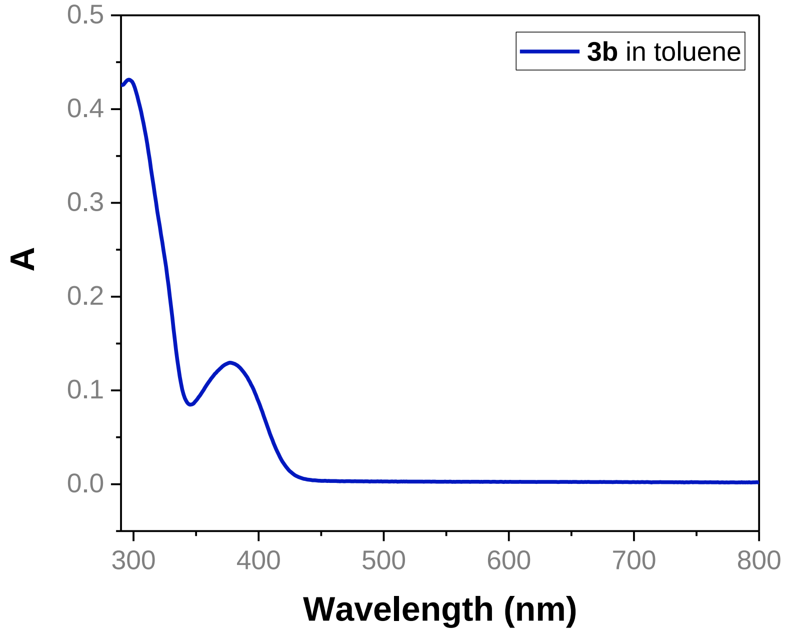

Figure S34. UV-Vis spectrum of **3b** in toluene at 25 °C, the concentration was ca. 3×10^{-5} M

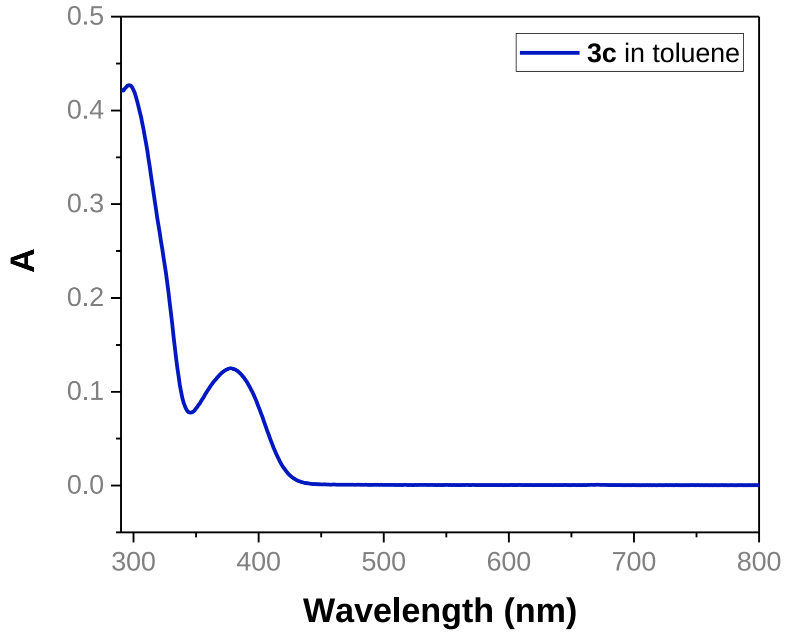

Figure S35. UV-Vis spectrum of **3c** in toluene at 25 ^{o}C, the concentration was ca. $3{\times}10^{-5}$ M

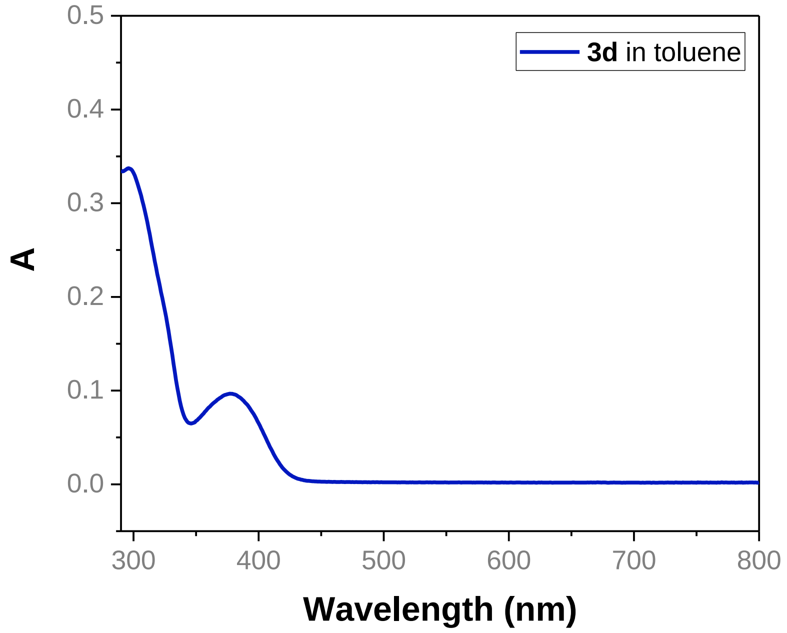

Figure S36. UV-Vis spectrum of **3d** in toluene at 25 ^{o}C, the concentration was ca. $3{\times}10^{-5}$ M

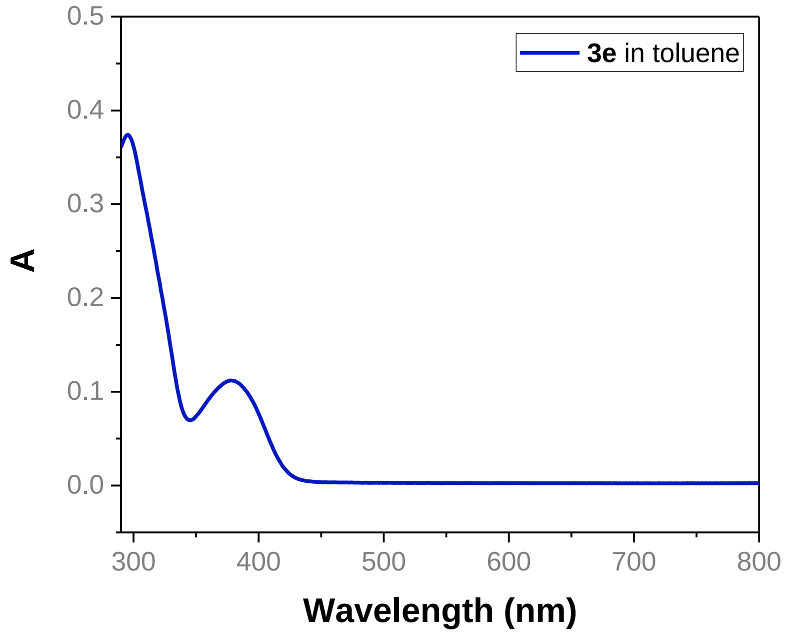

Figure S37. UV-Vis spectrum of **3e** in toluene at 25 ^{o}C, the concentration was ca. 3×10^{-5} M

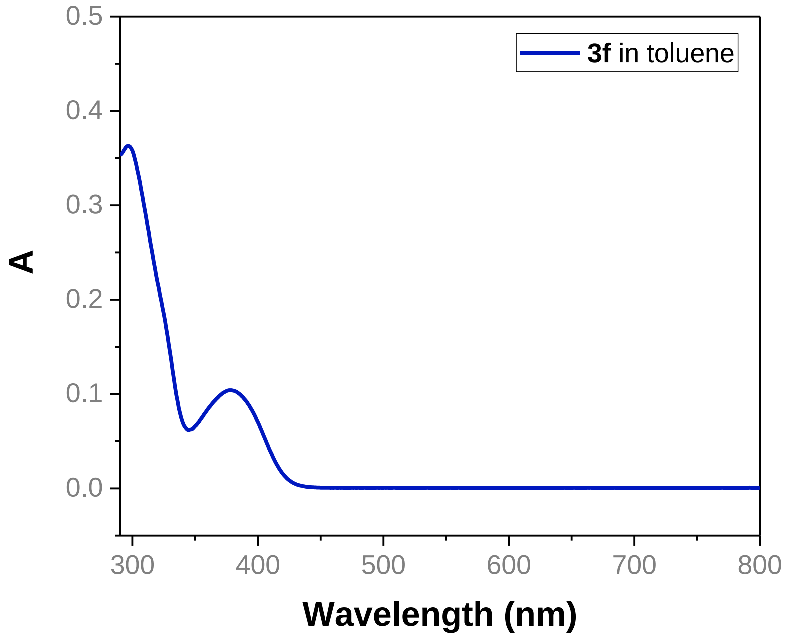

Figure S38. UV-Vis spectrum of **3f** in toluene at 25 ^{o}C, the concentration was ca. 3×10^{-5} M

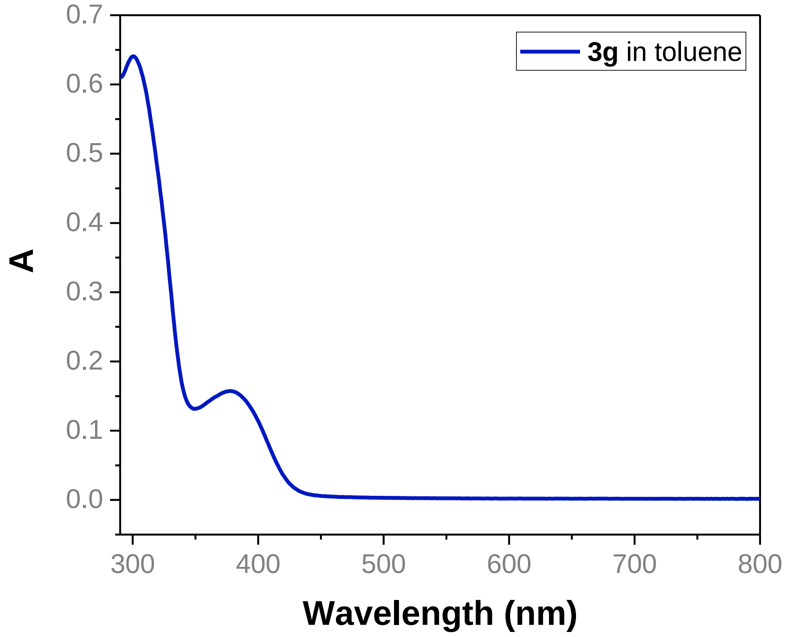

Figure S39. UV-Vis spectrum of **3g** in toluene at 25 ^{o}C, the concentration was ca. 3×10^{-5} M

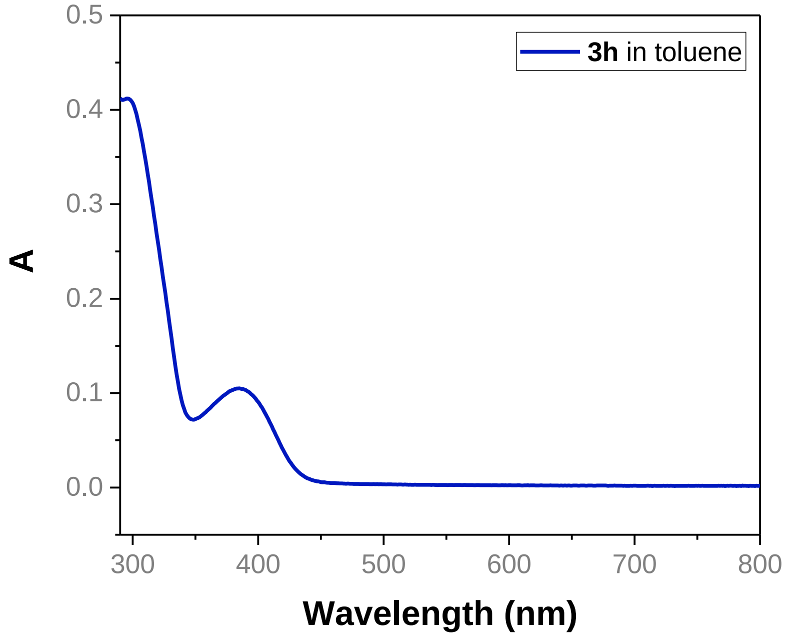

Figure S40. UV-Vis spectrum of **3h** in toluene at 25 ^{o}C, the concentration was ca. 3×10^{-5} M

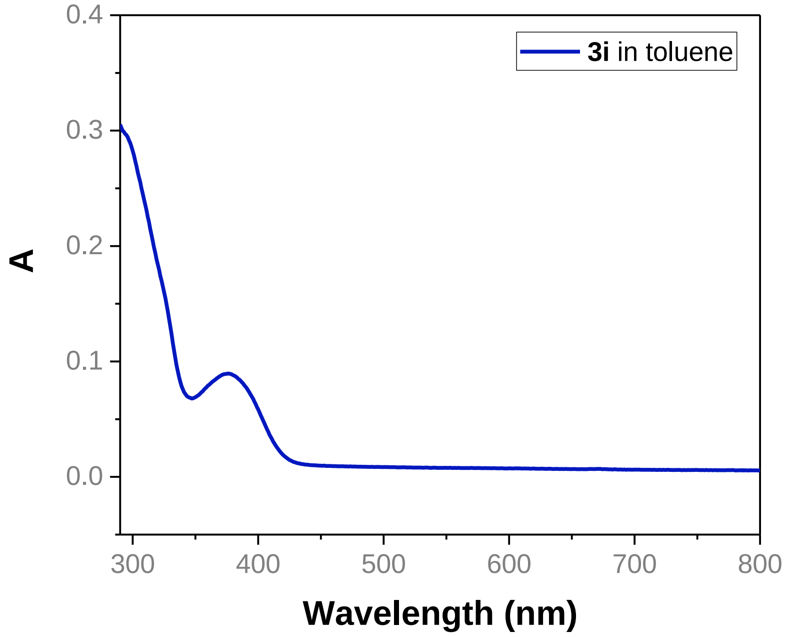

Figure S41. UV-Vis spectrum of **3i** in toluene at 25 °C, the concentration was ca. 3×10^{-5} M

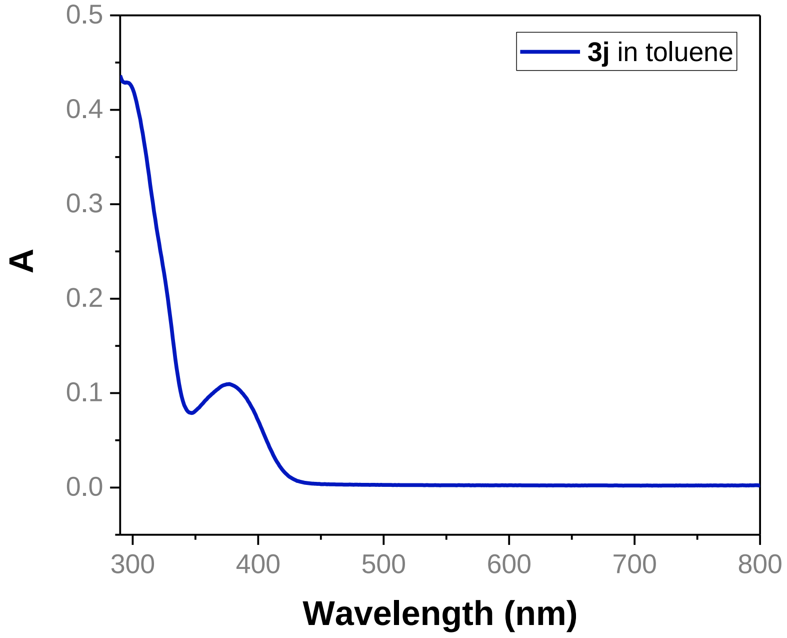

Figure S42. UV-Vis spectrum of **3j** in toluene at 25 °C, the concentration was ca. 3×10^{-5} M

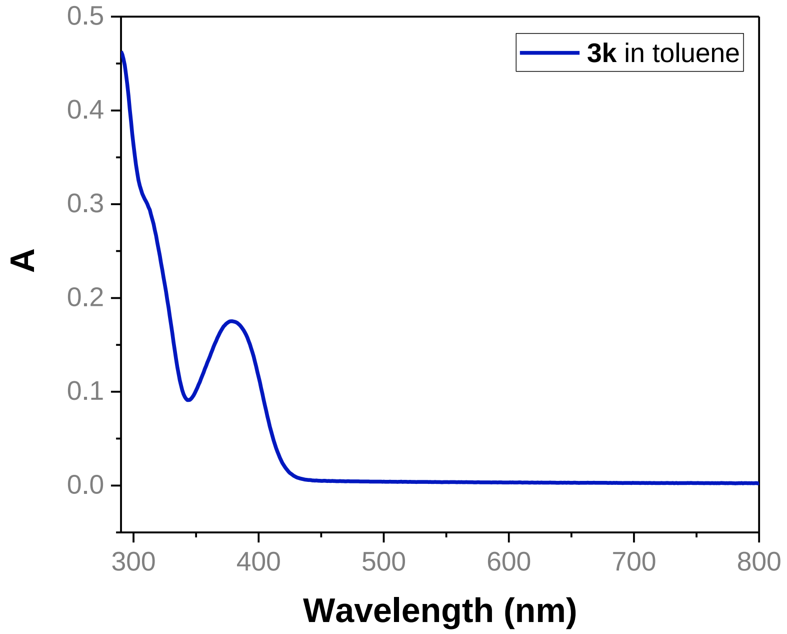

Figure S43. UV-Vis spectrum of **3k** in toluene at 25 °C, the concentration was ca. 3×10^{-5} M

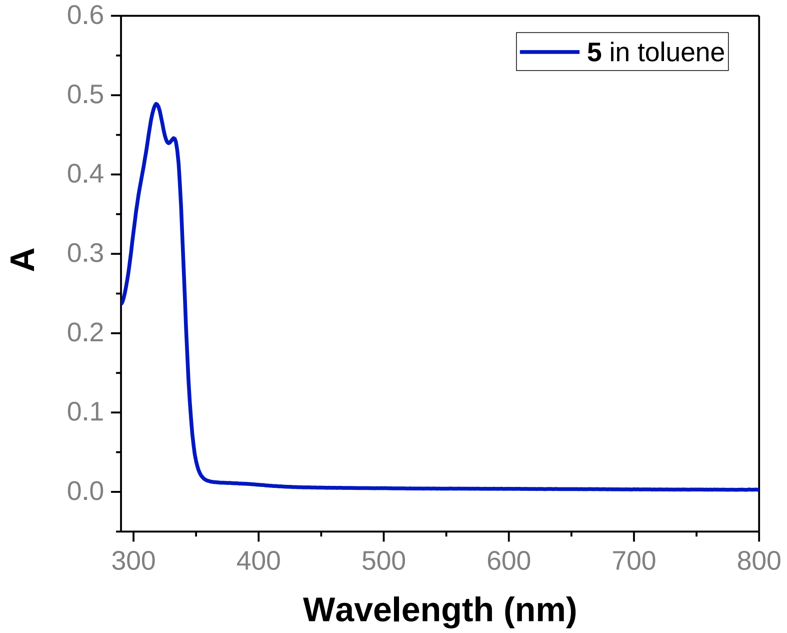

Figure S44. UV-Vis spectrum of **5** in toluene at 25 °C, the concentration was ca. 3×10^{-5} M

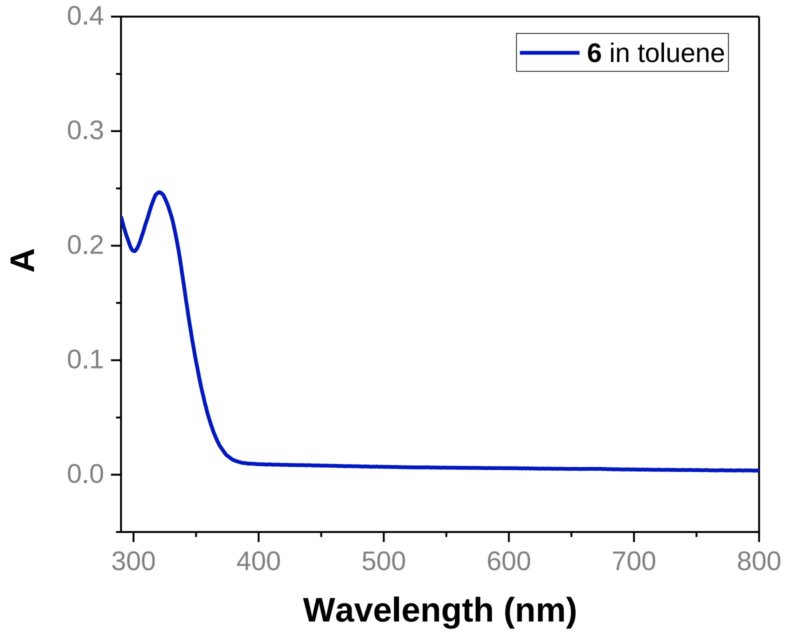

Figure S45. UV-Vis spectrum of **6** in toluene at 25 °C, the concentration was ca. 3×10^{-5} M

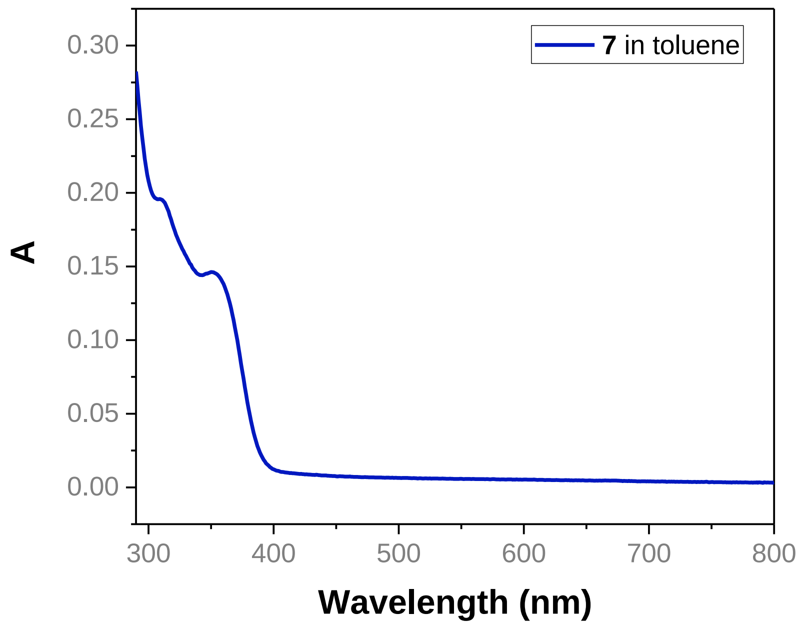

Figure S46. UV-Vis spectrum of **7** in toluene at 25 °C, the concentration was ca. 3×10^{-5} M

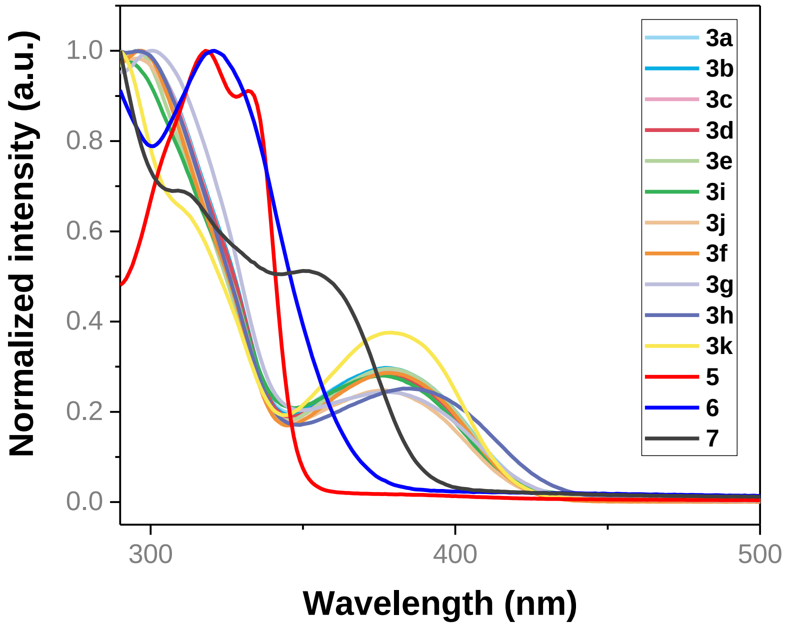

Figure S47. Normalized UV-Vis spectra of **3a-3k**, **5**, **6**, and **7** in toluene at 25 ^{o}C

16. Copies of fluorescence spectra of 3a-3k, 5, 6, and 7

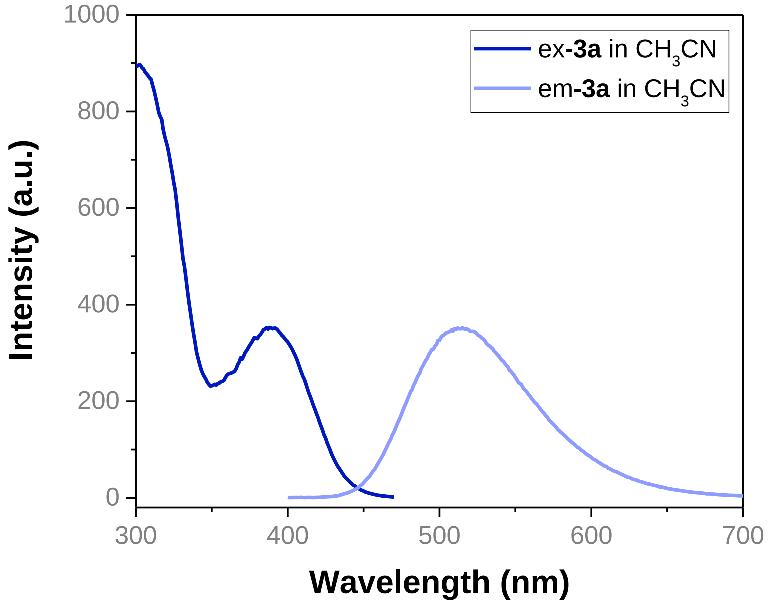

Figure S48. Fluorescence spectrum of **3a** in CH_3CN at 25 °C, the concentration was ca. 2×10^{-5} M

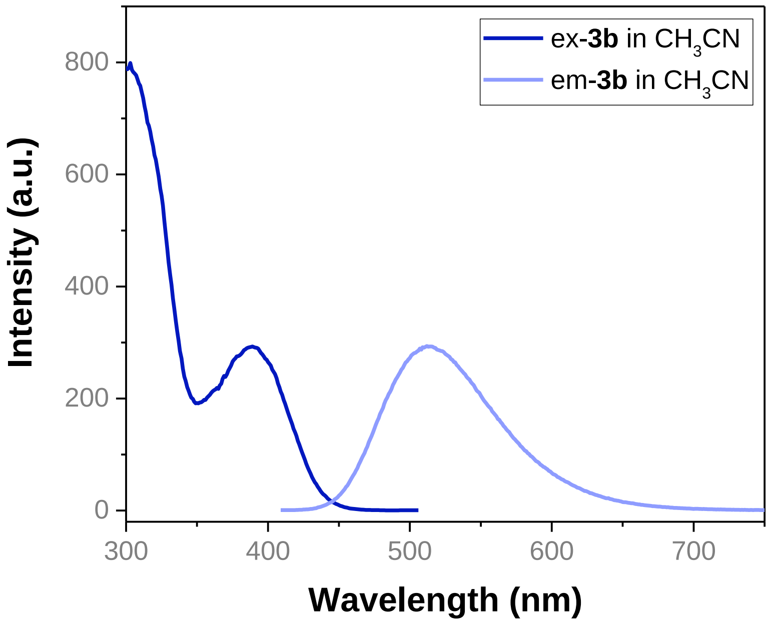

Figure S49. Fluorescence spectrum of **3b** in CH_3CN at 25 °C, the concentration was ca. 2×10^{-5} M

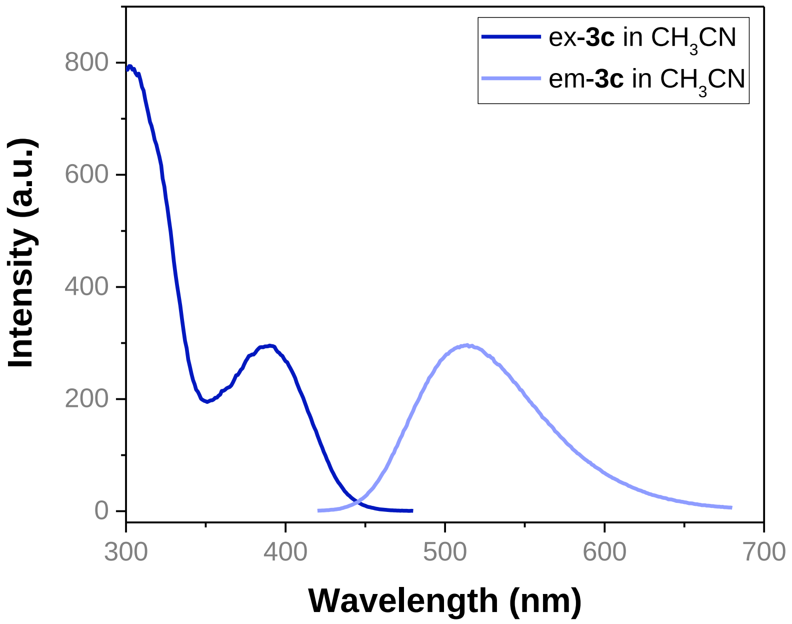

Figure S50. Fluorescence spectrum of **3c** in CH_3CN at 25 ^oC, the concentration was ca. 2×10^{-5} M

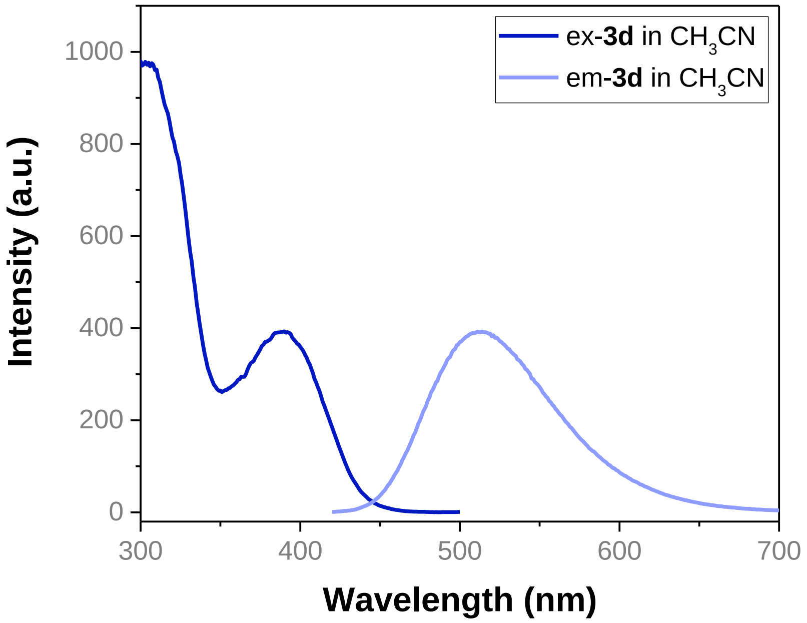

Figure S51. Fluorescence spectrum of **3d** in CH_3CN at 25 ^oC, the concentration was ca. 2×10^{-5} M

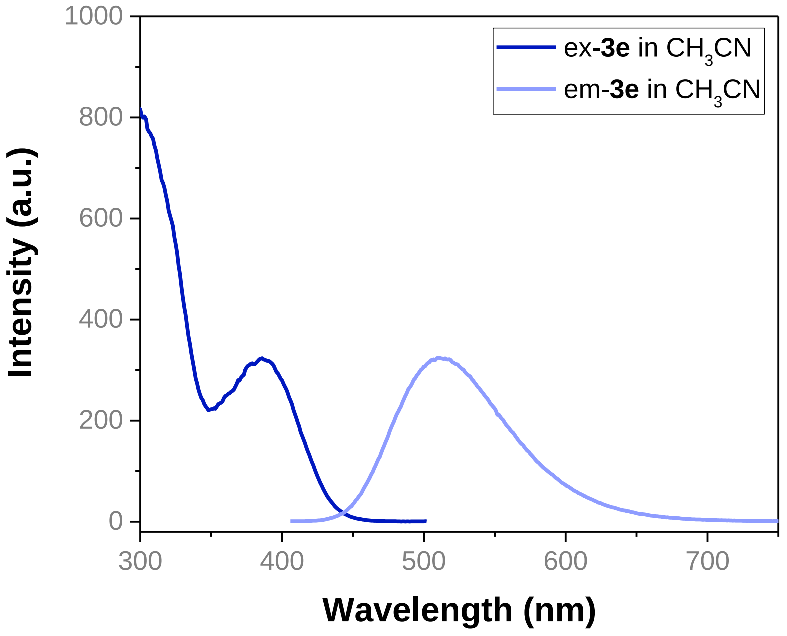

Figure S52. Fluorescence spectrum of **3e** in CH_3CN at 25 ^oC, the concentration was ca. 2×10^{-5} M

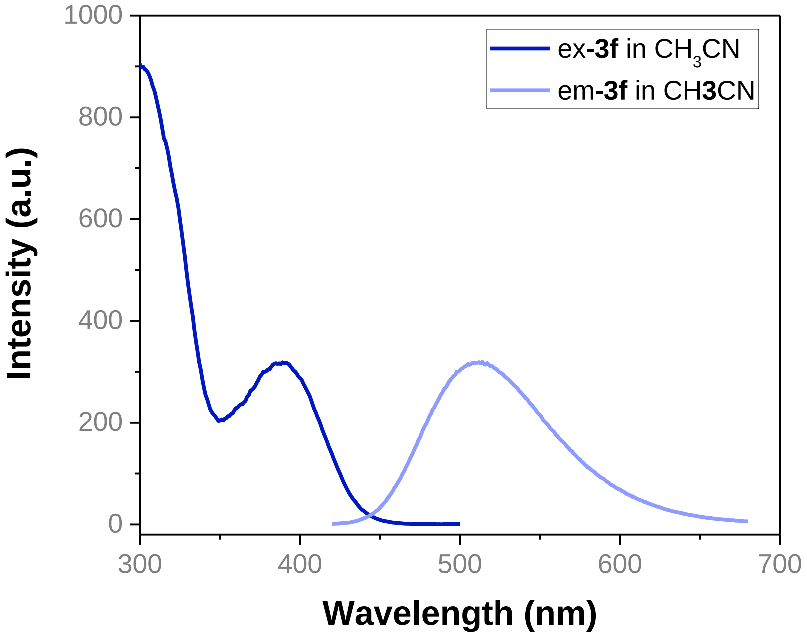

Figure S53. Fluorescence spectrum of **3f** in CH_3CN at 25 ^oC, the concentration was ca. 2×10^{-5} M

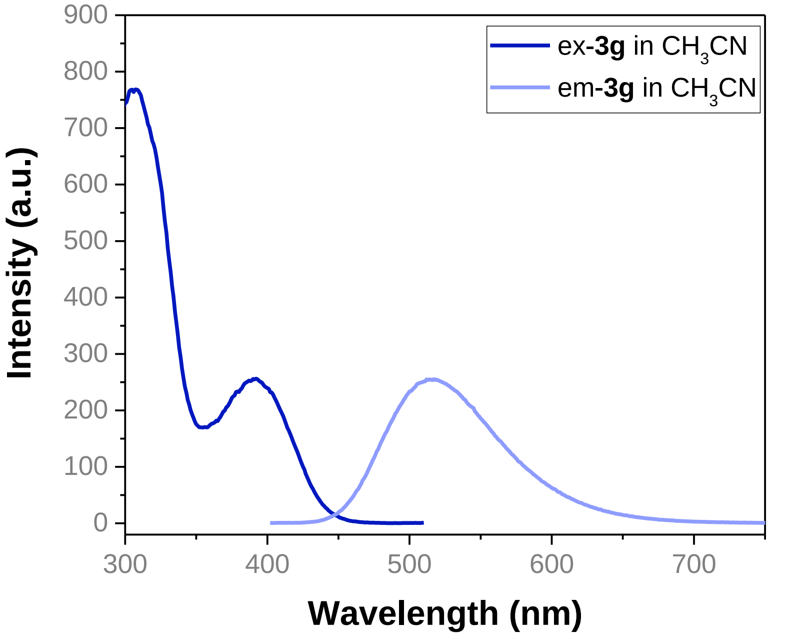

Figure S54. Fluorescence spectrum of **3g** in CH_3CN at 25 ^{o}C, the concentration was ca. 2×10^{-5} M

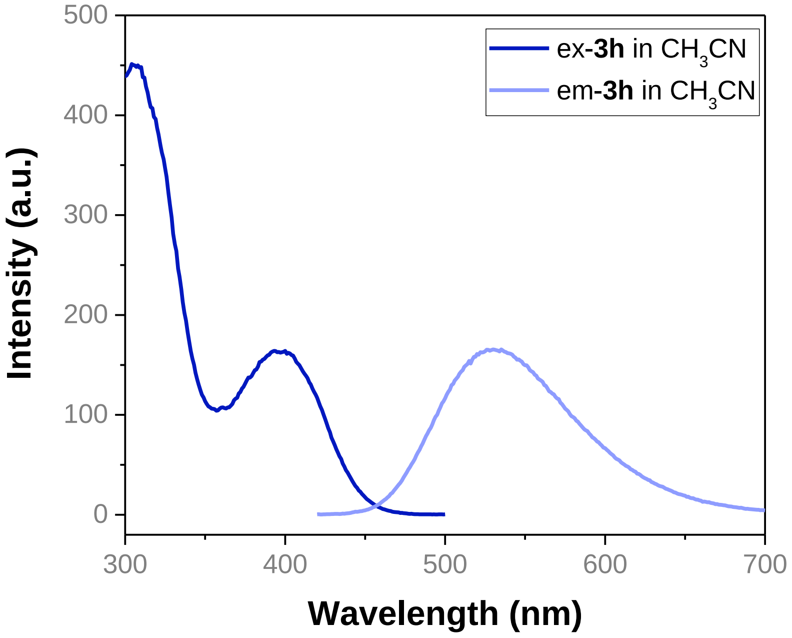

Figure S55. Fluorescence spectrum of **3h** in CH_3CN at 25 ^{o}C, the concentration was ca. 2×10^{-5} M

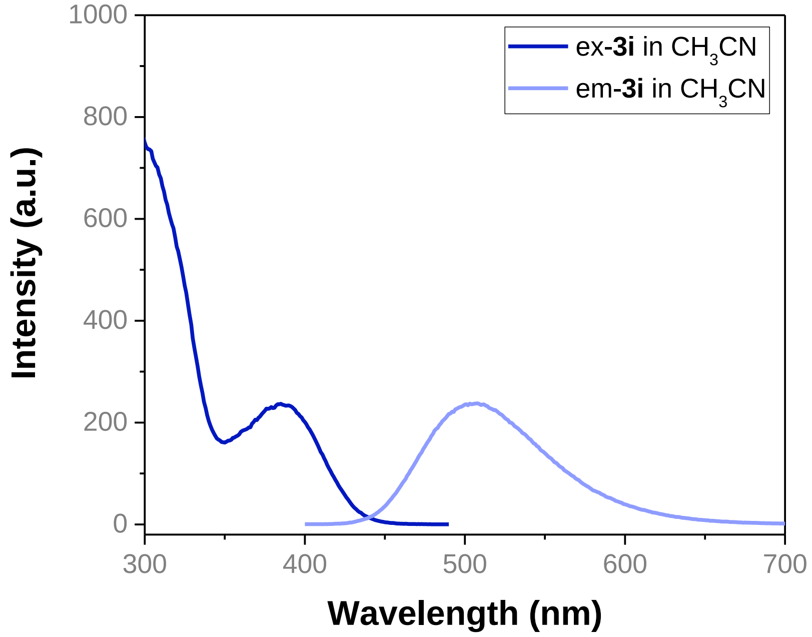

Figure S56. Fluorescence spectrum of **3i** in CH_3CN at 25 °C, the concentration was ca. 2×10^{-5} M

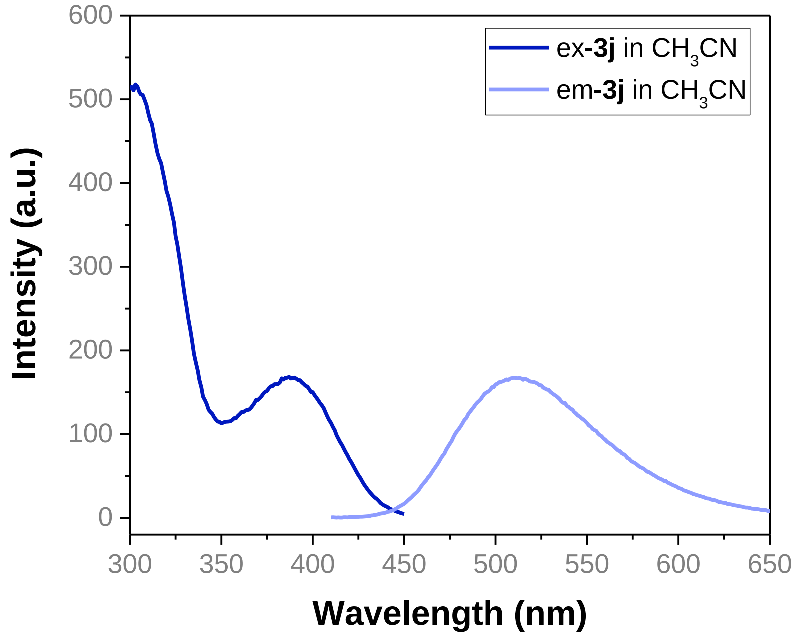

Figure S57. Fluorescence spectrum of **3j** in CH_3CN at 25 °C, the concentration was ca. 2×10^{-5} M

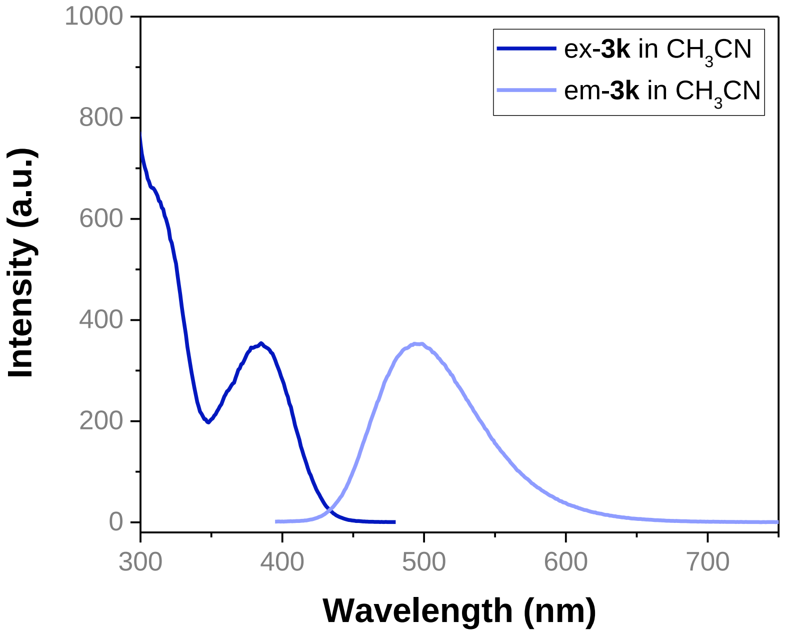

Figure S58. Fluorescence spectrum of **3k** in CH_3CN at 25 °C, the concentration was ca. 2×10^{-5} M

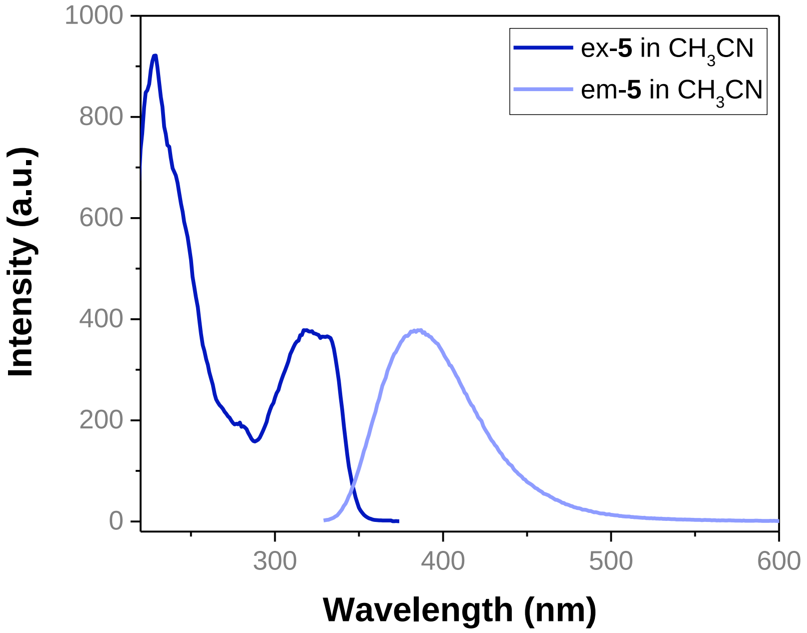

Figure S59. Fluorescence spectrum of **5** in CH_3CN at 25 °C, the concentration was ca. 2×10^{-5} M

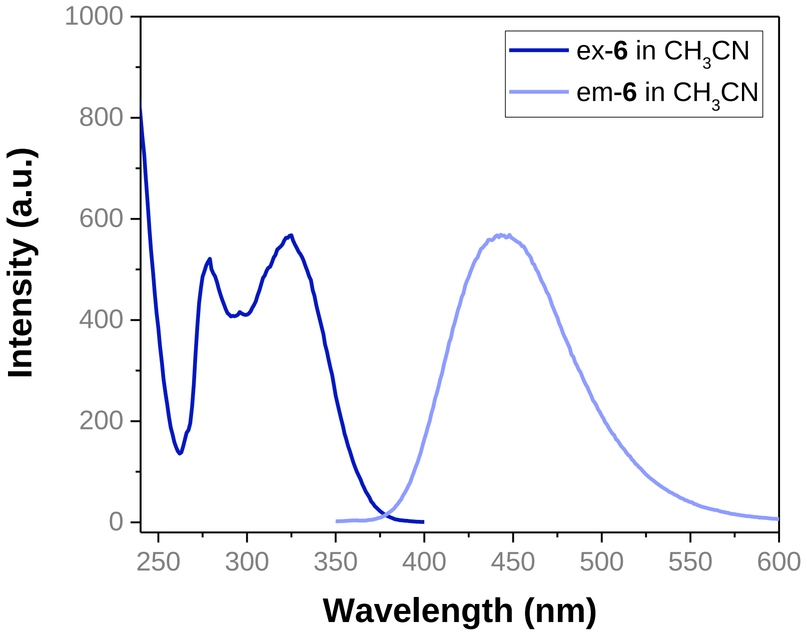

Figure S60. Fluorescence spectrum of **6** in CH_3CN at 25 °C, the concentration was ca. 2×10^{-5} M

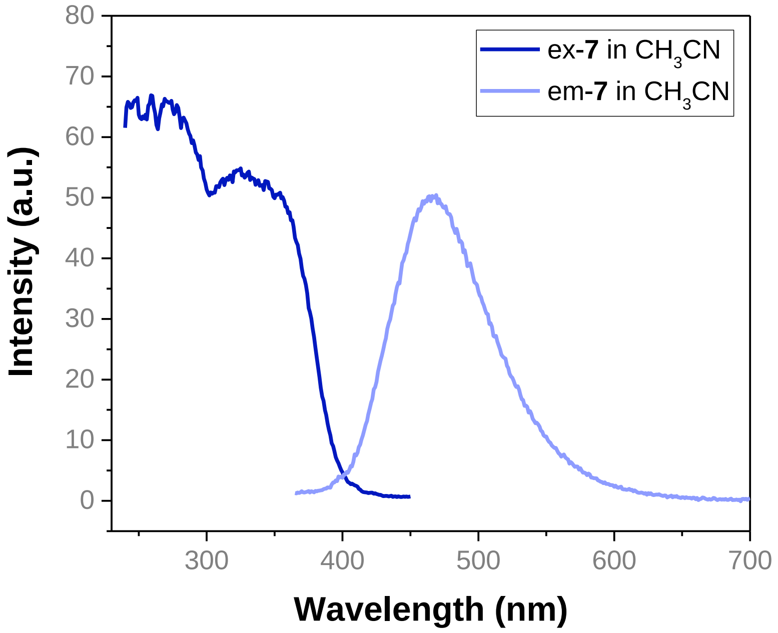

Figure S61. Fluorescence spectrum of **7** in CH_3CN at 25 °C, the concentration was ca. 2×10^{-5} M

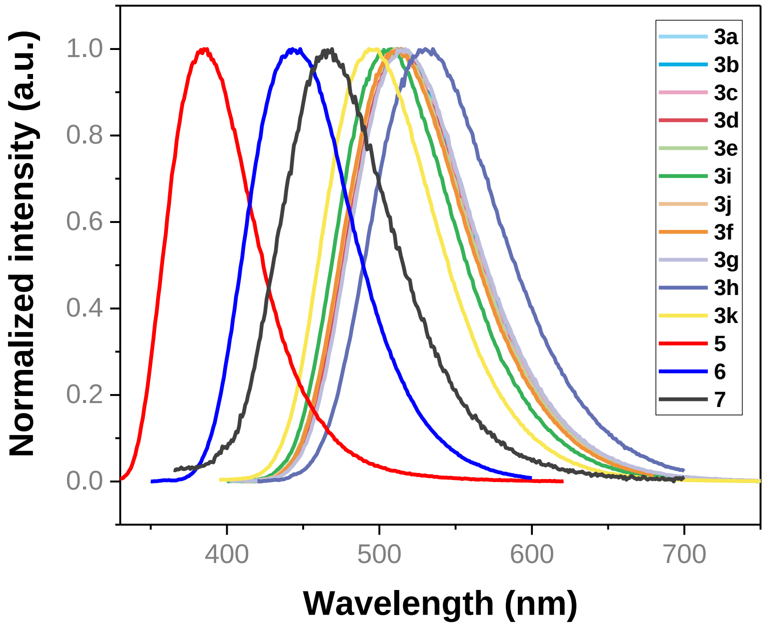

Figure S62. Normalized Fluorescence spectra of **3a**-**3k**, **5**, **6**, and **7** in CH_3CN at 25 °C.

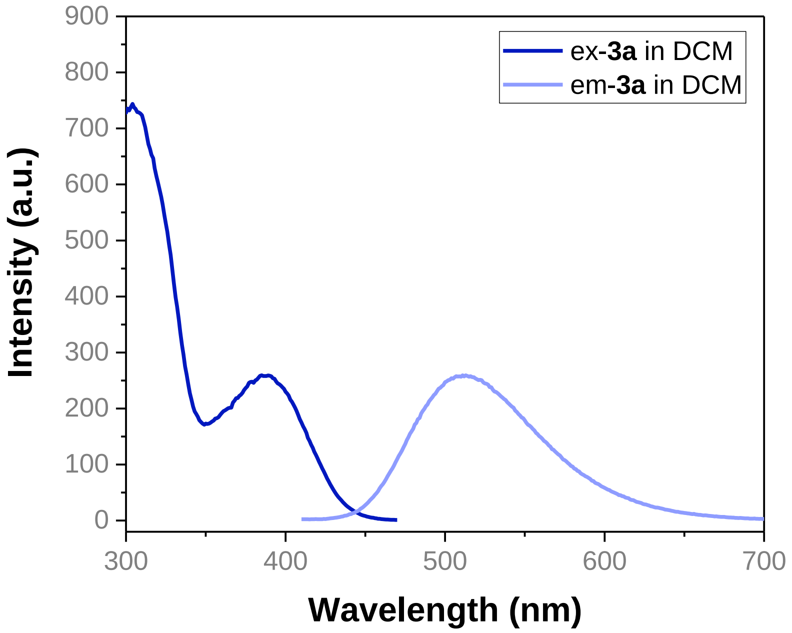

Figure S63. Fluorescence spectrum of **3a** in DCM at 25 °C, the concentration was ca. 2×10^{-5} M

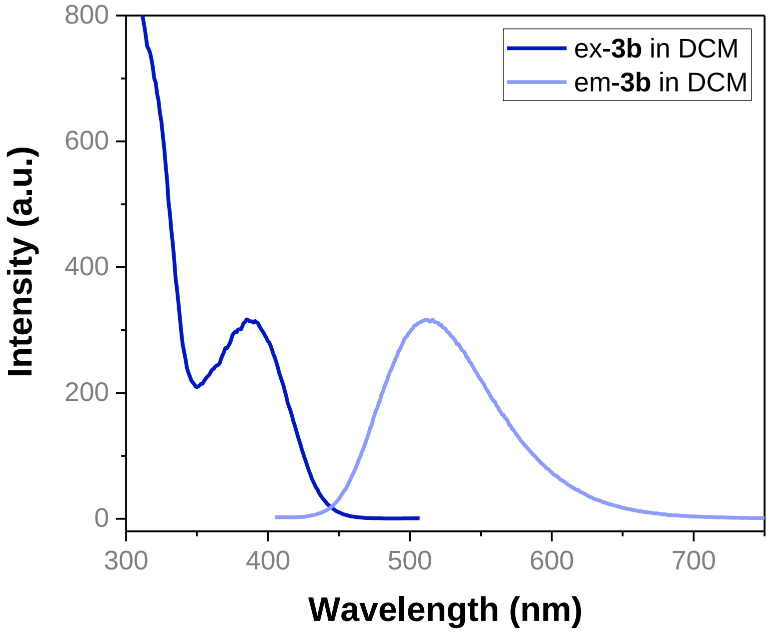

Figure S64. Fluorescence spectrum of **3b** in DCM at 25 ºC, the concentration was ca. 2×10^{-5} M

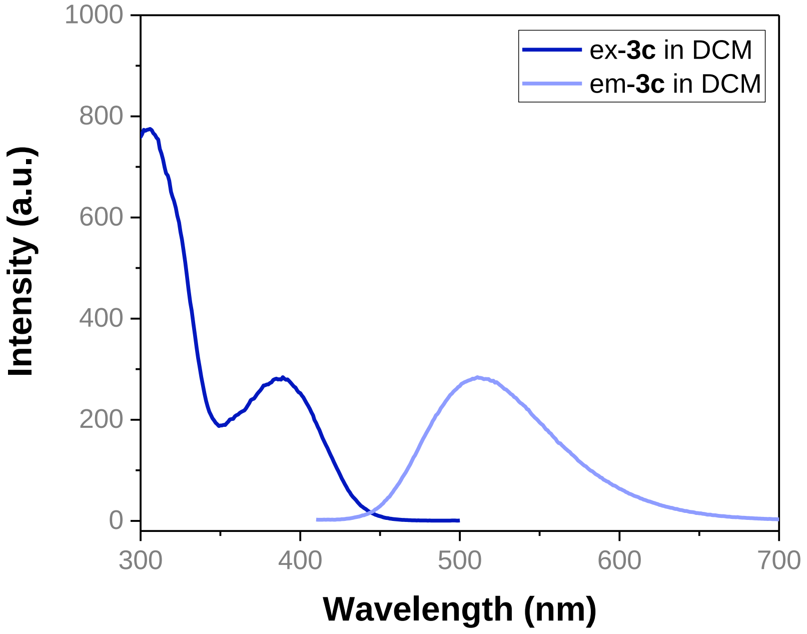

Figure S65. Fluorescence spectrum of **3c** in DCM at 25 ºC, the concentration was ca. 2×10^{-5} M

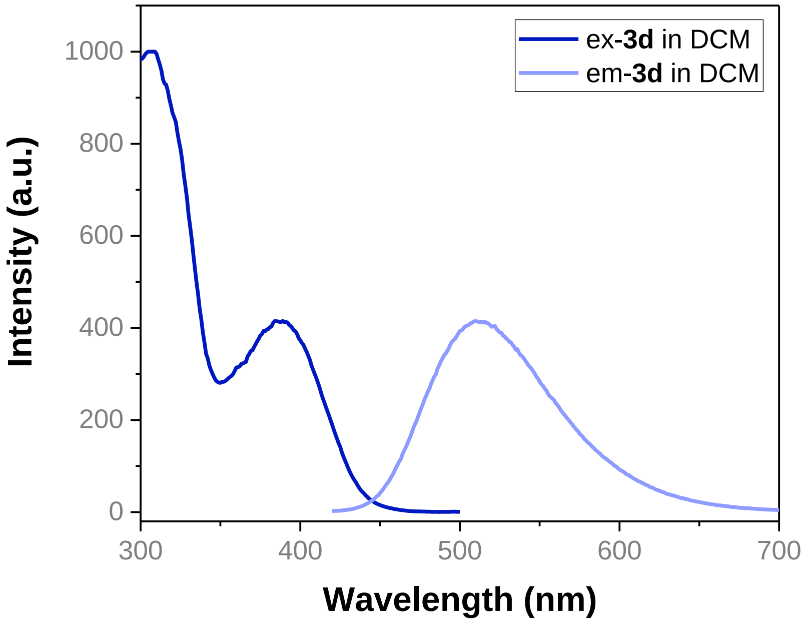

Figure S66. Fluorescence spectrum of **3d** in DCM at 25 ºC, the concentration was ca. 2×10^{-5} M

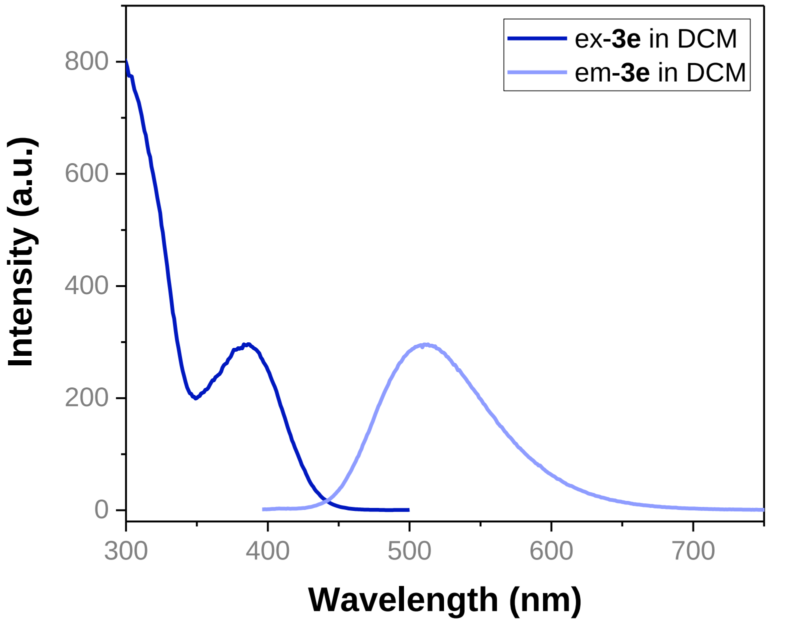

Figure S67. Fluorescence spectrum of **3e** in DCM at 25 ºC, the concentration was ca. 2×10^{-5} M

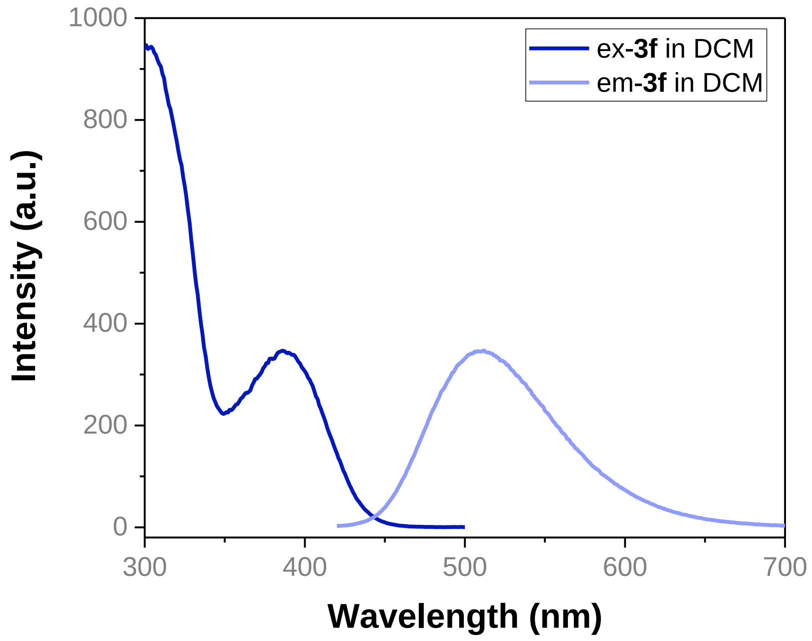

Figure S68. Fluorescence spectrum of **3f** in DCM at 25 °C, the concentration was ca. 2×10^{-5} M

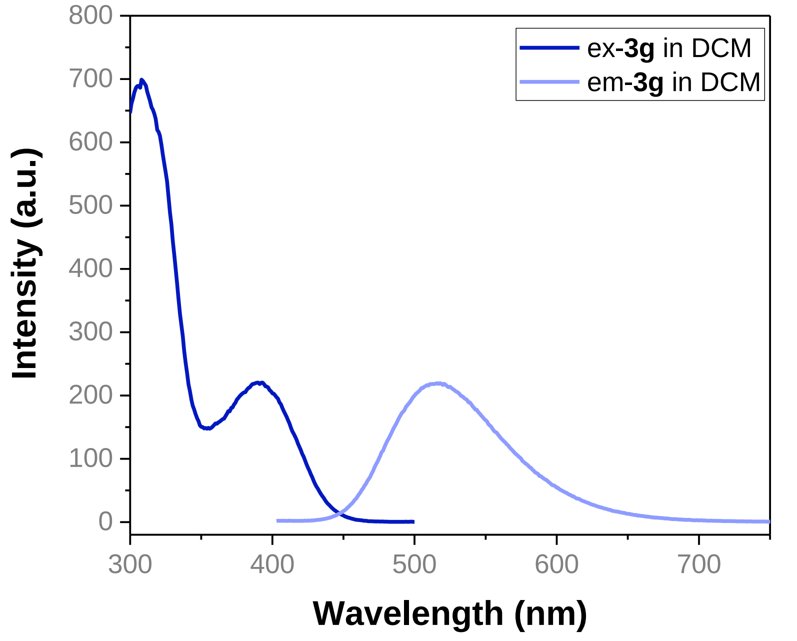

Figure S69. Fluorescence spectrum of **3g** in DCM at 25 °C, the concentration was ca. 2×10^{-5} M

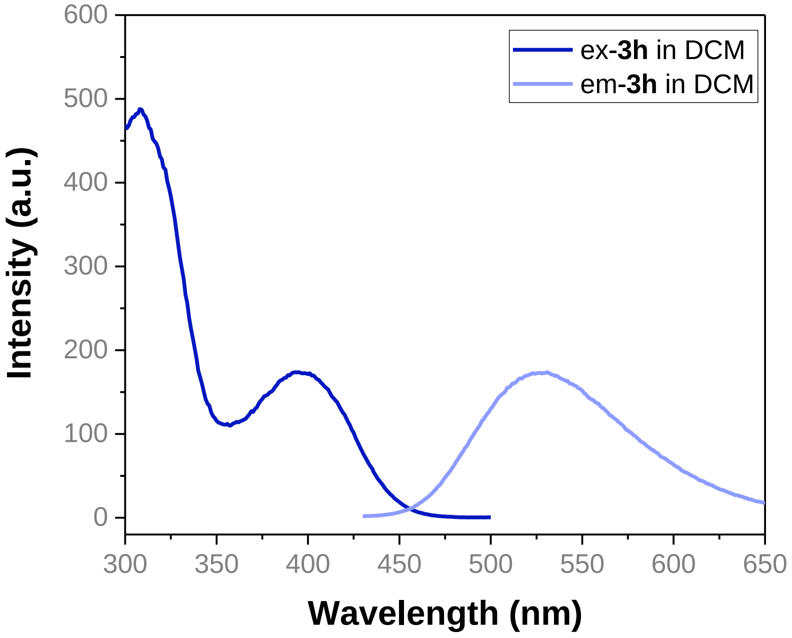

Figure S70. Fluorescence spectrum of **3h** in DCM at 25 ºC, the concentration was ca. 2×10^{-5} M

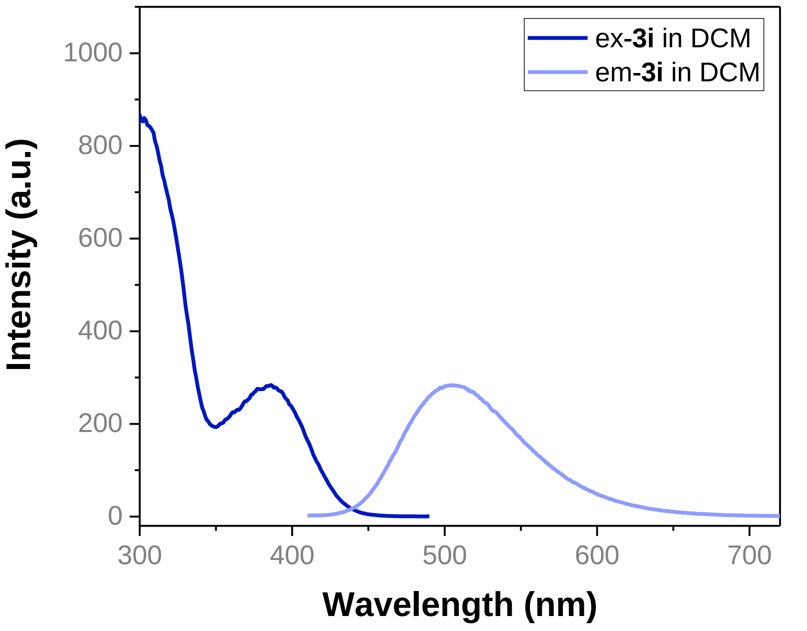

Figure S71. Fluorescence spectrum of **3i** in DCM at 25 ºC, the concentration was ca. 2×10^{-5} M

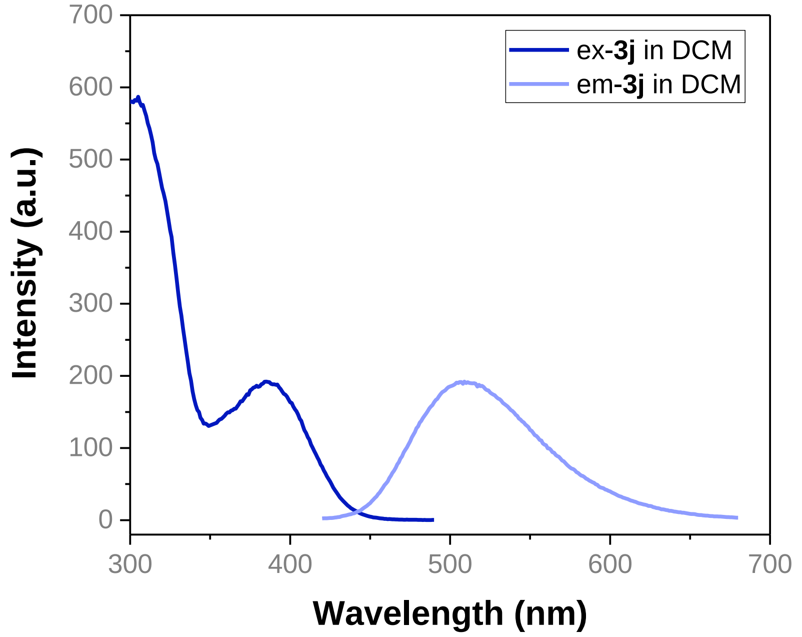

Figure S72. Fluorescence spectrum of **3j** in DCM at 25 ºC, the concentration was ca. 2×10^{-5} M

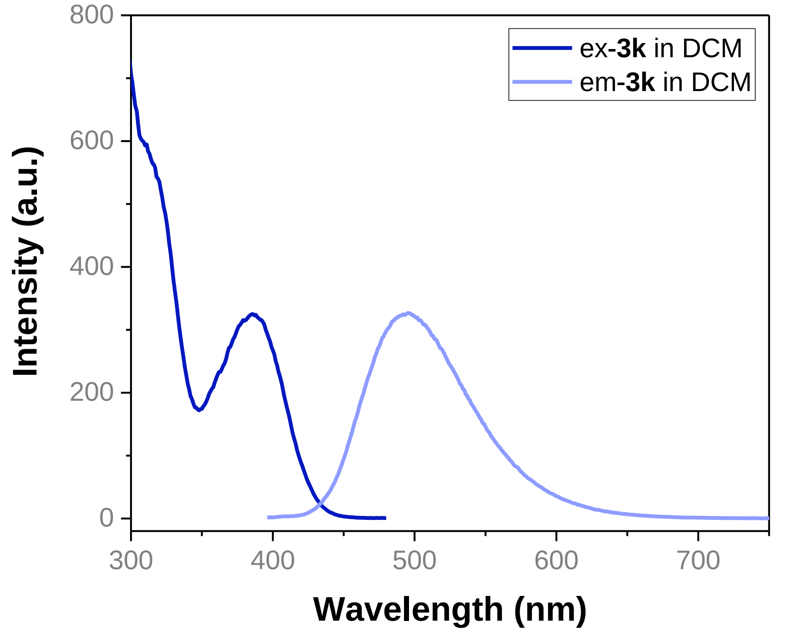

Figure S73. Fluorescence spectrum of **3k** in DCM at 25 ºC, the concentration was ca. 2×10^{-5} M

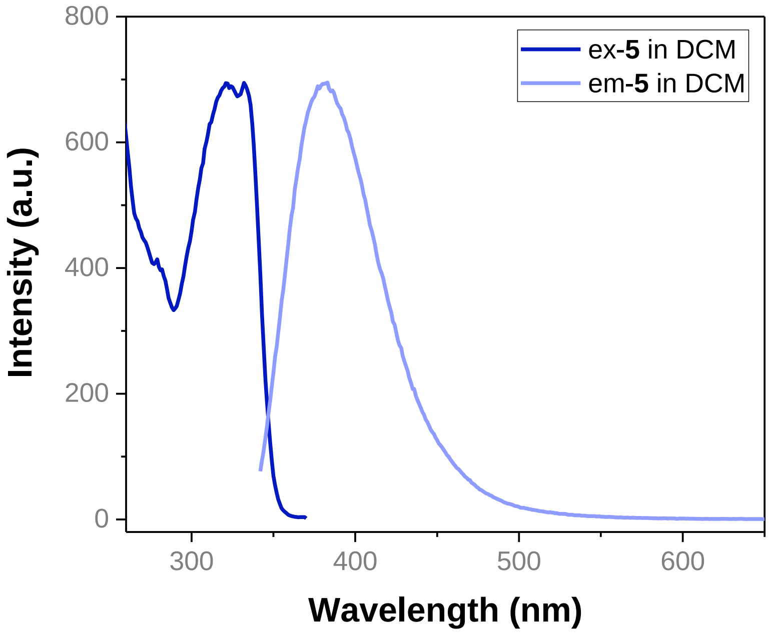

Figure S74. Fluorescence spectrum of **5** in DCM at 25 ºC, the concentration was ca. 2×10^{-5} M

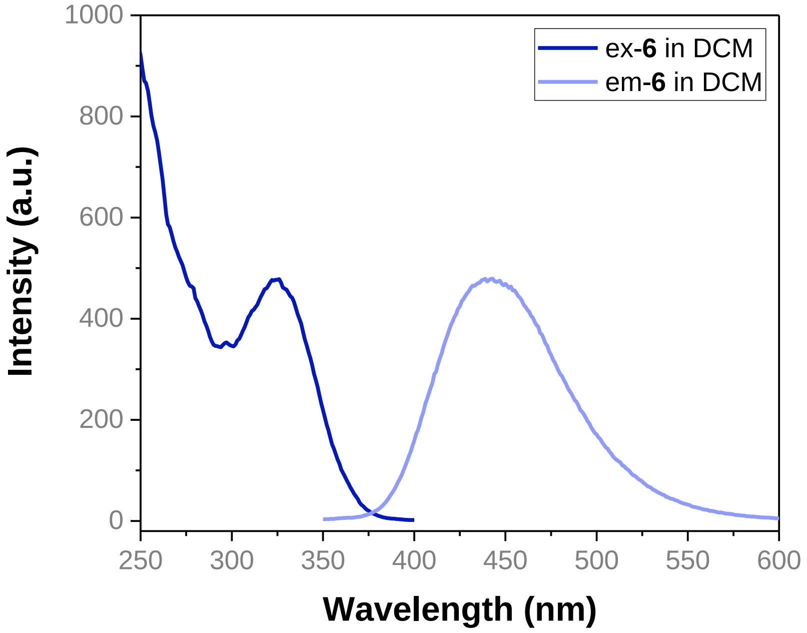

Figure S75. Fluorescence spectrum of **6** in DCM at 25 ºC, the concentration was ca. 2×10^{-5} M

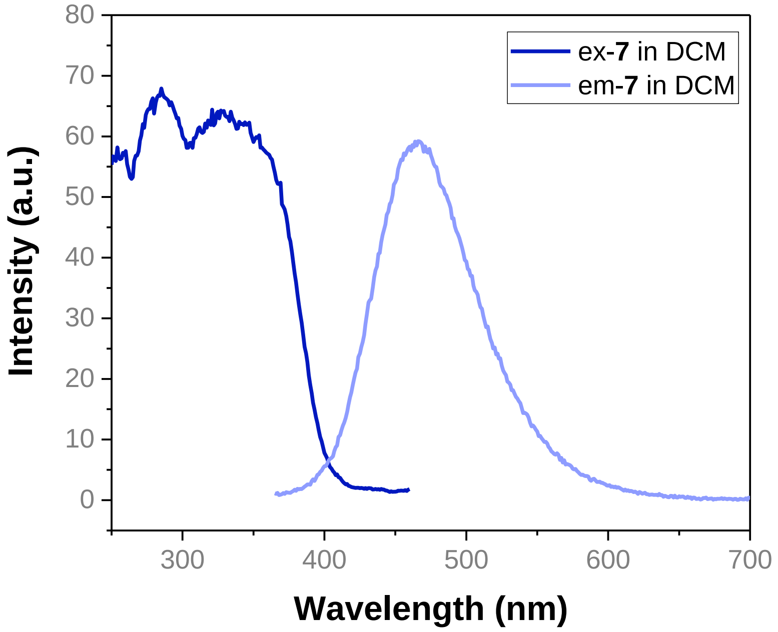

Figure S76. Fluorescence spectrum of **7** in DCM at 25 °C, the concentration was ca. 2×10^{-5} M

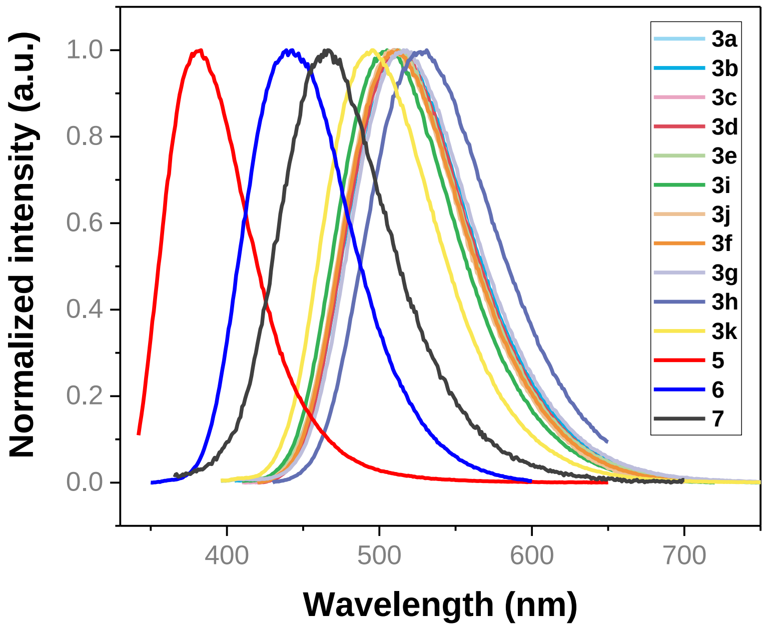

Figure S77. Normalized Fluorescence spectra of **3a-3k**, **5**, **6**, and **7** in DCM at 25 °C.

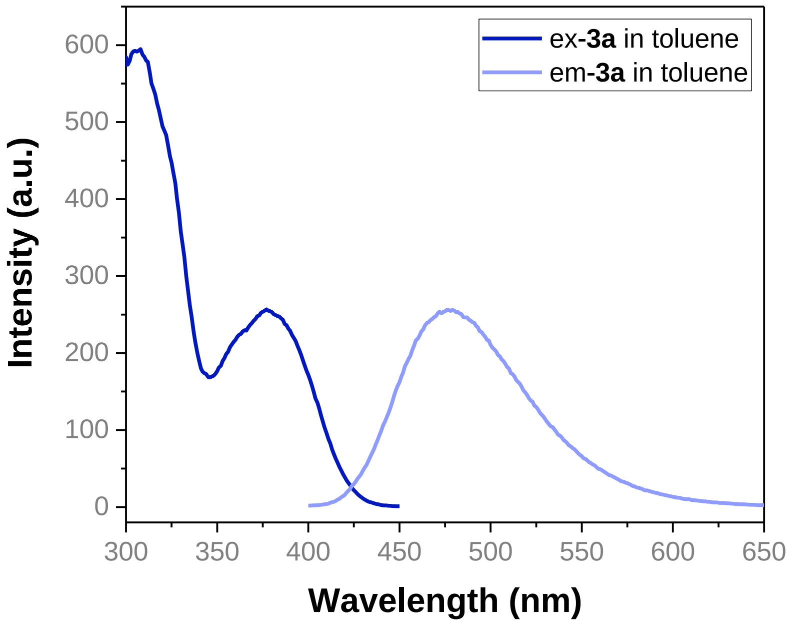

Figure S78. Fluorescence spectrum of **3a** in toluene at 25 ºC, the concentration was ca. 2×10^{-5} M

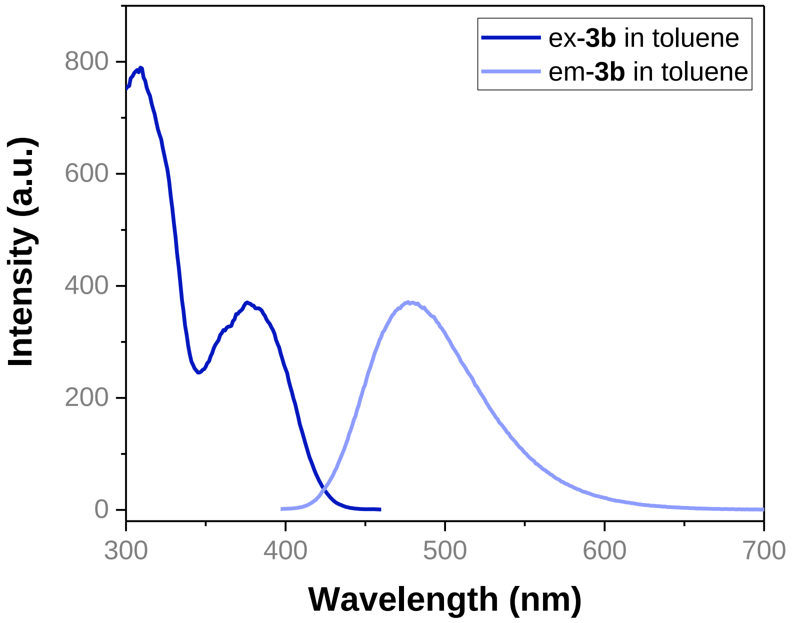

Figure S79. Fluorescence spectrum of **3b** in toluene at 25 ºC, the concentration was ca. 2×10^{-5} M

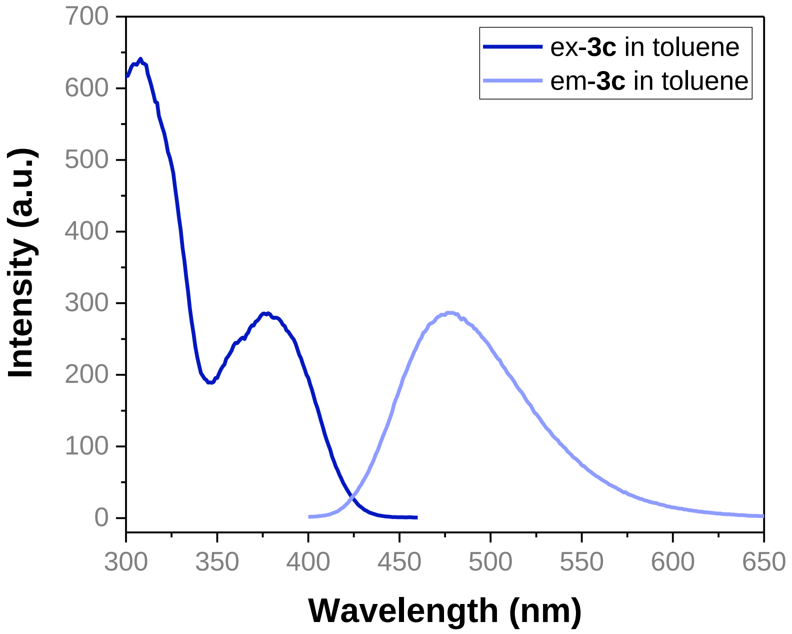

Figure S80. Fluorescence spectrum of **3c** in toluene at 25 ºC, the concentration was ca. 2×10^{-5} M

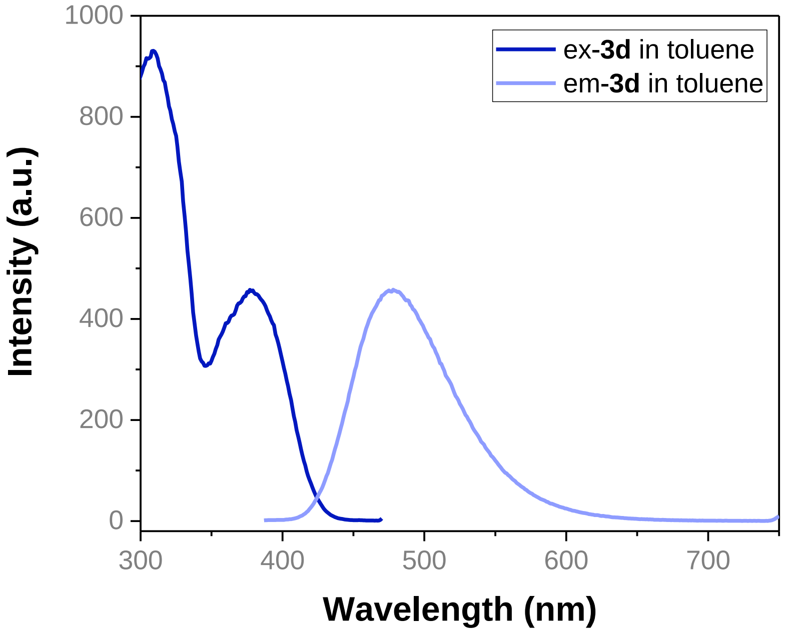

Figure S81. Fluorescence spectrum of **3d** in toluene at 25 ºC, the concentration was ca. 2×10^{-5} M

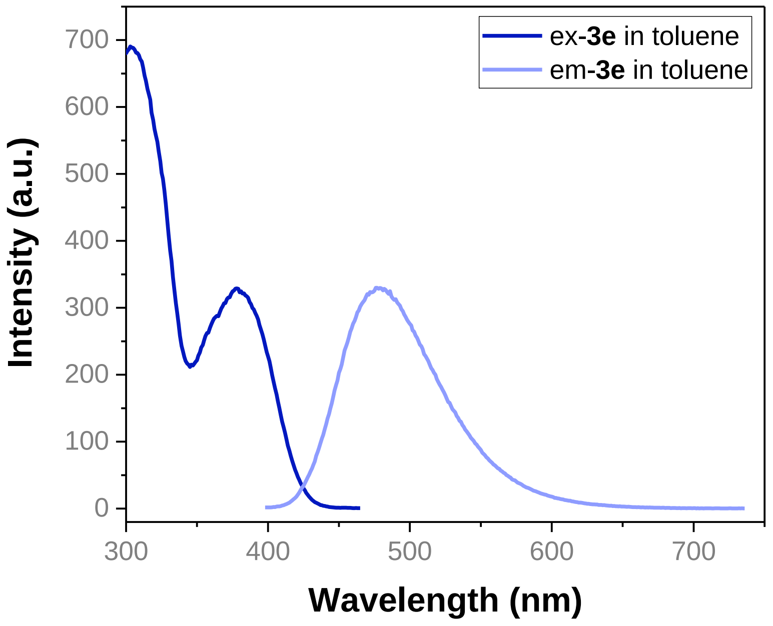

Figure S82. Fluorescence spectrum of **3e** in toluene at 25 ºC, the concentration was ca. 2×10^{-5} M

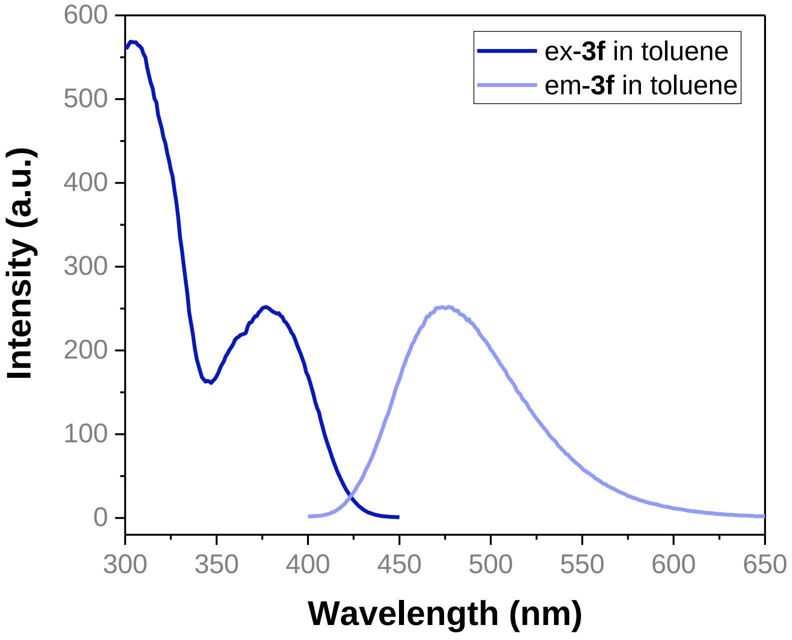

Figure S83. Fluorescence spectrum of **3f** in toluene at 25 ºC, the concentration was ca. 2×10^{-5} M

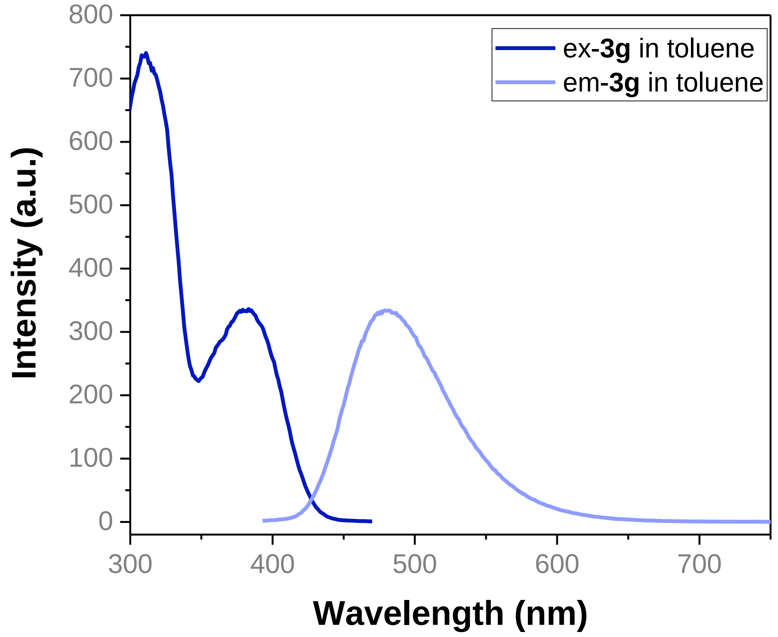

Figure S84. Fluorescence spectrum of **3g** in toluene at 25 ºC, the concentration was ca. 2×10^{-5} M

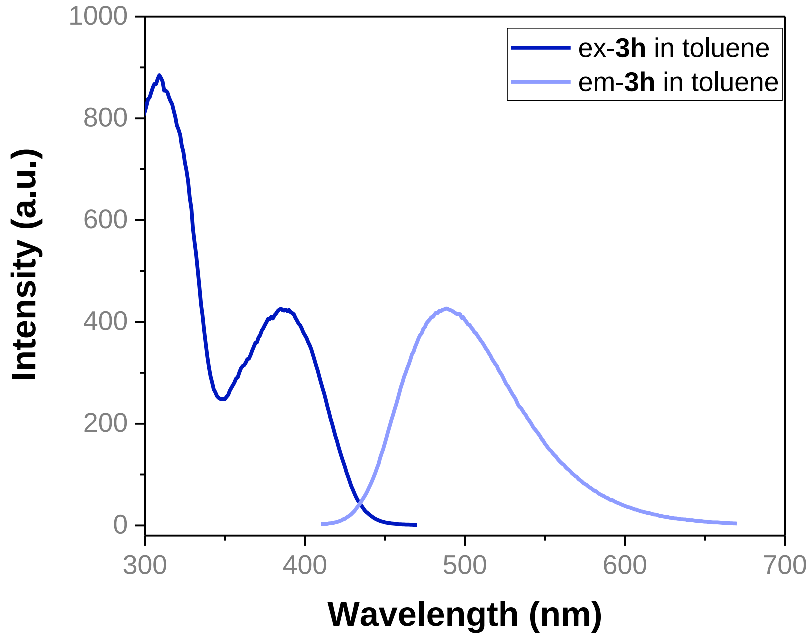

Figure S85. Fluorescence spectrum of **3h** in toluene at 25 ºC, the concentration was ca. 2×10^{-5} M

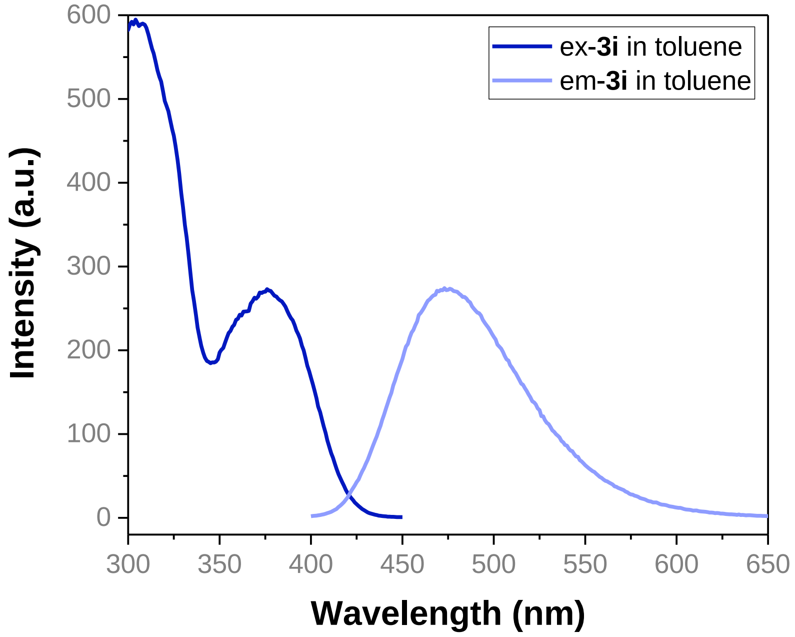

Figure S86. Fluorescence spectrum of **3i** in toluene at 25 °C, the concentration was ca. 2×10^{-5} M

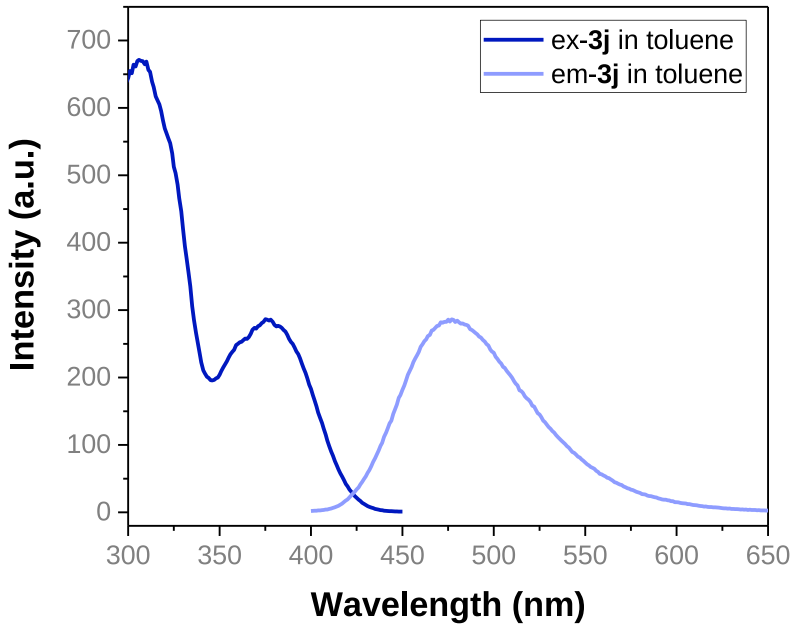

Figure S87. Fluorescence spectrum of **3j** in toluene at 25 °C, the concentration was ca. 2×10^{-5} M

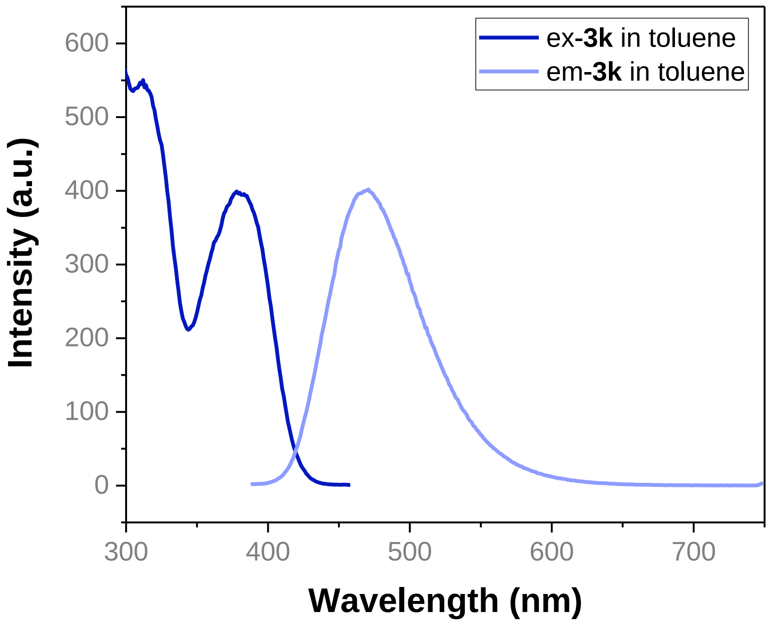

Figure S88. Fluorescence spectrum of **3k** in toluene at 25 °C, the concentration was ca. 2×10^{-5} M

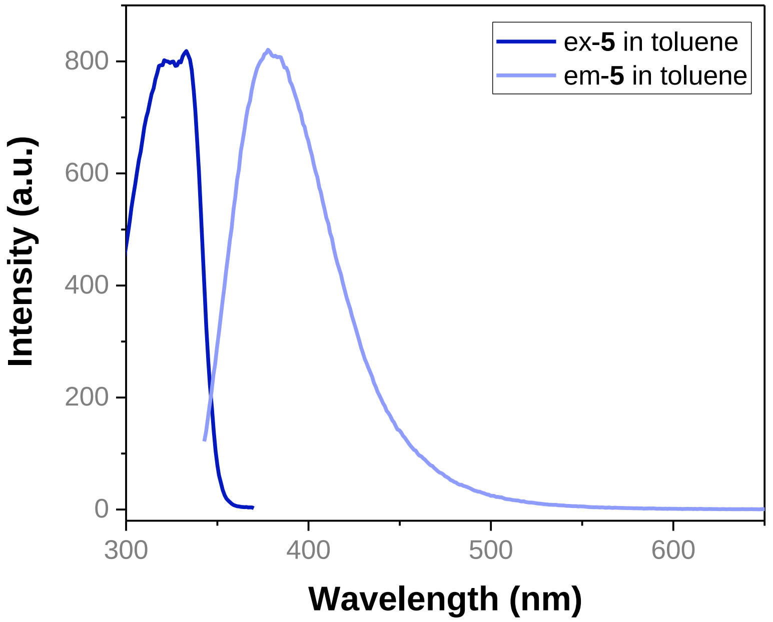

Figure S89. Fluorescence spectrum of **5** in toluene at 25 °C, the concentration was ca. 2×10^{-5} M

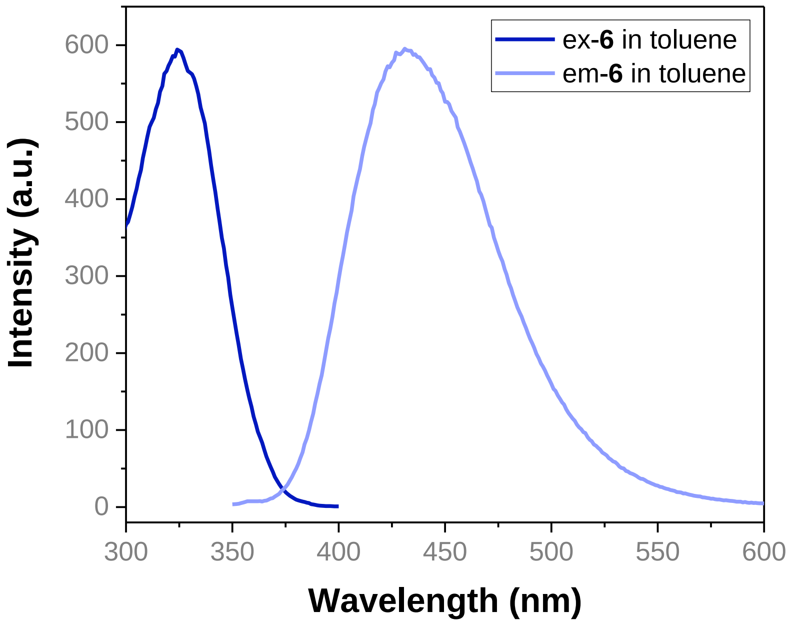

Figure S90. Fluorescence spectrum of **6** in toluene at 25 °C, the concentration was ca. 2×10^{-5} M

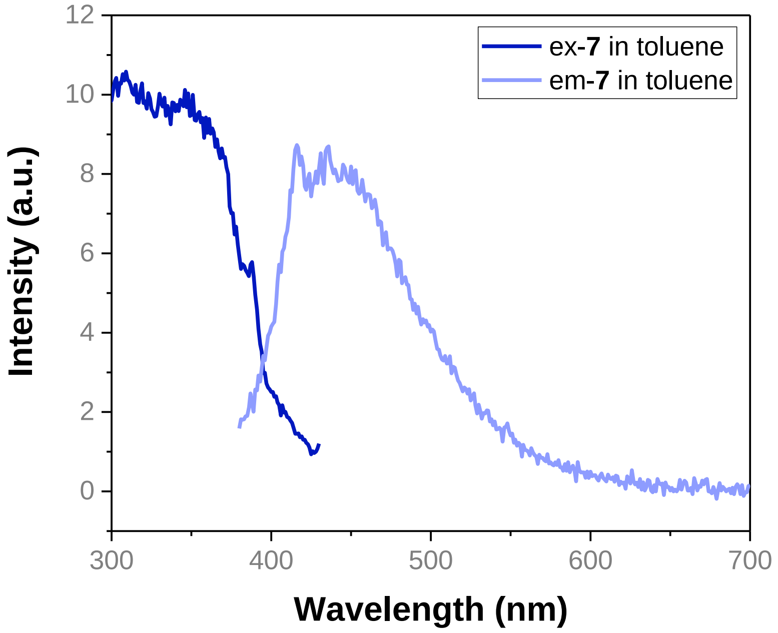

Figure S91. Fluorescence spectrum of **7** in toluene at 25 °C, the concentration was ca. 5×10^{-5} M

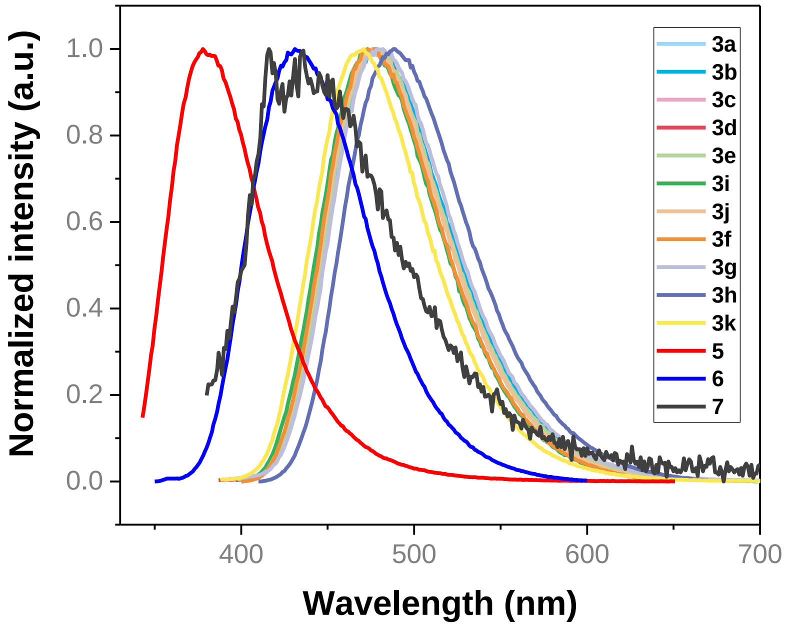

Figure S92. Normalized Fluorescence spectra of **3a**-**3k**, **5**, **6**, and **7** in toluene at 25 °C.

17. Copies of CD spectra of 3a-3k, 5, 6, and 7

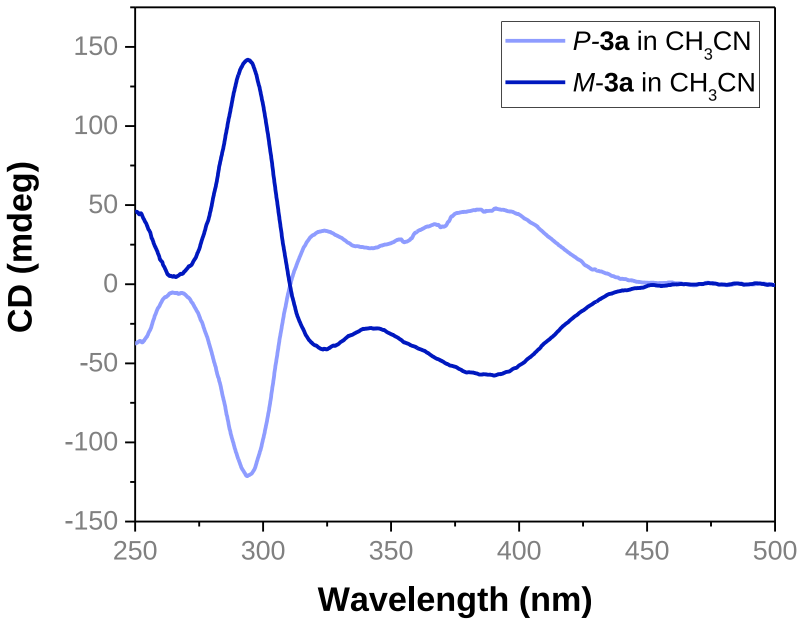

Figure S93. CD spectra of *P*-**3a** and *M*-**3a** in CH_3CN at 25 °C (ca. 5×10^{-5} M)

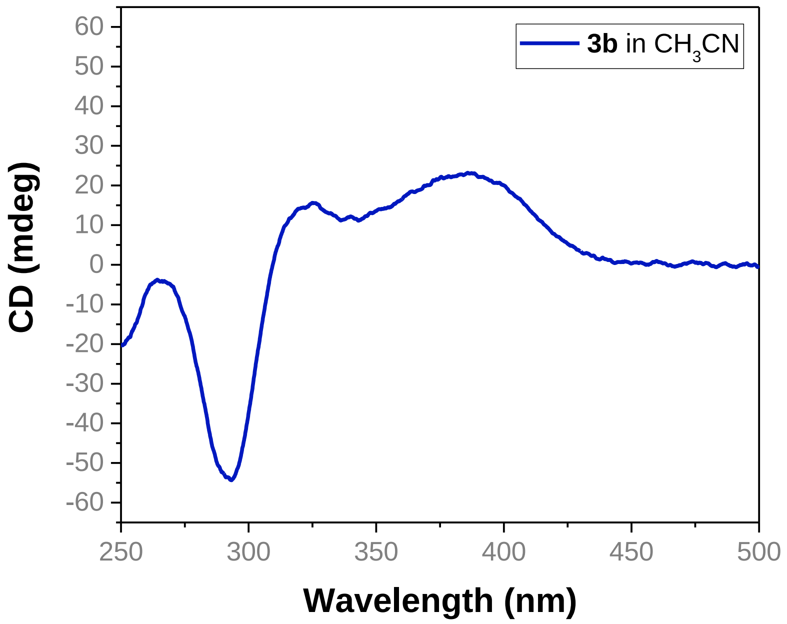

Figure S94. CD spectrum of **3b** in CH_3CN at 25 °C (ca. 5×10^{-5} M)

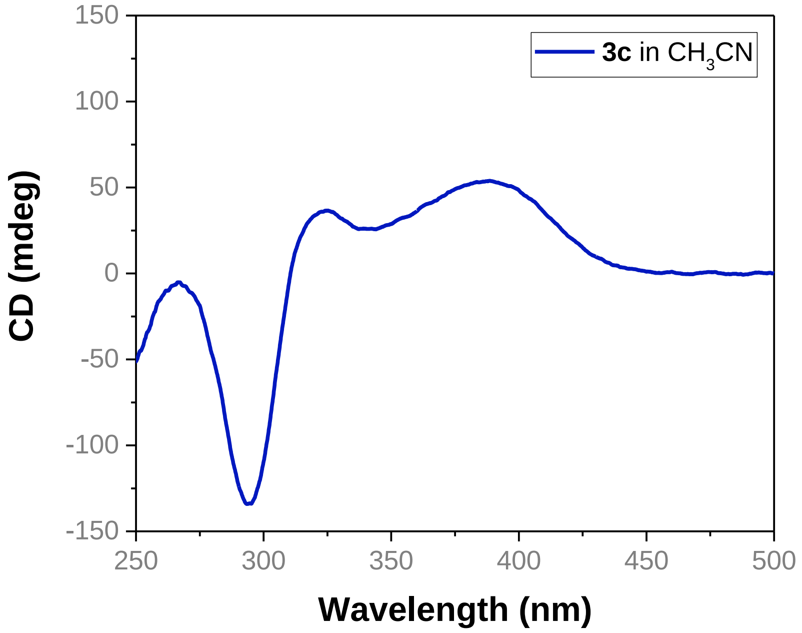

Figure S95. CD spectrum of **3c** in CH_3CN at 25 °C (ca. 5×10^{-5} M)

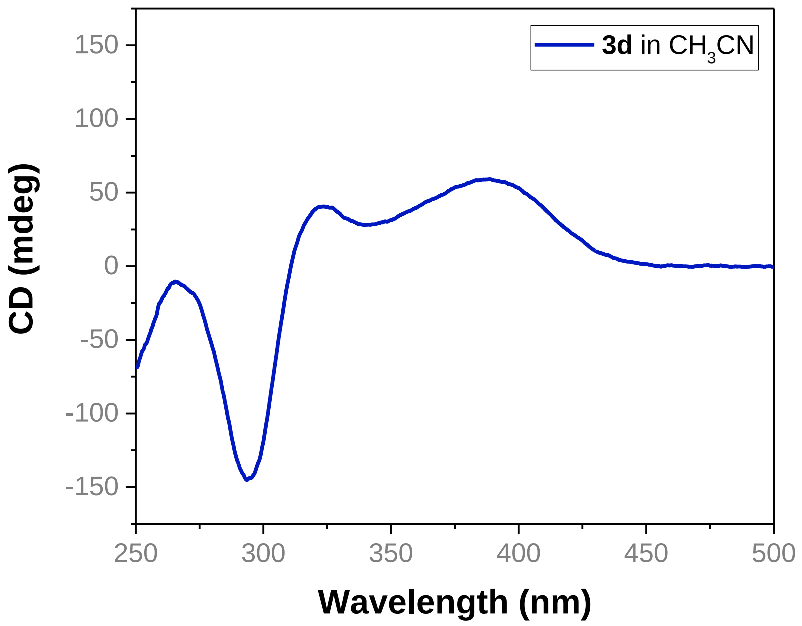

Figure S96. CD spectrum of **3d** in CH_3CN at 25 °C (ca. 5×10^{-5} M)

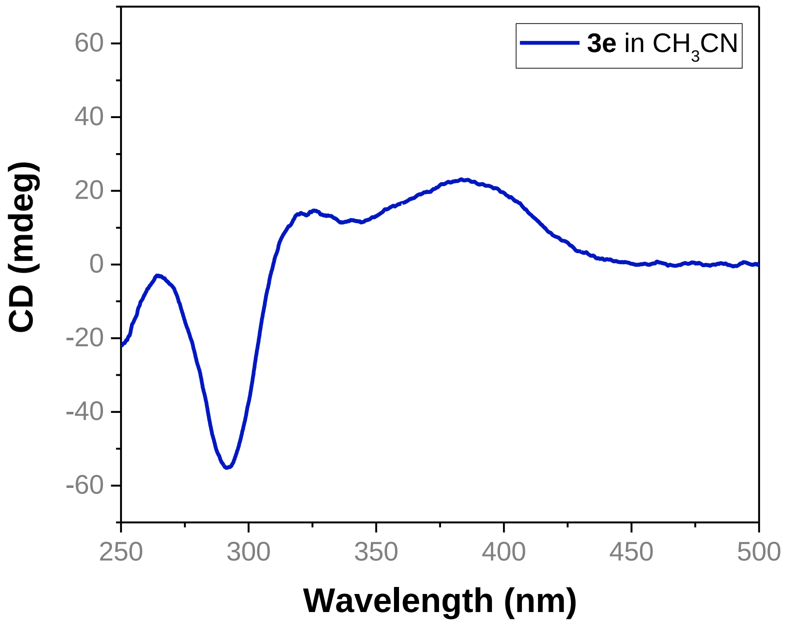

Figure S97. CD spectrum of **3e** in CH_3CN at 25 °C (ca. 5×10^{-5} M)

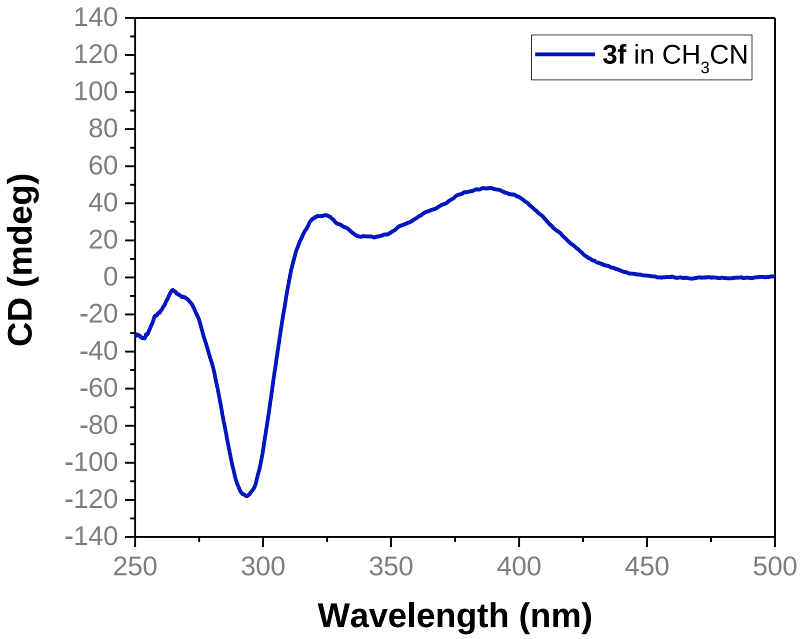

Figure S98. CD spectrum of **3f** in CH_3CN at 25 °C (ca. 5×10^{-5} M)

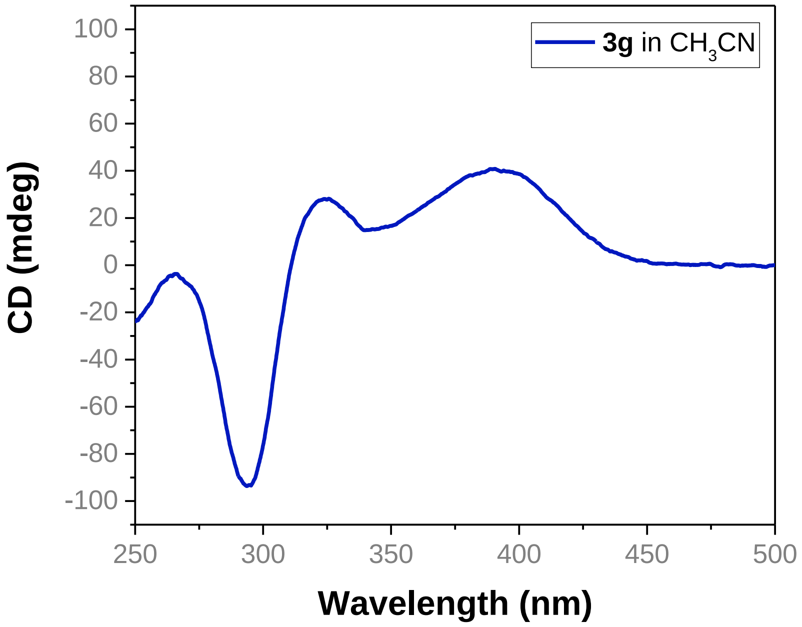

Figure S99. CD spectrum of **3g** in CH_3CN at 25 °C (ca. 5×10^{-5} M)

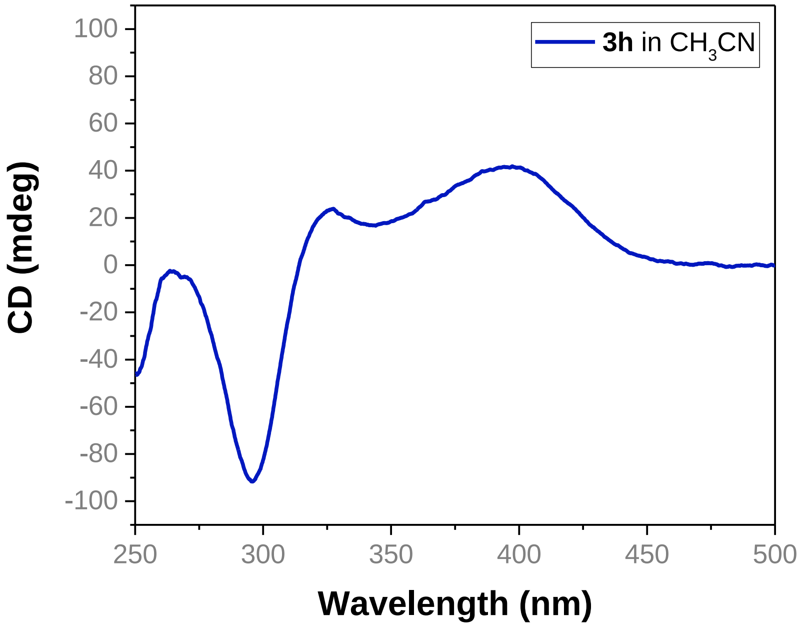

Figure S100. CD spectrum of **3h** in CH_3CN at 25 °C (ca. 5×10^{-5} M)

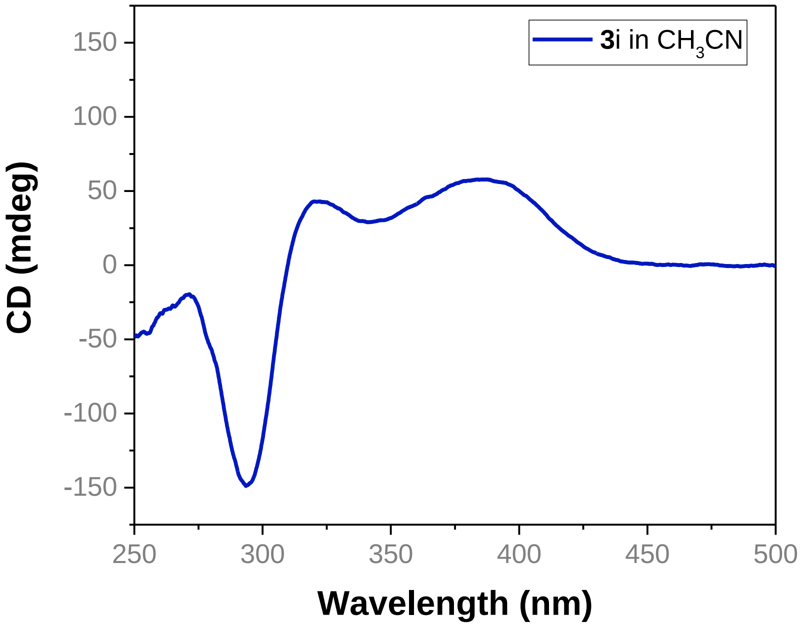

Figure S101. CD spectrum of **3i** in CH_3CN at 25 °C (ca. 5×10^{-5} M)

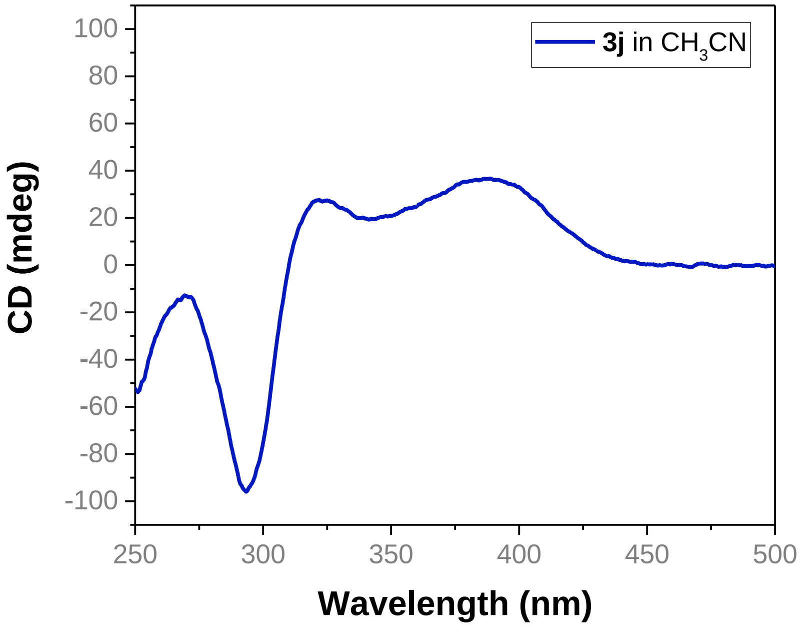

Figure S102. CD spectrum of **3j** in CH_3CN at 25 °C (ca. 5×10^{-5} M)

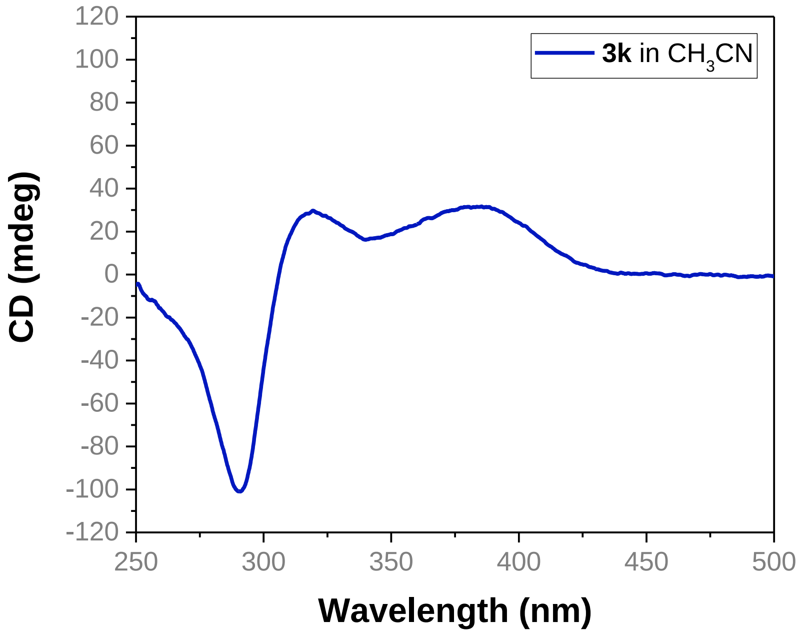

Figure S103. CD spectrum of **3k** in CH_3CN at 25 °C (ca. 5×10^{-5} M)

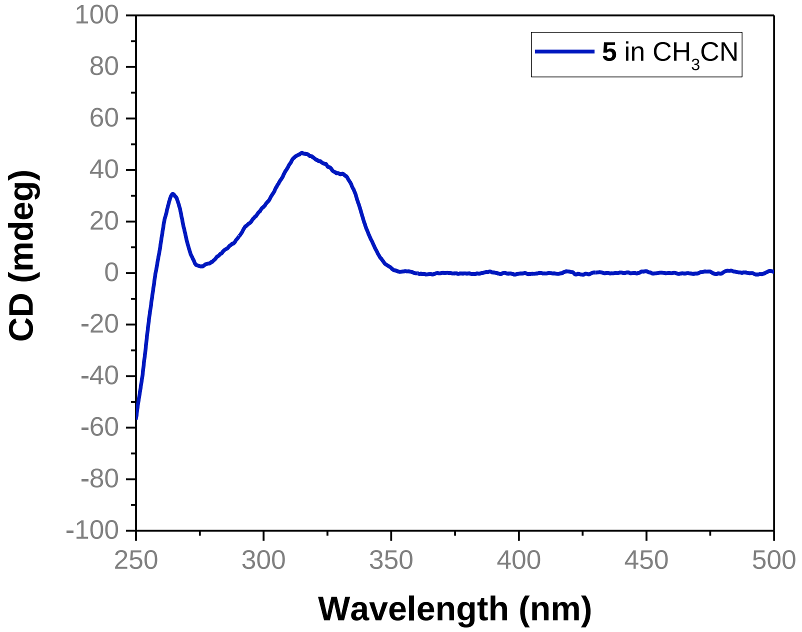

Figure S104. CD spectrum of **5** in CH_3CN at 25 °C (ca. 5×10^{-5} M)

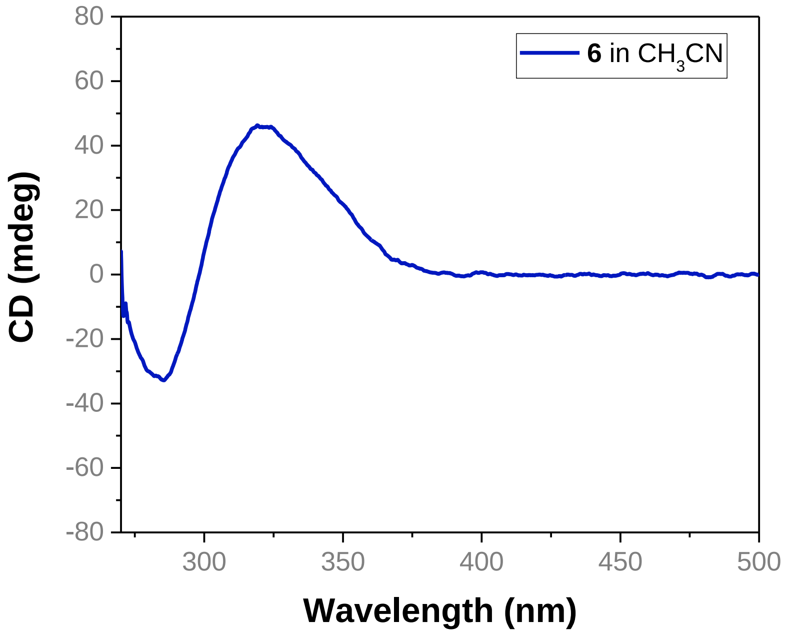

Figure S105. CD spectrum of **6** in CH_3CN at 25 ºC (ca. 4×10^{-5} M)

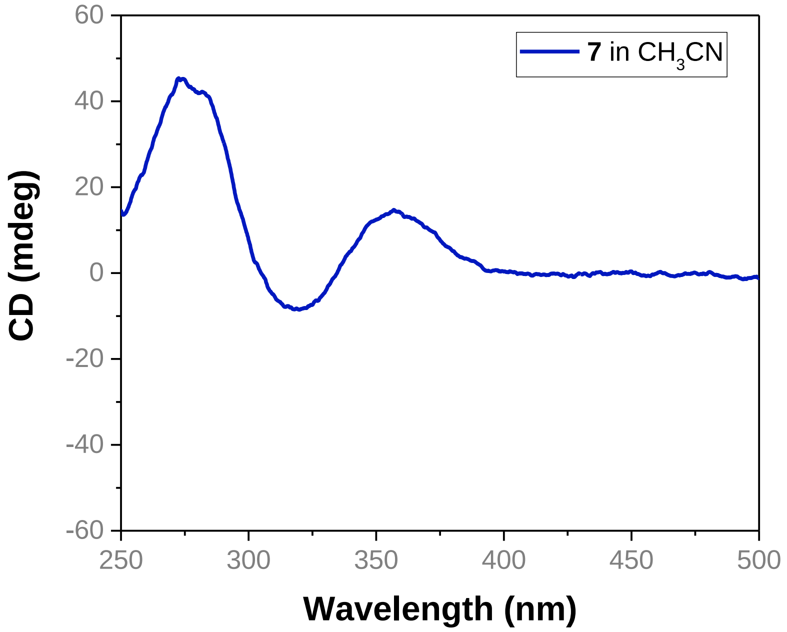

Figure S106. CD spectrum of **7** in CH_3CN at 25 ºC (ca. 4×10^{-5} M)

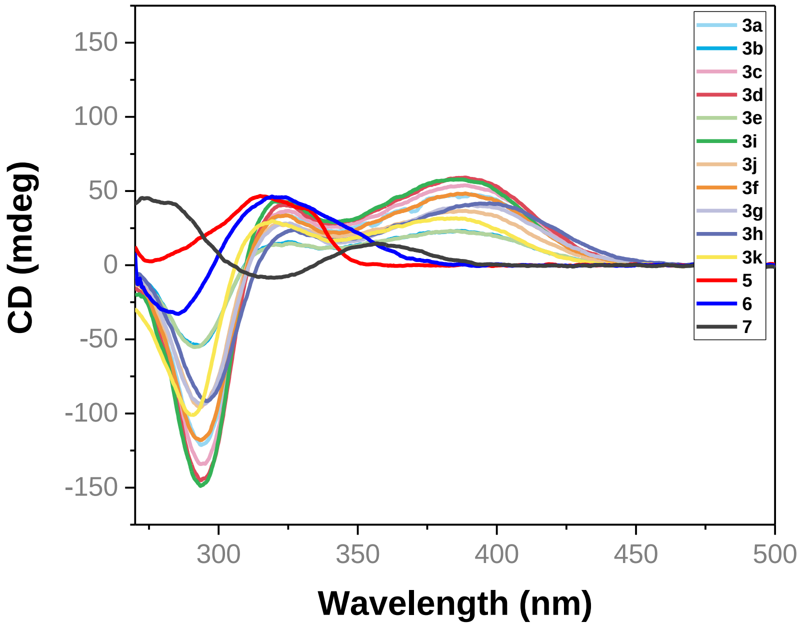

Figure S107. CD spectra of **3a**-**3k**, **5**, **6**, and **7** in CH_3CN at 25 ^oC

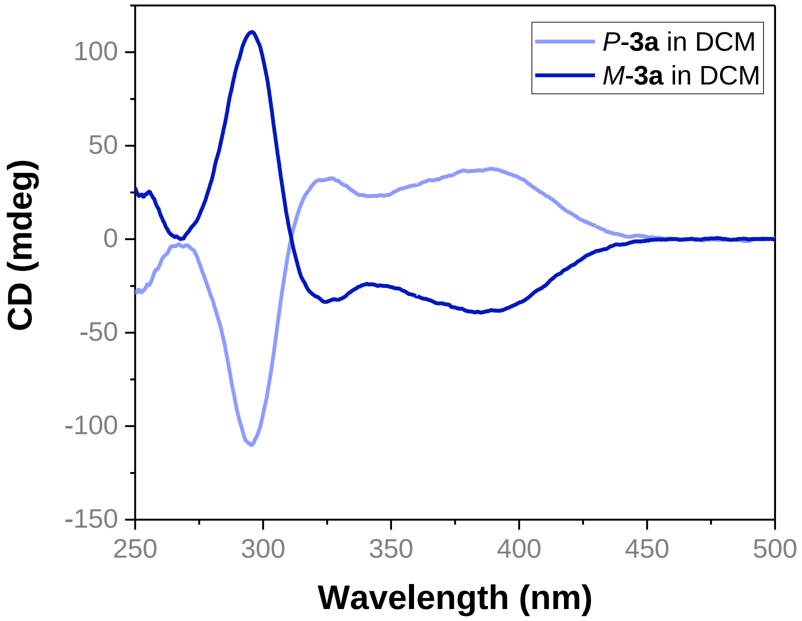

Figure S108. CD spectra of *P*-**3a and** *M*-**3a** in DCM at 25 ^oC (ca. 5×10^{-5} M)

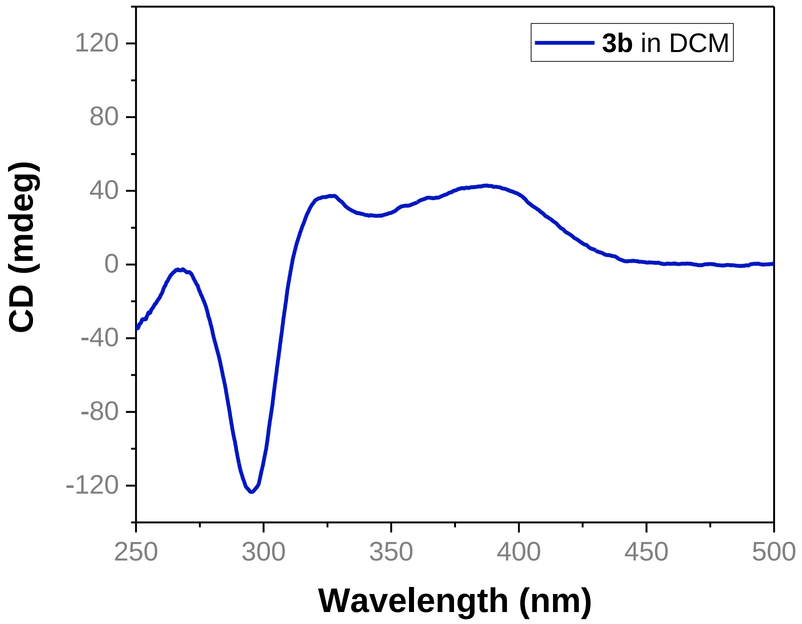

Figure S109. CD spectrum of **3b** in DCM at 25 ºC (ca. 5×10^{-5} M)

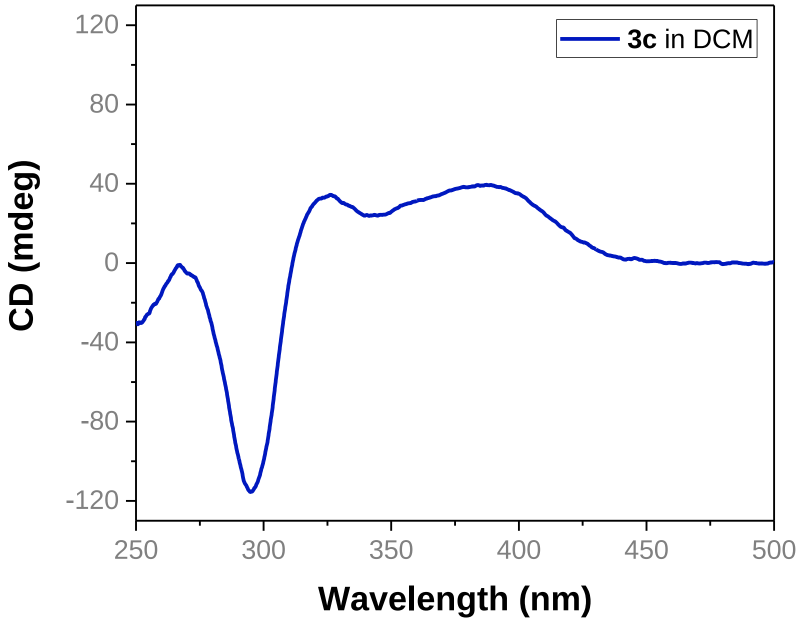

Figure S110. CD spectrum of **3c** in DCM at 25 ºC (ca. 5×10^{-5} M)

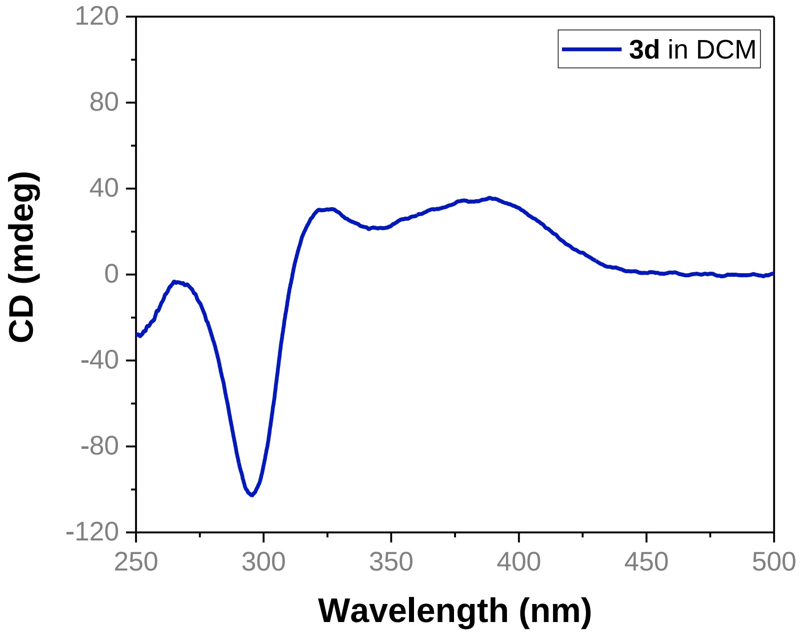

Figure S111. CD spectrum of **3d** in DCM at 25 ºC (ca. 5×10^{-5} M)

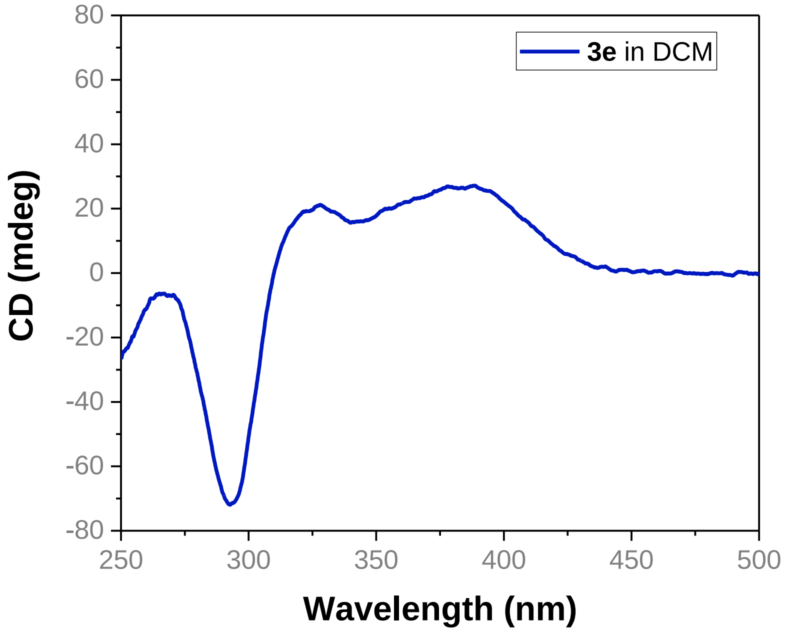

Figure S112. CD spectrum of **3e** in DCM at 25 ºC (ca. 5×10^{-5} M)

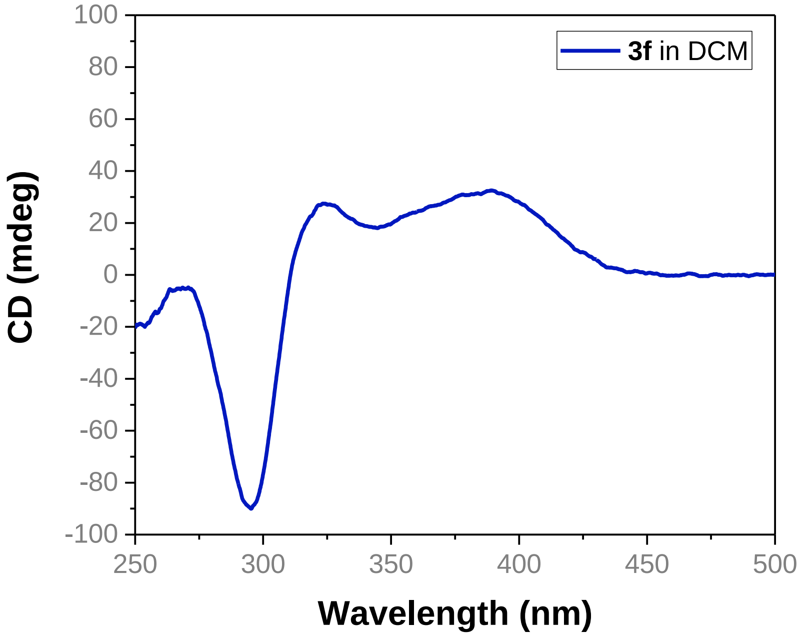

Figure S113. CD spectrum of **3f** in DCM at 25 ºC (ca. 5×10^{-5} M)

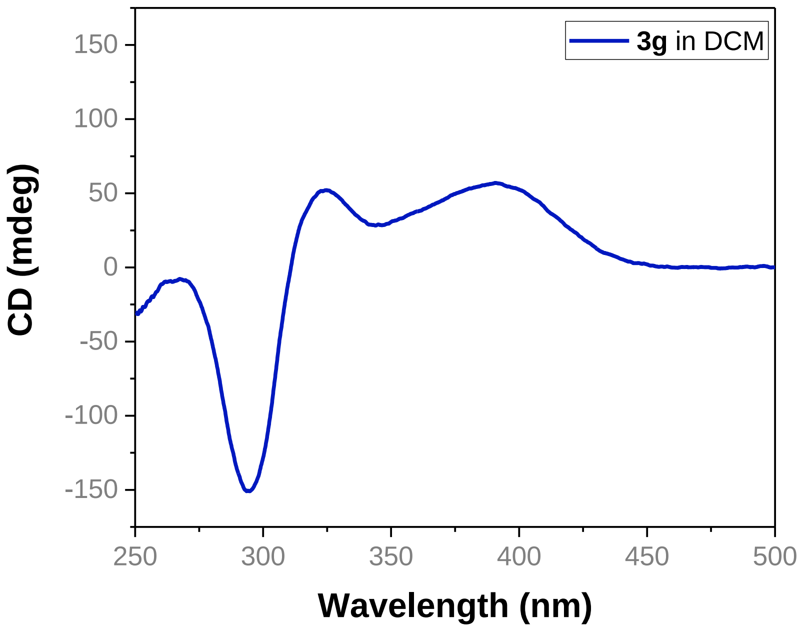

Figure S114. CD spectrum of **3g** in DCM at 25 ºC (ca. 5×10^{-5} M)

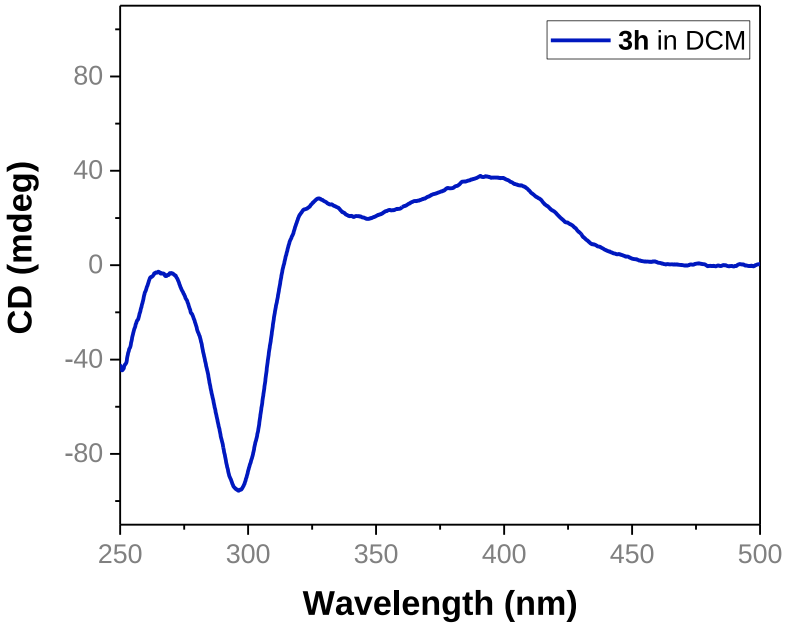

Figure S115. CD spectrum of **3h** in DCM at 25 ºC (ca. 5×10^{-5} M)

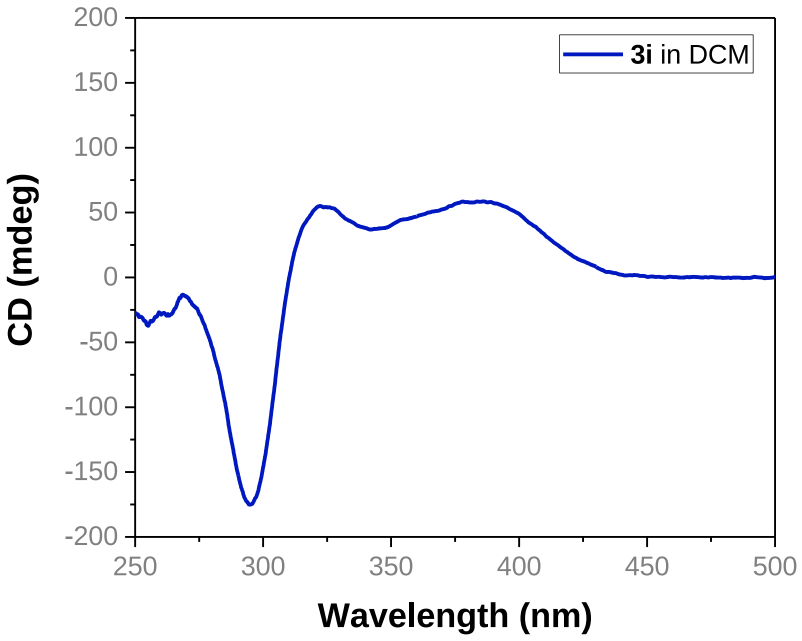

Figure S116. CD spectrum of **3i** in DCM at 25 ºC (ca. 5×10^{-5} M)

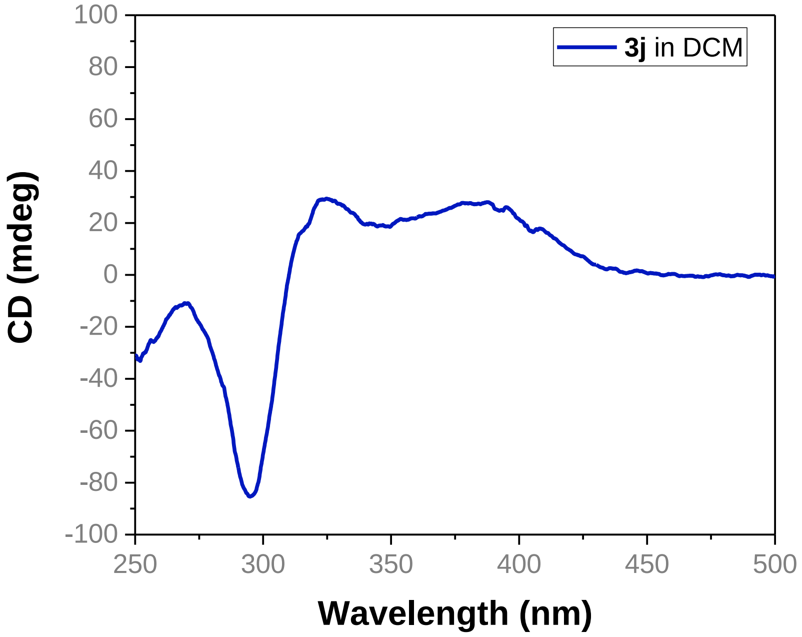

Figure S117. CD spectrum of **3j** in DCM at 25 ºC (ca. 5×10^{-5} M)

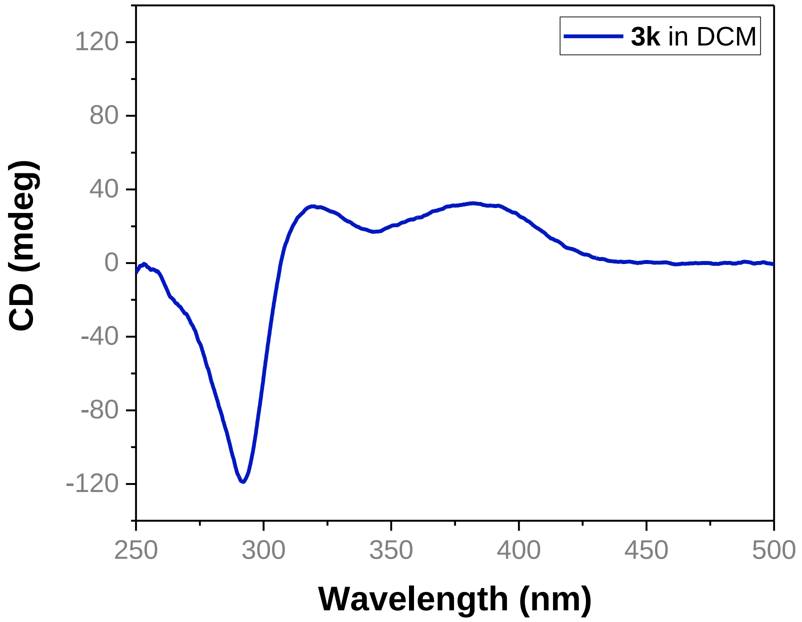

Figure S118. CD spectrum of **3k** in DCM at 25 ºC (ca. 5×10^{-5} M)

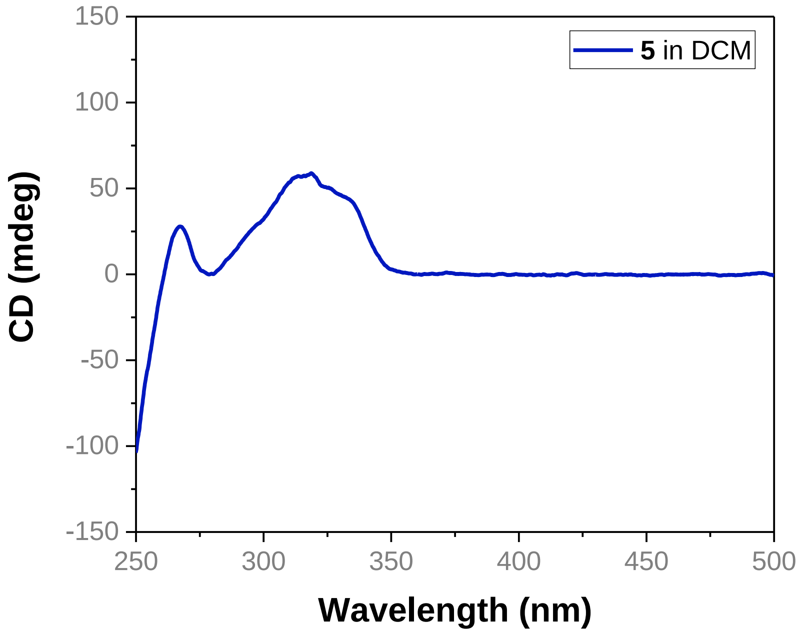

Figure S119. CD spectrum of **5** in DCM at 25 ºC (ca. 5×10^{-5} M)

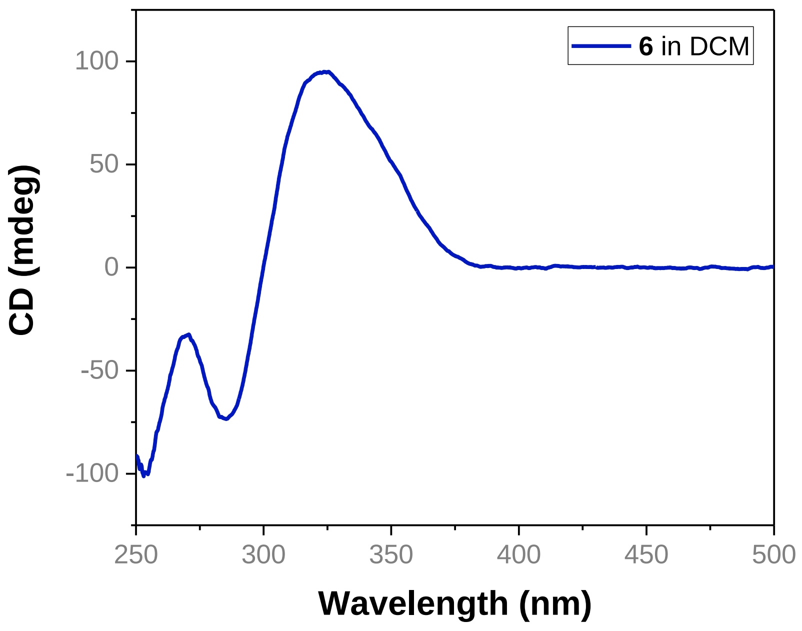

Figure S120. CD spectrum of **6** in DCM at 25 ºC (ca. 4×10^{-5} M)

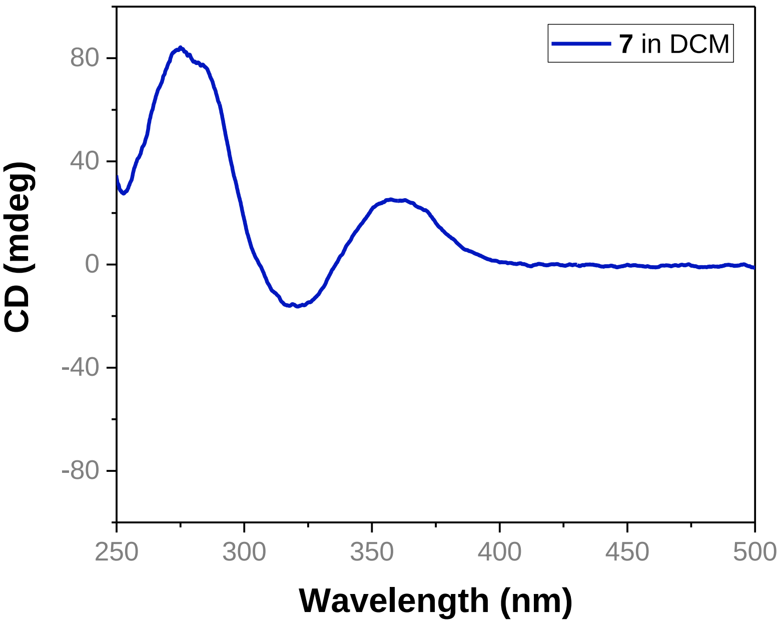

Figure S121. CD spectrum of **7** in DCM at 25 ºC (ca. 4×10^{-5} M)

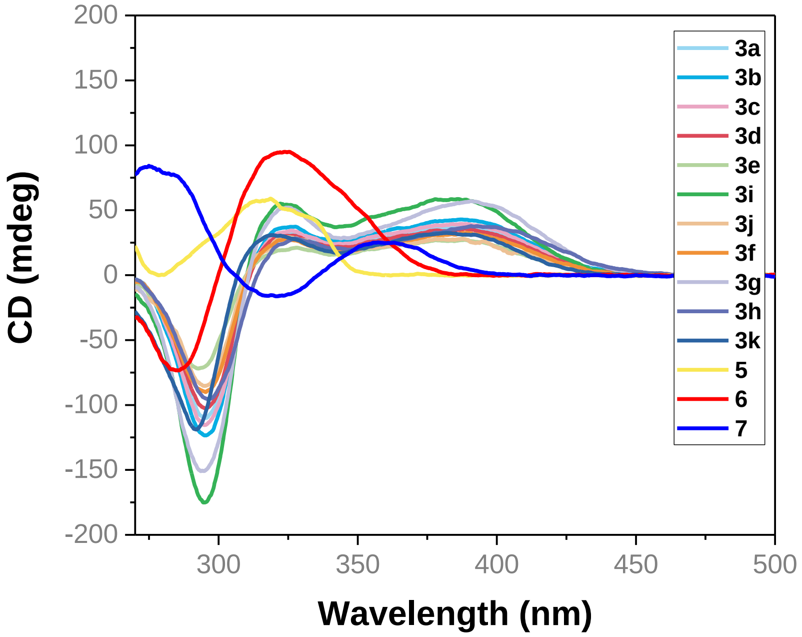

Figure S122. CD spectra of **3a**-**3k**, **5**, **6**, and **7** in DCM at 25 ºC

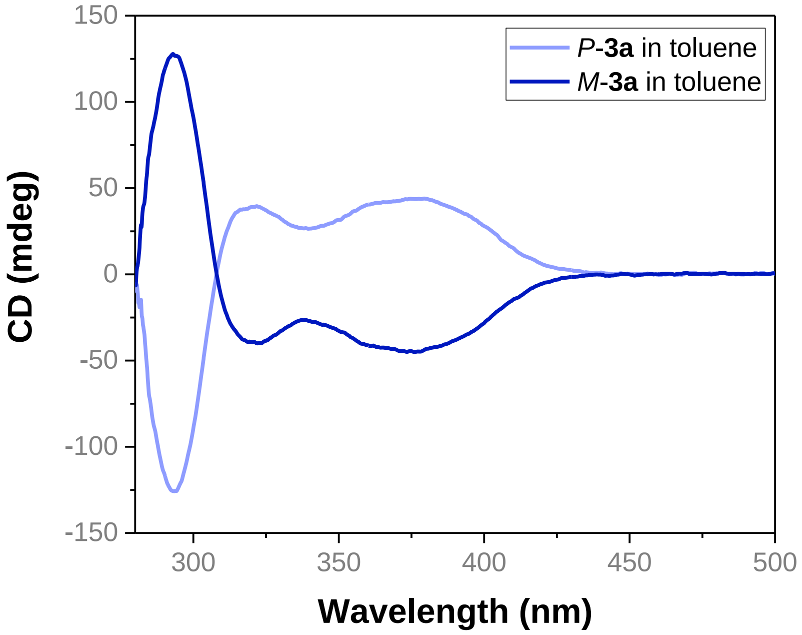

Figure S123. CD spectra of *P*-**3a and** *M*-**3a** in toluene at 25 ºC (ca. 5×10^{-5} M)

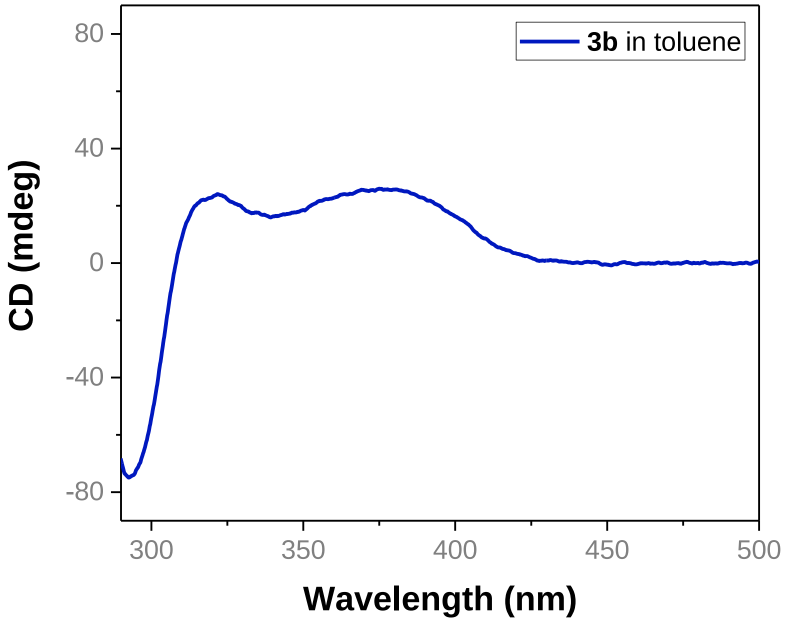

Figure S124. CD spectrum of **3b** in toluene at 25 ºC (ca. 5×10^{-5} M)

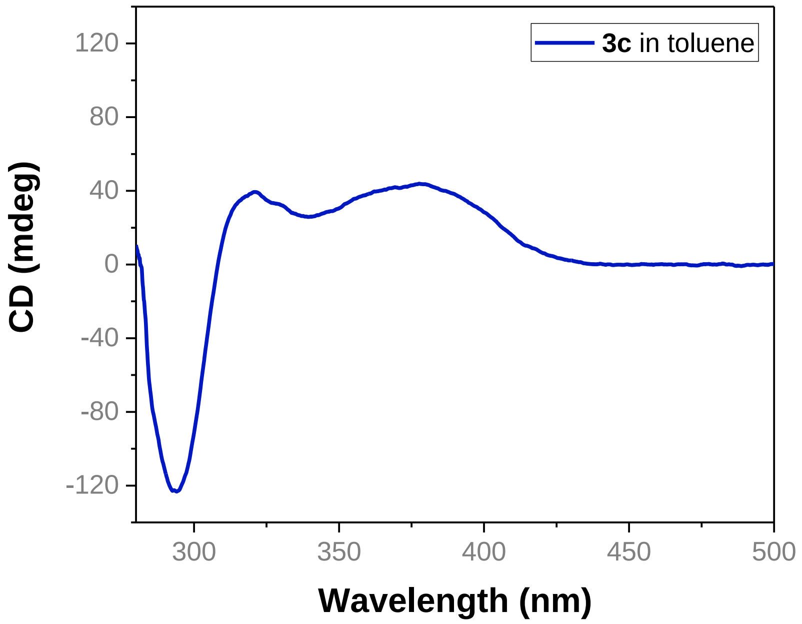

Figure S125. CD spectrum of **3c** in toluene at 25 ºC (ca. 5×10^{-5} M)

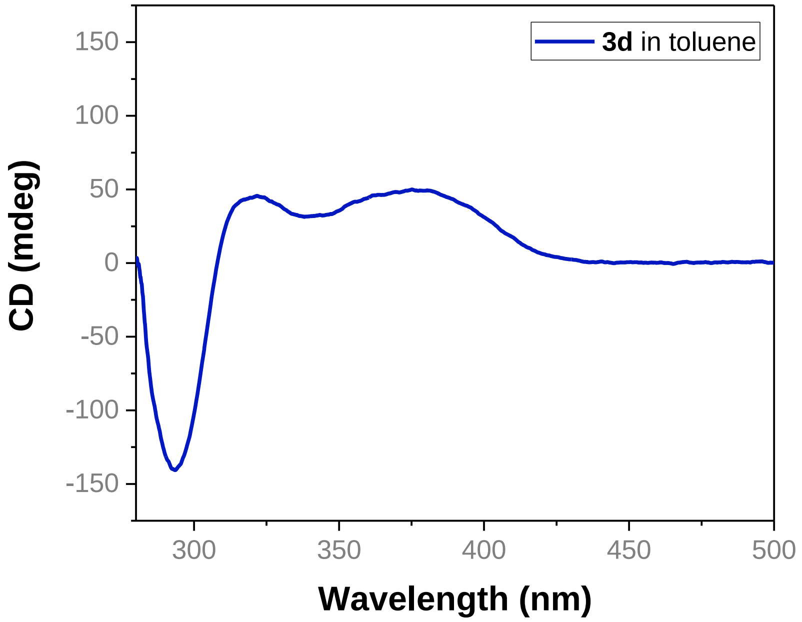

Figure S126. CD spectrum of **3d** in toluene at 25 ºC (ca. 5×10^{-5} M)

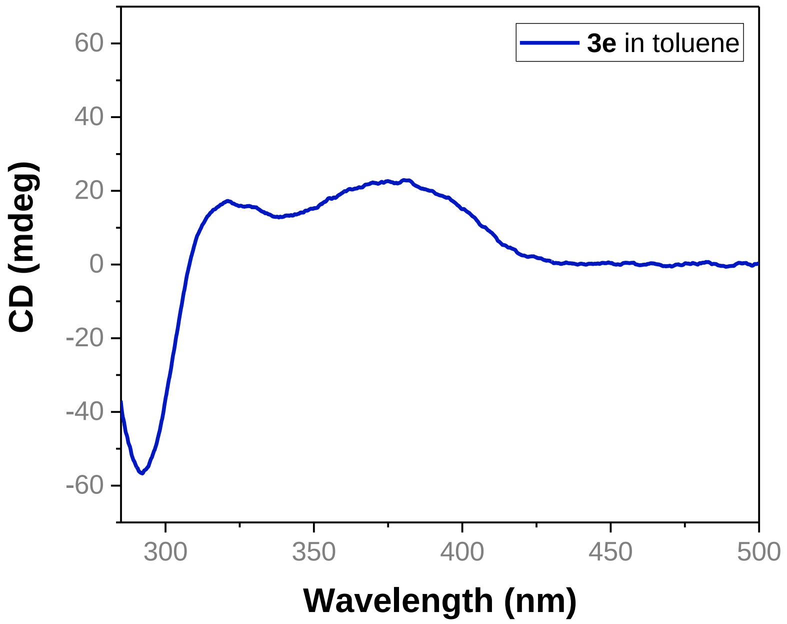

Figure S127. CD spectrum of **3e** in toluene at 25 ºC (ca. 5×10^{-5} M)

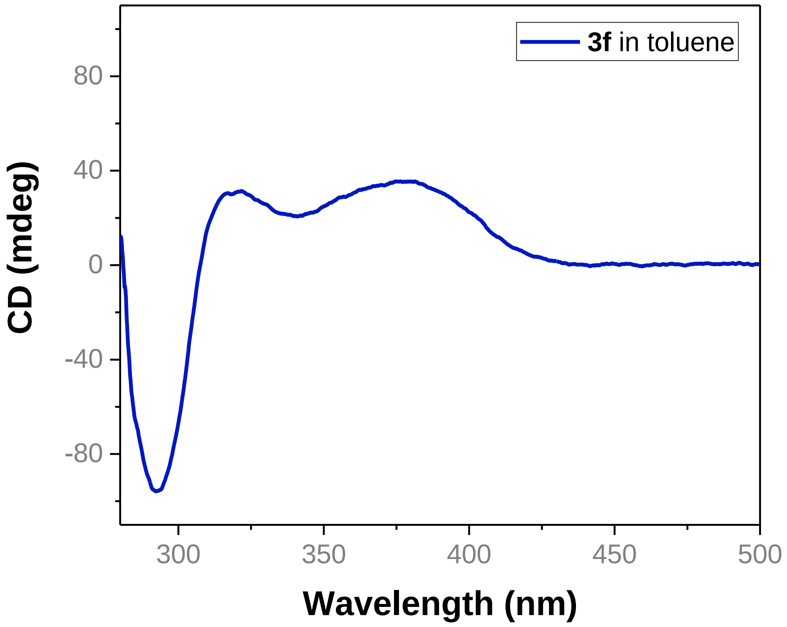

Figure S128. CD spectrum of **3h** in toluene at 25 ºC (ca. 5×10^{-5} M)

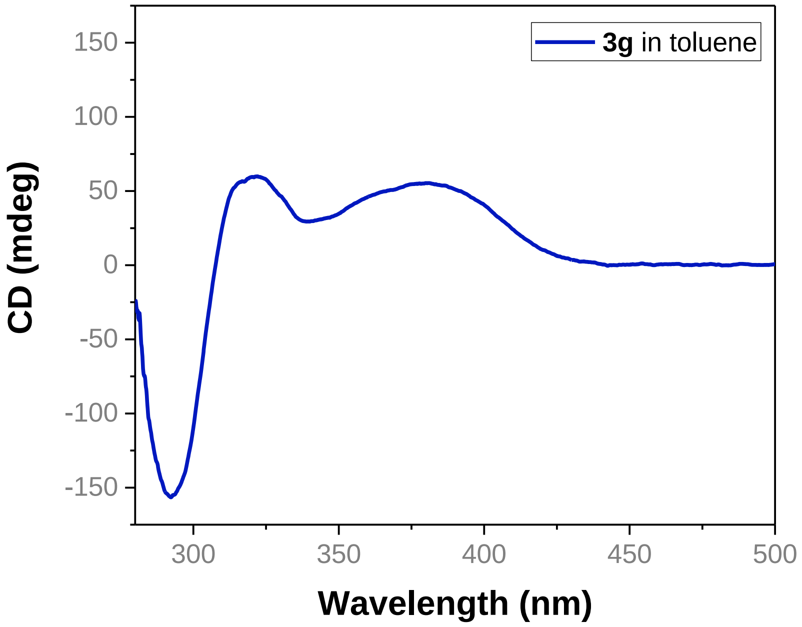

Figure S129. CD spectrum of **3g** in toluene at 25 ºC (ca. 5×10^{-5} M)

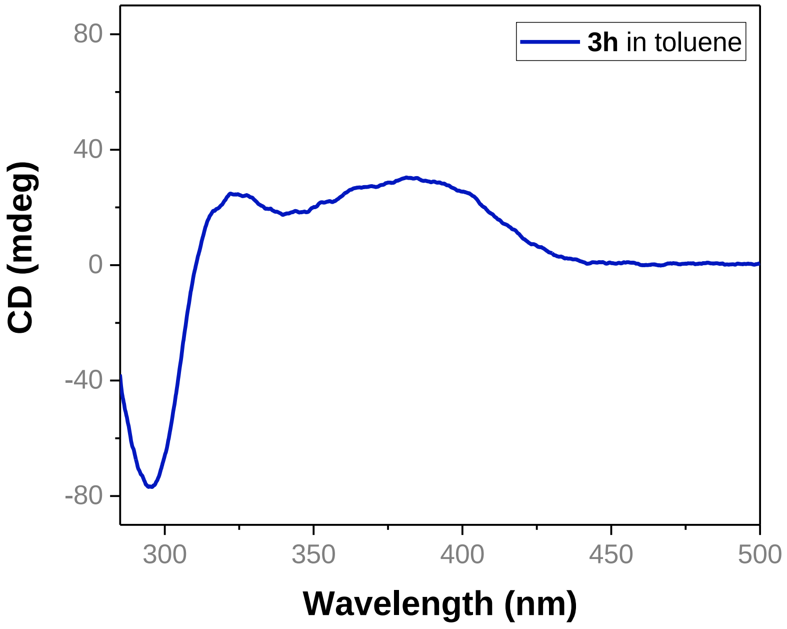

Figure S130. CD spectrum of **3h** in toluene at 25 ºC (ca. 5×10^{-5} M)

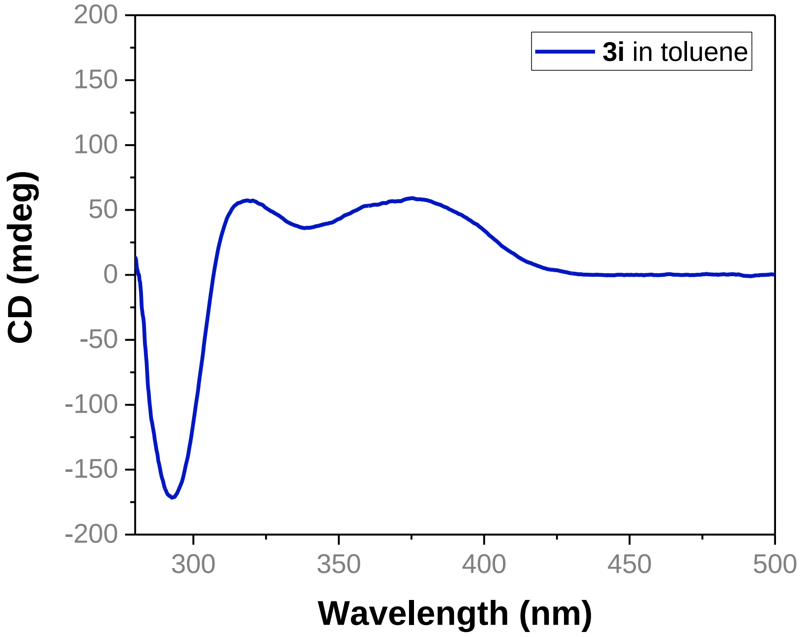

Figure S131. CD spectrum of **3i** in toluene at 25 °C (ca. 5×10^{-5} M)

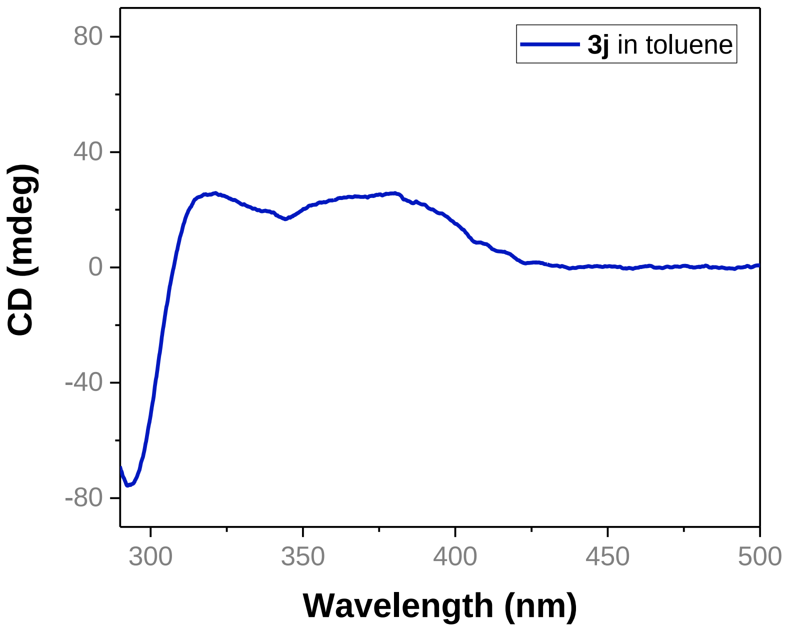

Figure S132. CD spectrum of **3j** in toluene at 25 °C (ca. 5×10^{-5} M)

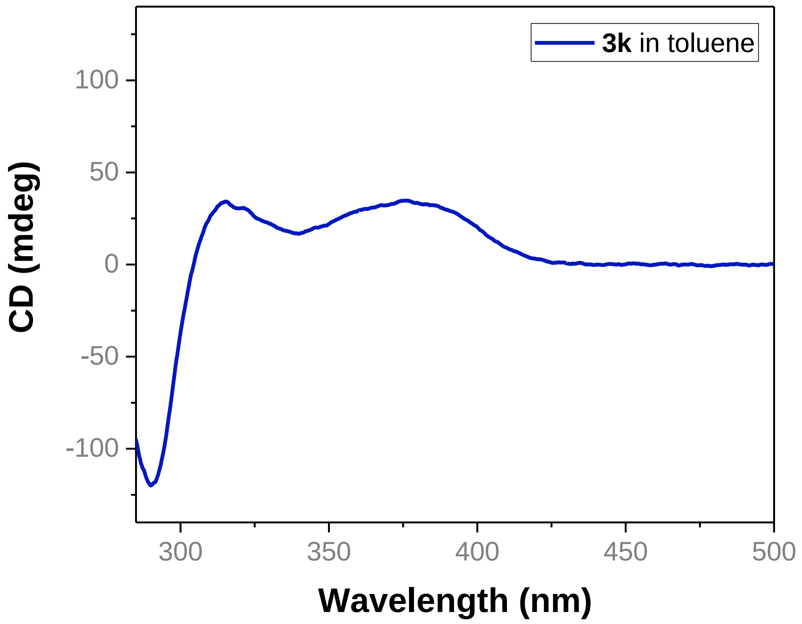

Figure S133. CD spectrum of **3k** in toluene at 25 °C (ca. 5×10^{-5} M)

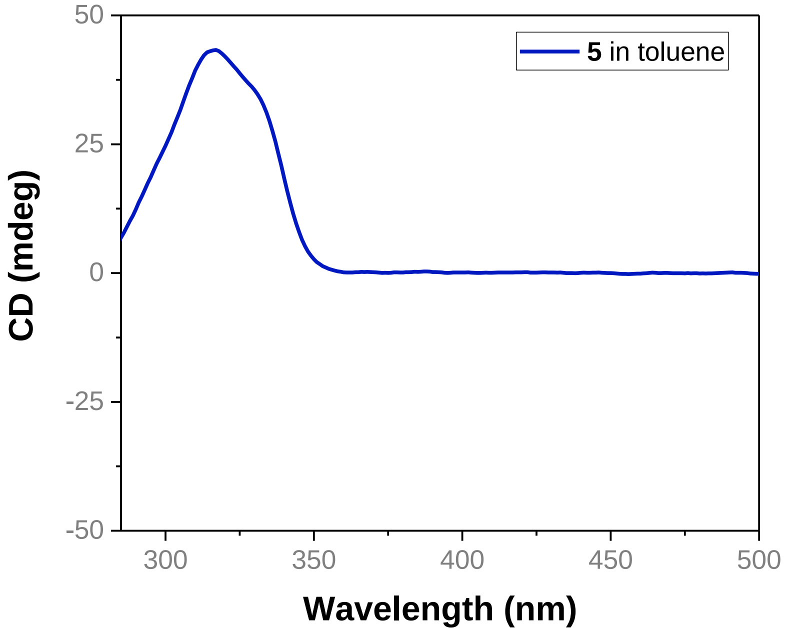

Figure S134. CD spectrum of **5** in toluene at 25 °C (ca. 5×10^{-5} M)

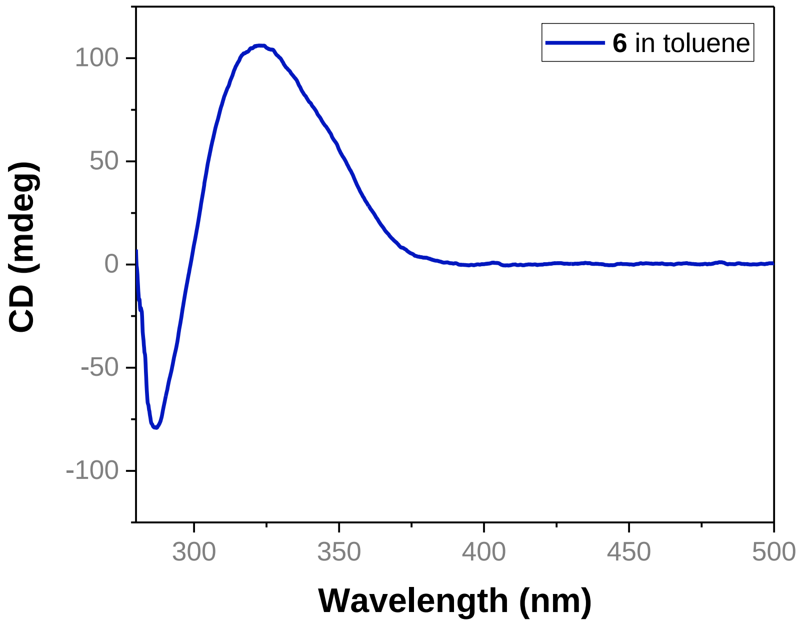

Figure S135. CD spectrum of **6** in toluene at 25 °C (ca. 4×10^{-5} M)

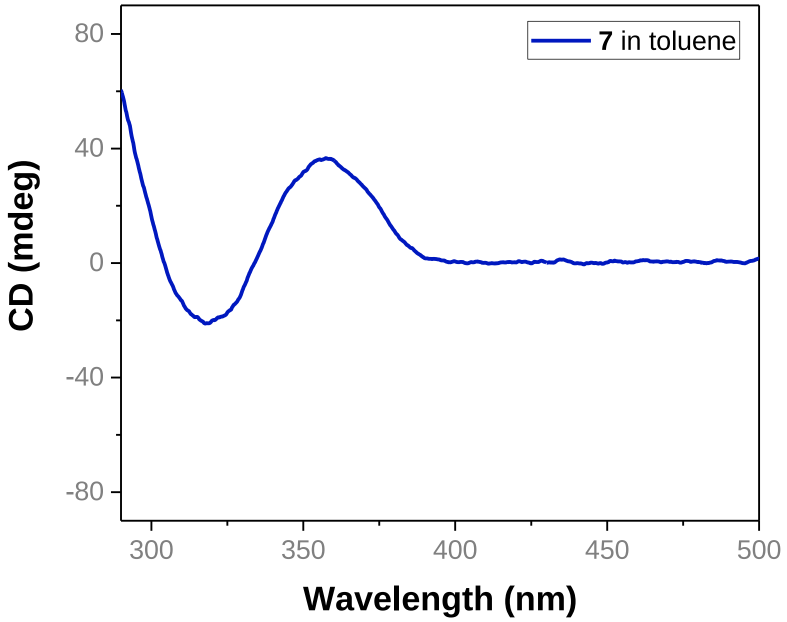

Figure S136. CD spectrum of **7** in toluene at 25 °C (ca. 4×10^{-5} M)

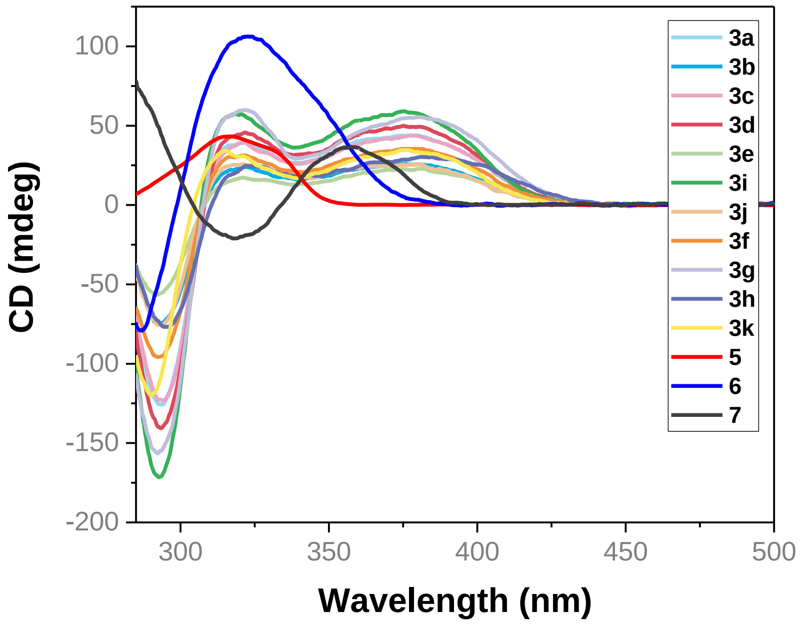

Figure S137. CD spectra of **3a-3k**, **5**, **6**, and **7** in toluene at 25 ºC

18. Copies of CPL spectra of 3a-3k, 5, 6, and 7

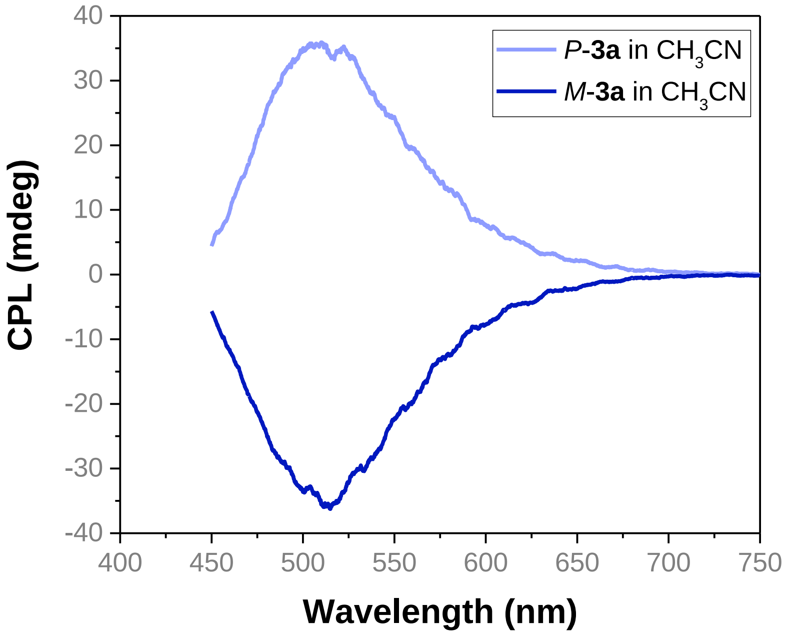

Figure S138. CPL spectra of *P*-**3a** and *M*-**3a** in CH_3CN at 25 °C (ca. 5×10^{-5} M)

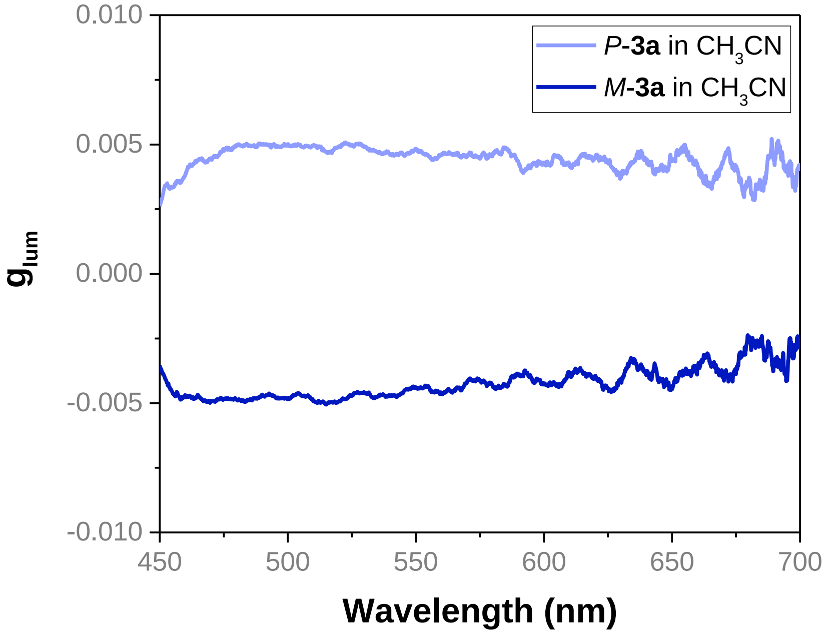

Figure S139. CPL (g_{lum}) of *P*-**3a** and *M*-**3a** in CH_3CN at 25 °C (ca. 5×10^{-5} M)

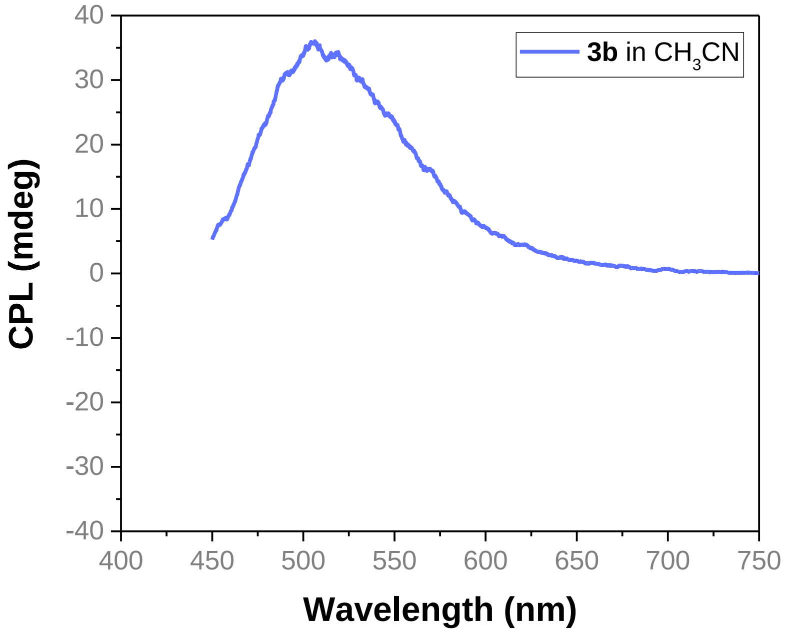

Figure S140. CPL spectrum of **3b** in CH_3CN at 25 °C (ca. 5×10^{-5} M)

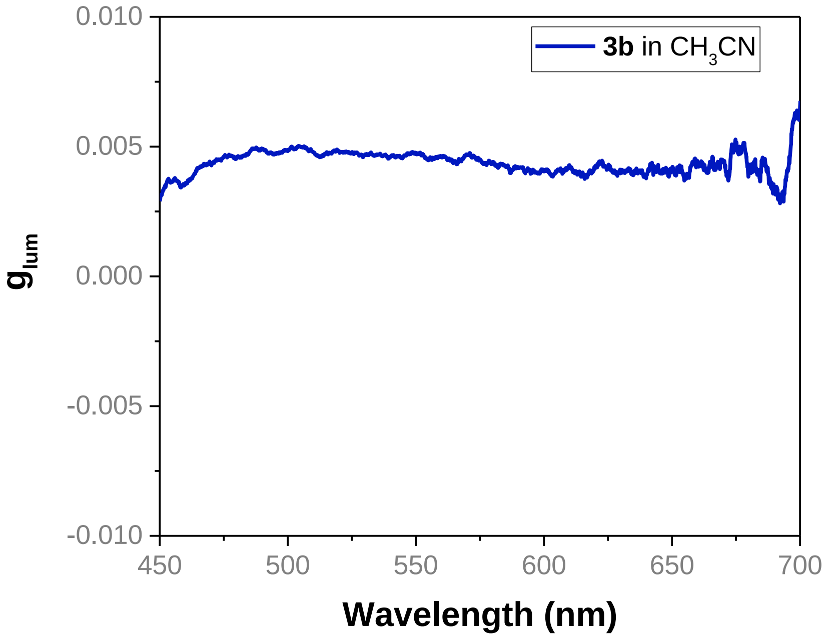

Figure S141. CPL (g_{lum}) of **3b** in CH_3CN at 25 °C (ca. 5×10^{-5} M)

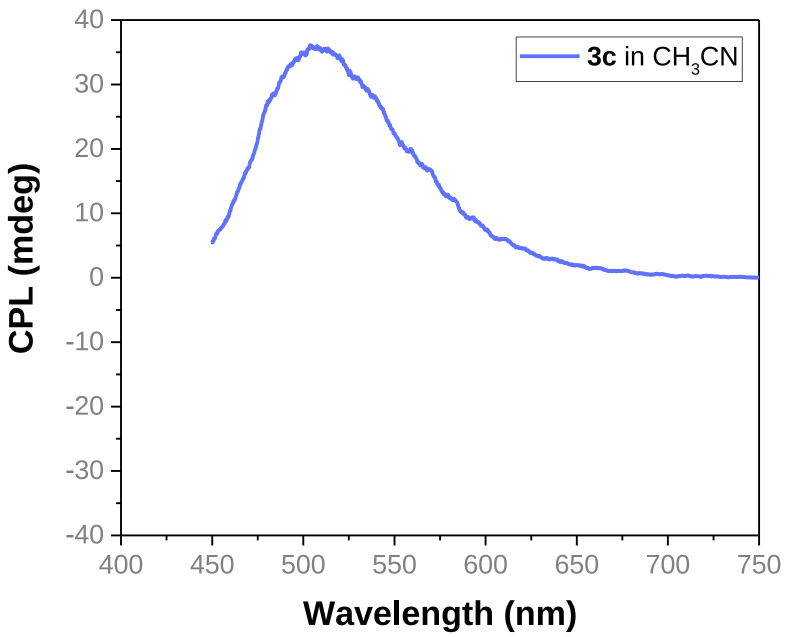

Figure S142. CPL spectrum of **3c** in CH_3CN at 25 °C (ca. 5×10^{-5} M)

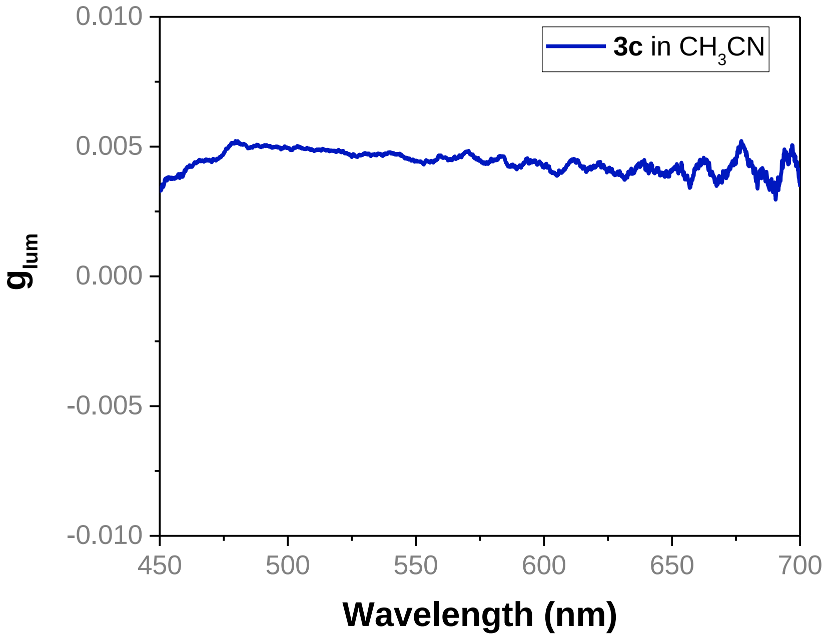

Figure S143. CPL (g_{lum}) of **3c** in CH_3CN at 25 °C (ca. 5×10^{-5} M)

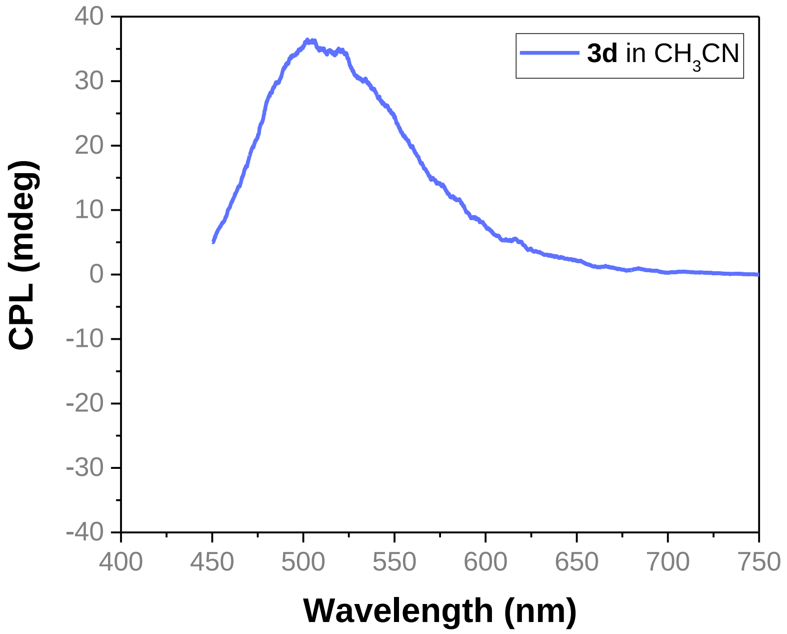

Figure S144. CPL spectrum of **3d** in CH_3CN at 25 ºC (ca. 5×10^{-5} M)

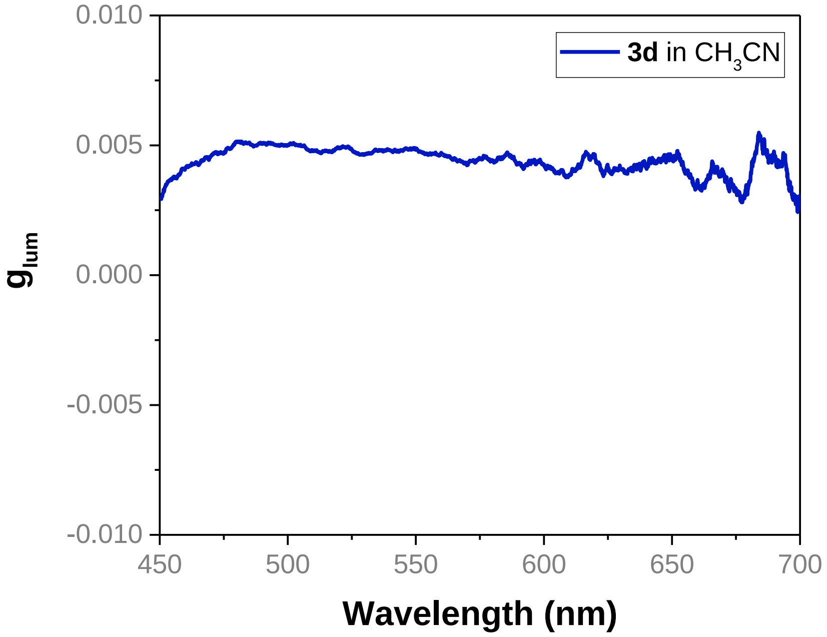

Figure S145. CPL (g_{lum}) of **3d** in CH_3CN at 25 ºC (ca. 5×10^{-5} M)

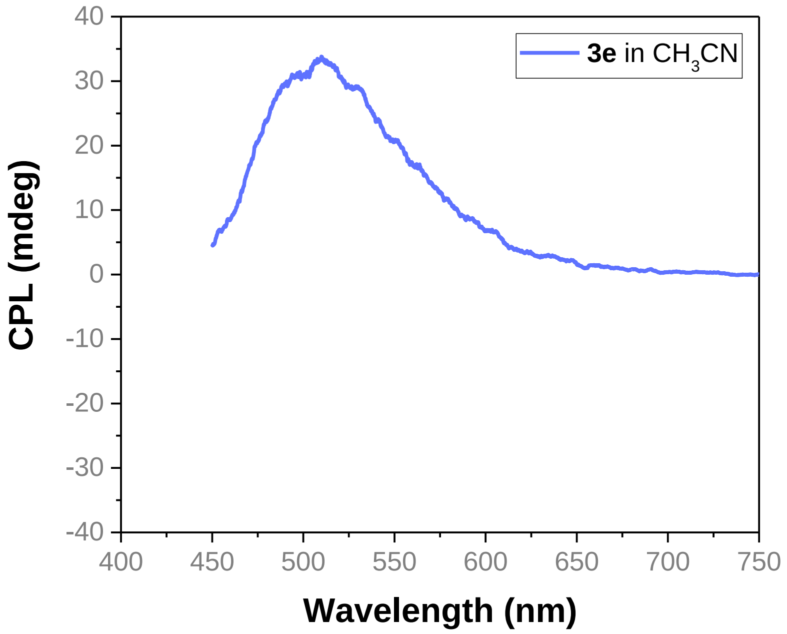

Figure S146. CPL spectrum of **3e** in CH_3CN at 25 °C (ca. 5×10^{-5} M)

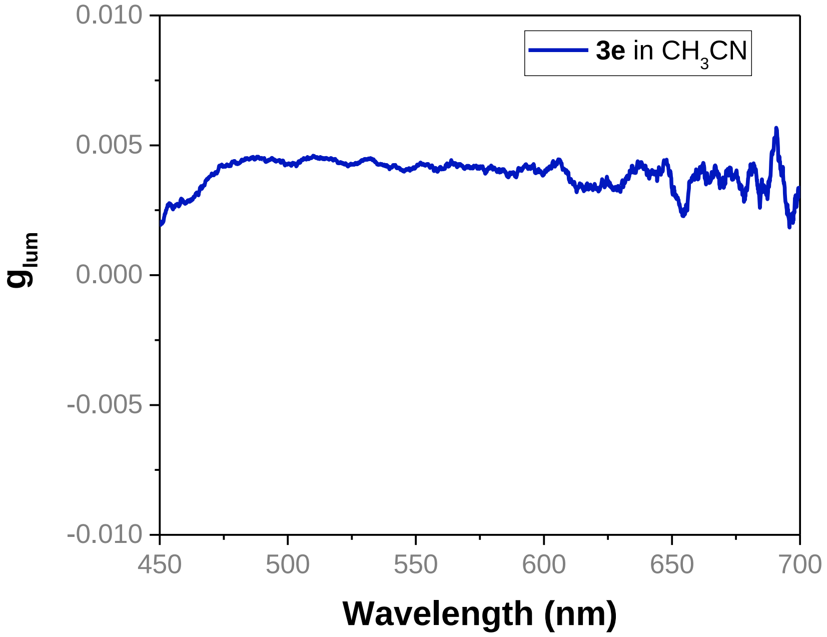

Figure S147. CPL (g_{lum}) of **3e** in CH_3CN at 25 °C (ca. 5×10^{-5} M)

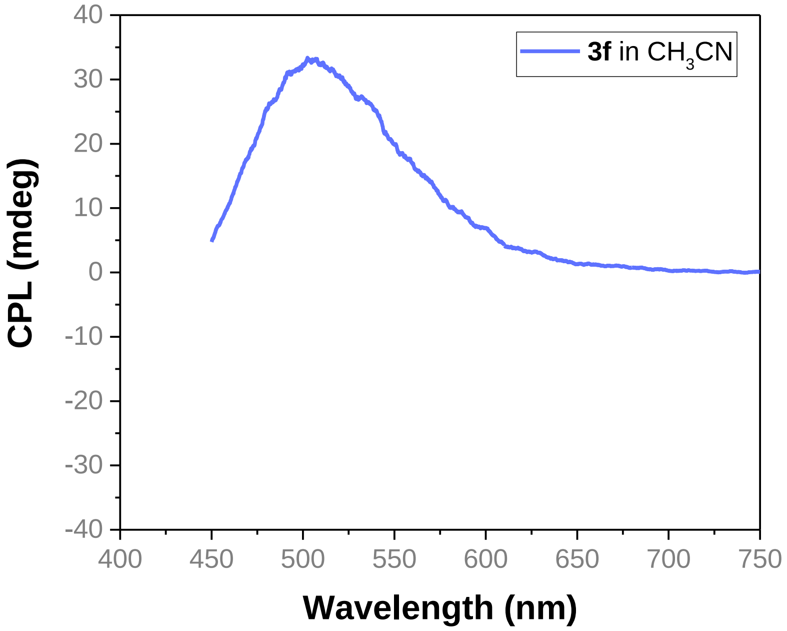

Figure S148. CPL spectrum of **3f** in CH_3CN at 25 °C (ca. $5×10^{-5}$ M)

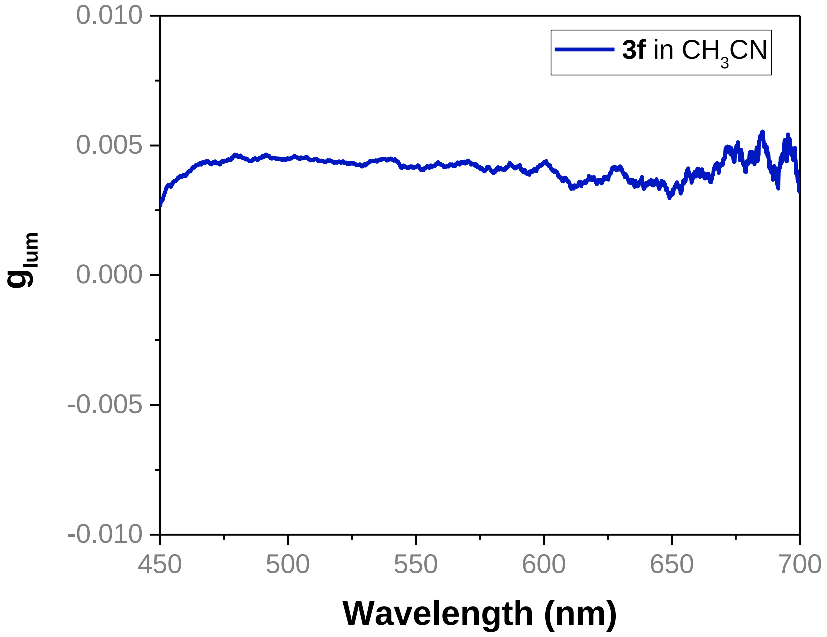

Figure S149. CPL (g_{lum}) of **3f** in CH_3CN at 25 °C (ca. $5×10^{-5}$ M)

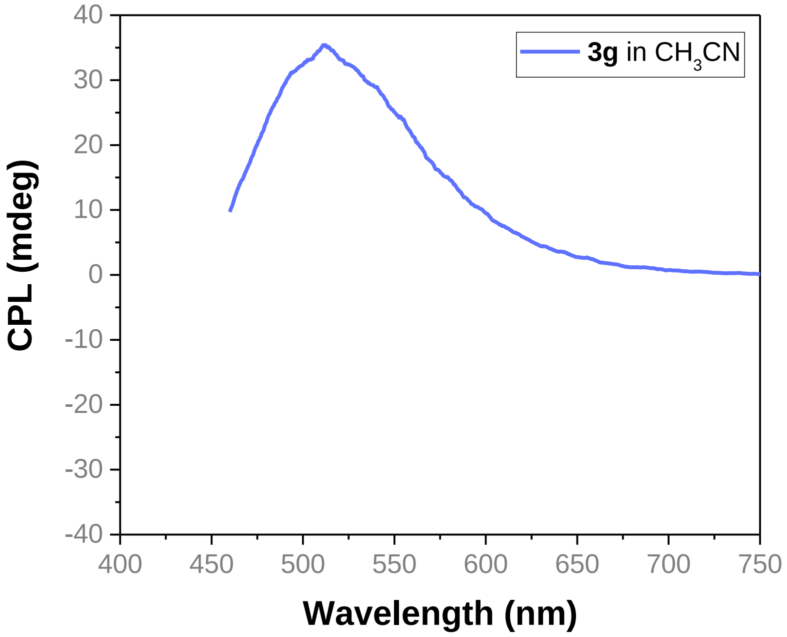

Figure S150. CPL spectrum of **3g** in CH_3CN at 25 °C (ca. 5×10^{-5} M)

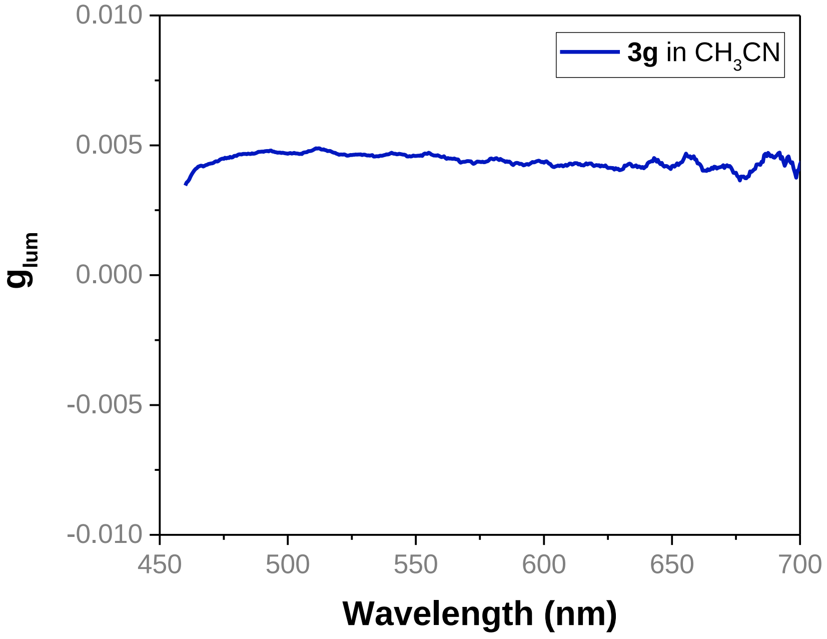

Figure S151. CPL (g_{lum}) of **3g** in CH_3CN at 25 °C (ca. 5×10^{-5} M)

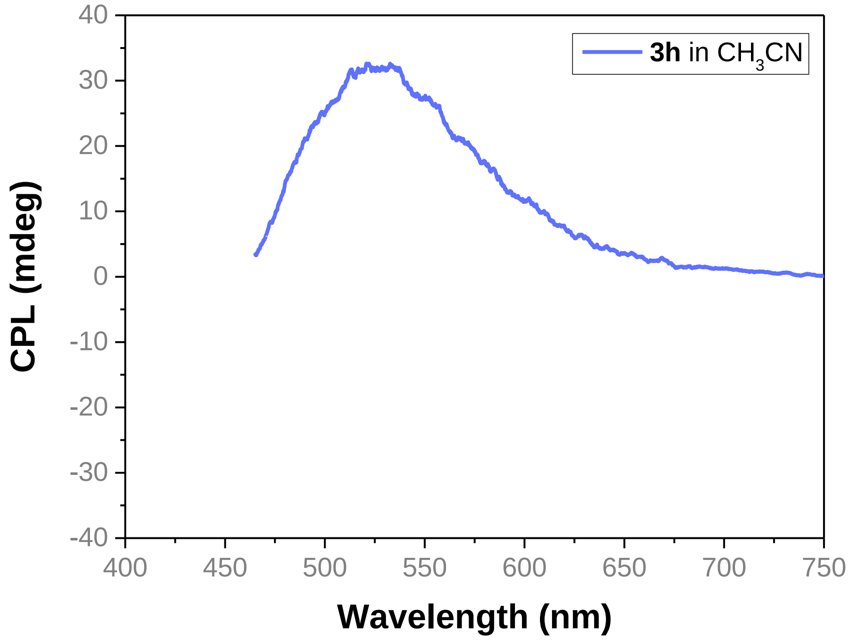

Figure S152. CPL spectrum of **3h** in CH_3CN at 25 ºC (ca. 5×10^{-5} M)

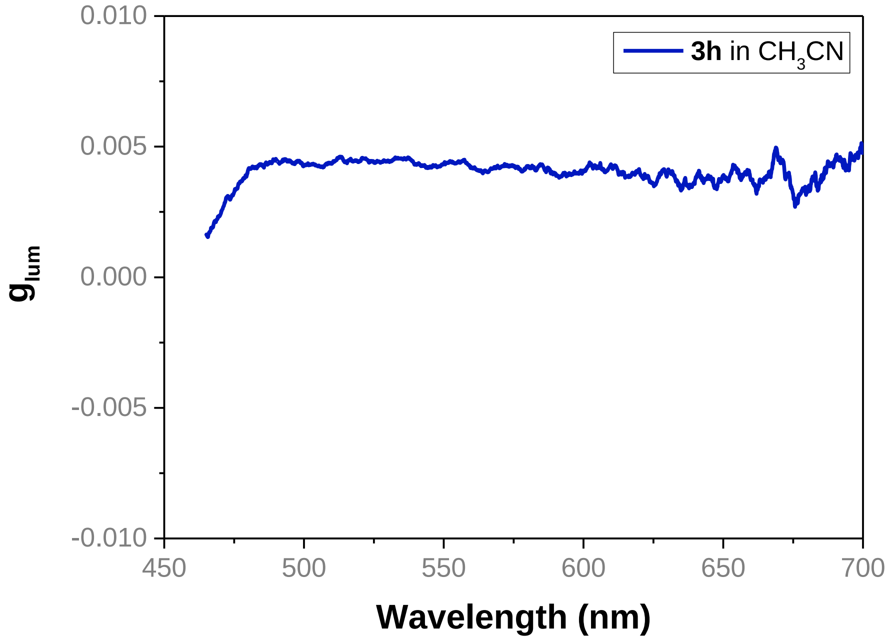

Figure S153. CPL (g_{lum}) of **3h** in CH_3CN at 25 ºC (ca. 5×10^{-5} M)

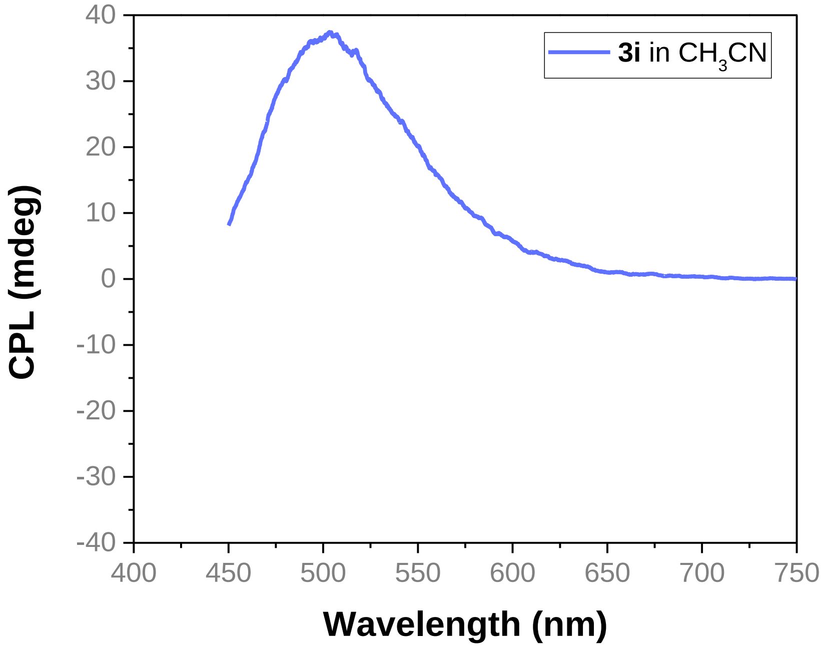

Figure S154. CPL spectrum of **3i** in CH_3CN at 25 °C (ca. 5×10^{-5} M)

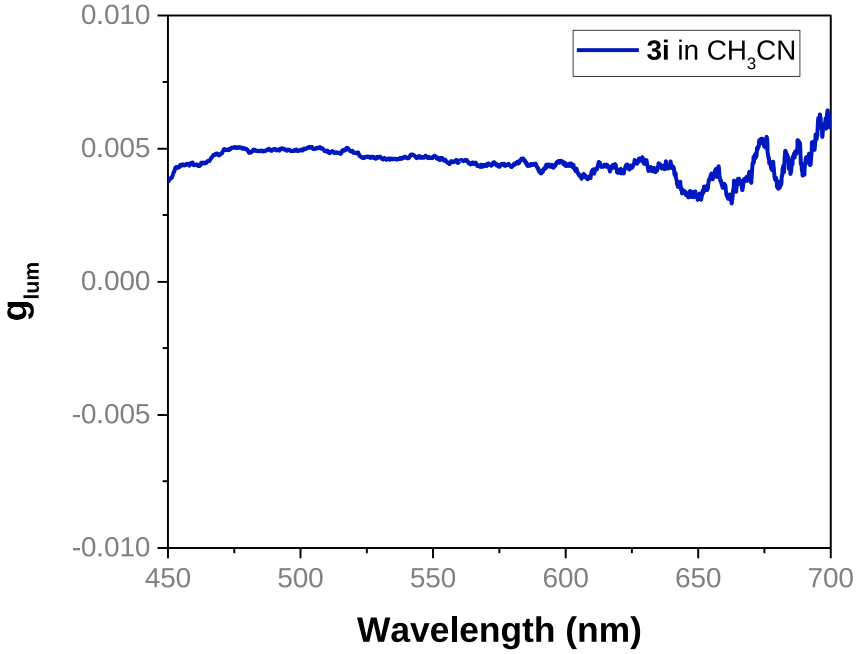

Figure S155. CPL (g_{lum}) of **3i** in CH_3CN at 25 °C (ca. 5×10^{-5} M)

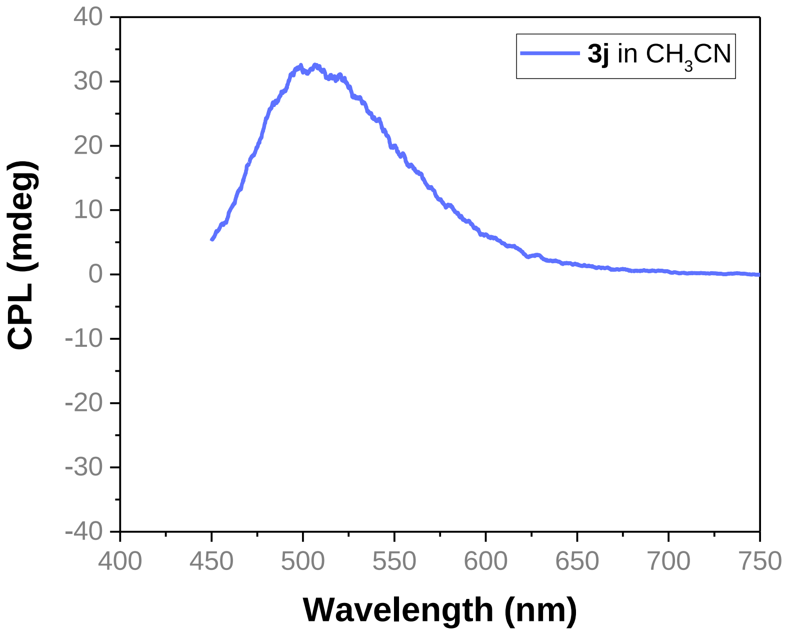

Figure S156. CPL spectrum of **3j** in CH_3CN at 25 °C (ca. 5×10^{-5} M)

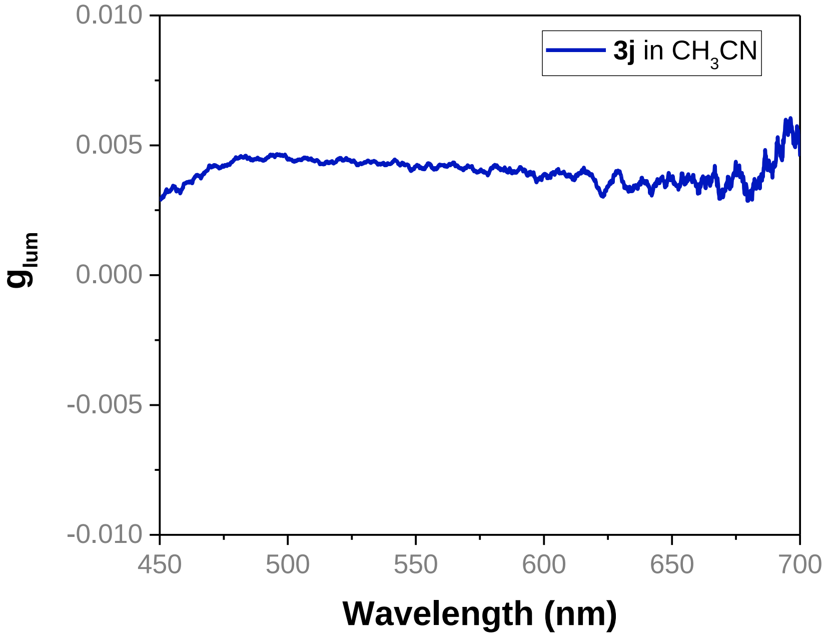

Figure S157. CPL (g_{lum}) of **3j** in CH_3CN at 25 °C (ca. 5×10^{-5} M)

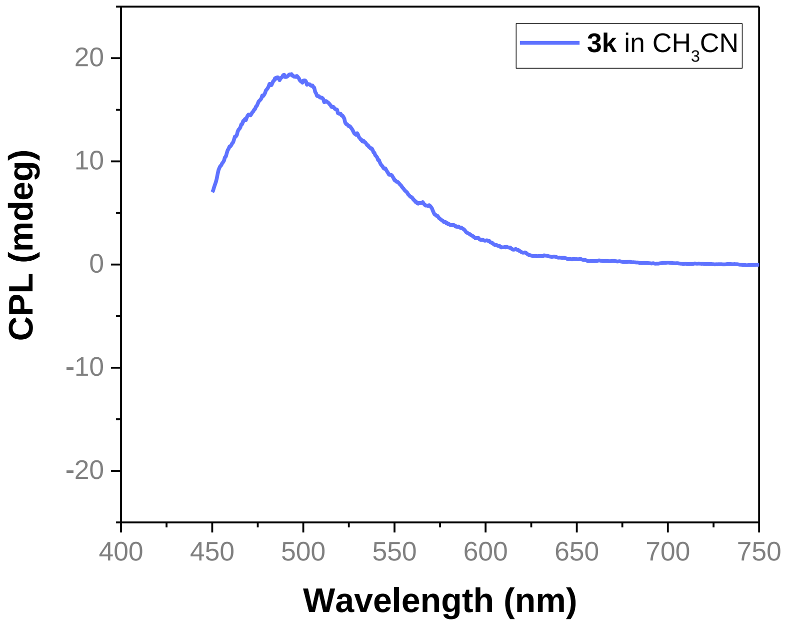

Figure S158. CPL spectrum of **3k** in CH_3CN at 25 °C (ca. 5×10^{-5} M)

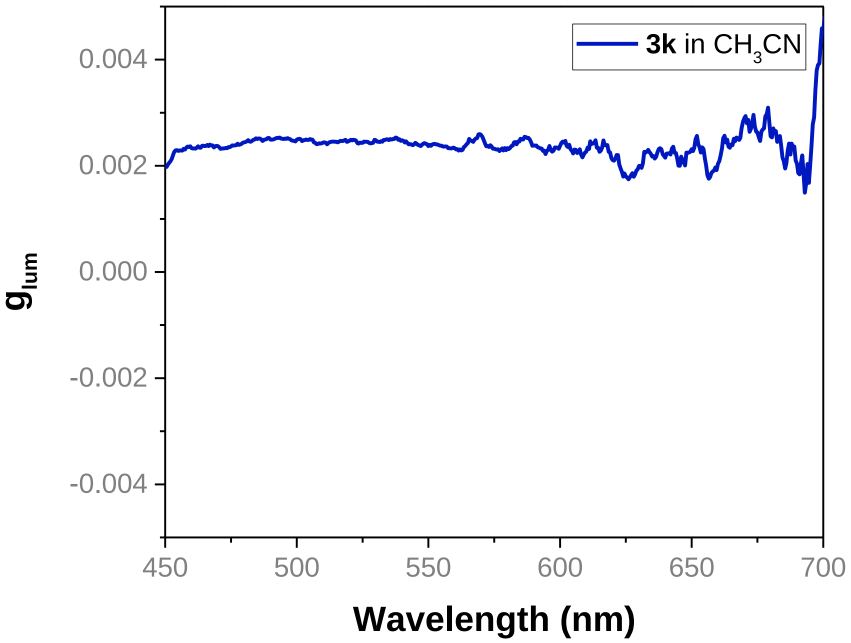

Figure S159. CPL (g_{lum}) of **3k** in CH_3CN at 25 °C (ca. 5×10^{-5} M)

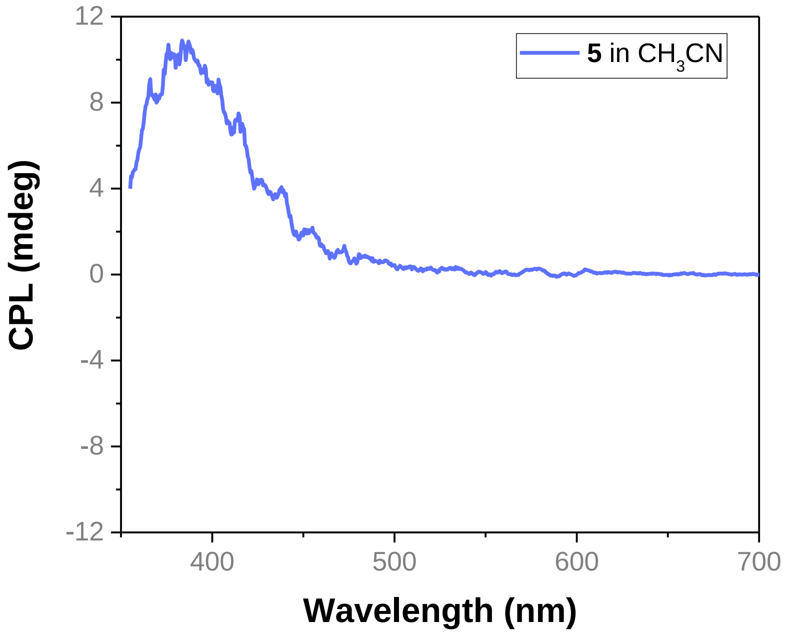

Figure S160. CPL spectrum of **5** in CH_3CN at 25 °C (ca. 5×10^{-5} M)

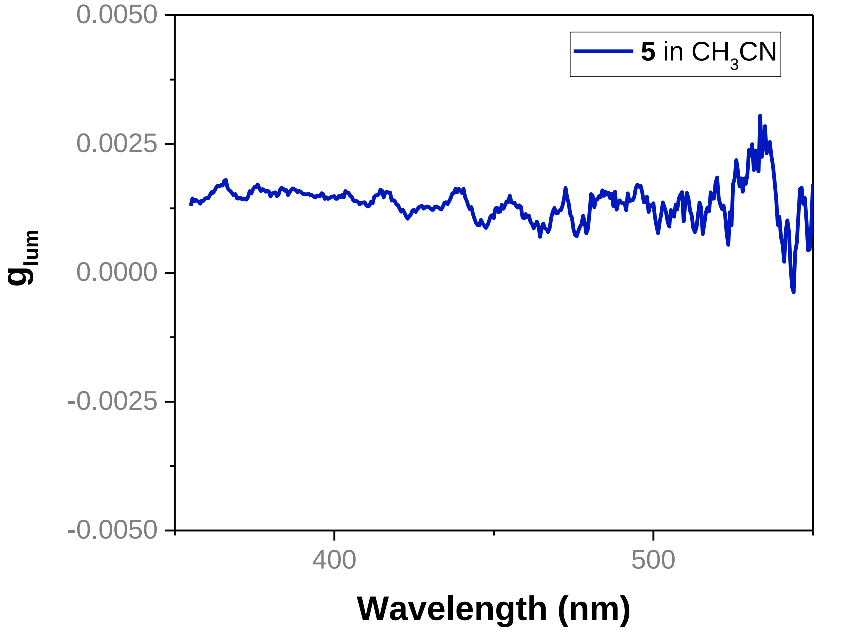

Figure S161. CPL (g_{lum}) of **5** in CH_3CN at 25 °C (ca. 5×10^{-5} M)

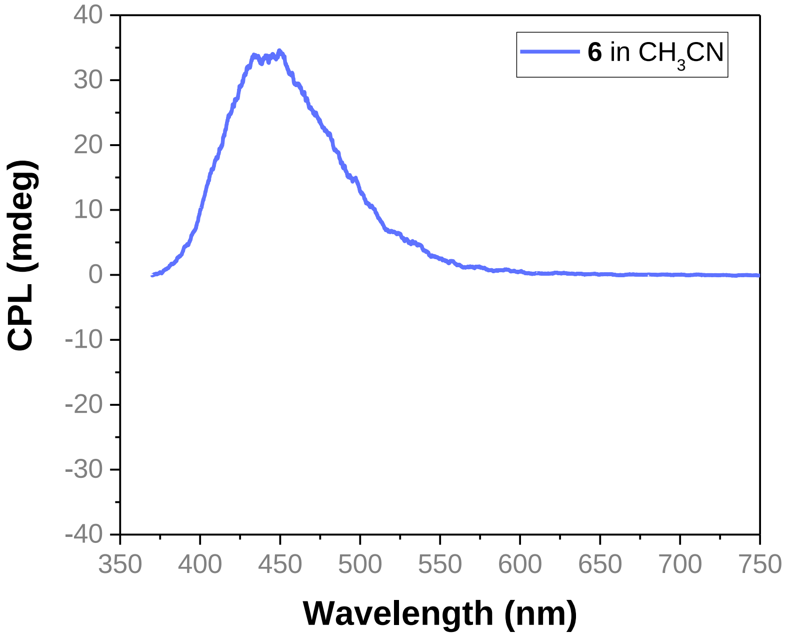

Figure S162. CPL spectrum of **6** in CH_3CN at 25 °C (ca. 5×10^{-5} M)

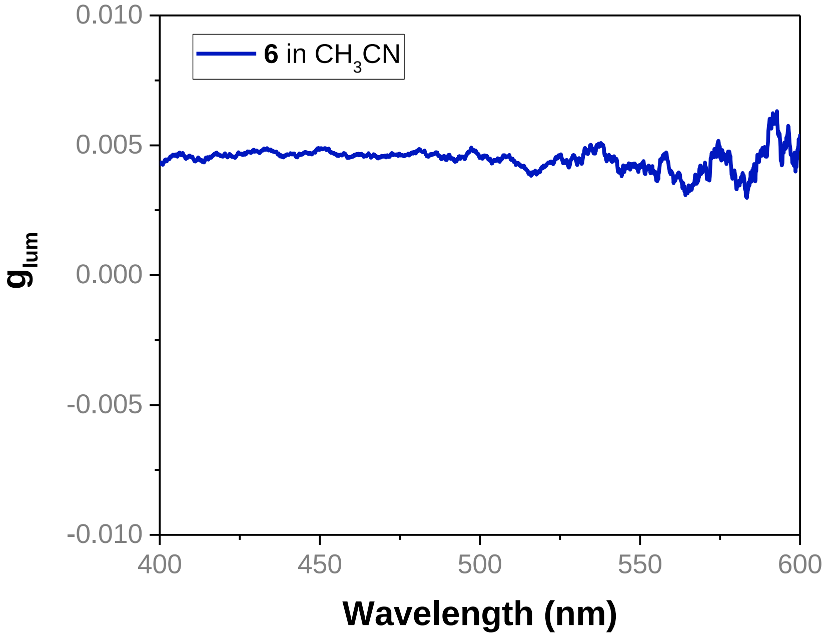

Figure S163. CPL (g_{lum}) of **6** in CH_3CN at 25 °C (ca. 5×10^{-5} M)

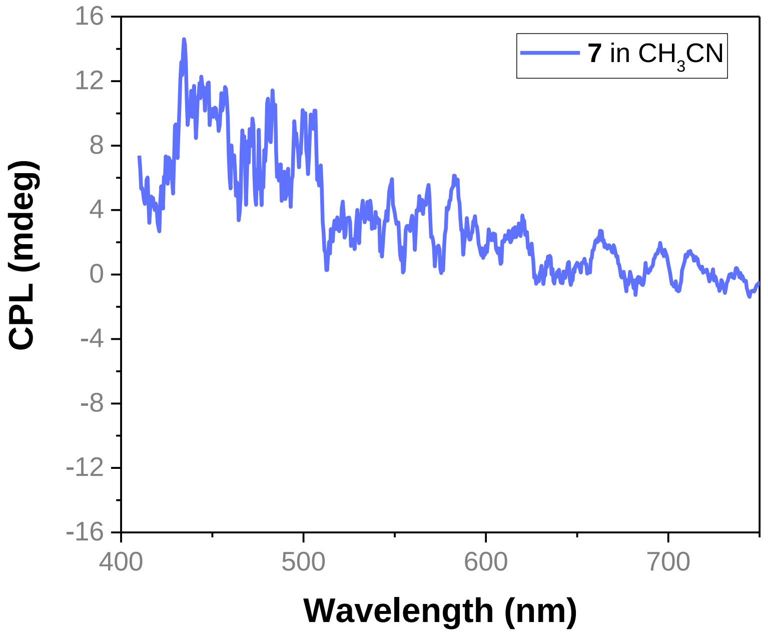

Figure S164. CPL spectrum of **7** in CH_3CN at 25 °C (ca. 5×10^{-5} M)

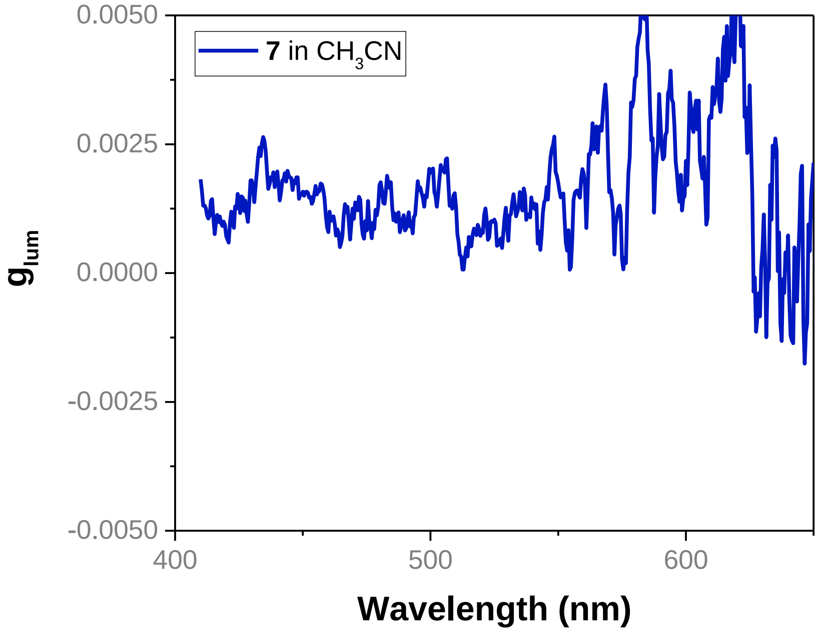

Figure S165. CPL (g_{lum}) of **7** in CH_3CN at 25 °C (ca. 5×10^{-5} M)

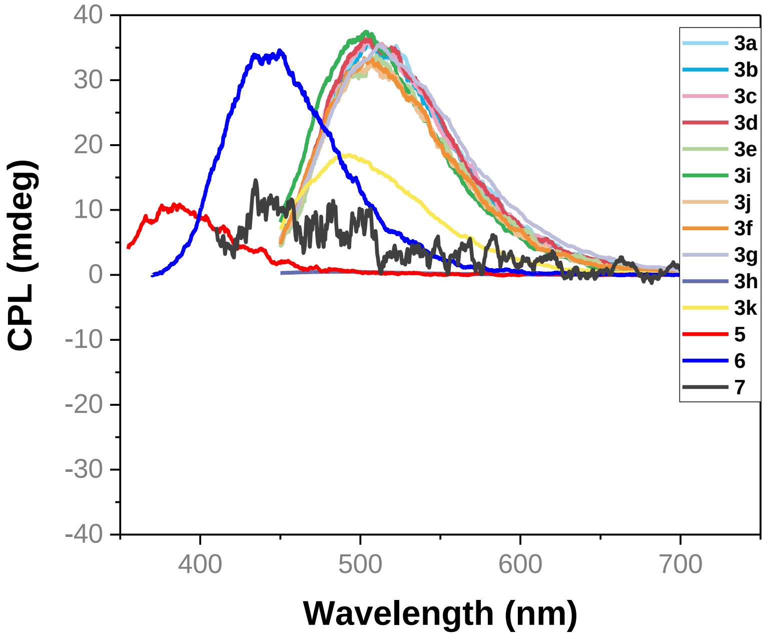

Figure S166. CPL spectra of **3a-3k**, **5**, **6**, and **7** in CH_3CN at 25 ºC

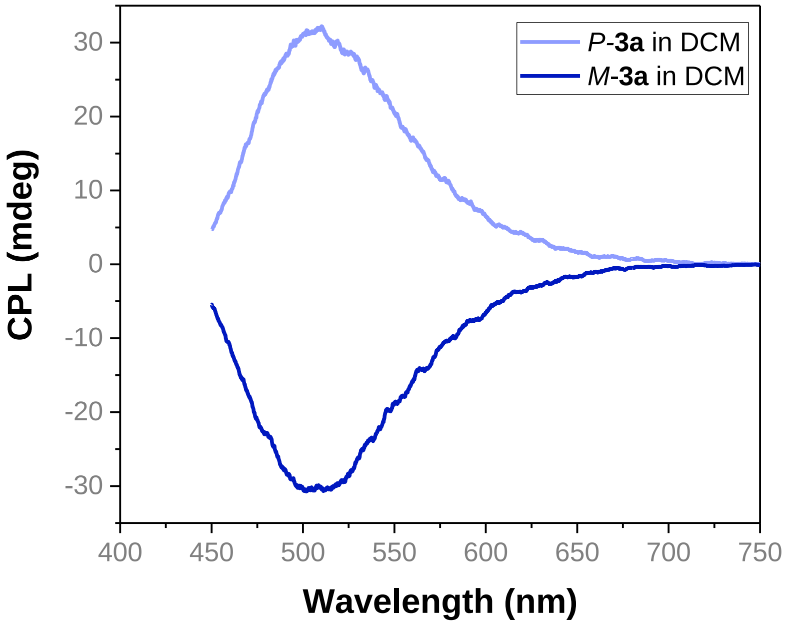

Figure S167. CPL spectra of *P*-**3a** and *M*-**3a** in DCM at 25 ºC (ca. 5×10^{-5} M)

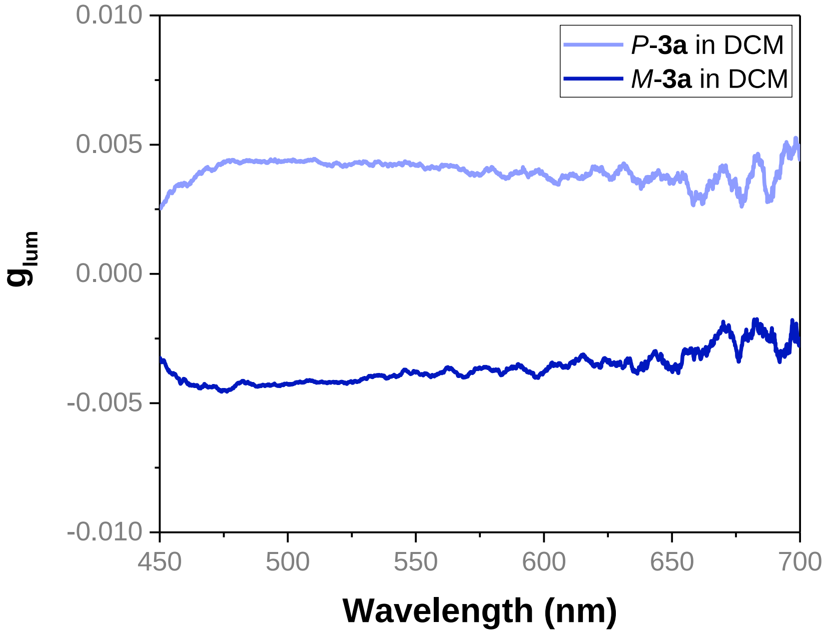

Figure S168. CPL (g_{lum}) of *P*-**3a** and *M*-**3a** in DCM at 25 ºC (ca. 5×10^{-5} M)

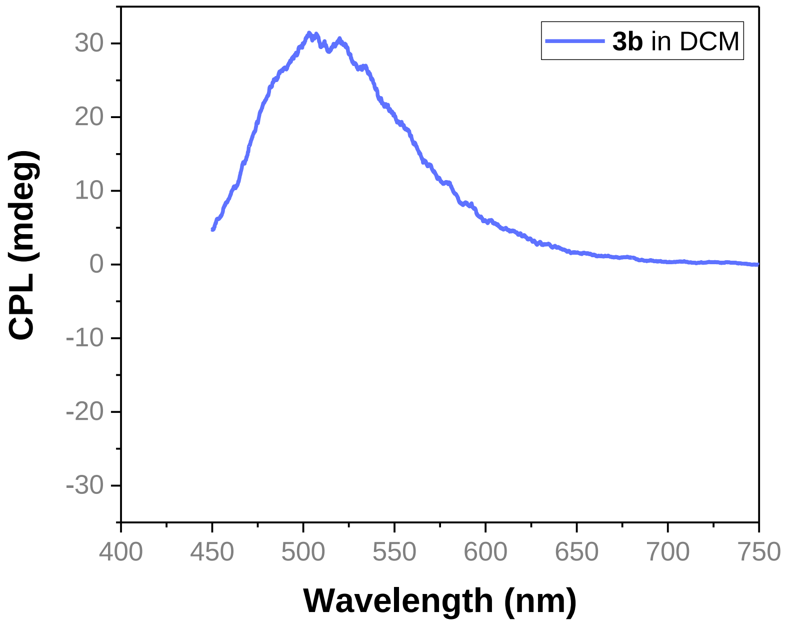

Figure S169. CPL spectrum of **3b** in DCM at 25 ºC (ca. 5×10^{-5} M)

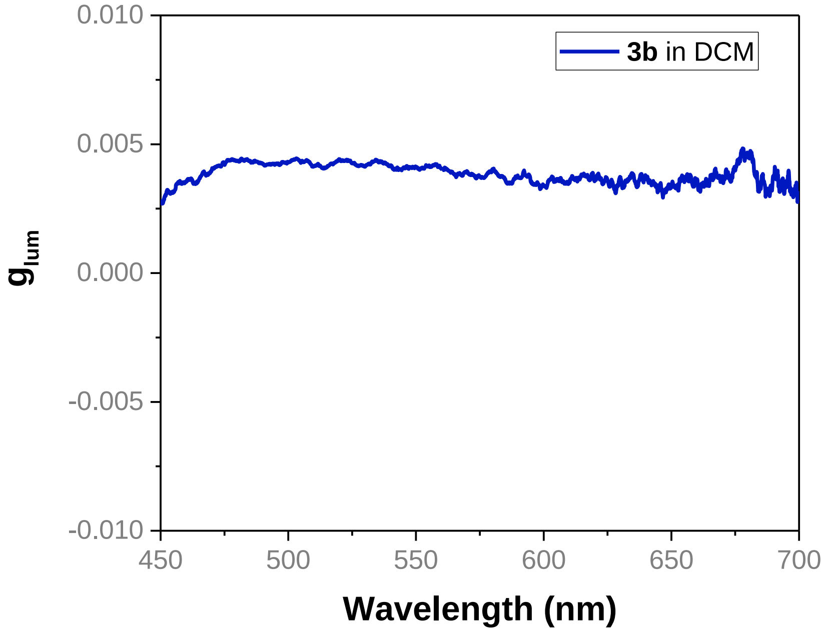

Figure S170. CPL (g_{lum}) of **3b** in DCM at 25 ºC (ca. 5×10^{-5} M)

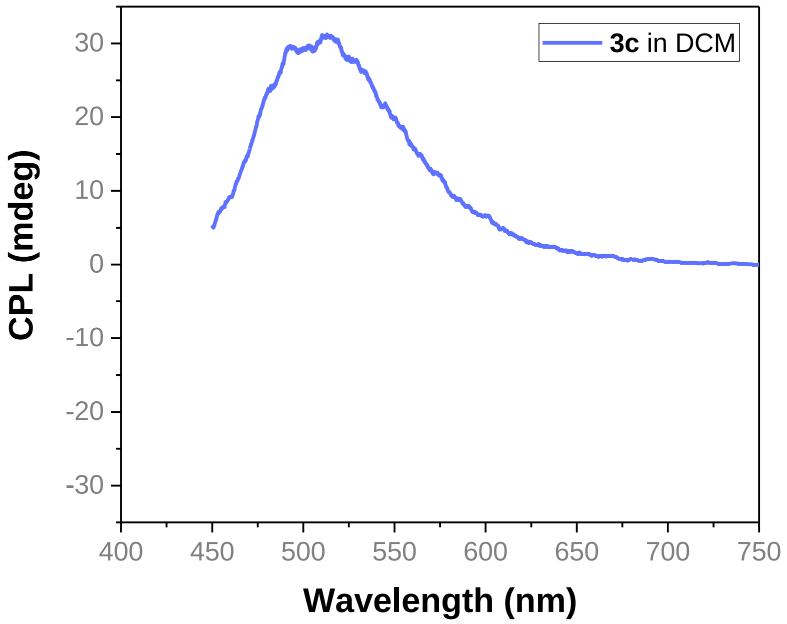

Figure S171. CPL spectrum of **3c** in DCM at 25 ºC (ca. 5×10^{-5} M)

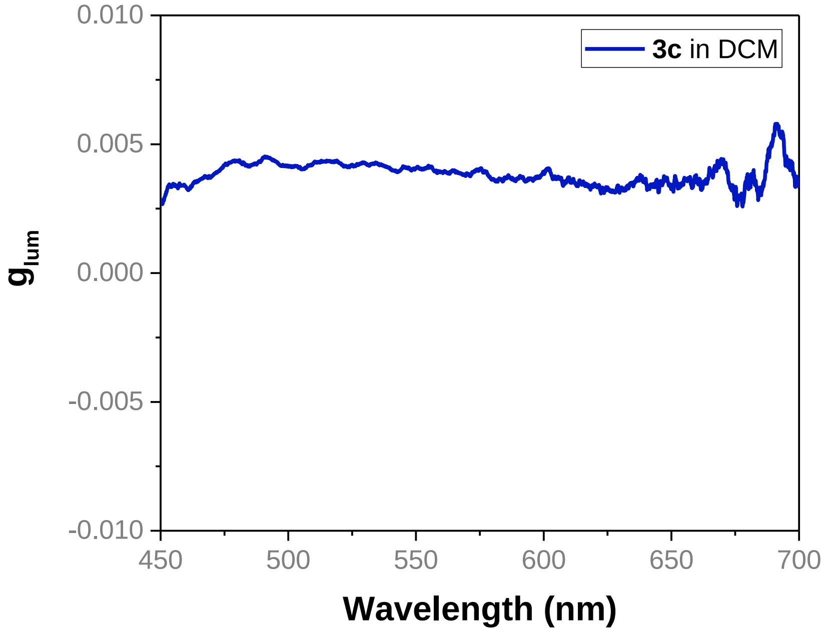

Figure S172. CPL (g_{lum}) of **3c** in DCM at 25 ºC (ca. 5×10^{-5} M)

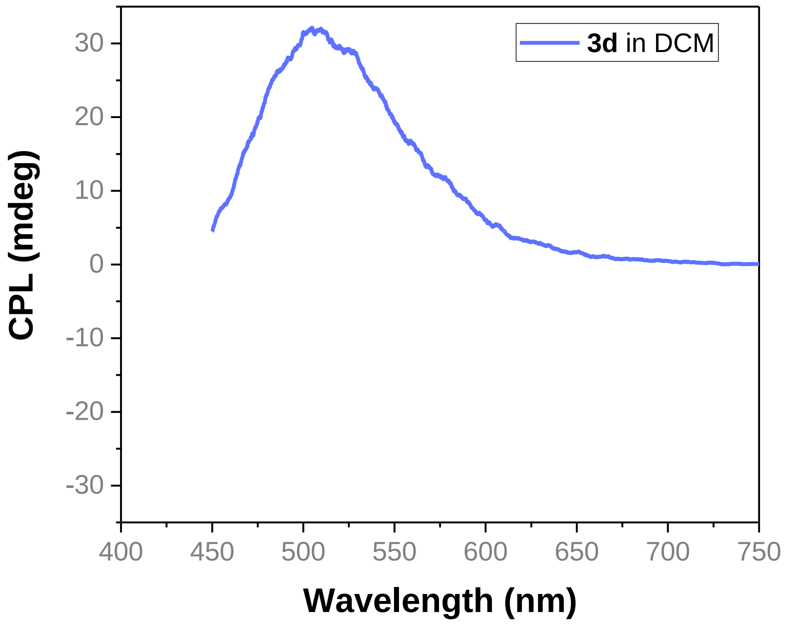

Figure S173. CPL spectrum of **3d** in DCM at 25 ºC (ca. 5×10^{-5} M)

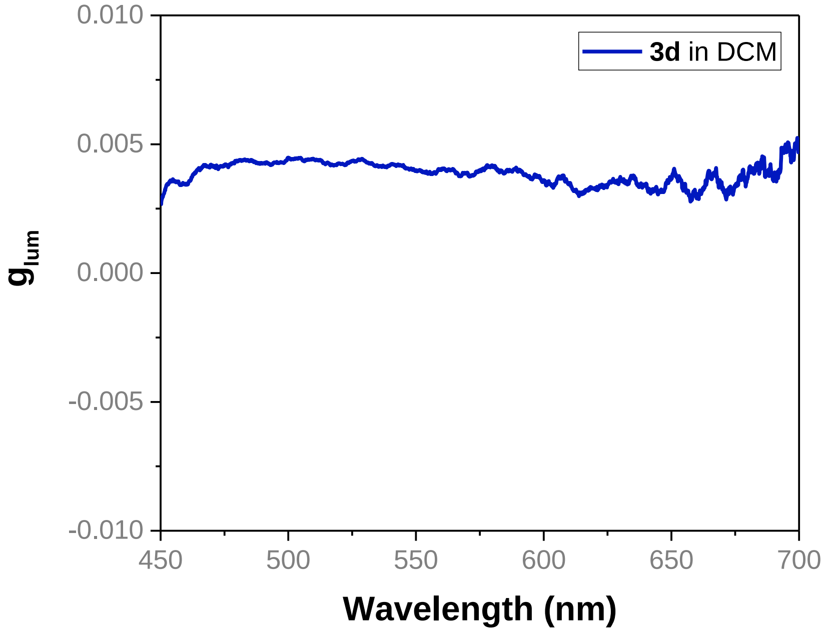

Figure S174. CPL (g_{lum}) of **3d** in DCM at 25 ºC (ca. 5×10^{-5} M)

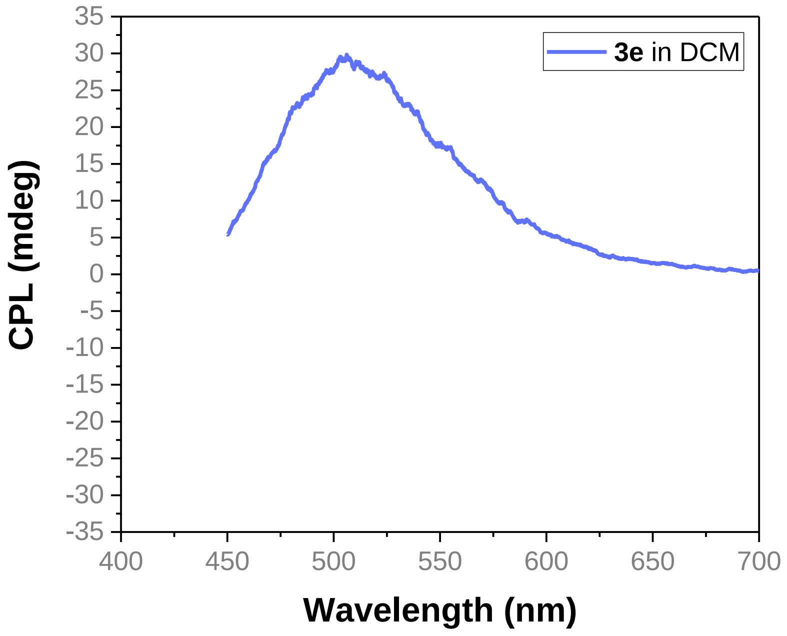

Figure S175. CPL spectrum of **3e** in DCM at 25 ºC (ca. 5×10^{-5} M)

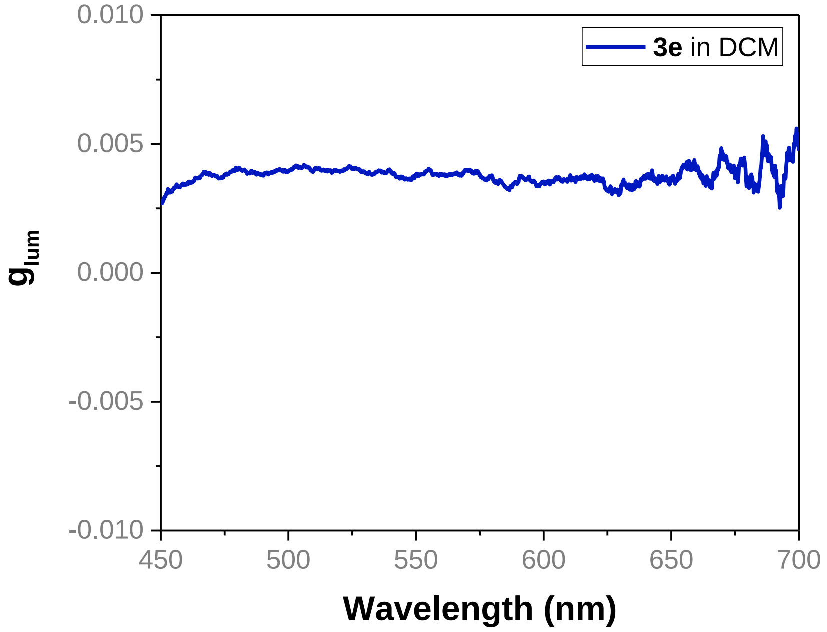

Figure S176. CPL (g_{lum}) of **3e** in DCM at 25 ºC (ca. 5×10^{-5} M)

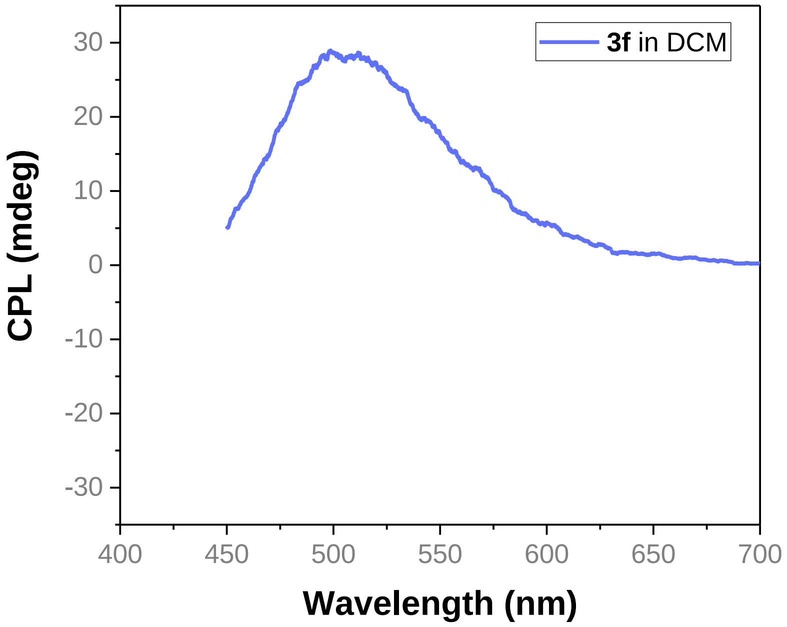

Figure S177. CPL spectrum of **3f** in DCM at 25 ºC (ca. 5×10^{-5} M)

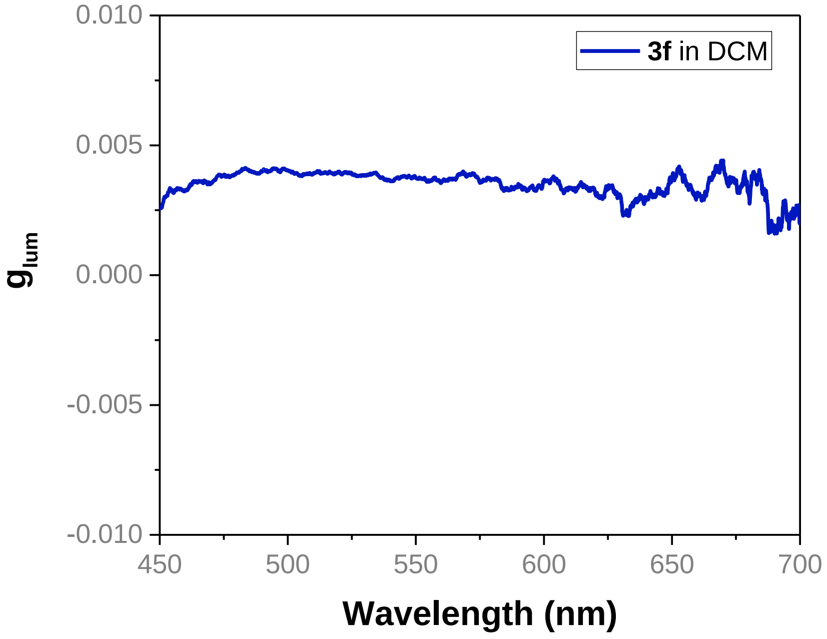

Figure S178. CPL (g_{lum}) of **3f** in DCM at 25 ºC (ca. 5×10^{-5} M)

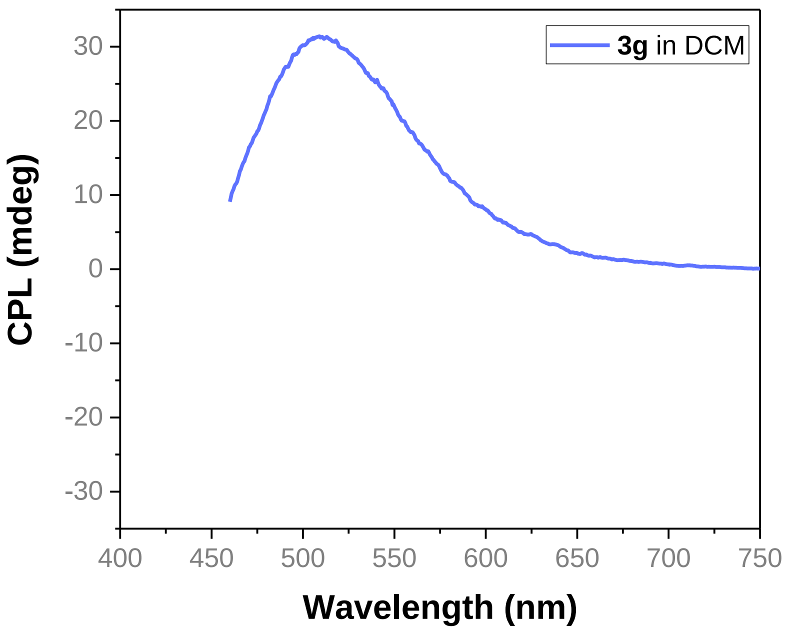

Figure S179. CPL spectrum of **3g** in DCM at 25 ºC (ca. 5×10^{-5} M)

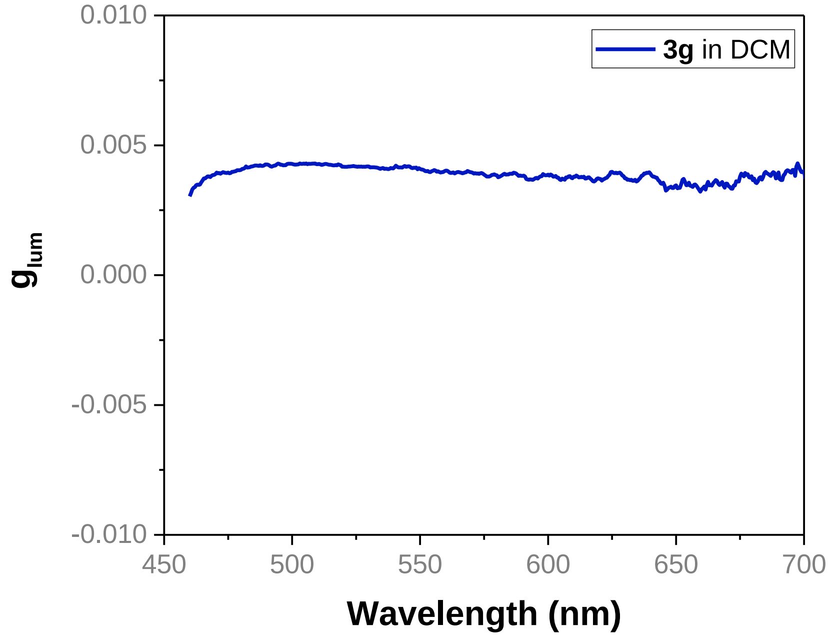

Figure S180. CPL (g_{lum}) of **3g** in DCM at 25 ºC (ca. 5×10^{-5} M)

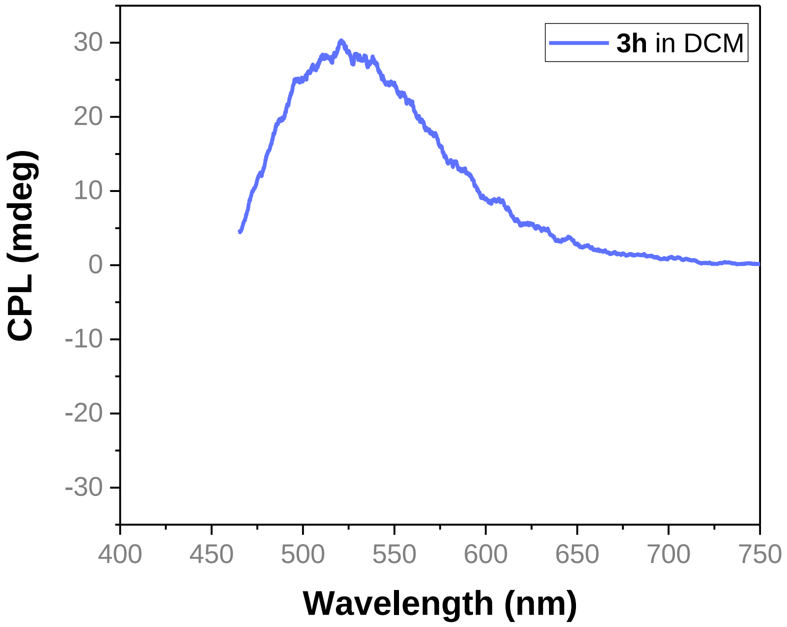

Figure S181. CPL spectrum of **3h** in DCM at 25 ºC (ca. 5×10^{-5} M)

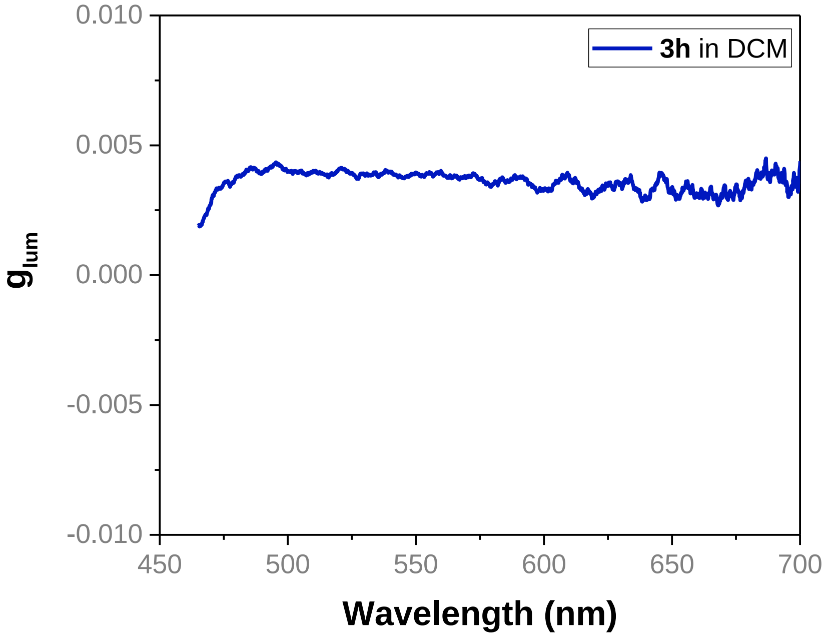

Figure S182. CPL (g_{lum}) of **3h** in DCM at 25 ºC (ca. 5×10^{-5} M)

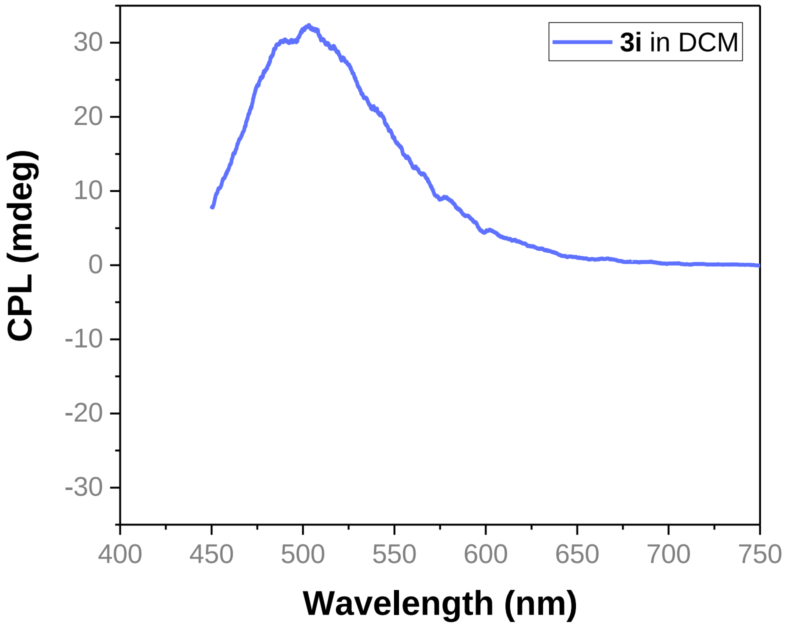

Figure S183. CPL spectrum of **3i** in DCM at 25 ºC (ca. 5×10^{-5} M)

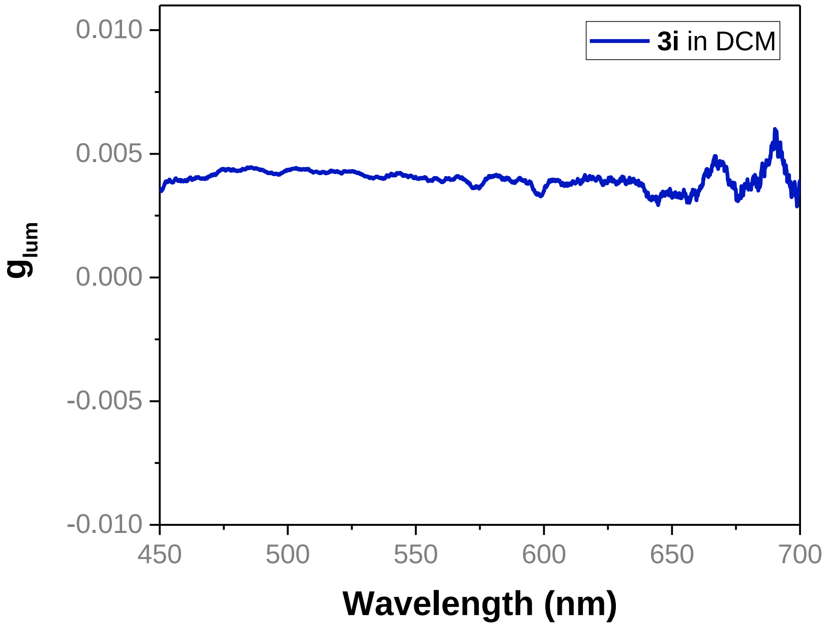

Figure S184. CPL (g_{lum}) of **3i** in DCM at 25 ºC (ca. 5×10^{-5} M)

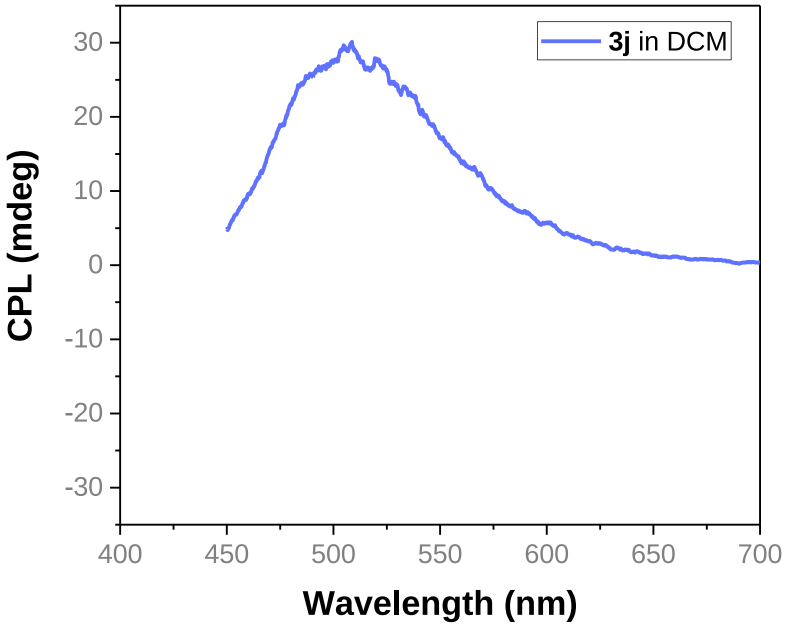

Figure S185. CPL spectrum of **3j** in DCM at 25 ºC (ca. 5×10^{-5} M)

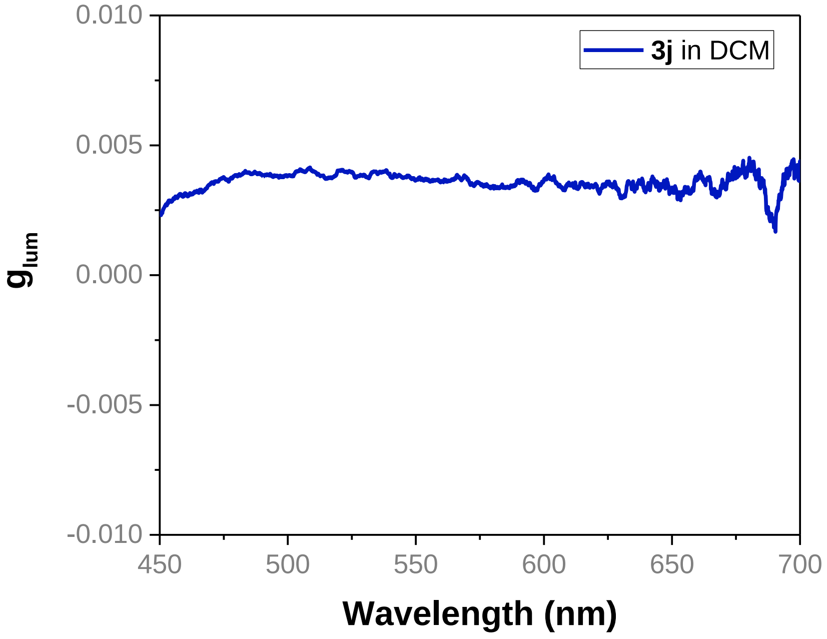

Figure S186. CPL (g_{lum}) of **3j** in DCM at 25 ºC (ca. 5×10^{-5} M)

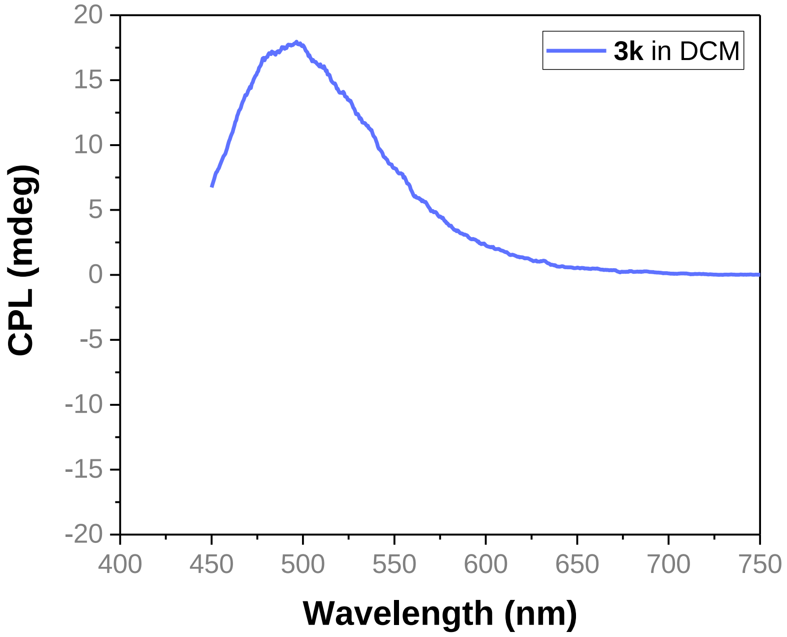

Figure S187. CPL spectrum of **3k** in DCM at 25 °C (ca. 5×10^{-5} M)

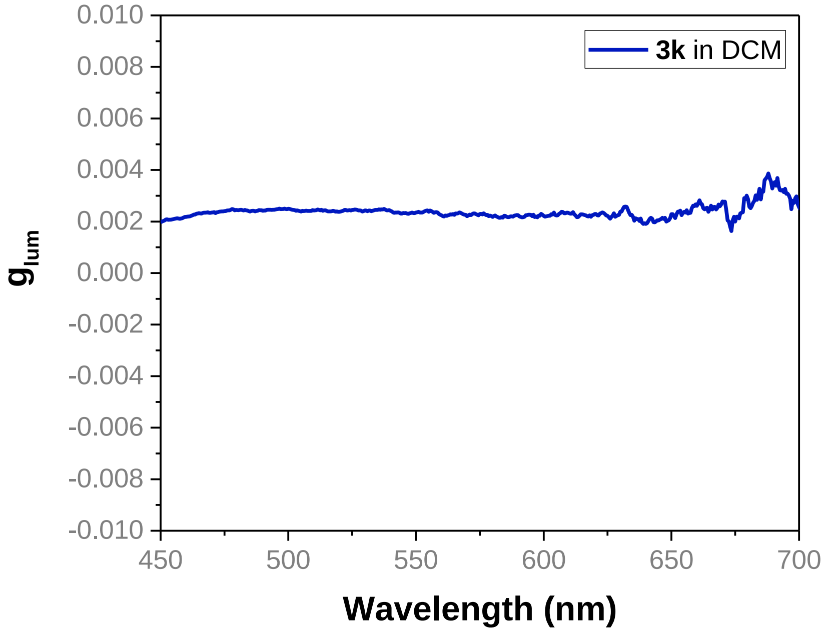

Figure S188. CPL (g_{lum}) of **3k** in DCM at 25 °C (ca. 5×10^{-5} M)

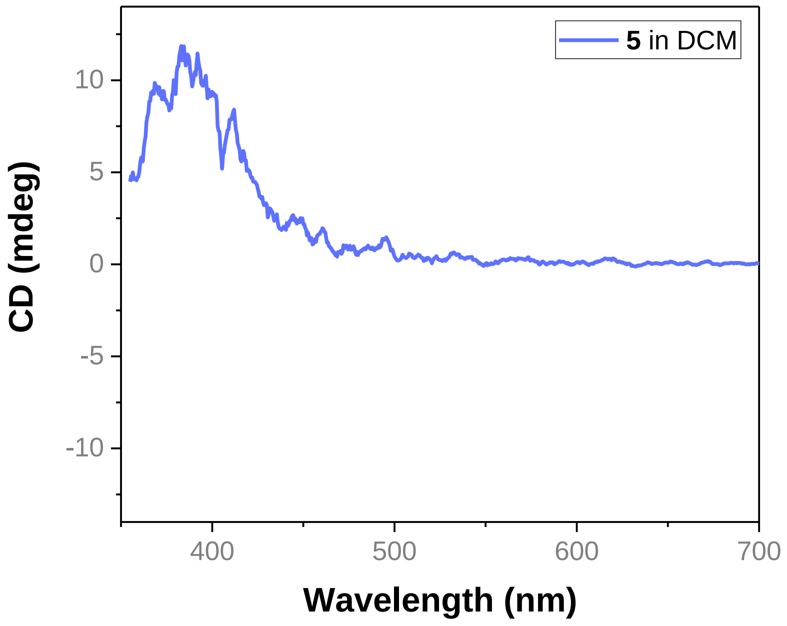

Figure S189. CPL spectrum of **5** in DCM at 25 °C (ca. 5×10^{-5} M)

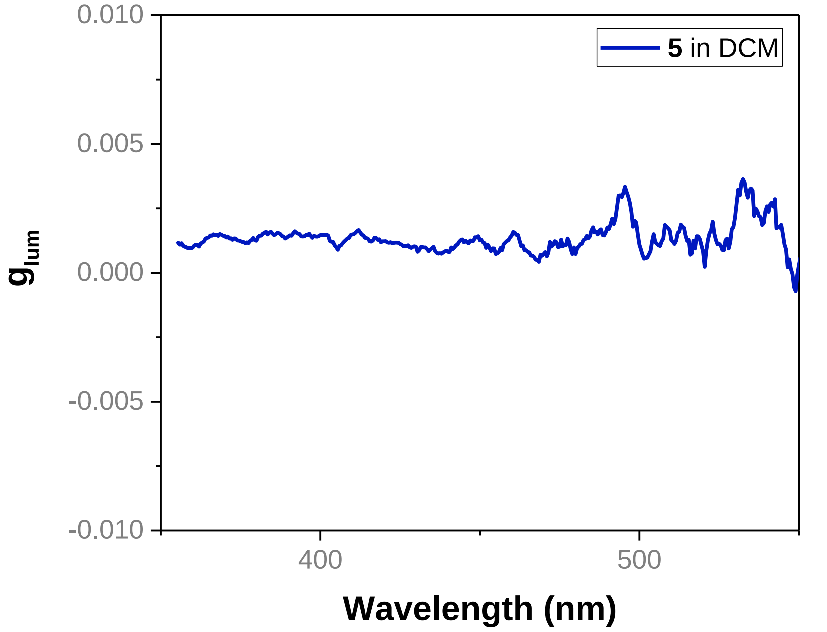

Figure S190. CPL (g_{lum}) of **5** in DCM at 25 °C (ca. 5×10^{-5} M)

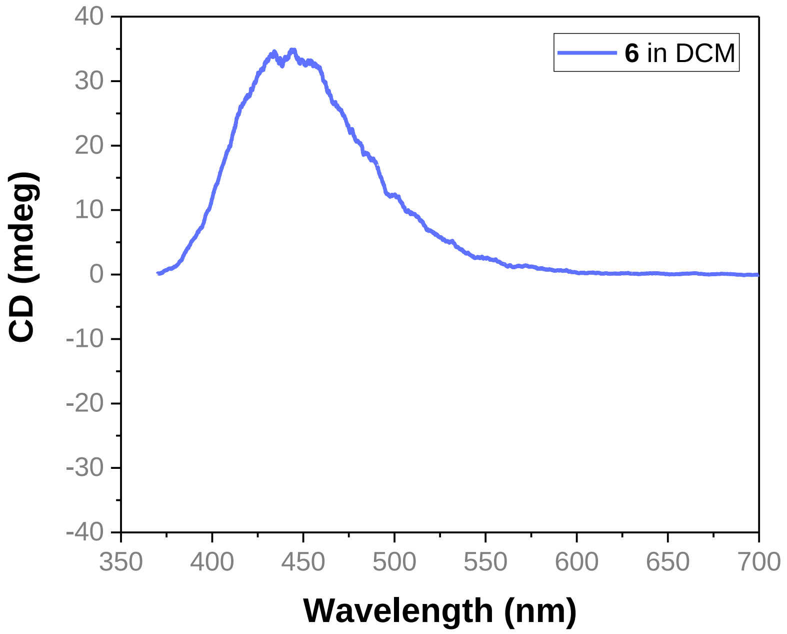

Figure S191. CPL spectrum of **6** in DCM at 25 °C (ca. 5×10^{-5} M)

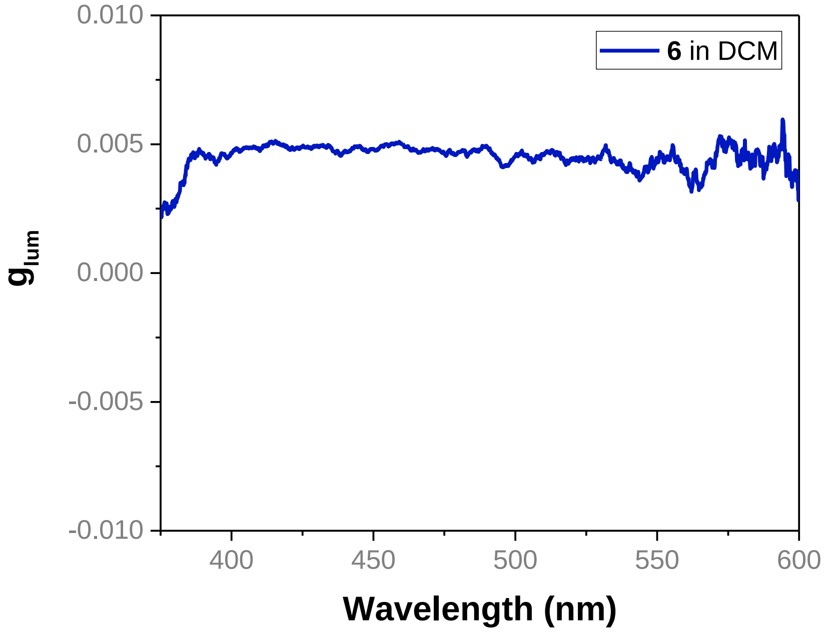

Figure S192. CPL (g_{lum}) of **6** in DCM at 25 °C (ca. 5×10^{-5} M)

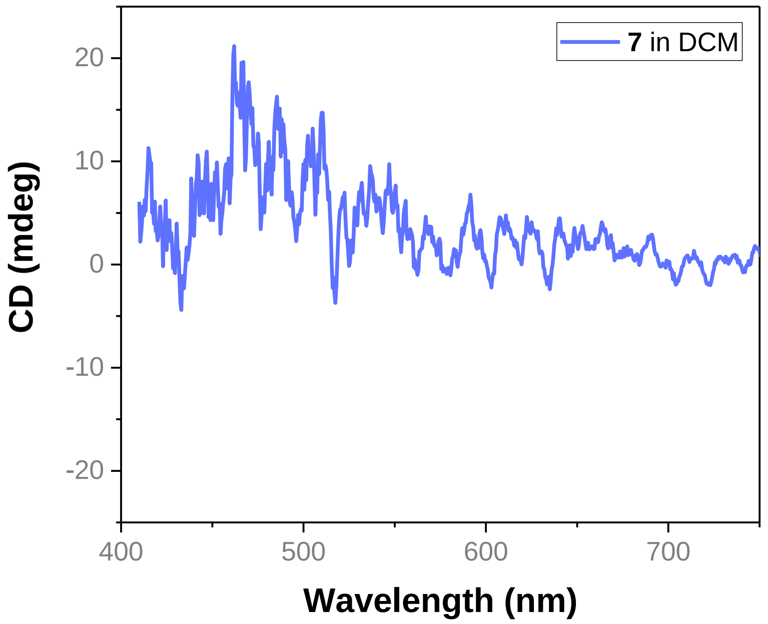

Figure S193. CPL spectrum of **7** in DCM at 25 ºC (ca. 5×10^{-5} M)

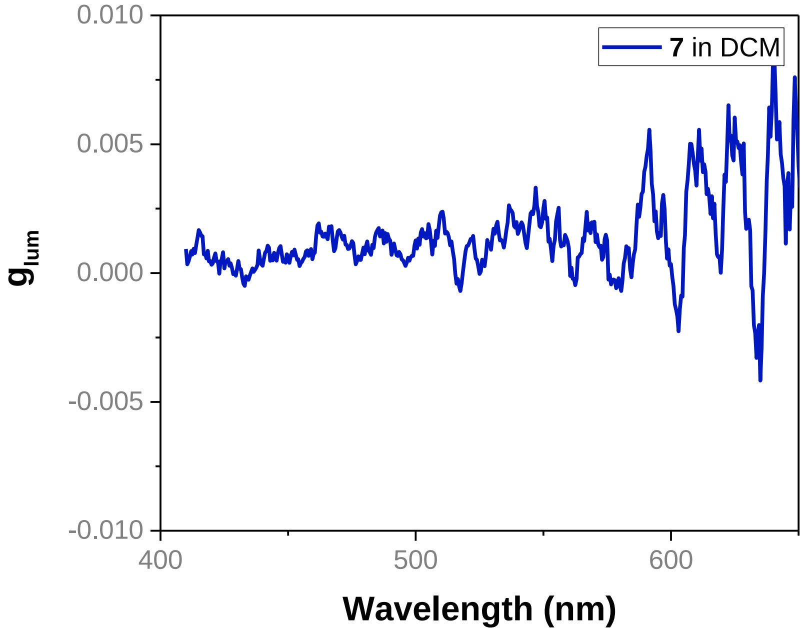

Figure S194. CPL (g$_{lum}$) of **7** in DCM at 25 ºC (ca. 5×10^{-5} M)

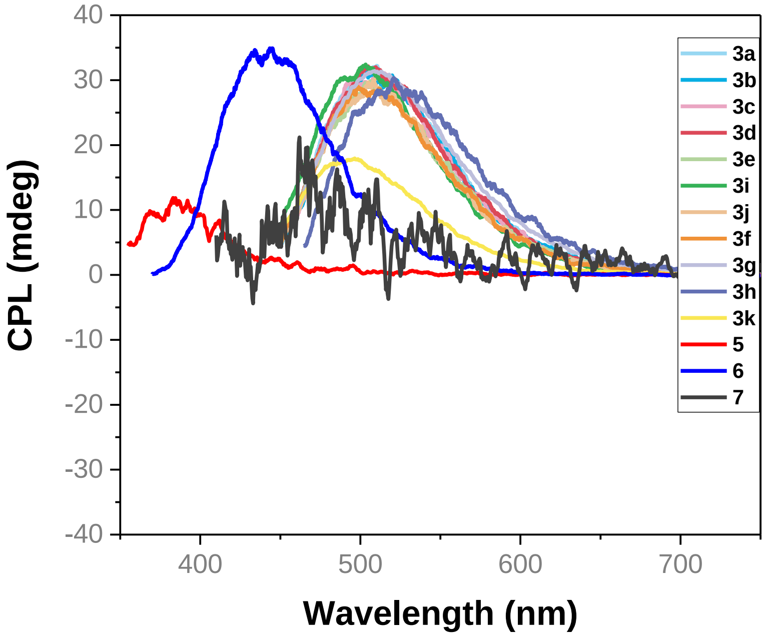

Figure S195. CPL spectra of **3a**-**3k**, **5**, **6**, and **7** in DCM at 25 ºC

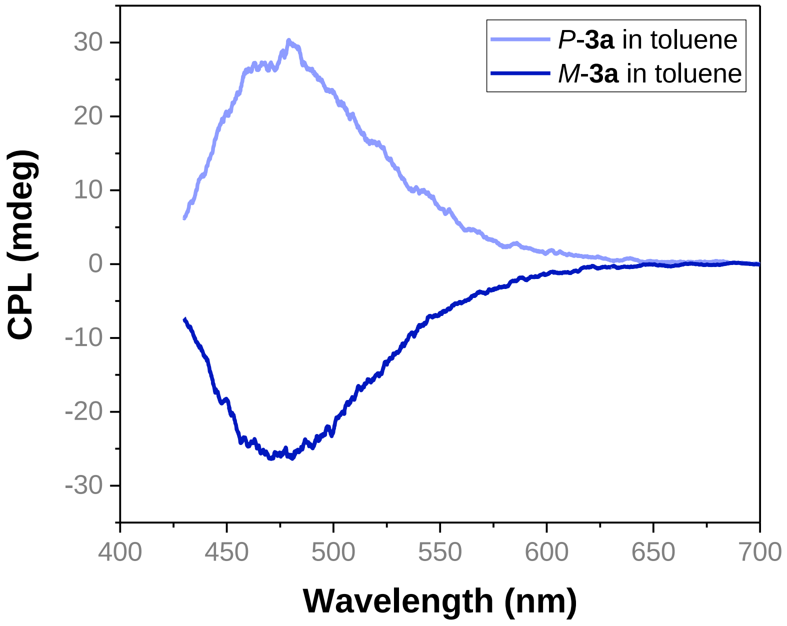

Figure S196. CPL spectra of *P*-**3a and** *M*-**3a** in toluene at 25 ºC (ca. 5×10^{-5} M)

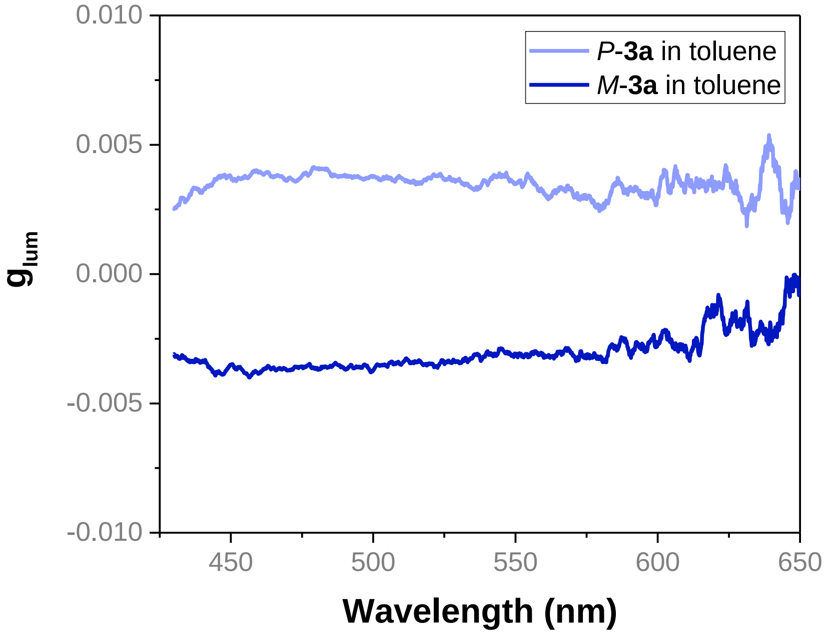

Figure S197. CPL (g_{lum}) of *P*-**3a** and *M*-**3a** in toluene at 25 ºC (ca. 5×10^{-5} M)

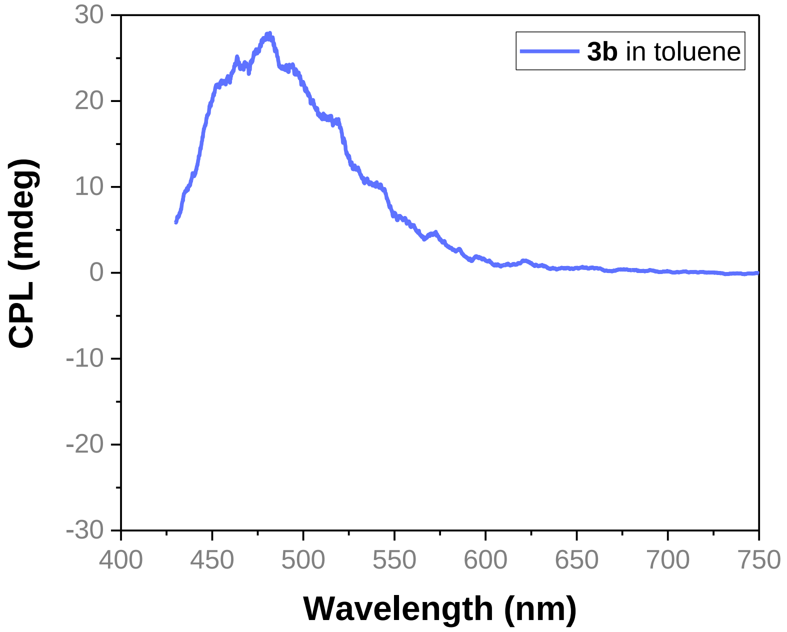

Figure S198. CPL spectrum of **3b** in toluene at 25 ºC (ca. 5×10^{-5} M)

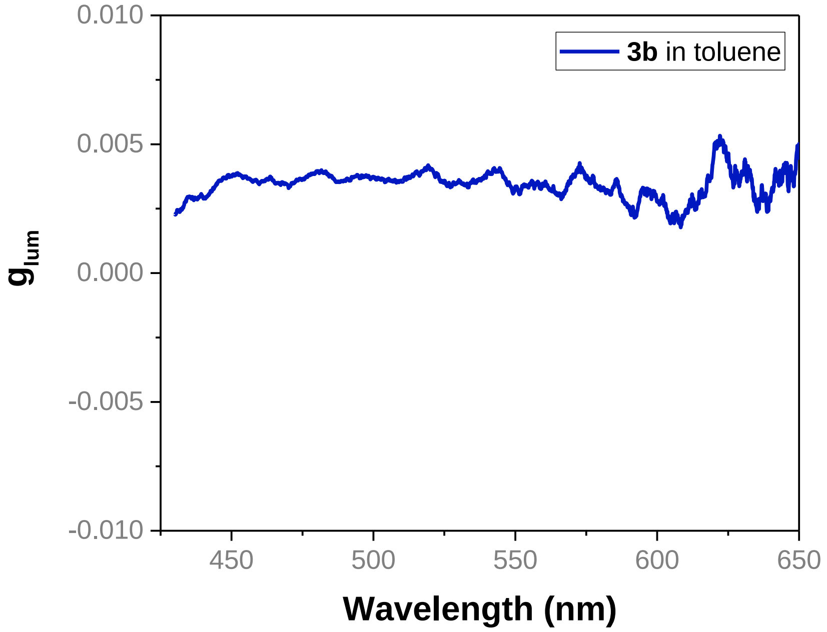

Figure S199. CPL (g_{lum}) of **3b** in toluene at 25 ºC (ca. 5×10^{-5} M)

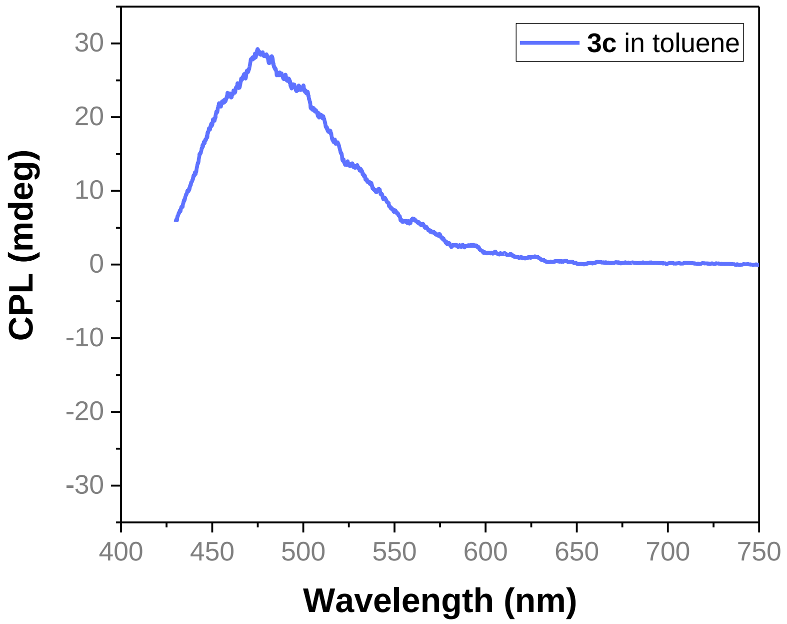

Figure S200. CPL spectrum of **3c** in toluene at 25 ºC (ca. 5×10^{-5} M)

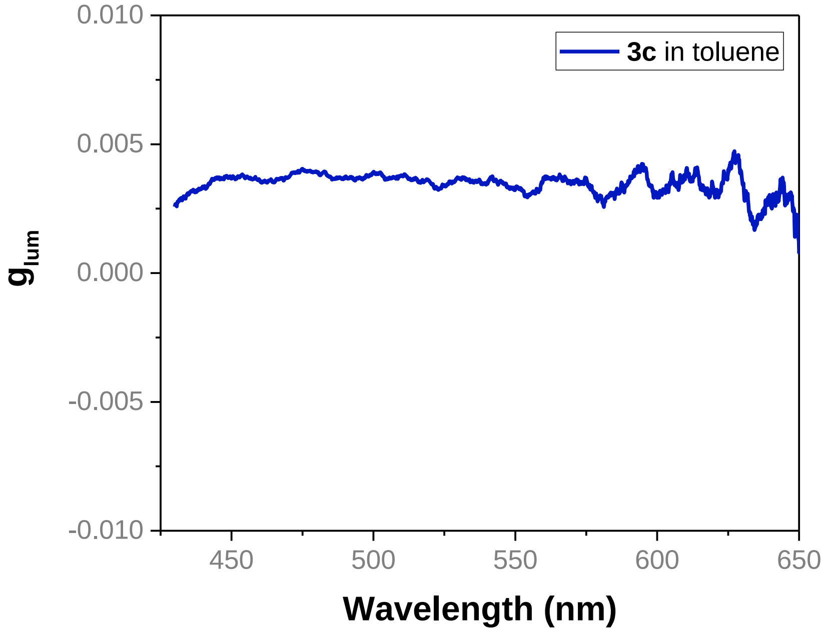

Figure S201. CPL (g_{lum}) of **3c** in toluene at 25 ºC (ca. 5×10^{-5} M)

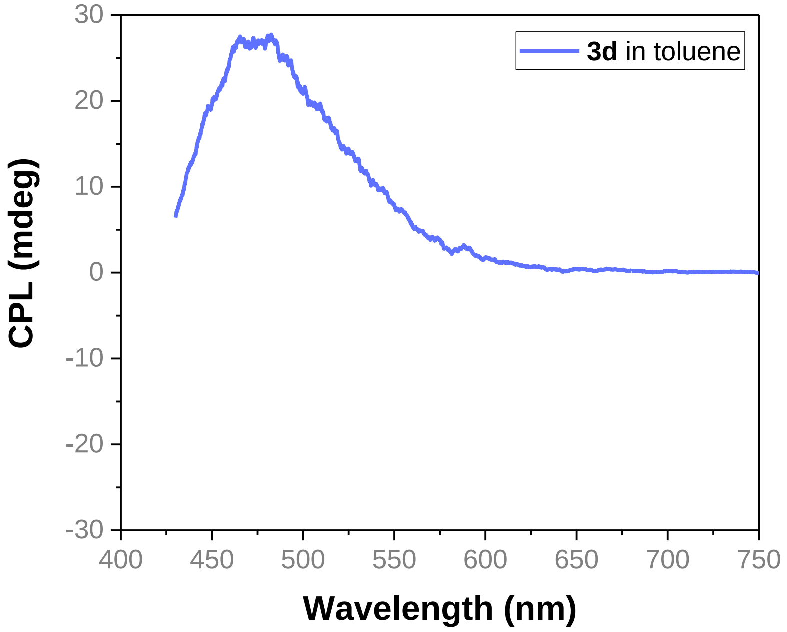

Figure S202. CPL spectrum of **3d** in toluene at 25 ºC (ca. 5×10^{-5} M)

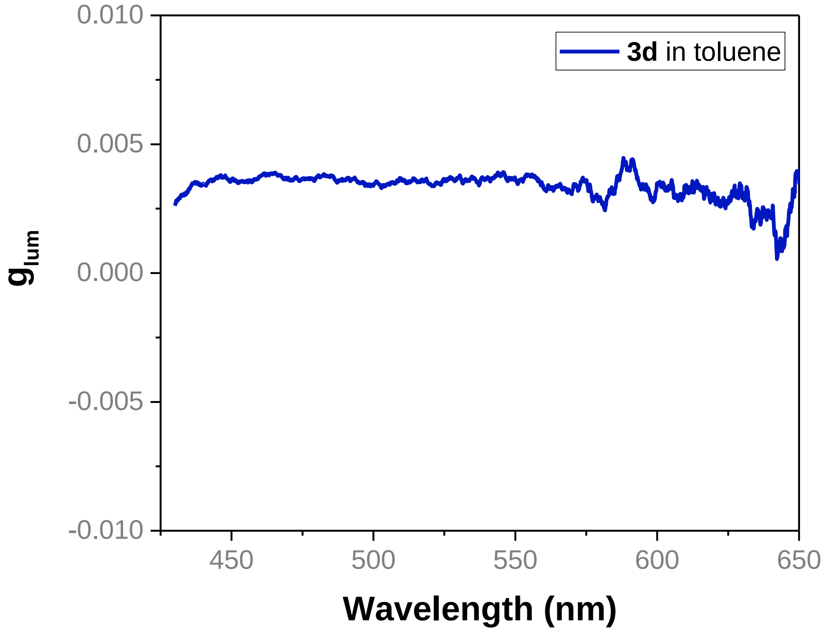

Figure S203. CPL (g_{lum}) of **3d** in toluene at 25 ºC (ca. 5×10^{-5} M)

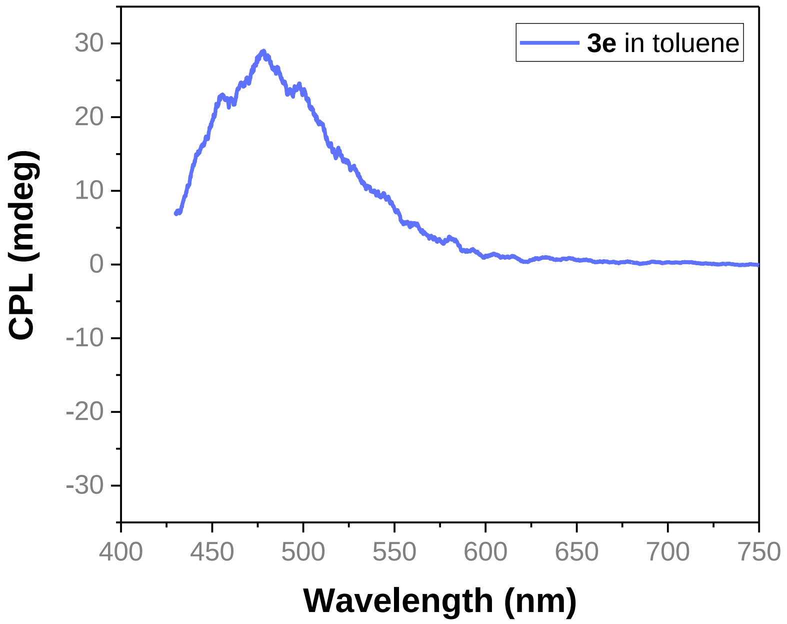

Figure S204. CPL spectrum of **3e** in toluene at 25 °C (ca. 5×10^{-5} M)

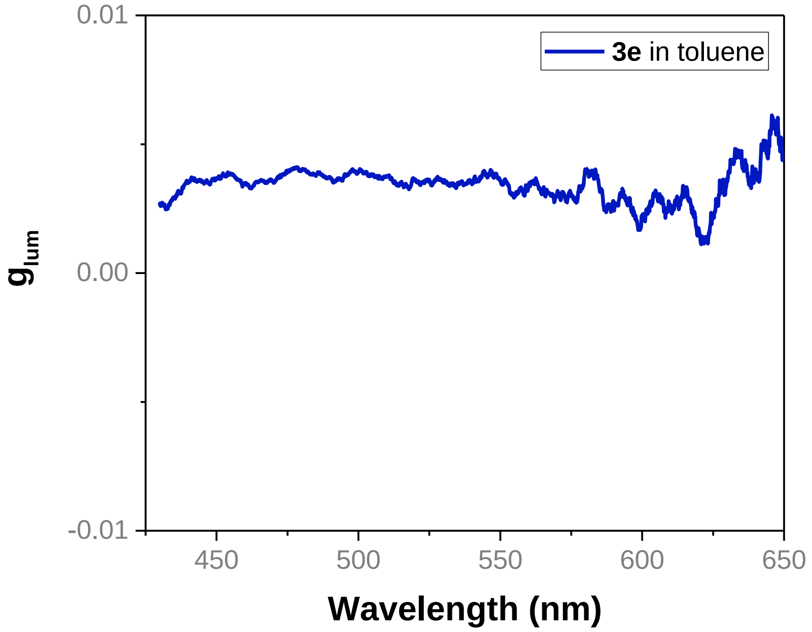

Figure S205. CPL (g_{lum}) of **3e** in toluene at 25 °C (ca. 5×10^{-5} M)

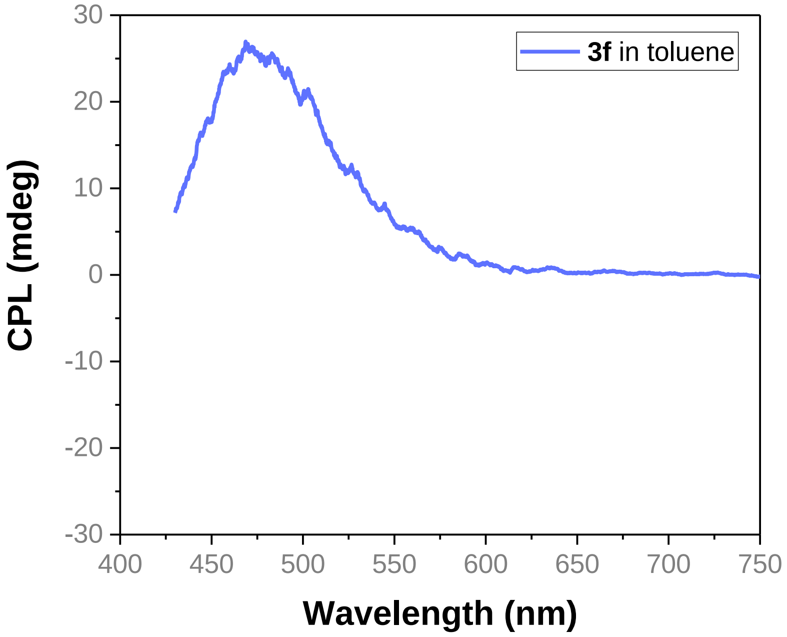

Figure S206. CPL spectrum of **3f** in toluene at 25 ºC (ca. 5×10^{-5} M)

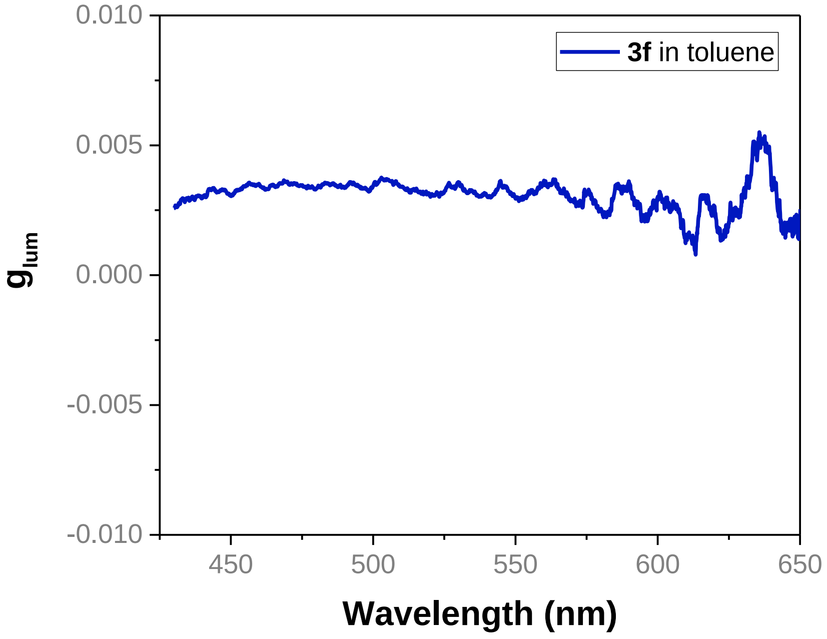

Figure S207. CPL (g_{lum}) of **3f** in toluene at 25 ºC (ca. 5×10^{-5} M)

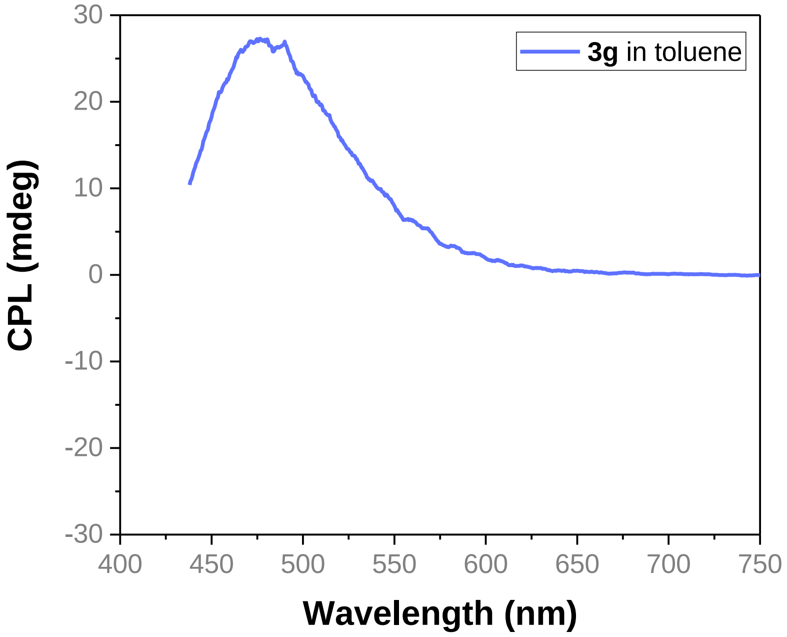

Figure S208. CPL spectrum of **3g** in toluene at 25 ºC (ca. 5×10^{-5} M)

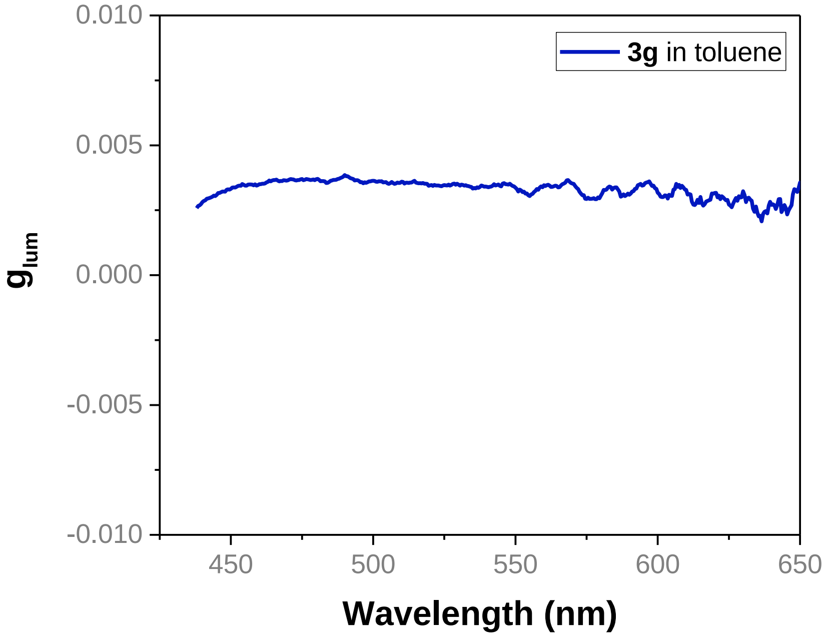

Figure S209. CPL (g_{lum}) of **3g** in toluene at 25 ºC (ca. 5×10^{-5} M)

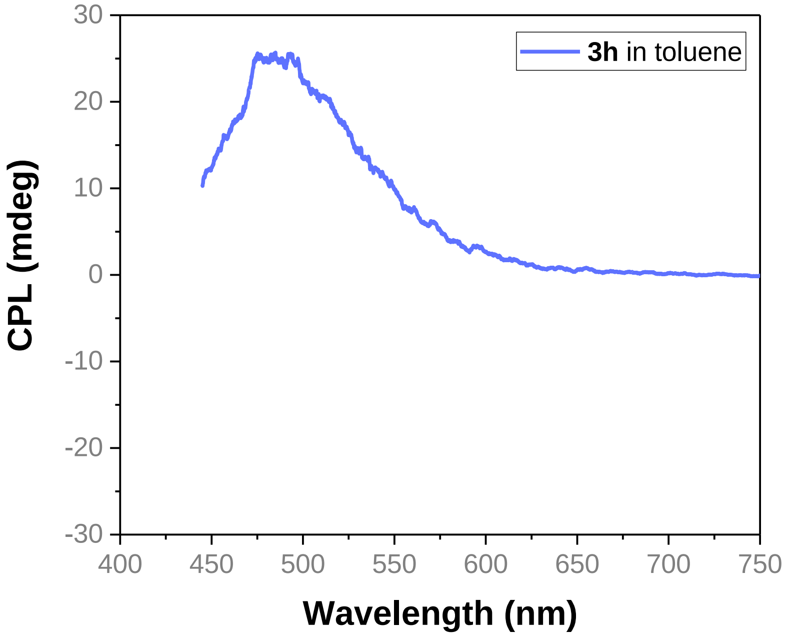

Figure S210. CPL spectrum of **3h** in toluene at 25 ºC (ca. 5×10^{-5} M)

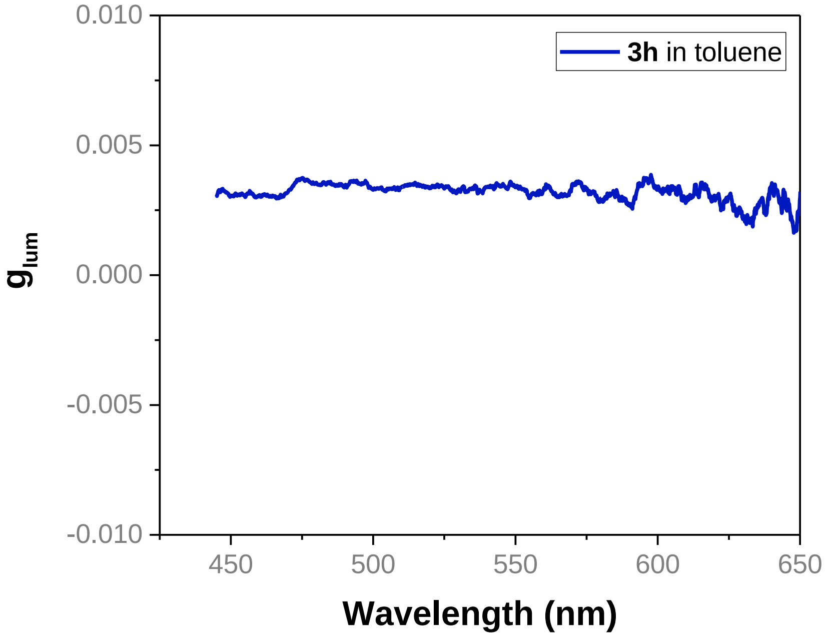

Figure S211. CPL (g_{lum}) of **3h** in toluene at 25 ºC (ca. 5×10^{-5} M)

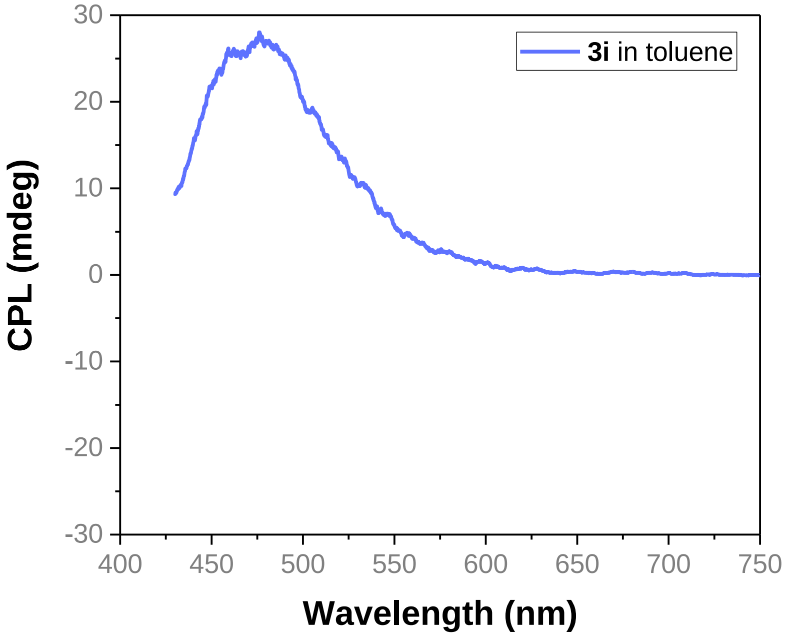

Figure S212. CPL spectrum of **3i** in toluene at 25 ºC (ca. 5×10^{-5} M)

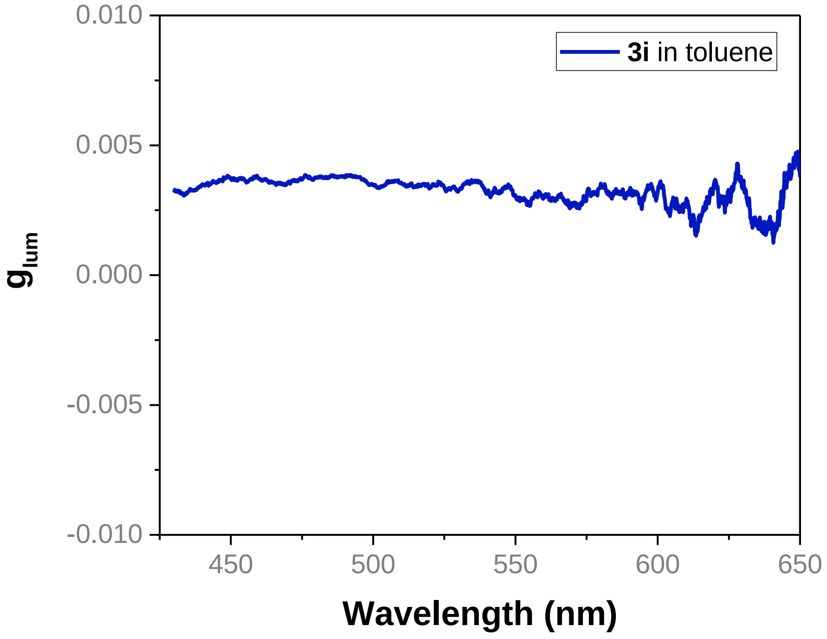

Figure S213. CPL (g_{lum}) of **3i** in toluene at 25 ºC (ca. 5×10^{-5} M)

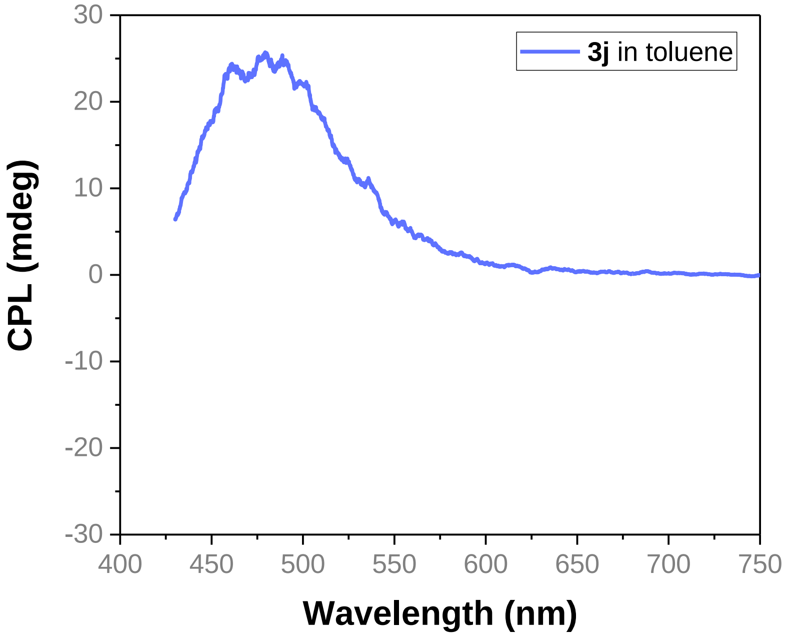

Figure S214. CPL spectrum of **3j** in toluene at 25 ºC (ca. 5×10^{-5} M)

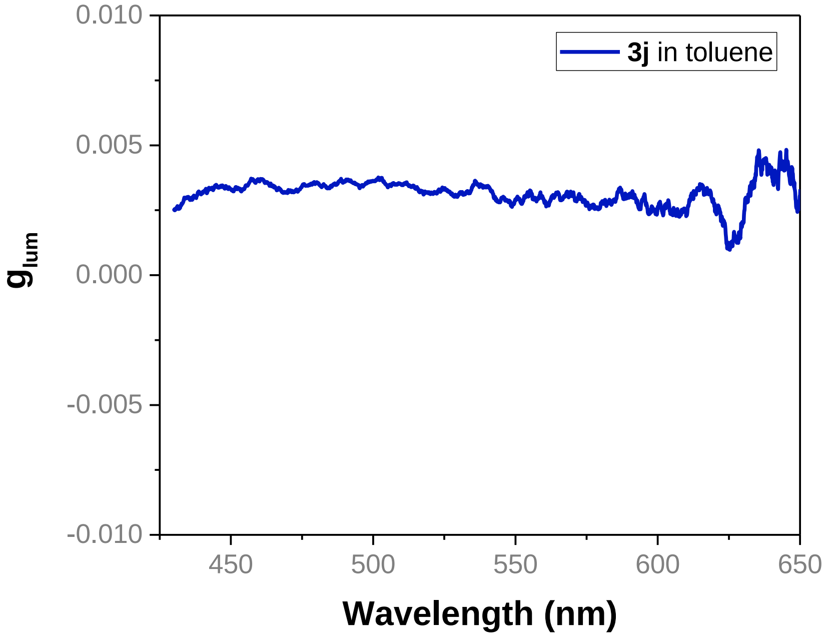

Figure S215. CPL (g_{lum}) of **3j** in toluene at 25 ºC (ca. 5×10^{-5} M)

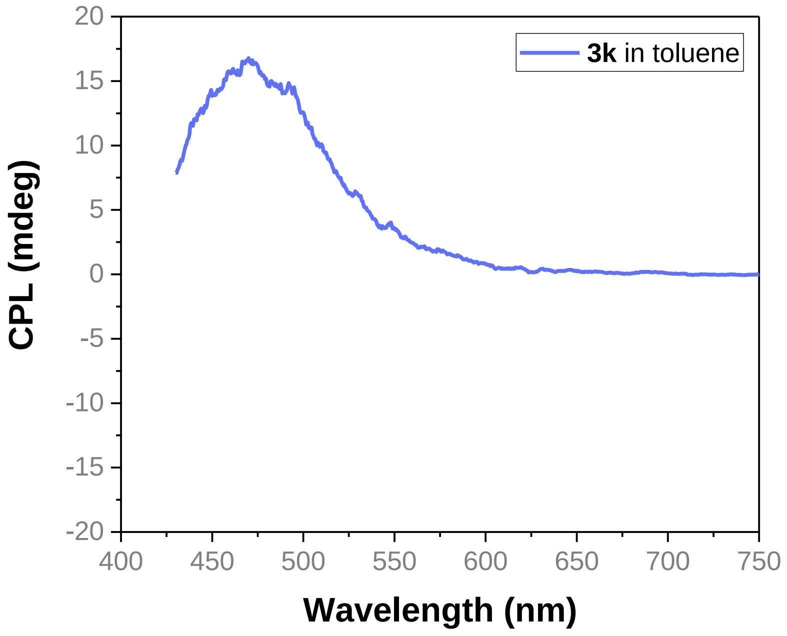

Figure S216. CPL spectrum of **3k** in toluene at 25 ºC (ca. 5×10^{-5} M)

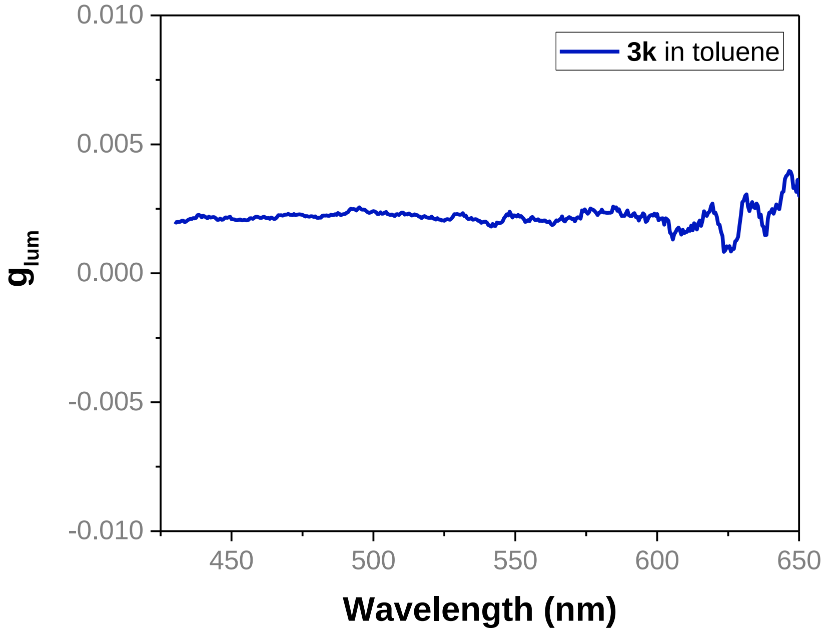

Figure S217. CPL (g_{lum}) of **3k** in toluene at 25 ºC (ca. 5×10^{-5} M)

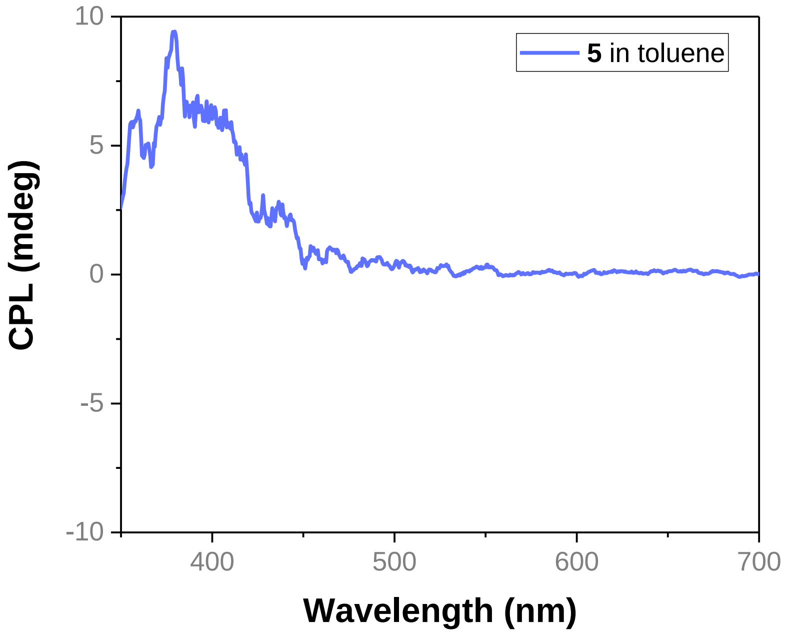

Figure S218. CPL spectrum of **5** in toluene at 25 ºC (ca. 5×10^{-5} M)

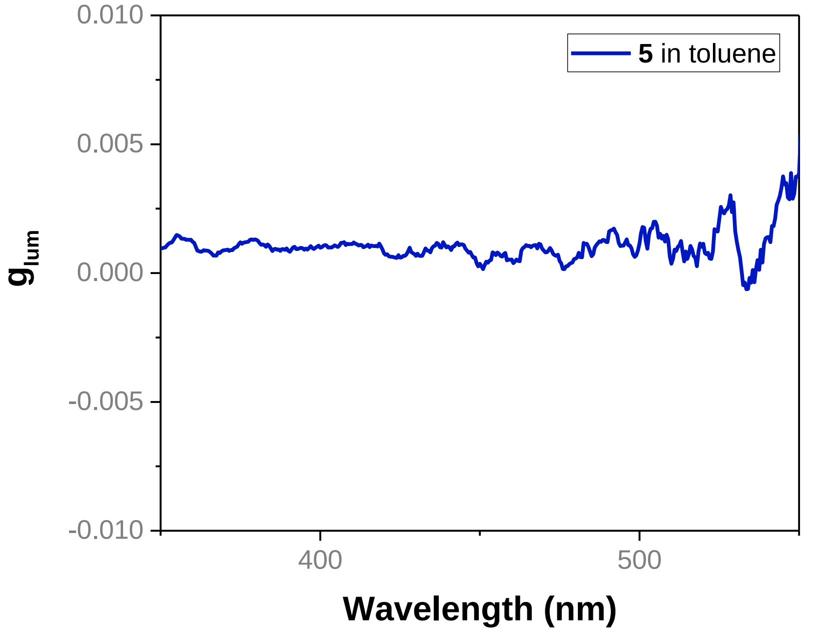

Figure S219. CPL (g_{lum}) of **5** in toluene at 25 ºC (ca. 5×10^{-5} M)

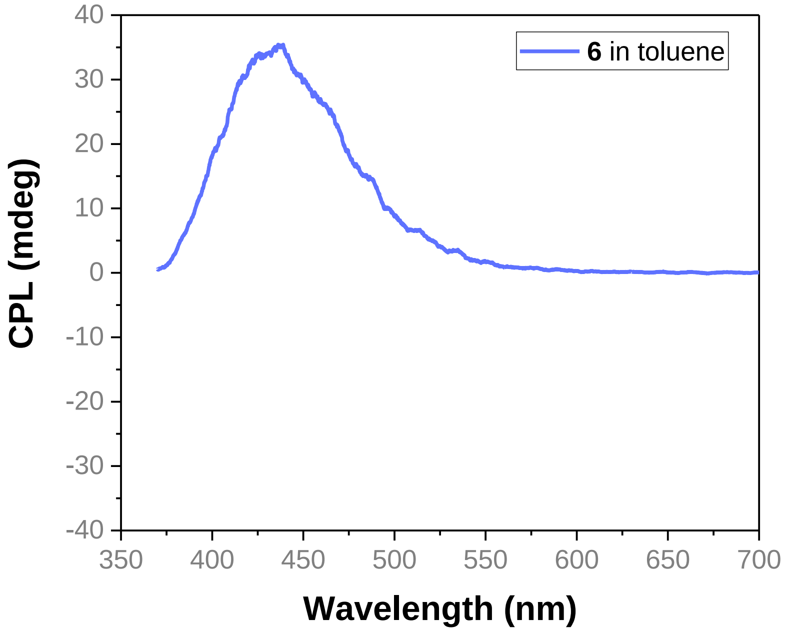

Figure S220. CPL spectrum of **6** in toluene at 25 ºC (ca. 5×10^{-5} M)

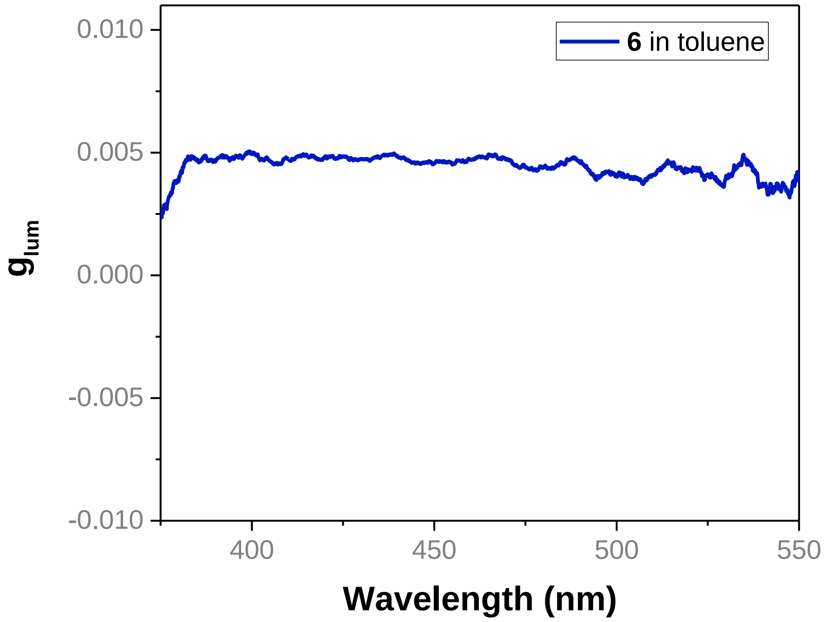

Figure S221. CPL (g_{lum}) of **6** in toluene at 25 ºC (ca. 5×10^{-5} M)

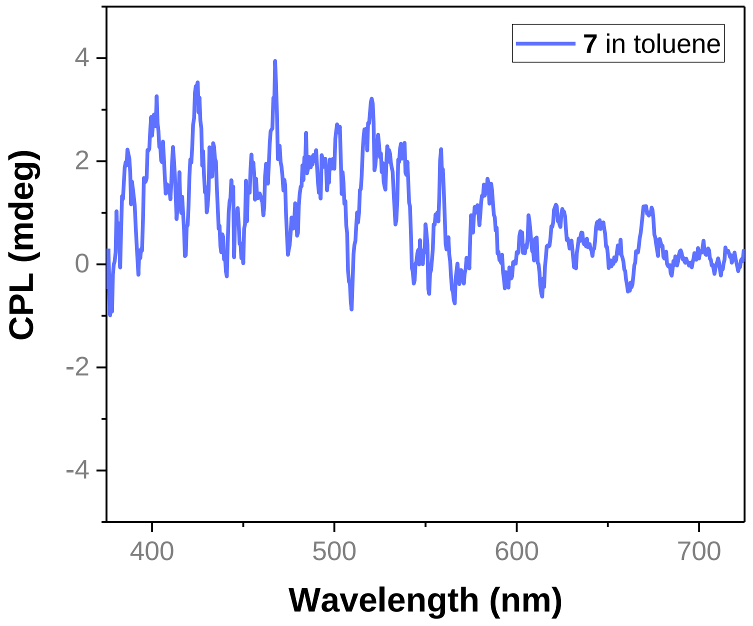

Figure S222. CPL spectrum of **7** in toluene at 25 ºC (ca. 5×10^{-5} M)

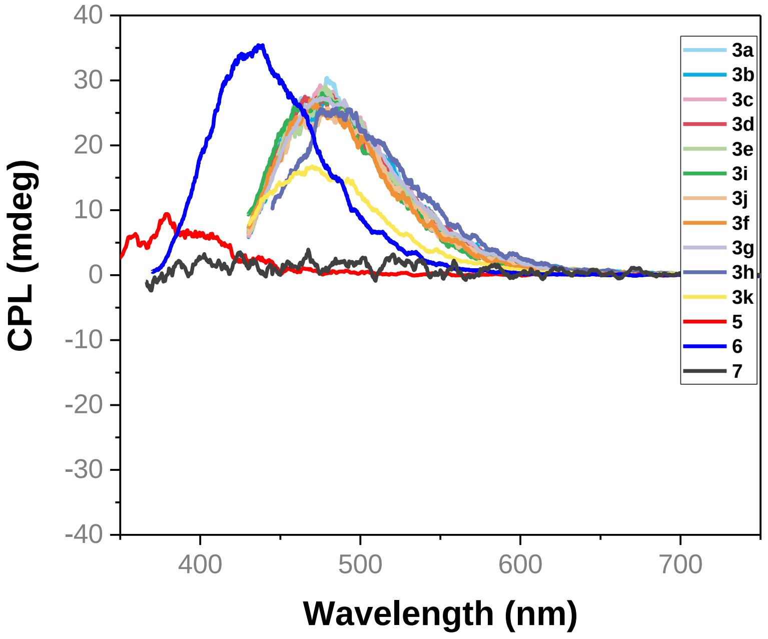

Figure S223. CPL spectrum of **3a-3k**, **5**, **6**, and **7** in toluene at 25 ºC

19. Summary of Optical properties

Table S3. Photophysical properties of 3, 5, 6 and 7 in CH_3CN

compd	λ_{max} [nm]	ε_{max} [M^{-1} cm^{-1}][a]	λ_{ex} [nm]	λ_{em} [nm]	Φ_{fl}[b]	λ_{CD} [nm][c]	$\lvert g_{abs}\rvert$ 10^{-3}	λ_{CPL} [nm][d]	$\lvert g_{lum}\rvert$ 10^{-3}
**3a**	388	2966	388	515	13.0%	391	6.4	510	5.0
**3b**	388	4157	389	512	16.1%	386	5.9	510	4.8
**3c**	388	5128	390	514	13.0%	388	6.1	508	4.9
**3d**	385	2161	390	514	10.7%	389	6.0	511	4.8
**3e**	384	3273	386	511	16.1%	386	5.9	510	4.6
**3f**	386	3454	389	514	16.3%	389	5.9	505	4.5
**3g**	391	2593	392	517	10.1%	391	6.5	511	4.9
**3h**	391	2527	393	530	6.3%	394	5.6	527	4.4
**3i**	385	3256	385	508	22.7%	387	6.2	503	5.0
**3j**	386	3494	387	510	18.2%	389	5.3	509	4.5
**3k**	384	5202	385	493	27.5%	385	3.7	493	2.5
**5**	330	12719	333	383	10.7%	331	2.1	384	1.6
**6**	321	7138	325	443	18.3%	322	3.8	442	4.7
**7**	352	4472	355	467	0.8%	357	2.3	457	1.7

[a]Measured in dilute CH_3CN solution (3.0×10^{-5} M). [b]The quantum yield was determined using quinine sulfate as reference, ϕ = 0.577 in 0.1 M H_2SO_4, λ_{ex} = 350 nm. [c]The wavelength corresponding to the first Cotton effect. [d]Measured in dilute solution (5.0×10^{-5} M).

Table S4. Photophysical properties of 3, 5, 6 and 7 in CH_2Cl_2

compd	λ_{max} [nm]	ε_{max} [M^{-1} cm^{-1}][a]	λ_{ex} [nm]	λ_{em} [nm]	Φ_{fl}[b]	λ_{CD} [nm][c]	$\lvert g_{abs}\rvert$ 10^{-3}	λ_{CPL} [nm][d]	$\lvert g_{lum}\rvert$ 10^{-3}
**3a**	386	2773	385	511	19.8%	389	5.6	510	4.4
**3b**	387	4339	385	512	19.4%	387	5.3	511	4.2
**3c**	385	2381	387	511	20.7%	386	5.2	510	4.3
**3d**	387	2650	389	510	19.3%	387	5.5	510	4.4
**3e**	384	3456	385	510	21.1%	387	5.0	507	4.1
**3f**	387	4467	386	512	19.7%	389	5.2	506	3.9
**3g**	388	5607	393	516	15.1%	391	5.6	510	4.3
**3h**	394	2603	395	531	10.3%	391	5.0	521	4.1
**3i**	385	2999	386	505	22.0%	384	5.5	501	4.4
**3j**	384	3319	385	509	21.6%	388	5.2	508	4.1
**3k**	386	4919	386	496	32.2%	382	3.2	492	2.5
**5**	332	9688	332	383	13.0%	332	1.5	385	1.5
**6**	322	6717	326	443	21.4%	322	3.4	443	4.9
**7**	352	3950	355	466	1.2%	358	2.1	466	1.8

[a]Measured in dilute CH_2Cl_2 solution (3.0×10^{-5} M). [b]The quantum yield was determined using quinine sulfate as reference, ϕ = 0.577 in 0.1 M H_2SO_4, λ_{ex} = 350 nm. [c]The wavelength corresponding to the first Cotton effect. [d]Measured in dilute solution (5.0×10^{-5} M).

Table S5. Photophysical properties of 3, 5, 6 and 7 in toluene

compd	λ_{max} [nm]	ε_{max} [M^{-1} cm^{-1}][a]	λ_{ex} [nm]	λ_{em} [nm]	Φ_{fl}[b]	λ_{CD} [nm][c]	$\lvert g_{abs}\rvert$ 10^{-3}	λ_{CPL} [nm][d]	$\lvert g_{lum}\rvert$ 10^{-3}
**3a**	377	3360	377	476	14.8%	377	5.4	475	3.8
**3b**	377	4321	376	477	17.0%	375	4.7	479	3.9
**3c**	377	4164	378	477	17.3%	378	5.3	475	4.0
**3d**	377	3325	378	476	19.4%	378	5.0	475	3.7
**3e**	378	3735	378	476	18.5%	375	5.3	478	4.0
**3f**	377	3471	377	477	14.1%	380	4.8	475	3.4
**3g**	378	5246	383	478	10.6%	380	5.1	479	3.7
**3h**	385	3499	385	488	12.2%	385	4.4	485	3.6
**3i**	376	2985	376	473	16.8%	375	5.4	476	3.9
**3j**	377	3654	375	477	22.8%	378	4.9	479	3.6
**3k**	379	5841	378	468	15.8%	376	3.4	472	2.3
**5**	332	14863	333	378	11.3%	331	1.4	379	1.3
**6**	321	8222	324	431	22.4%	322	3.3	434	4.8
**7**	350	4874	370	435	0.4%	358	2.6	455	1.3

[a]Measured in dilute CH_2Cl_2 solution (3.0×10^{-5} M). [b]The quantum yield was determined using quinine sulfate as reference, $\phi = 0.577$ in 0.1 M H_2SO_4, $\lambda_{ex} = 350$ nm. [c]The wavelength corresponding to the first Cotton effect. [d]Measured in dilute solution (5.0×10^{-5} M).

20. References

1. R. Hodgkinson, A. D. Grosso, G. Clarkson, M. Will, *Dalton Trans.* **2016**, *45*, 3992-4005.
2. T. R. Wu, L. Shen, J. M. Chong, *Org. Lett.* **2004**, *6*, 2701-2704.
3. H. Bao, X. Qi, U. K. Tambar, *J. Am. Chem. Soc.* **2011**, *133*, 1206-1208.

^{1}H NMR (400 MHz, 298 K) spectrum of **L6** in $CDCl_3$

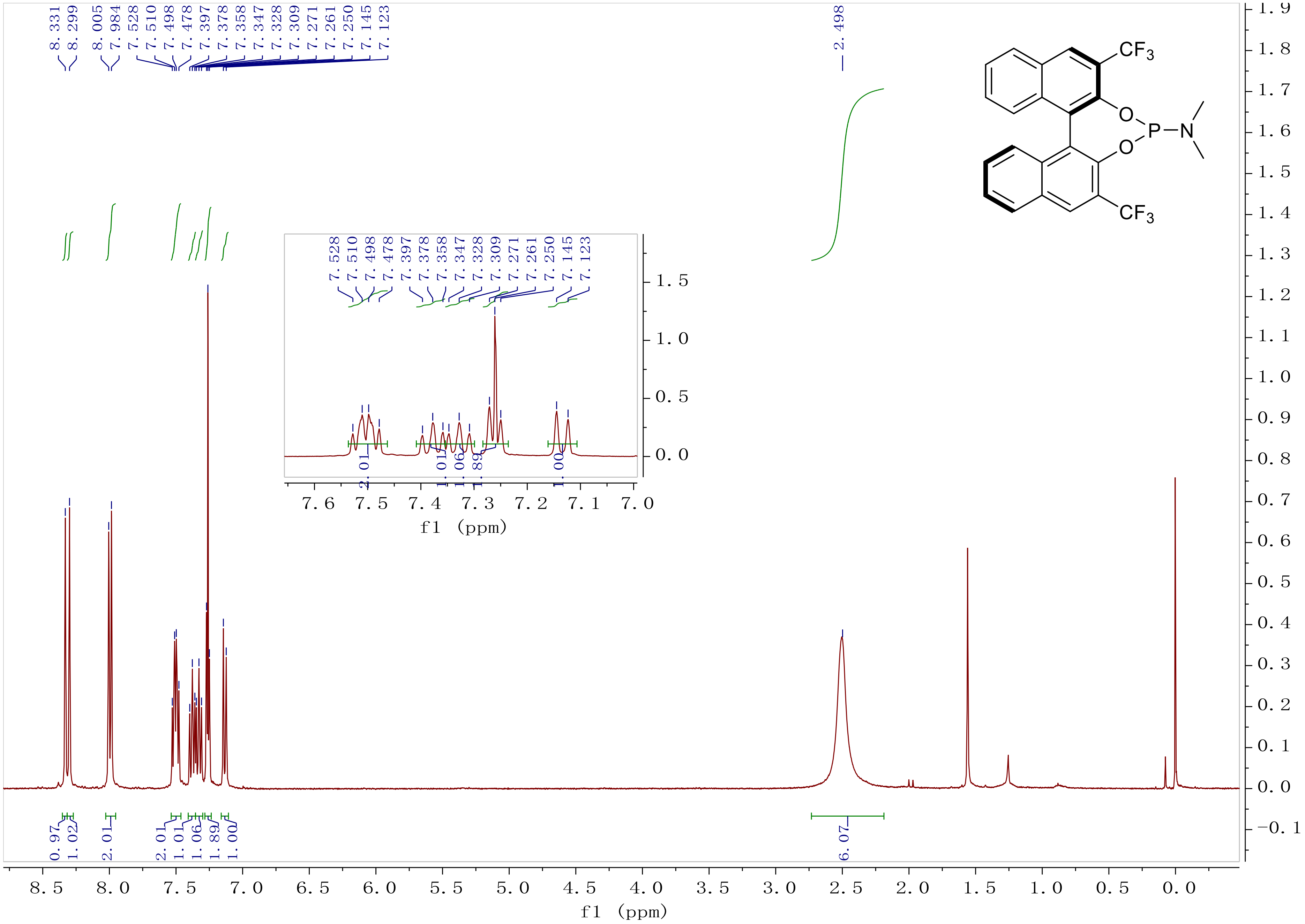

^{13}C NMR (100 MHz, 298 K) spectrum of **L6** in $CDCl_3$

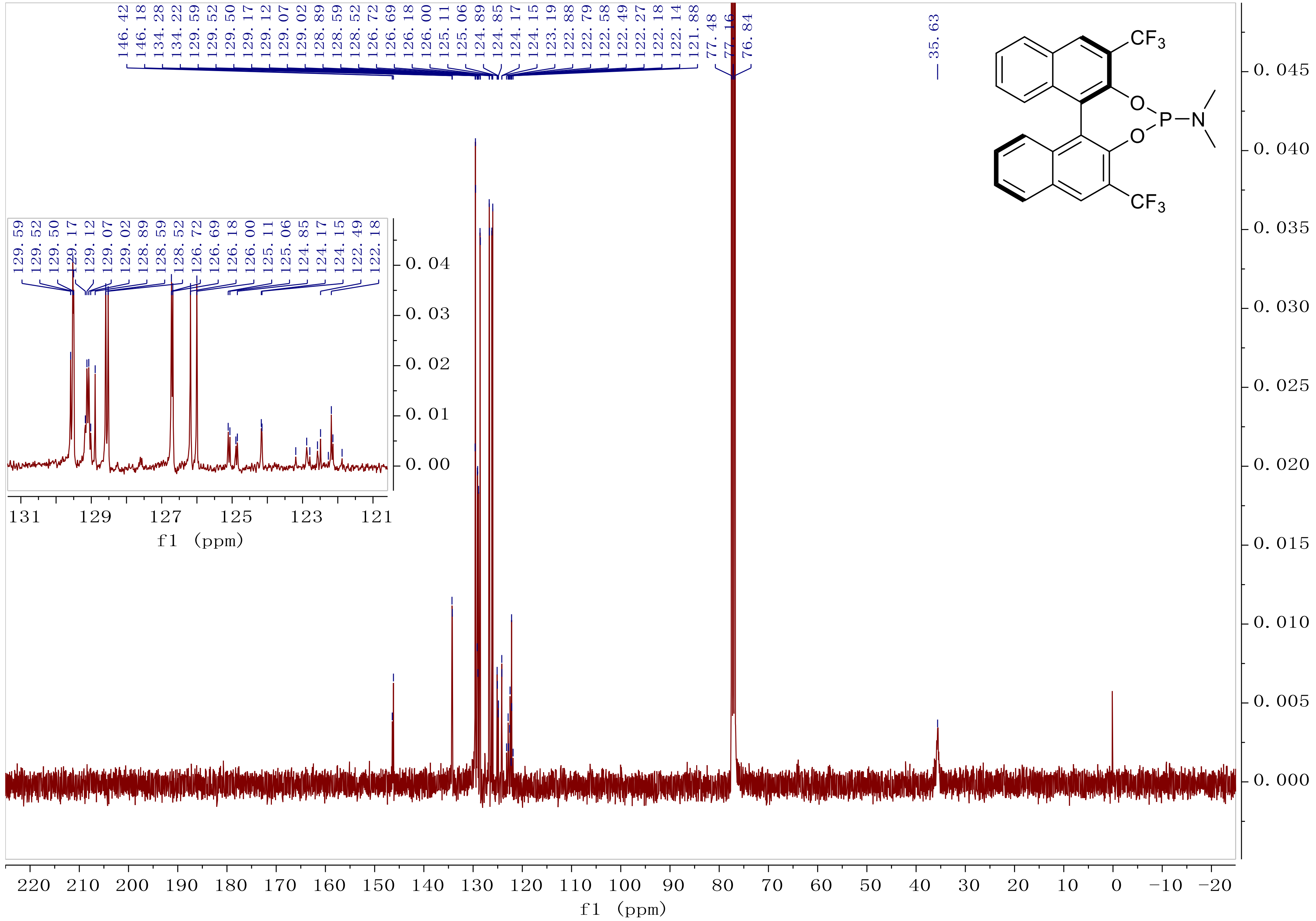

^{1}H NMR (400 MHz, 298 K) spectrum of **L10** in $CDCl_3$

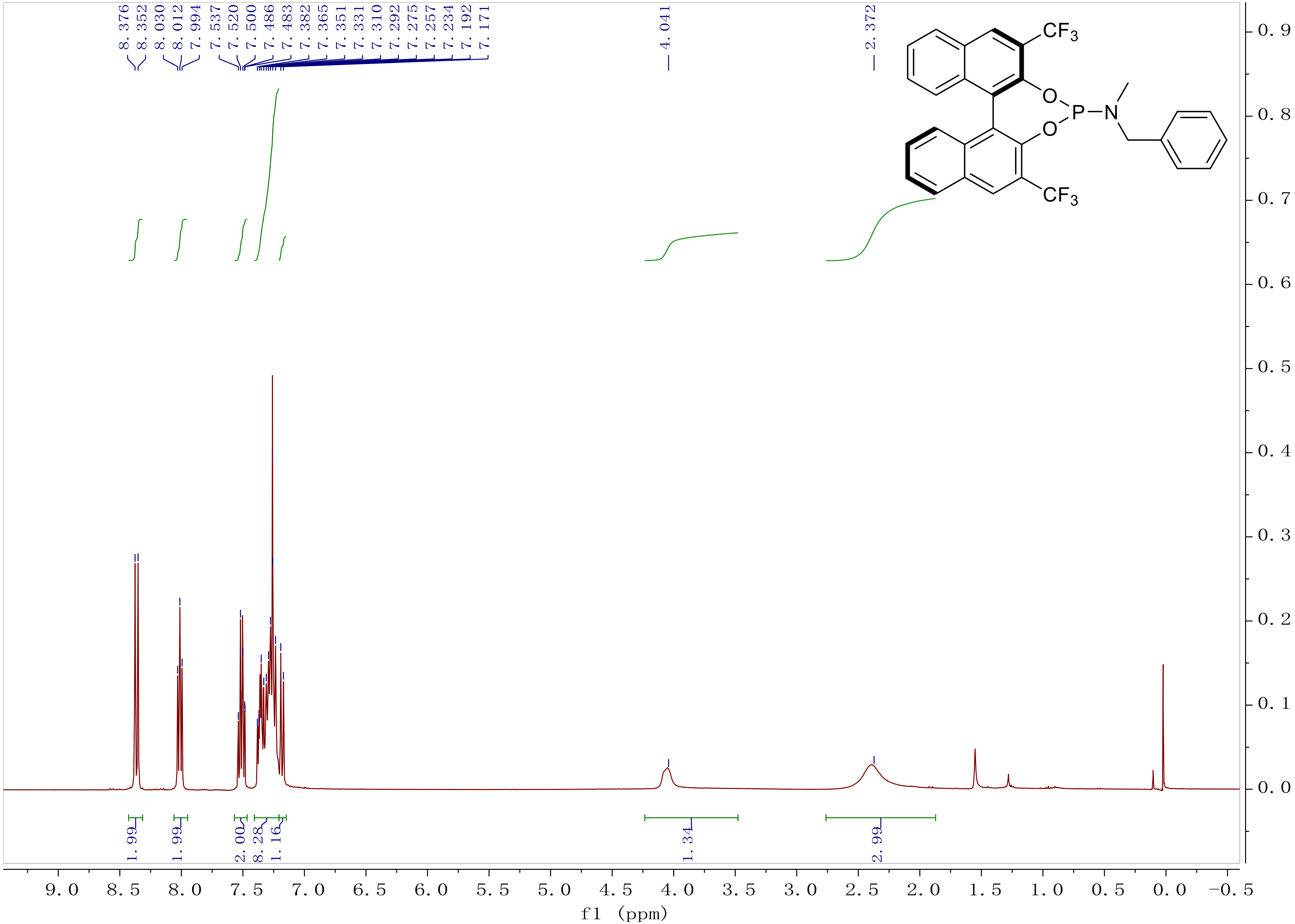

^{13}C NMR (100 MHz, 298 K) spectra of **L10** in $CDCl_3$

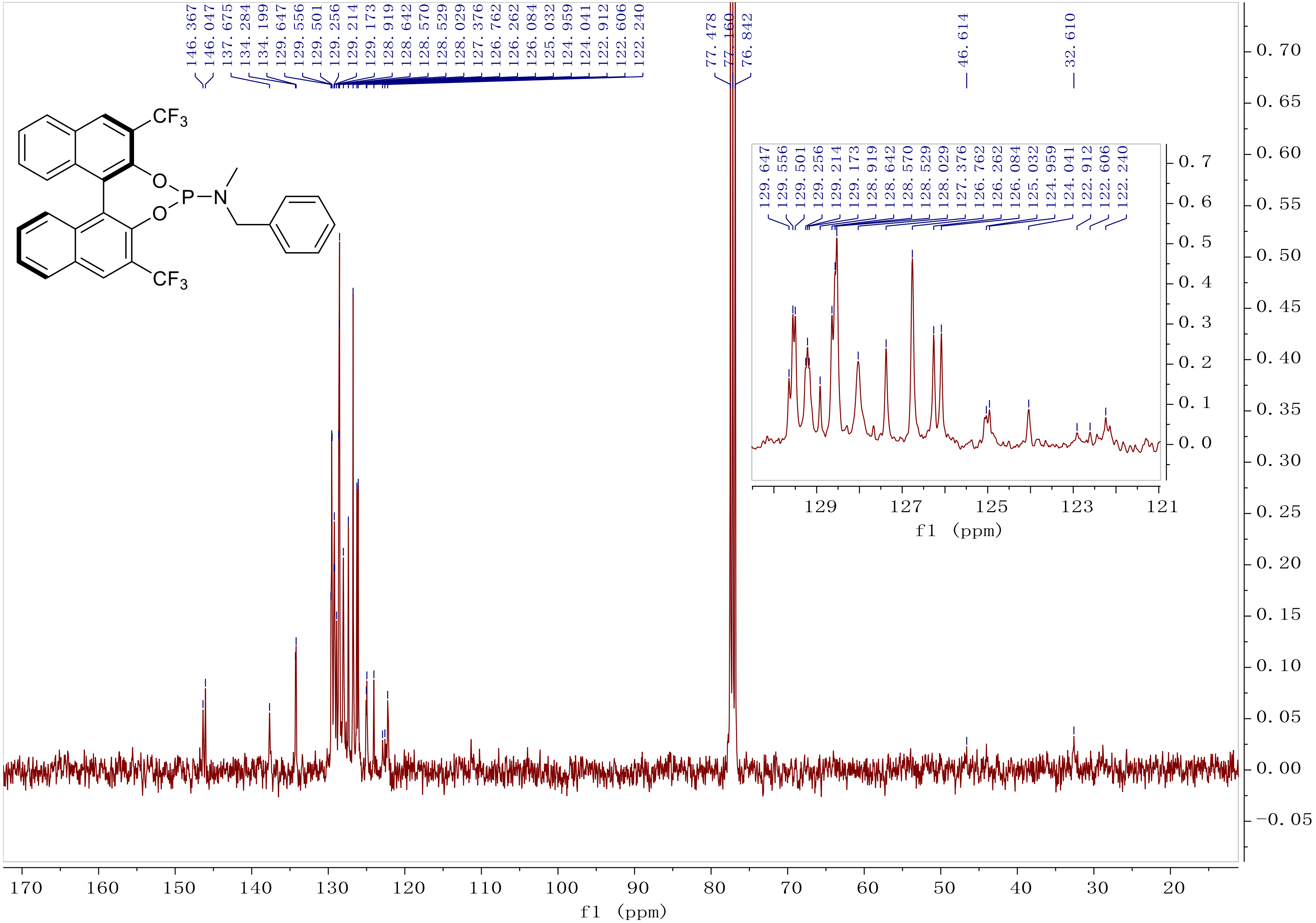

^1H NMR (400 MHz, 298 K) spectrum of **2a** in $CDCl_3$

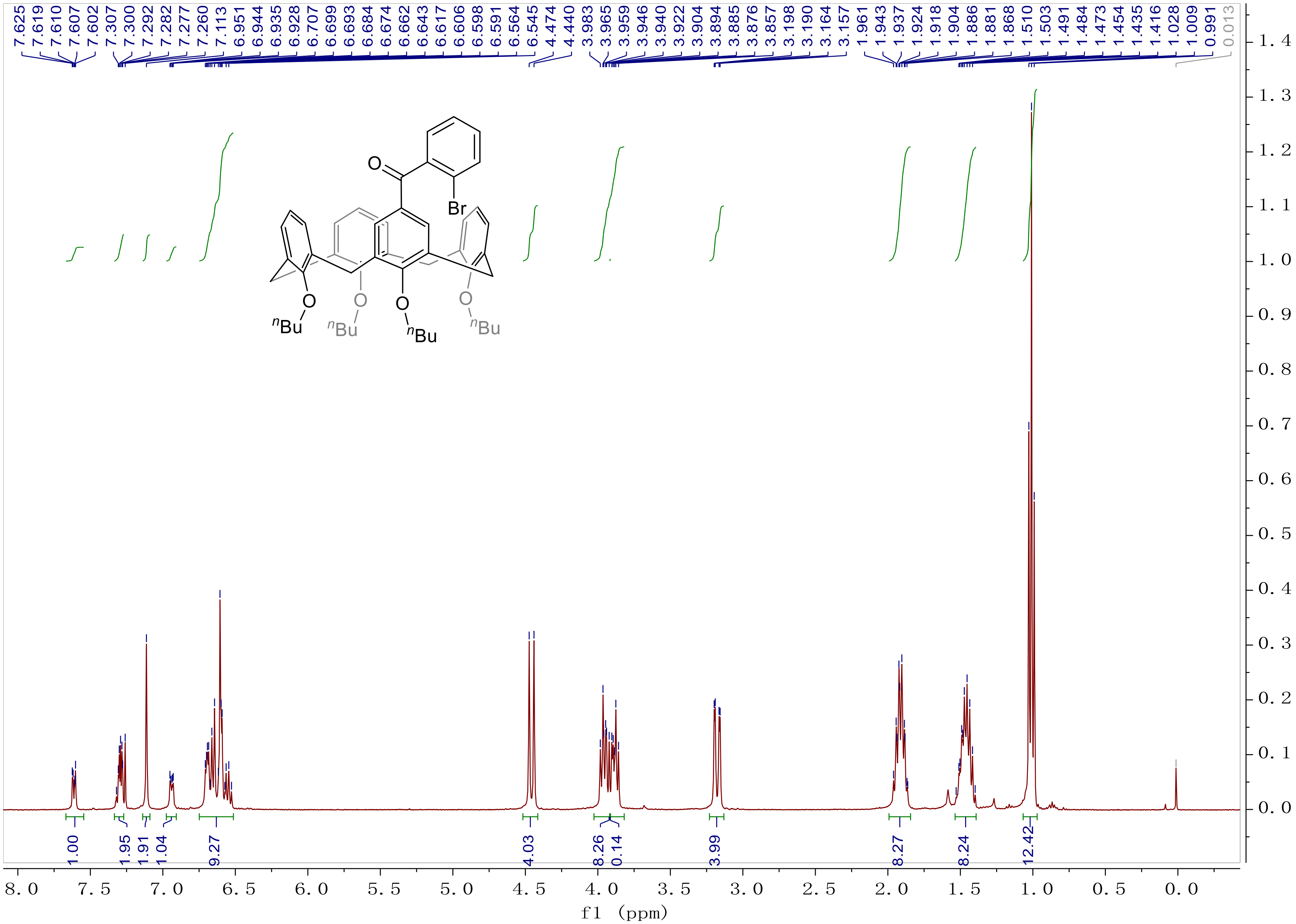

^{13}C NMR (100 MHz, 298 K) spectrum of **2a** in $CDCl_3$

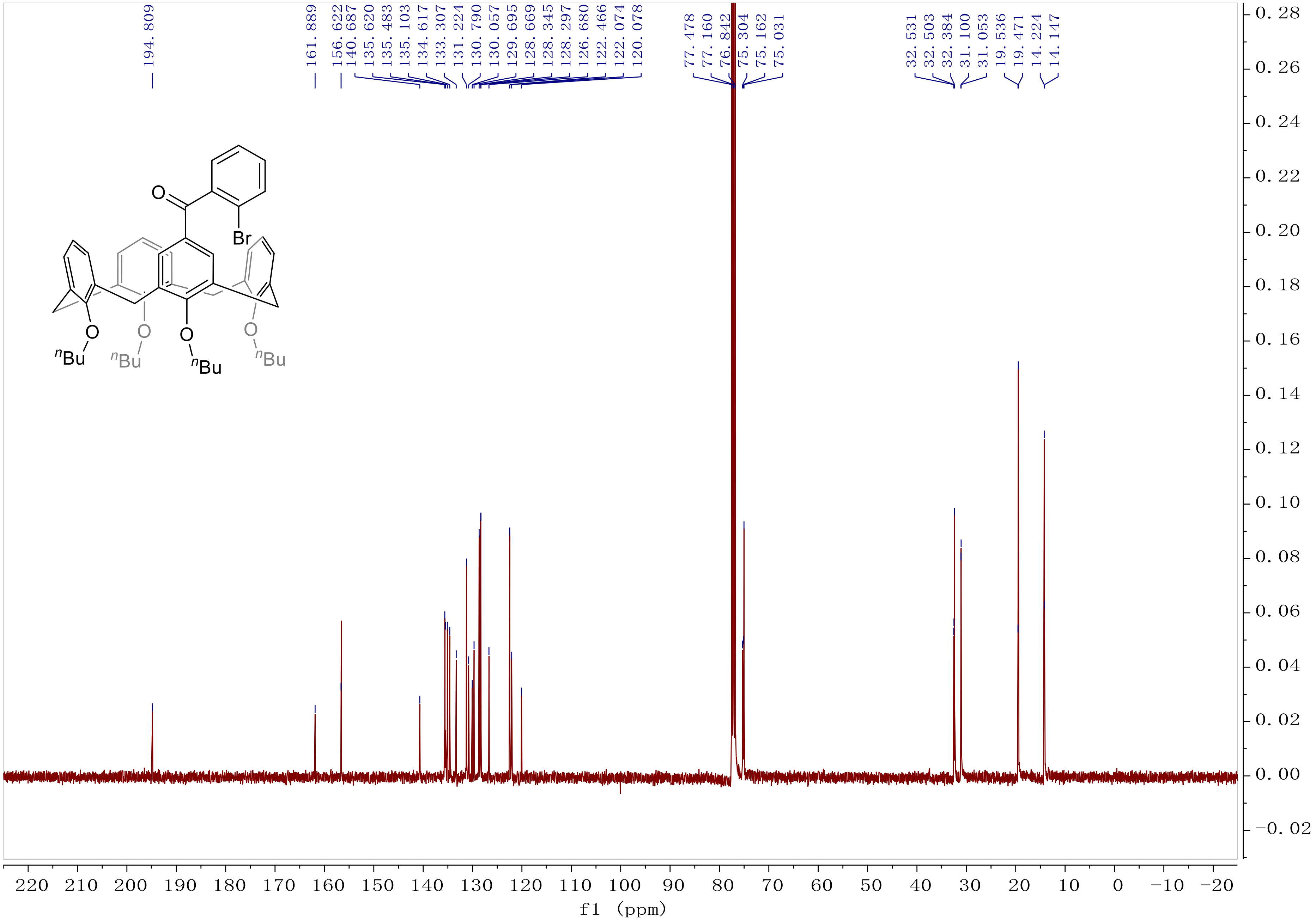

^{1}H NMR (400 MHz, 298 K) Spectra of **2b** in $CDCl_3$

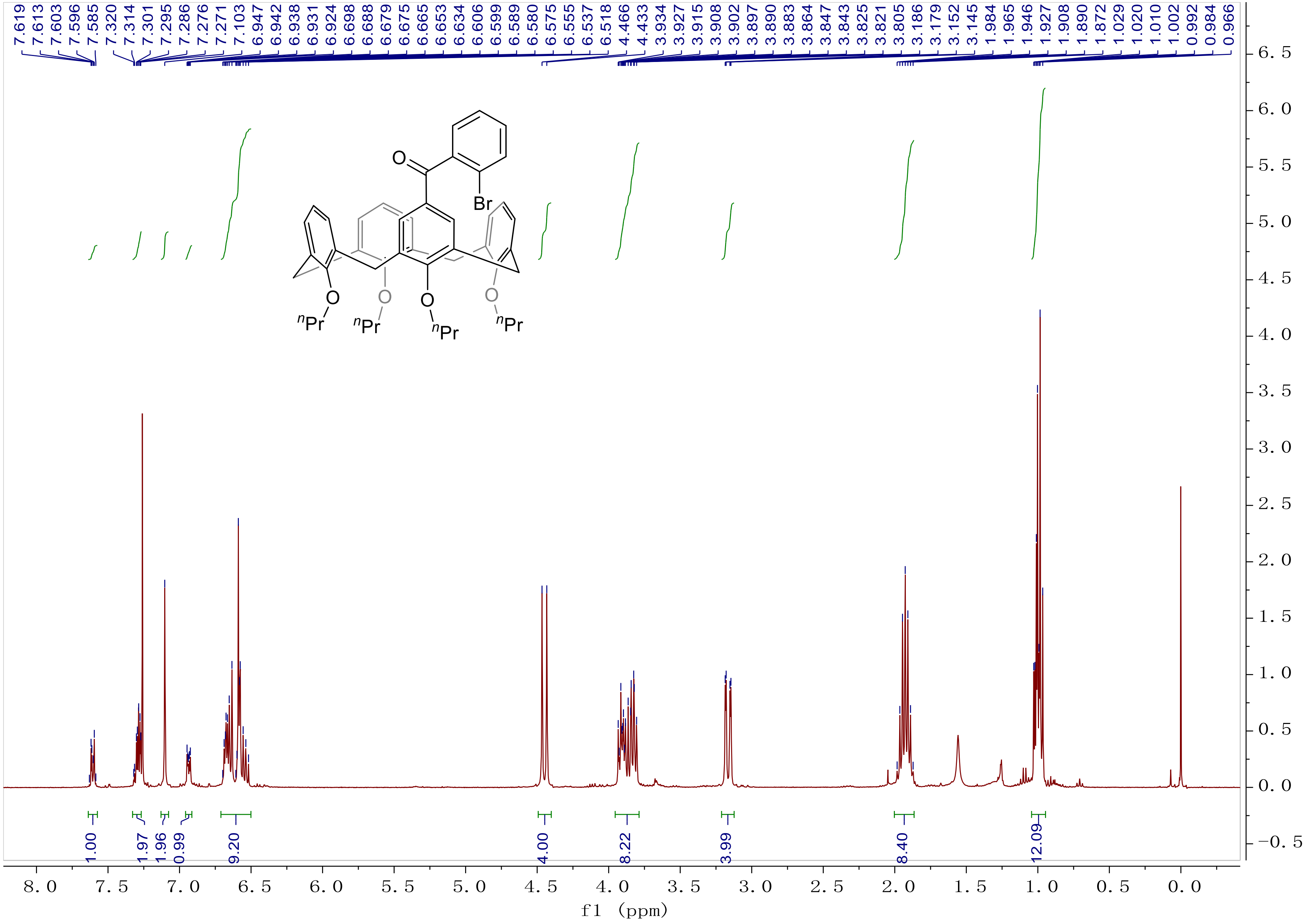

^{13}C NMR (100 MHz, 298 K) spectrum of **2b** in $CDCl_3$

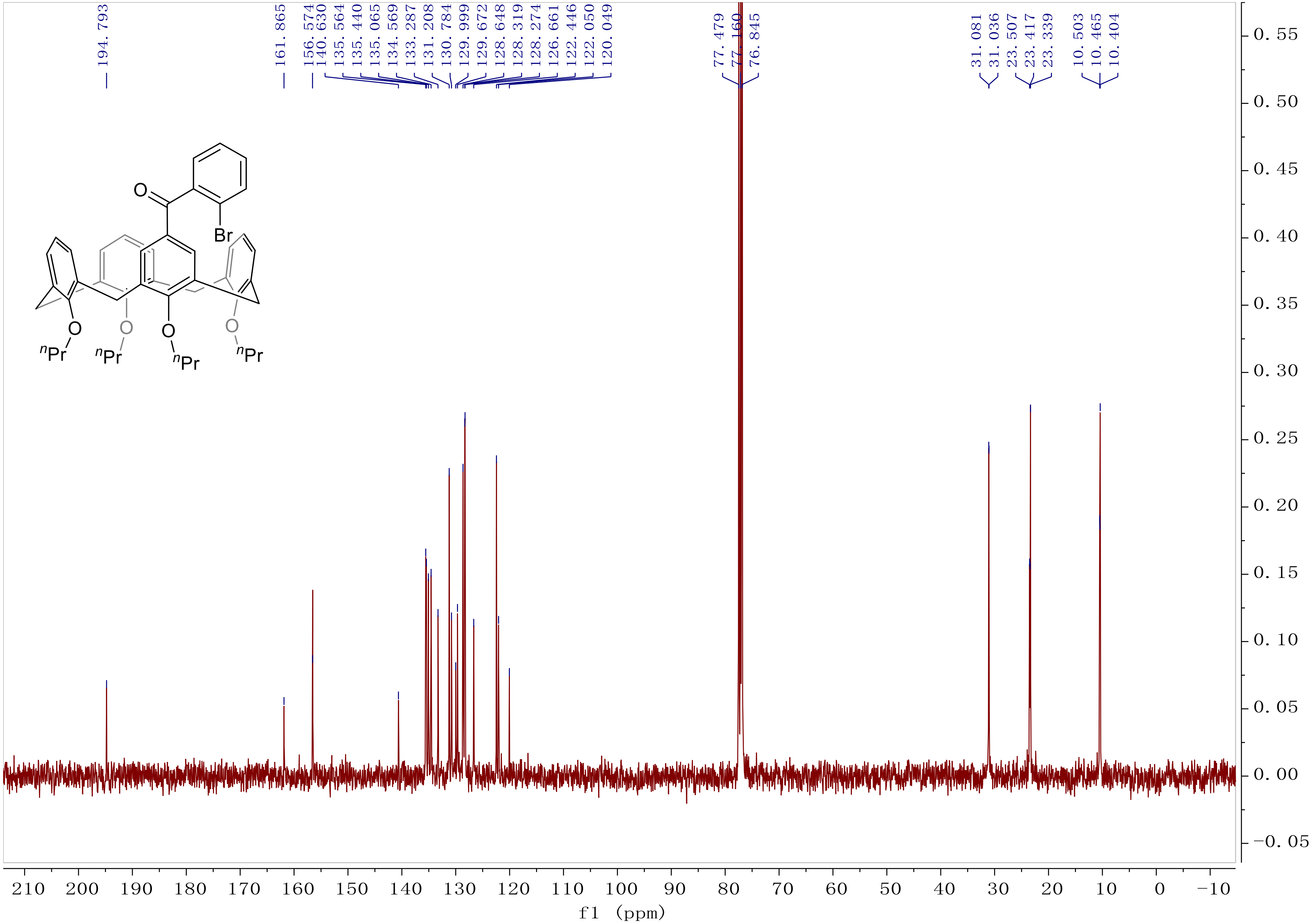

^{1}H NMR (400 MHz, 298 K) spectrum of **2c** in $CDCl_3$

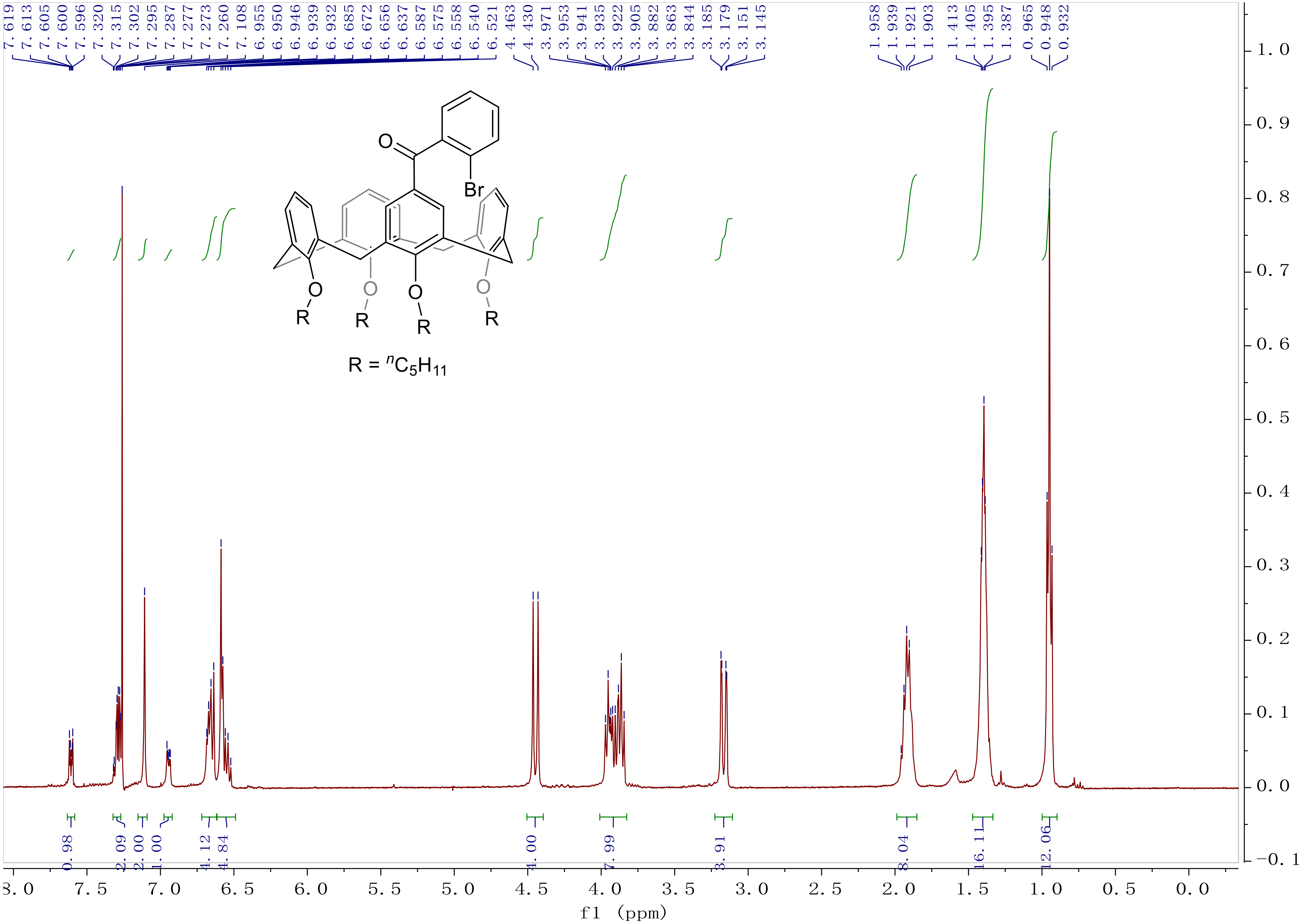

^{13}C NMR (100 MHz, 298 K) spectrum of **2c** in $CDCl_3$

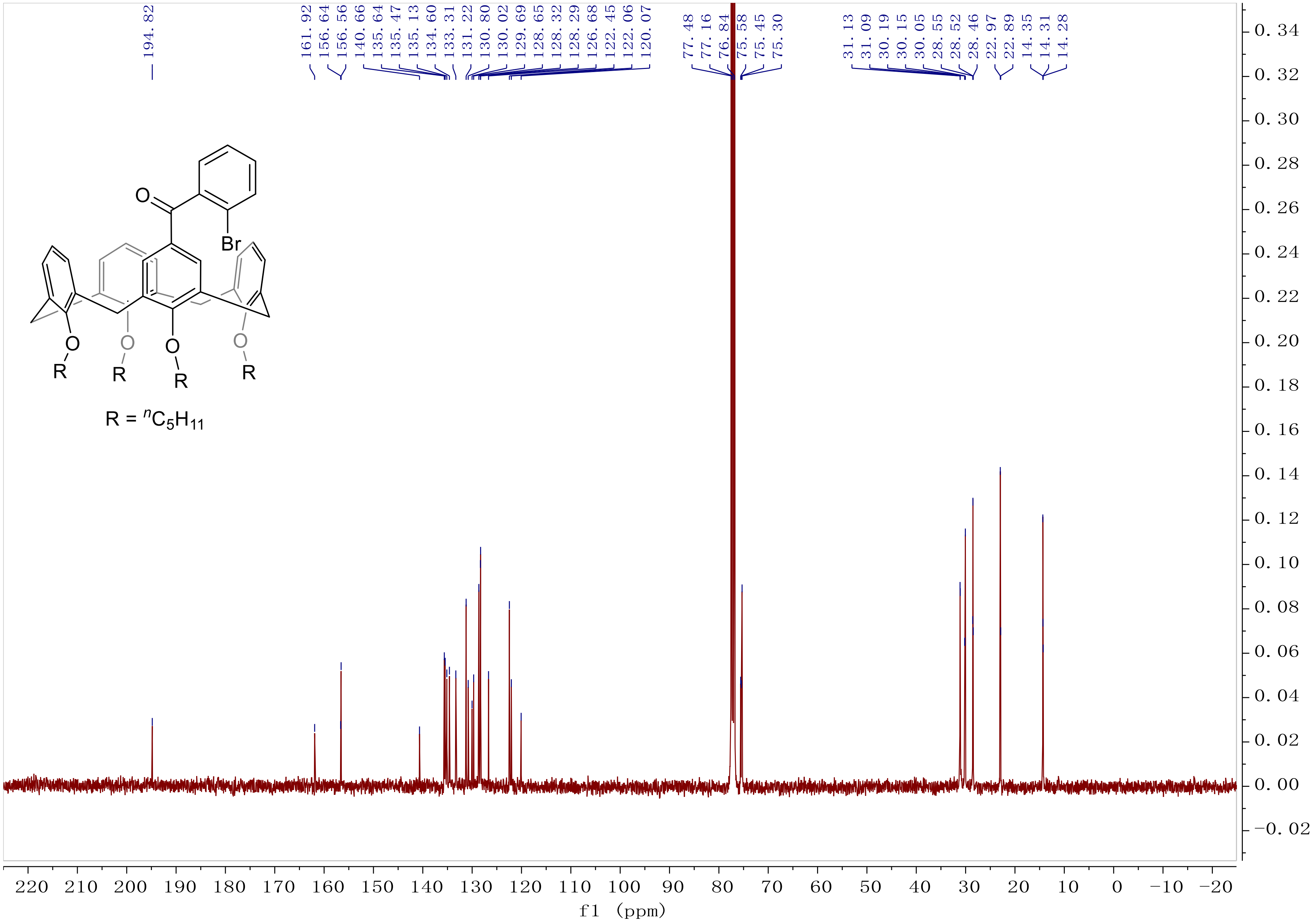

^{1}H NMR (400 MHz, 298 K) spectrum of **2d** in $CDCl_3$

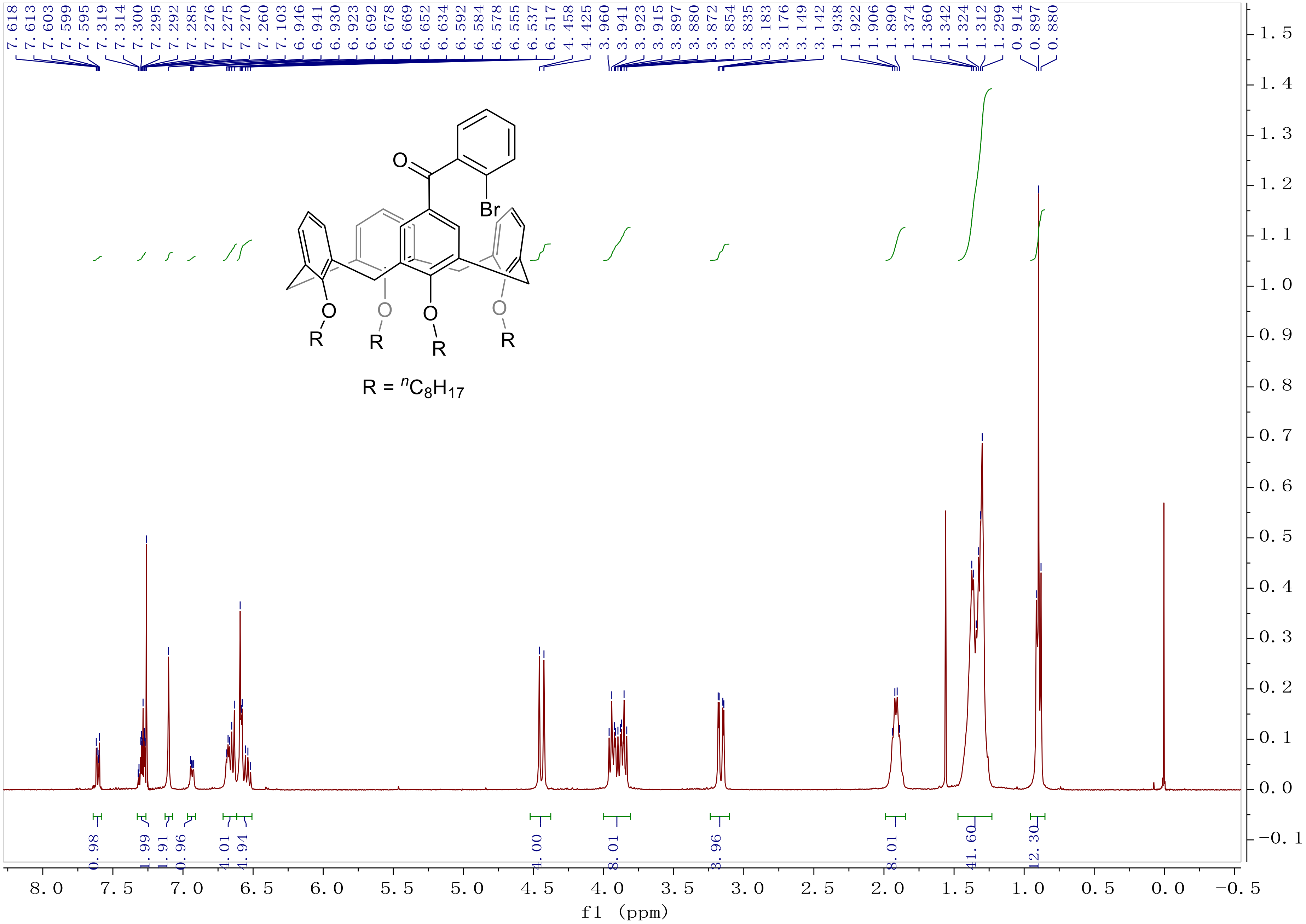

^{13}C NMR (100 MHz, 298 K) spectrum of **2d** in $CDCl_3$

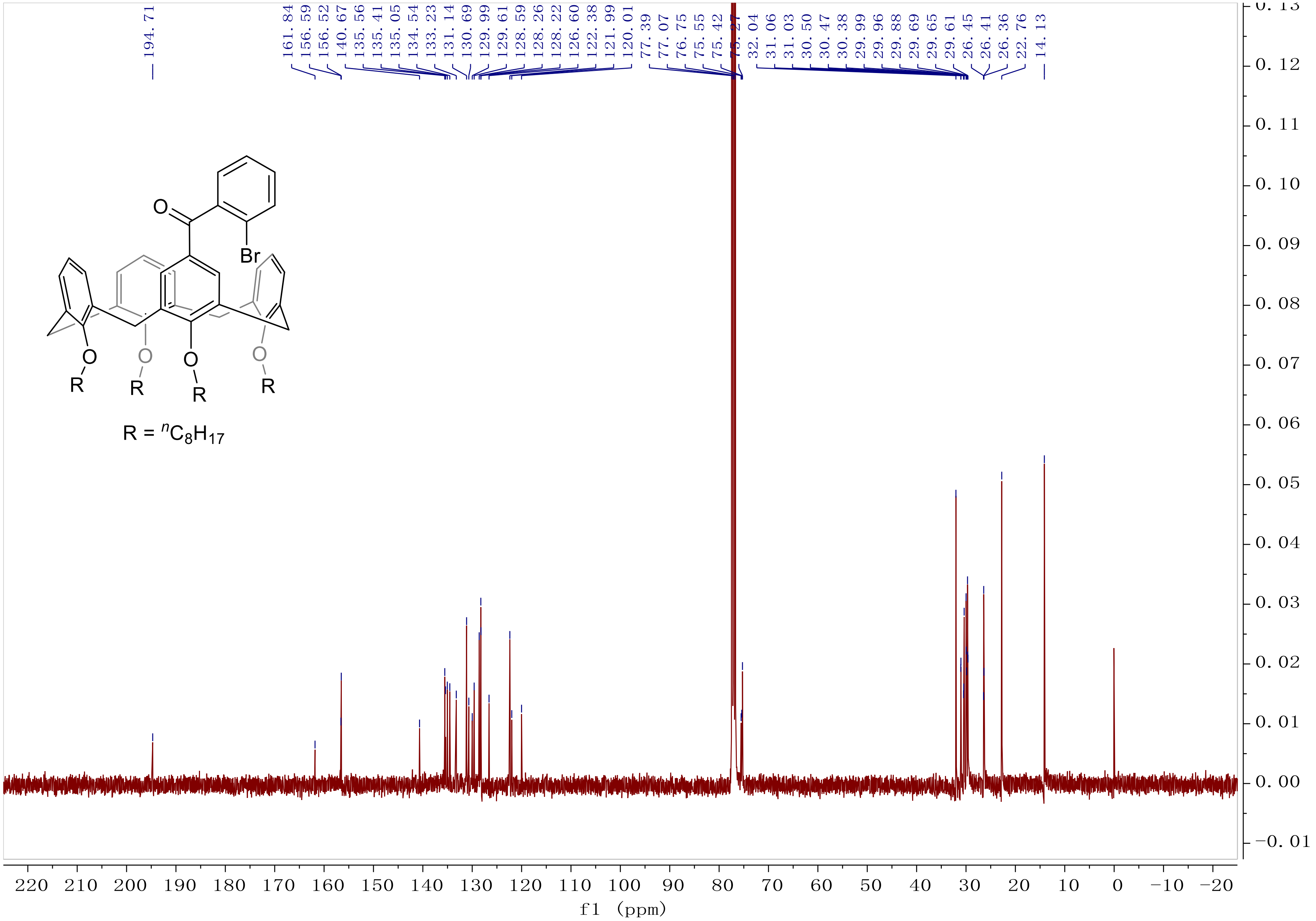

^{1}H NMR (400 MHz, 298 K) spectrum of **2e** in $CDCl_3$

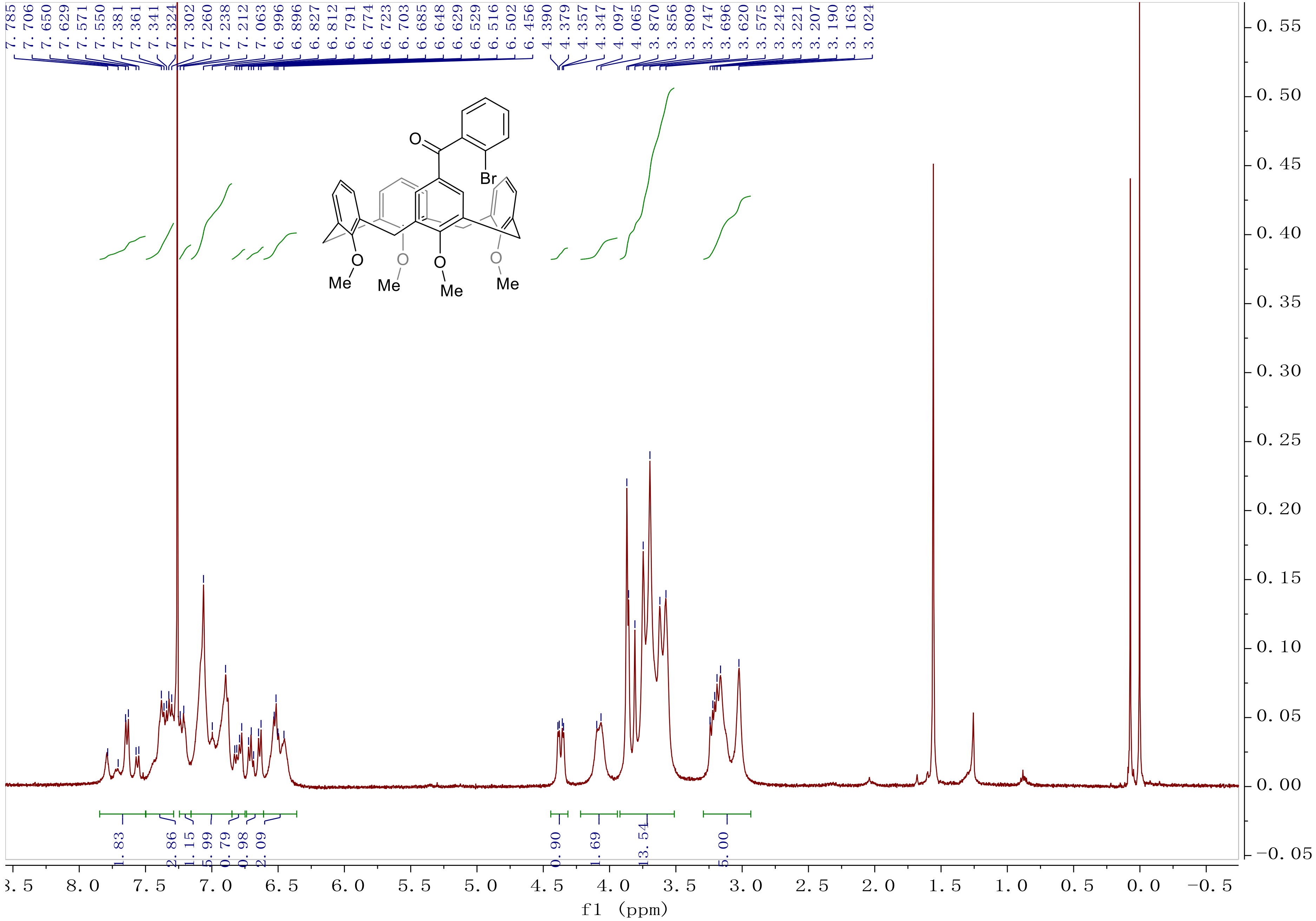

^{13}C NMR (100 MHz, 298 K) spectrum of **2e** in $CDCl_3$

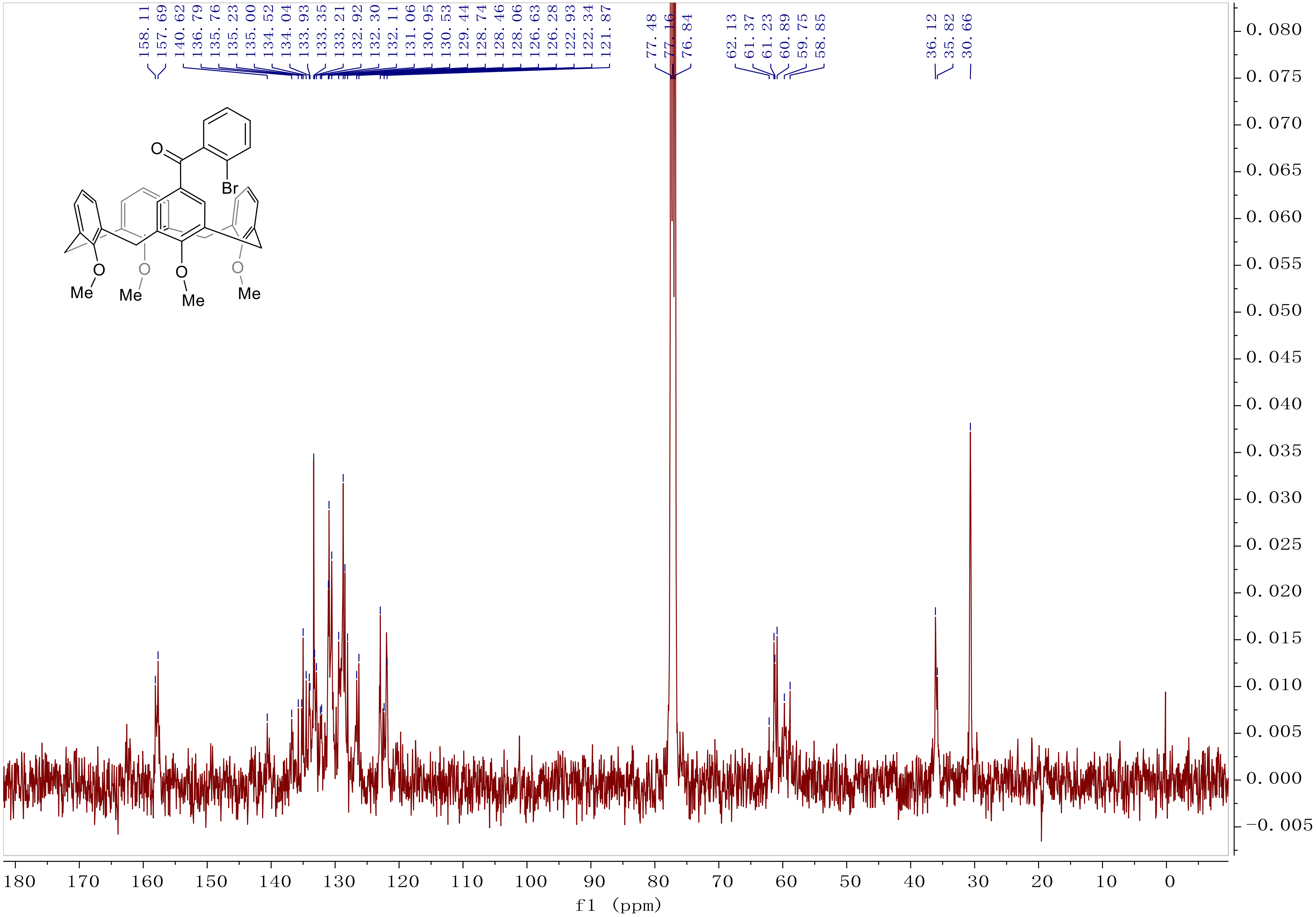

^{1}H NMR (400 MHz, 298 K) spectrum of **2f** in $CDCl_3$

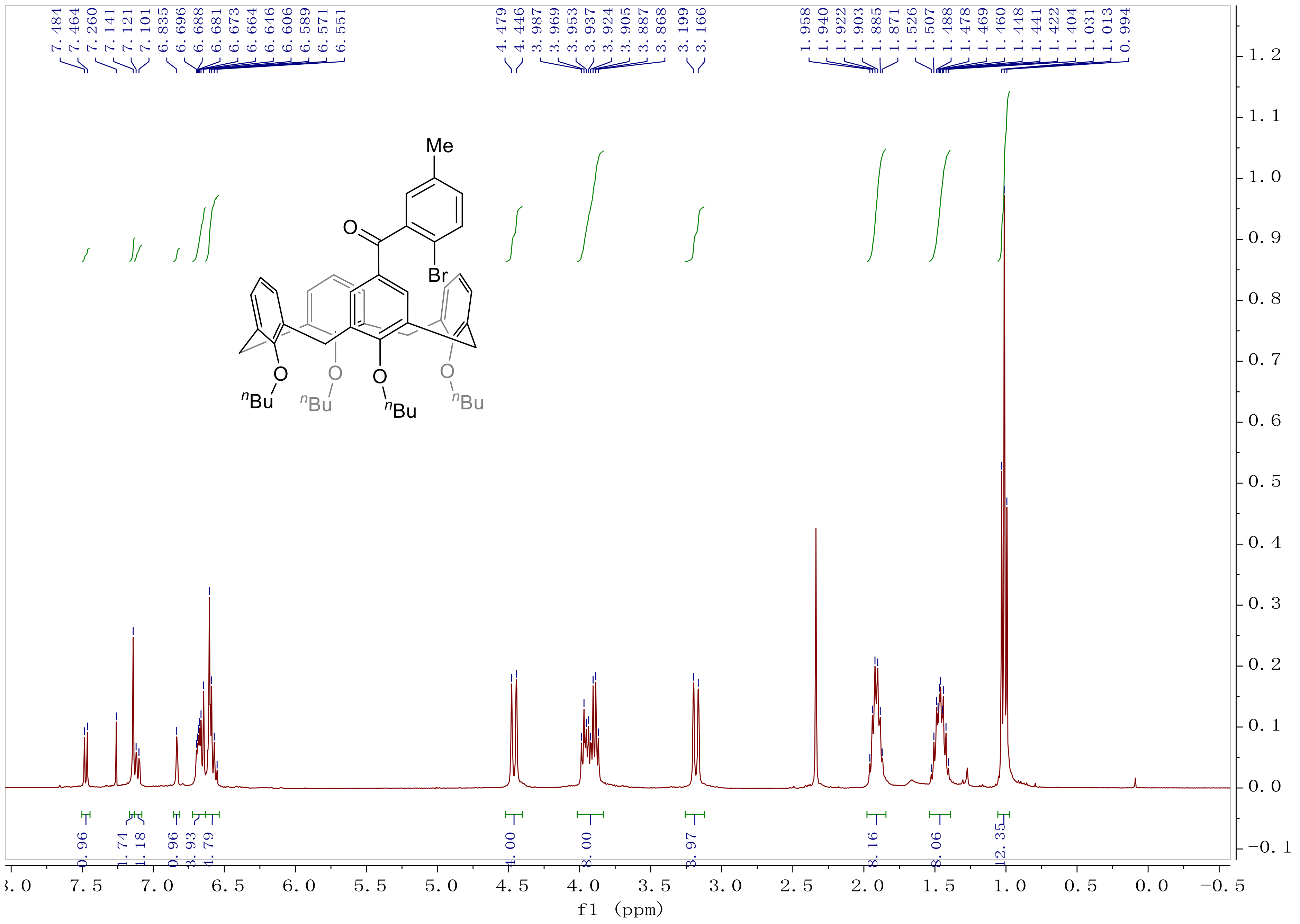

^{13}C NMR (100 MHz, 298 K) spectrum of **2f** in $CDCl_3$

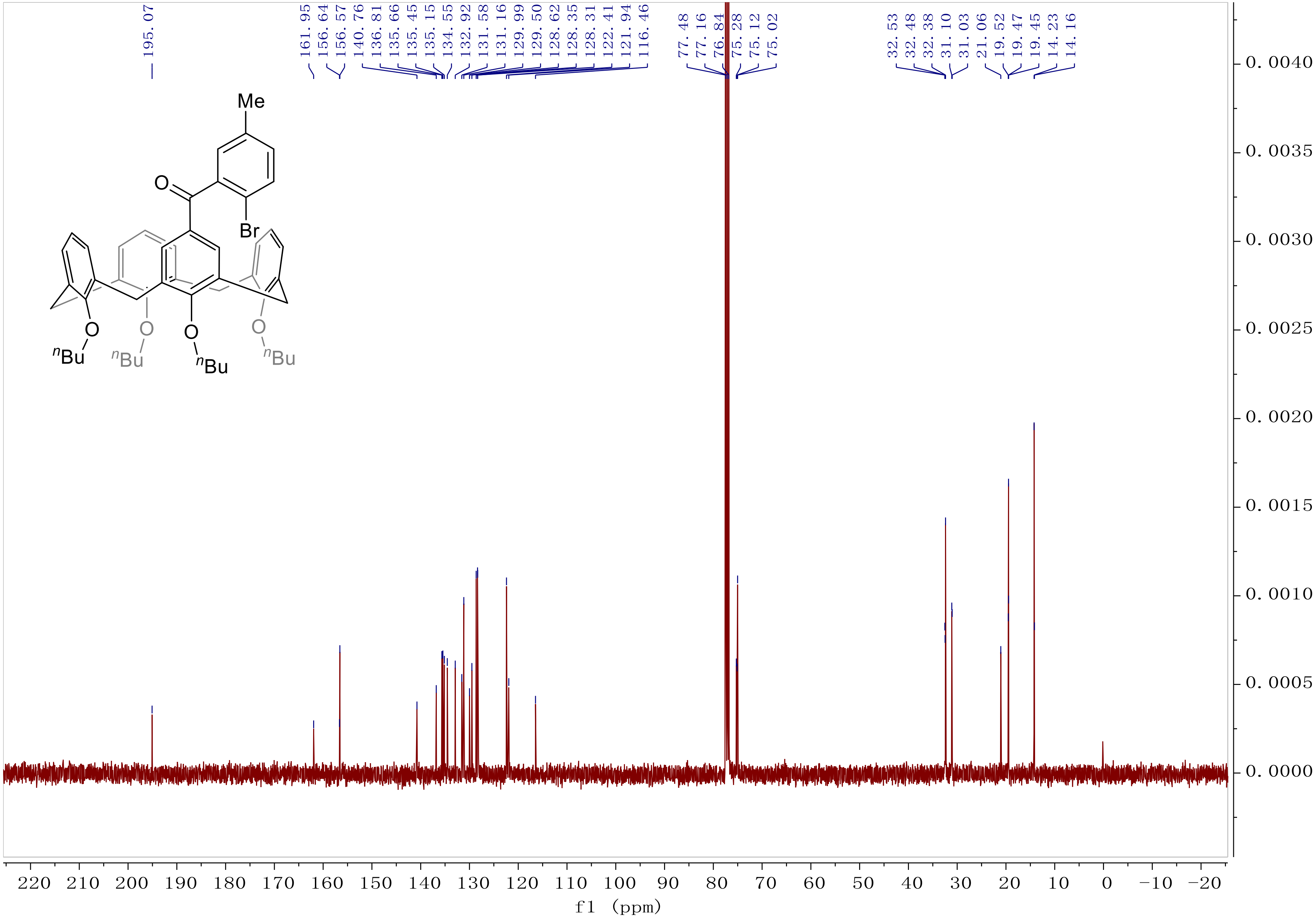

^{1}H NMR (400 MHz, 298 K) spectrum of **2g** in $CDCl_3$

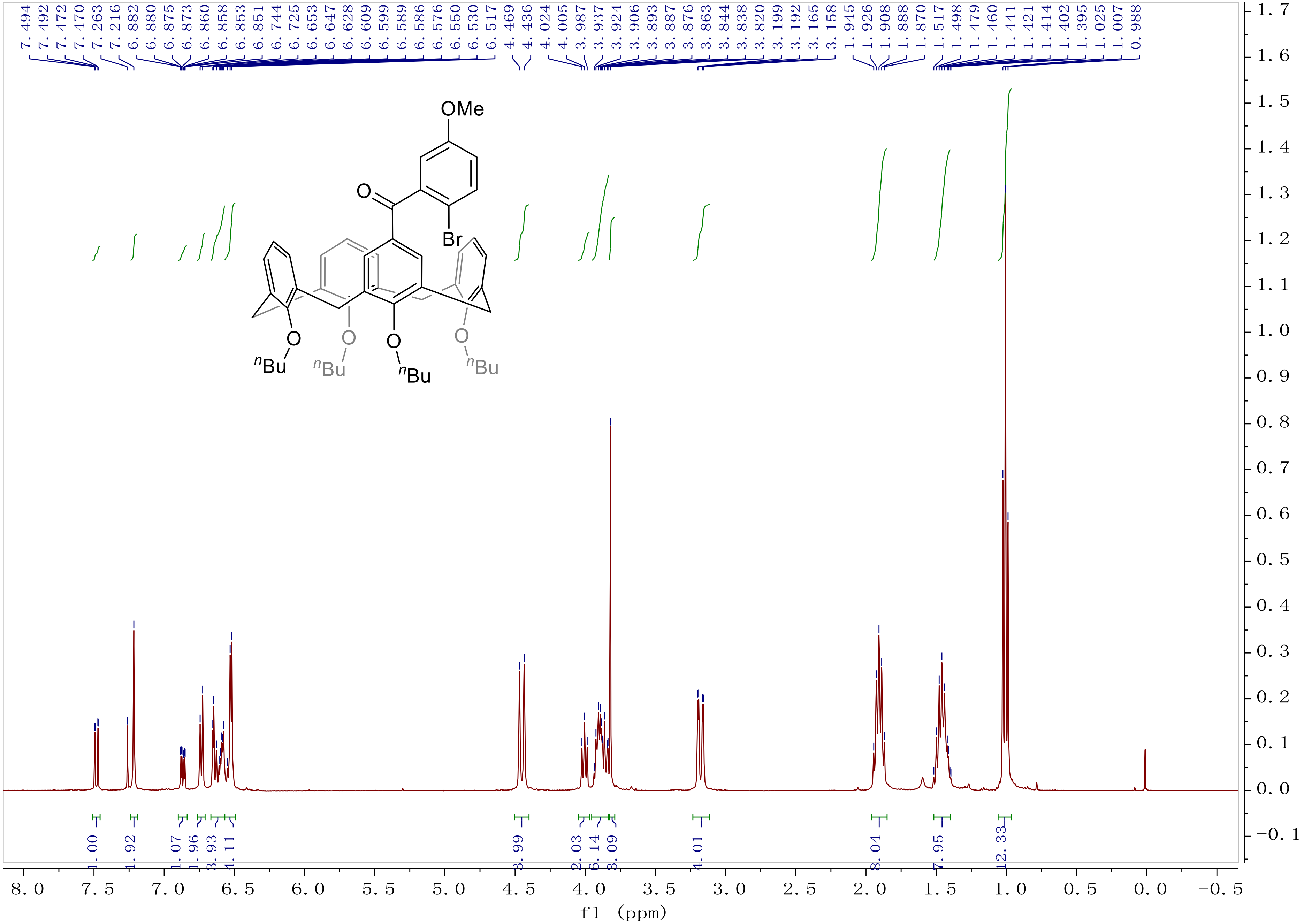

^{13}C NMR (100 MHz, 298 K) spectrum of **2g** in $CDCl_3$

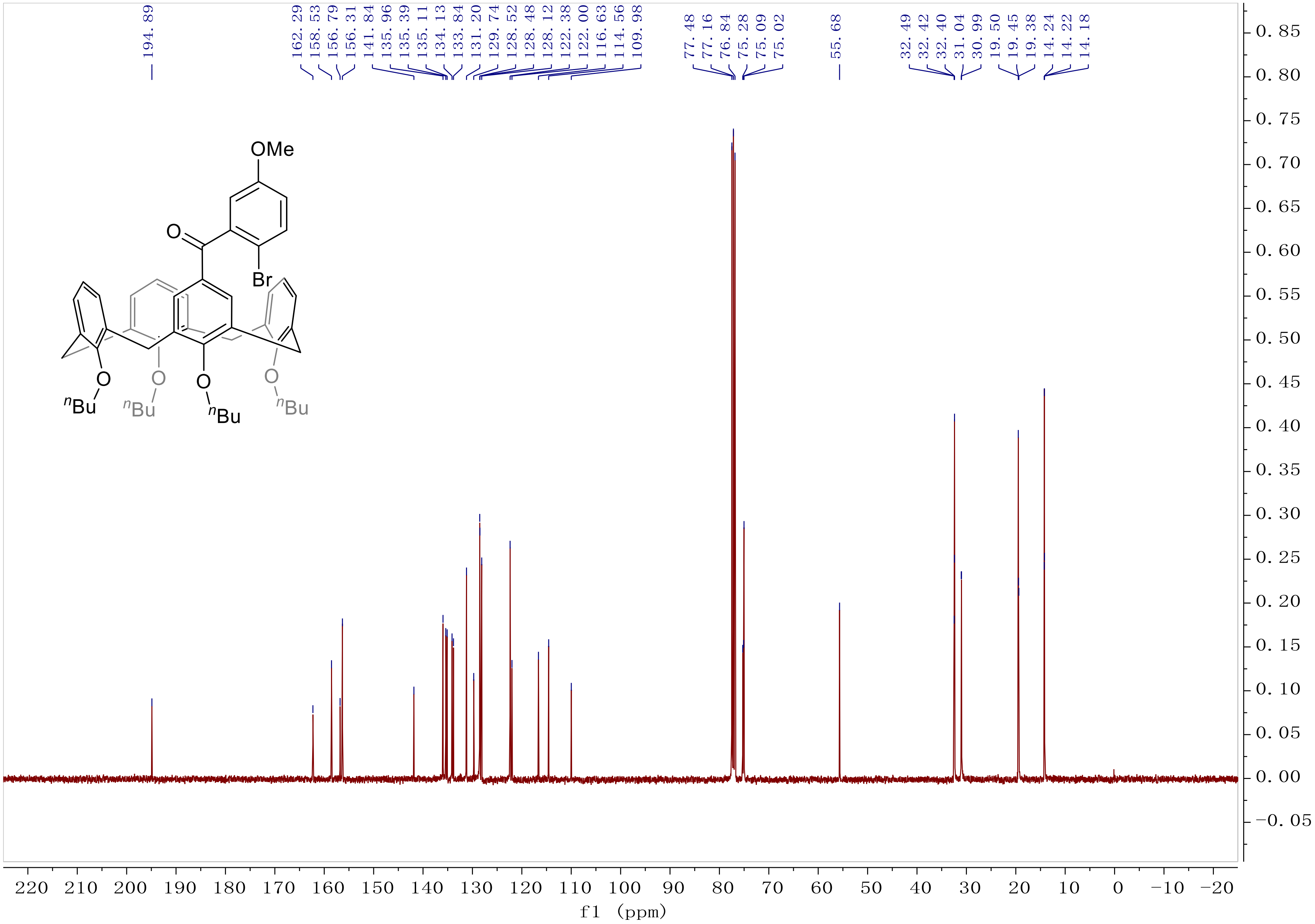

^{1}H NMR (400 MHz, 298 K) spectrum of **2h** in $CDCl_3$

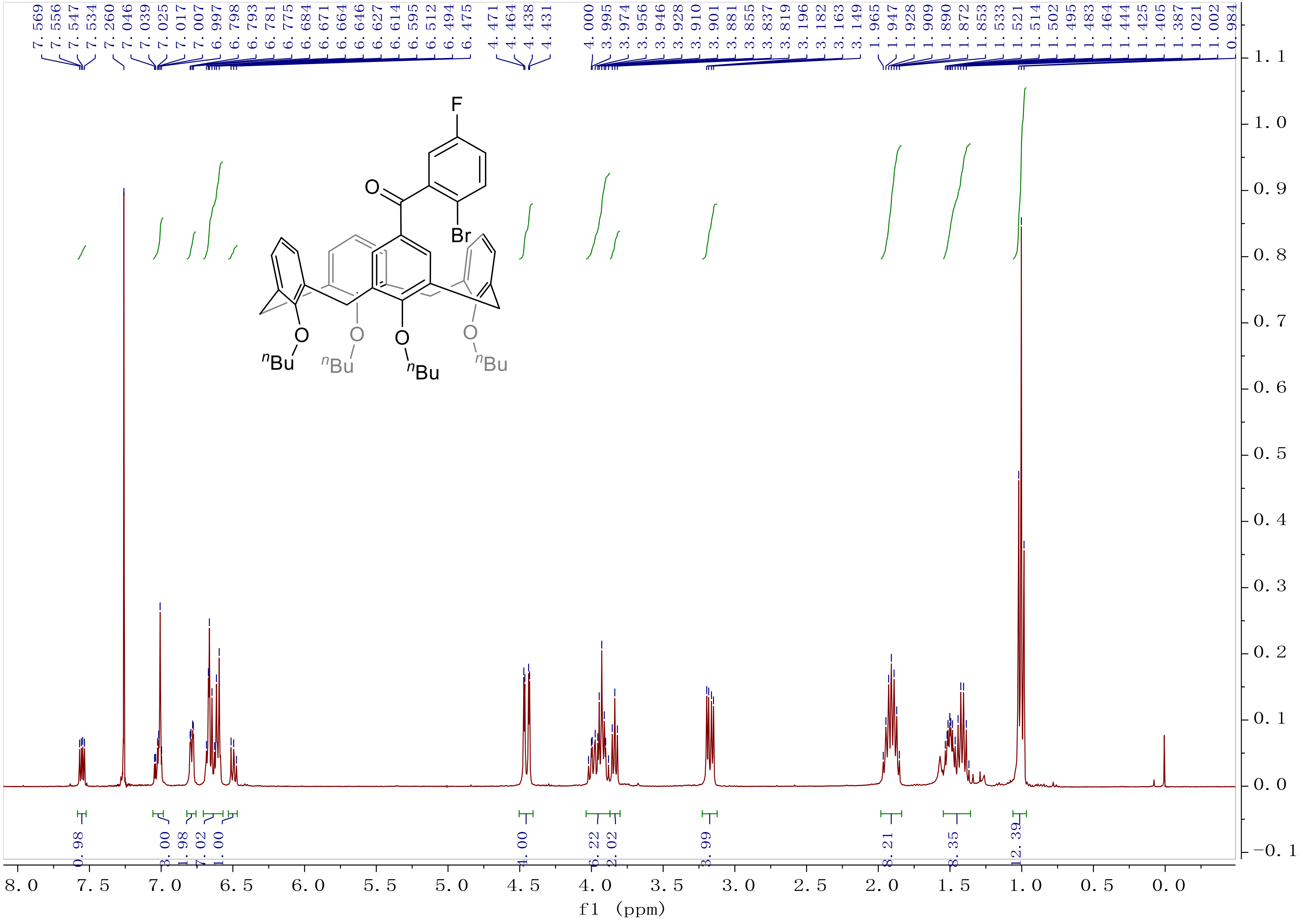

^{13}C NMR (100 MHz, 298 K) spectrum of **2h** in $CDCl_3$

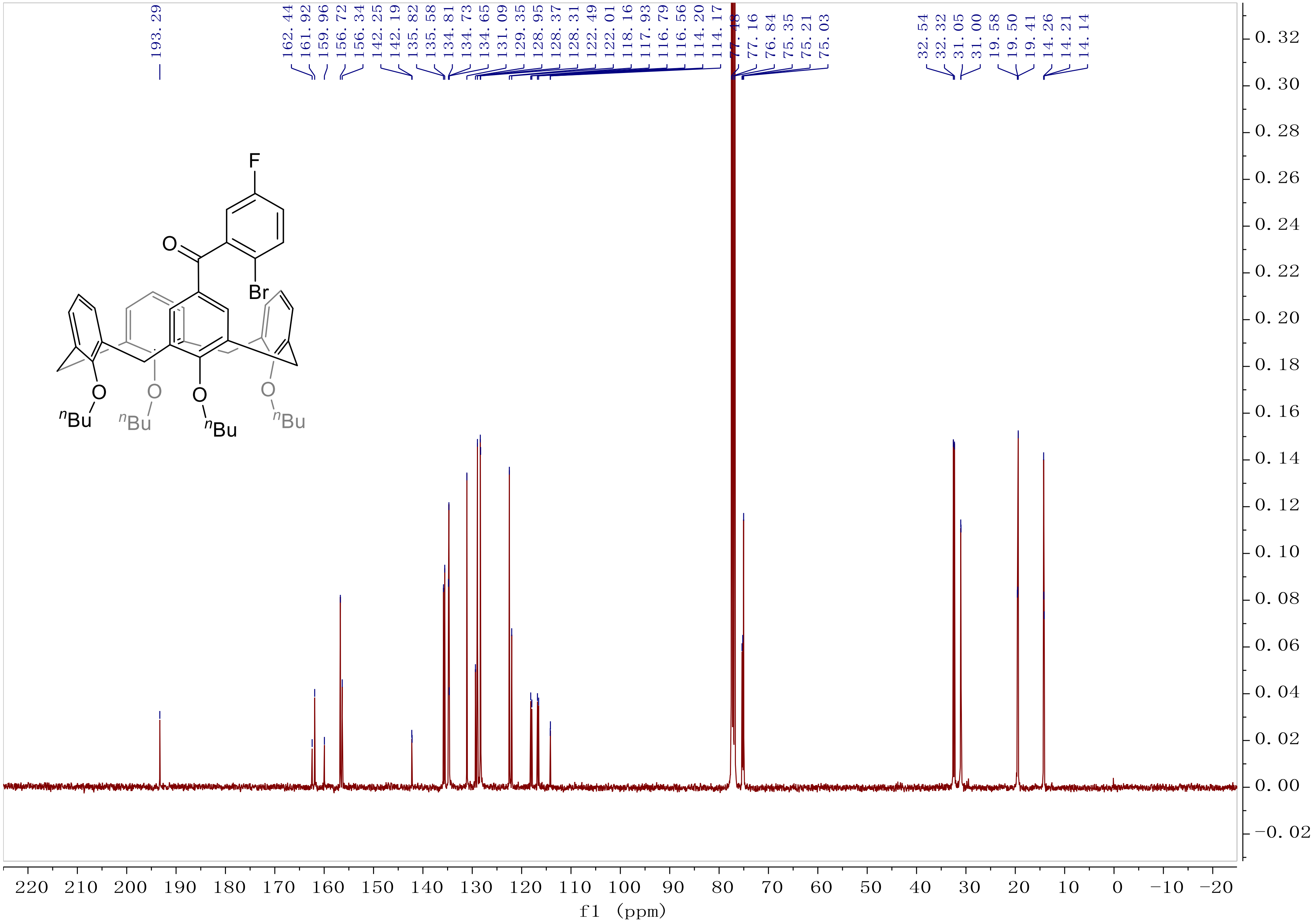

^{1}H NMR (400 MHz, 298 K) spectrum of **2i** in $CDCl_3$

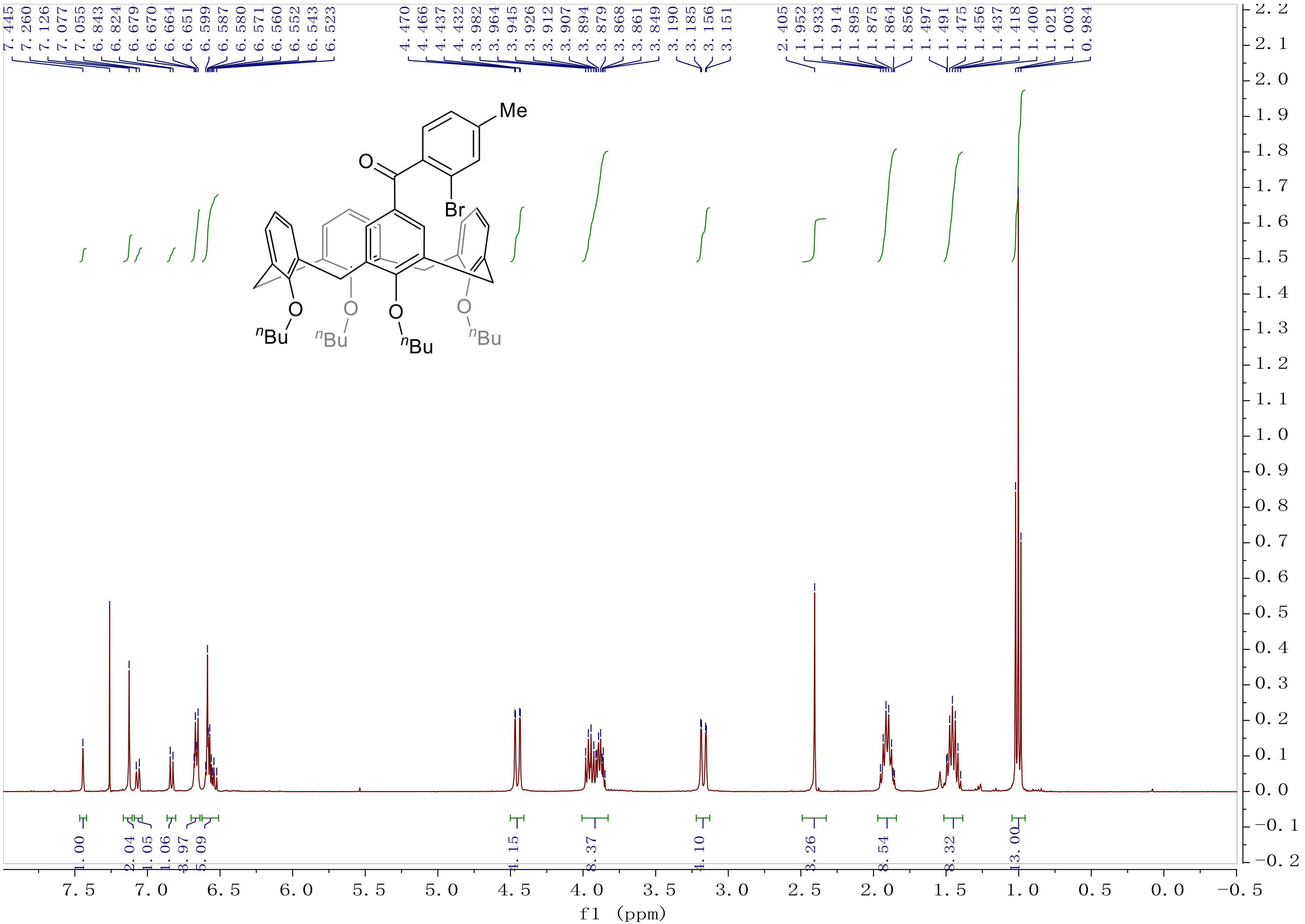

^{13}C NMR (100 MHz, 298 K) spectrum of **2i** in $CDCl_3$

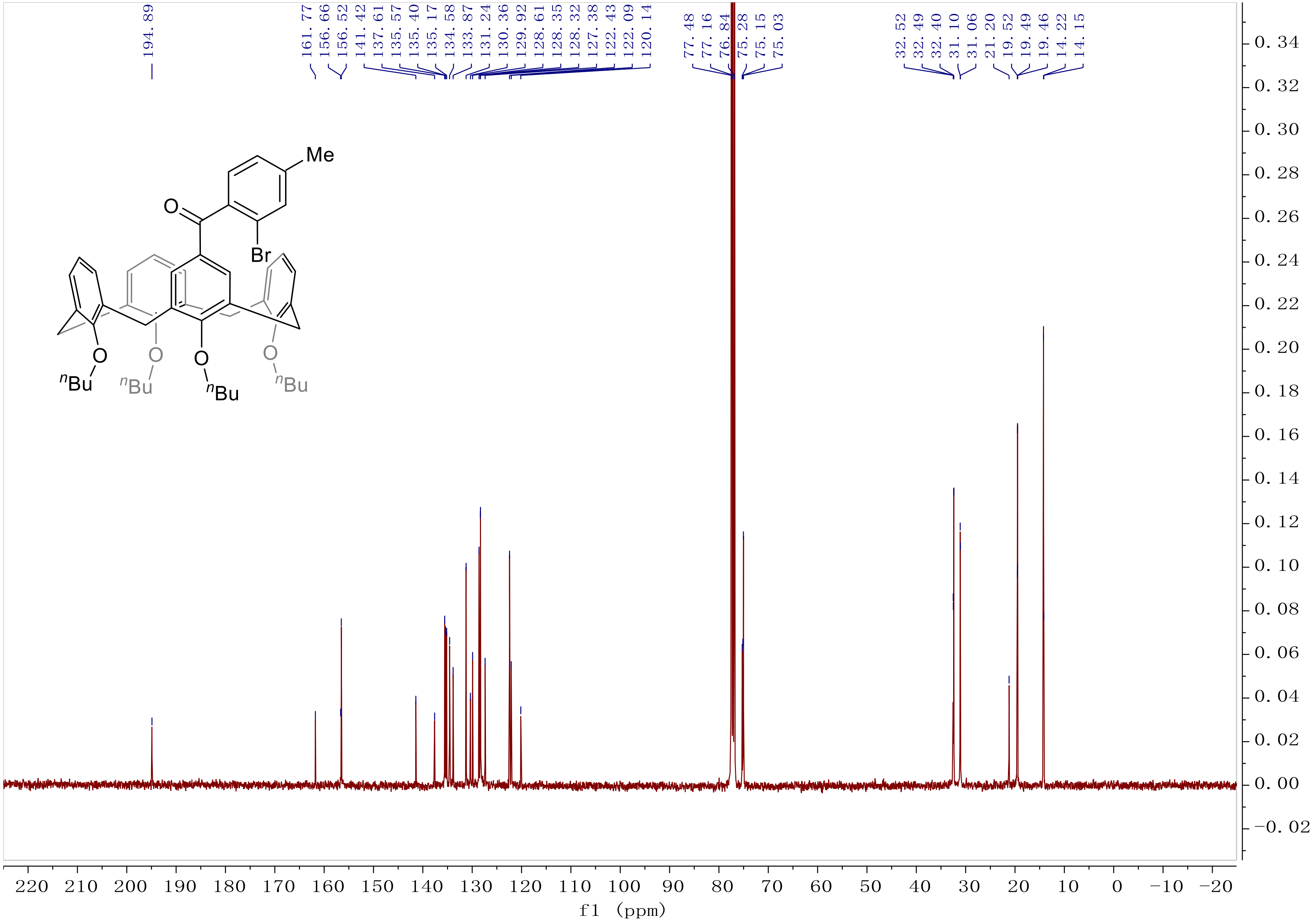

^1H NMR (400 MHz, 298 K) spectrum of **2j** in $CDCl_3$

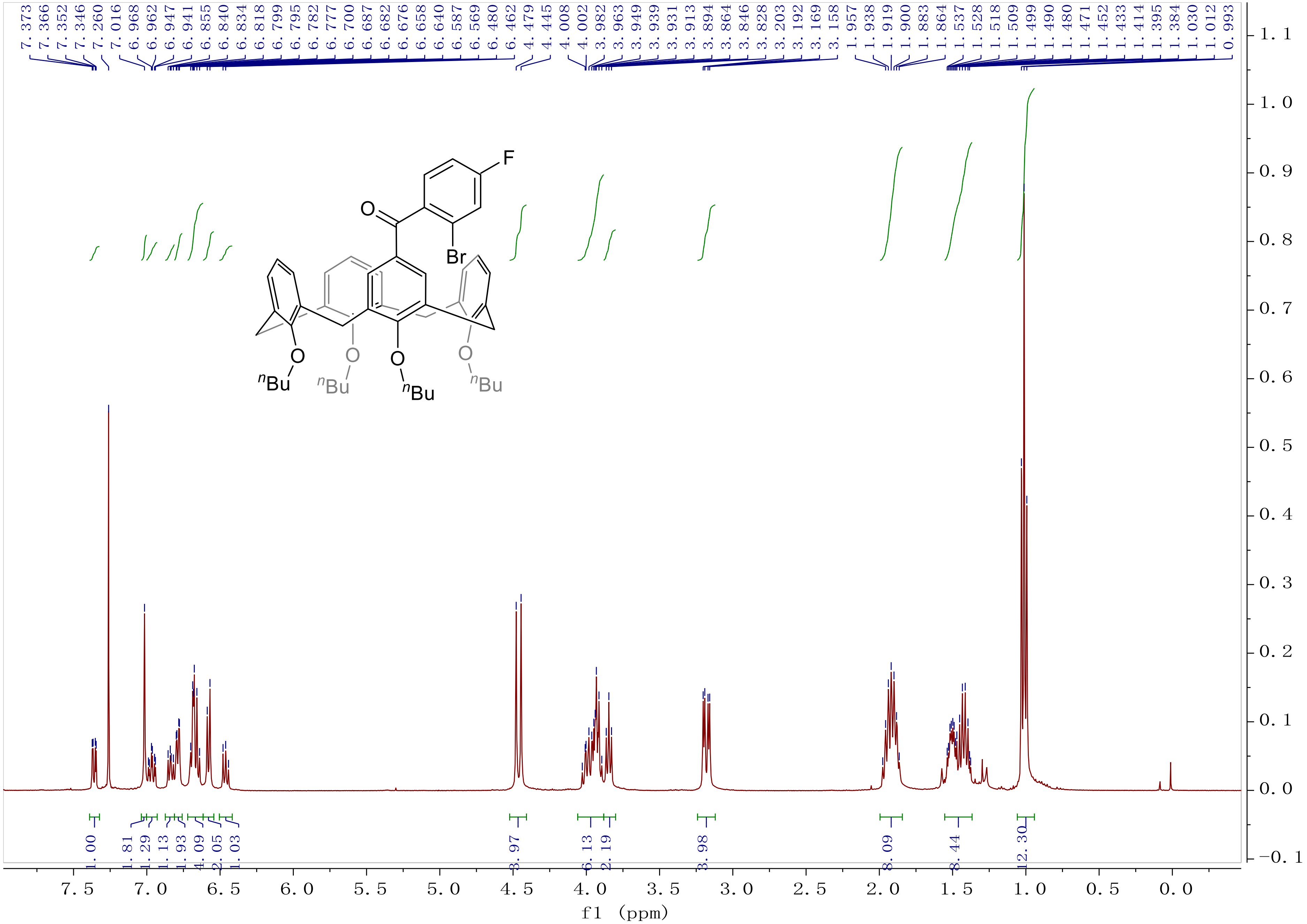

^{13}C NMR (100 MHz, 298 K) spectrum of **2j** in $CDCl_3$

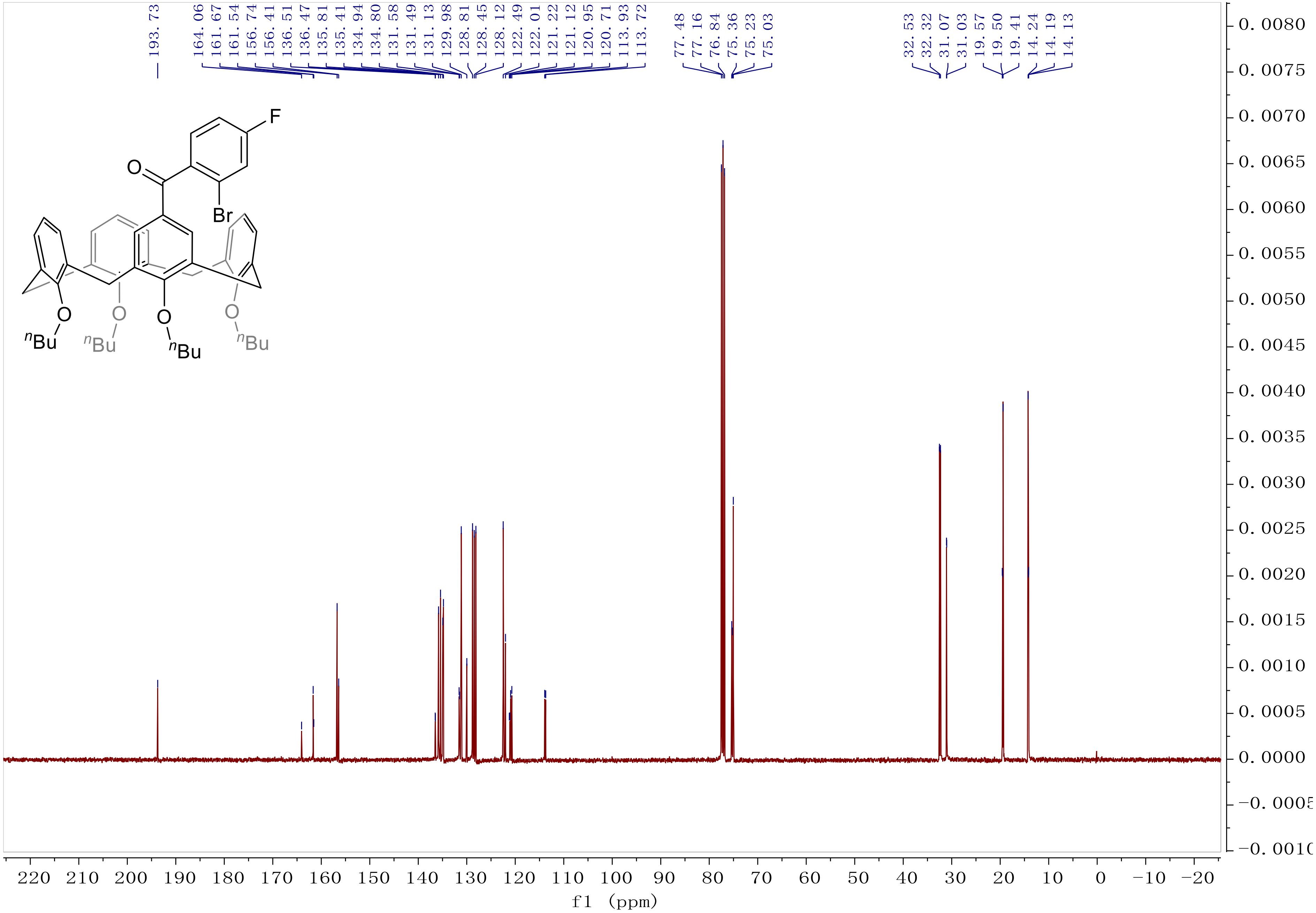

^1H NMR (400 MHz, 298 K) spectrum of **2k** in $CDCl_3$

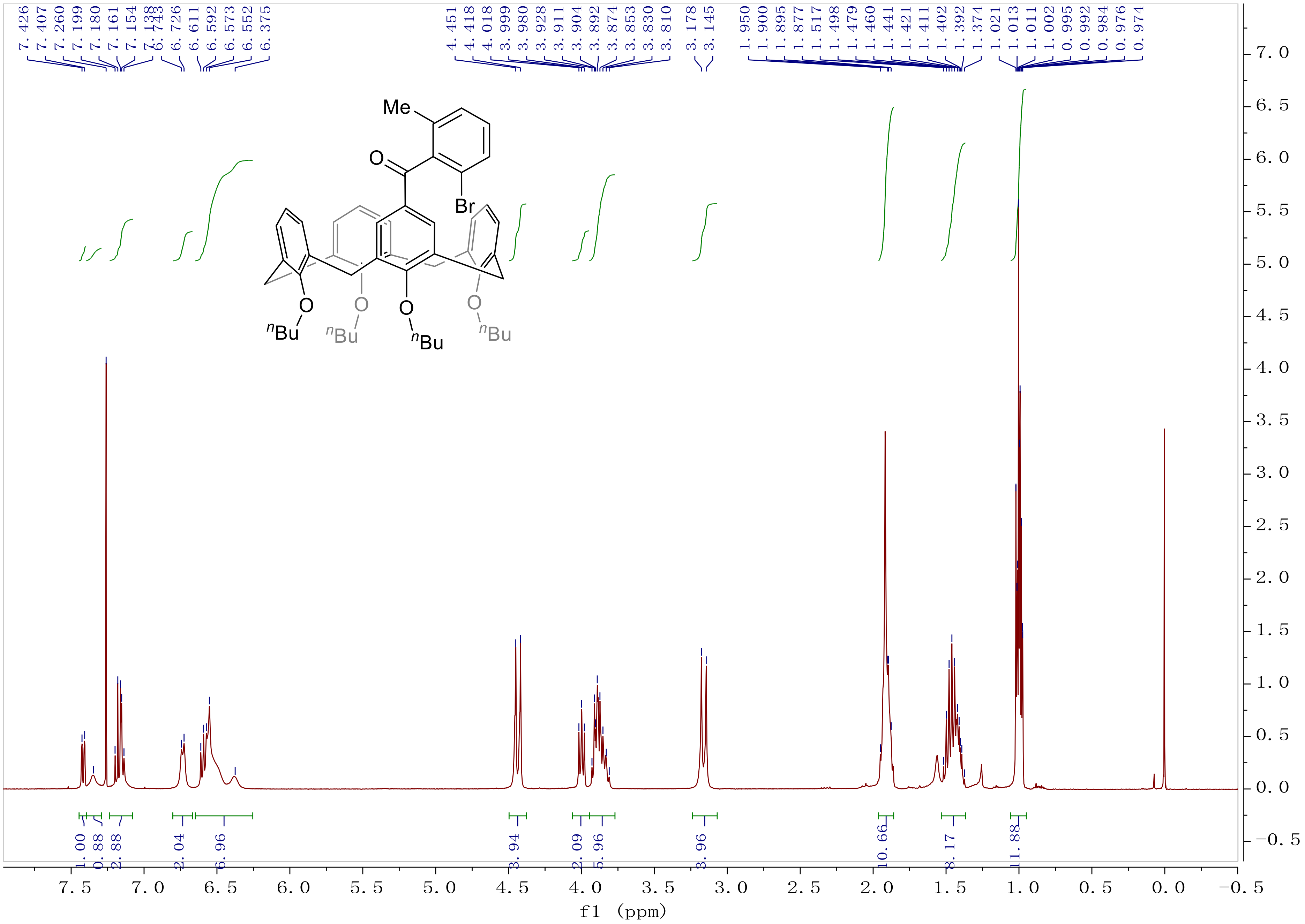

^{13}C NMR (100 MHz, 298 K) spectrum of **2k** in $CDCl_3$

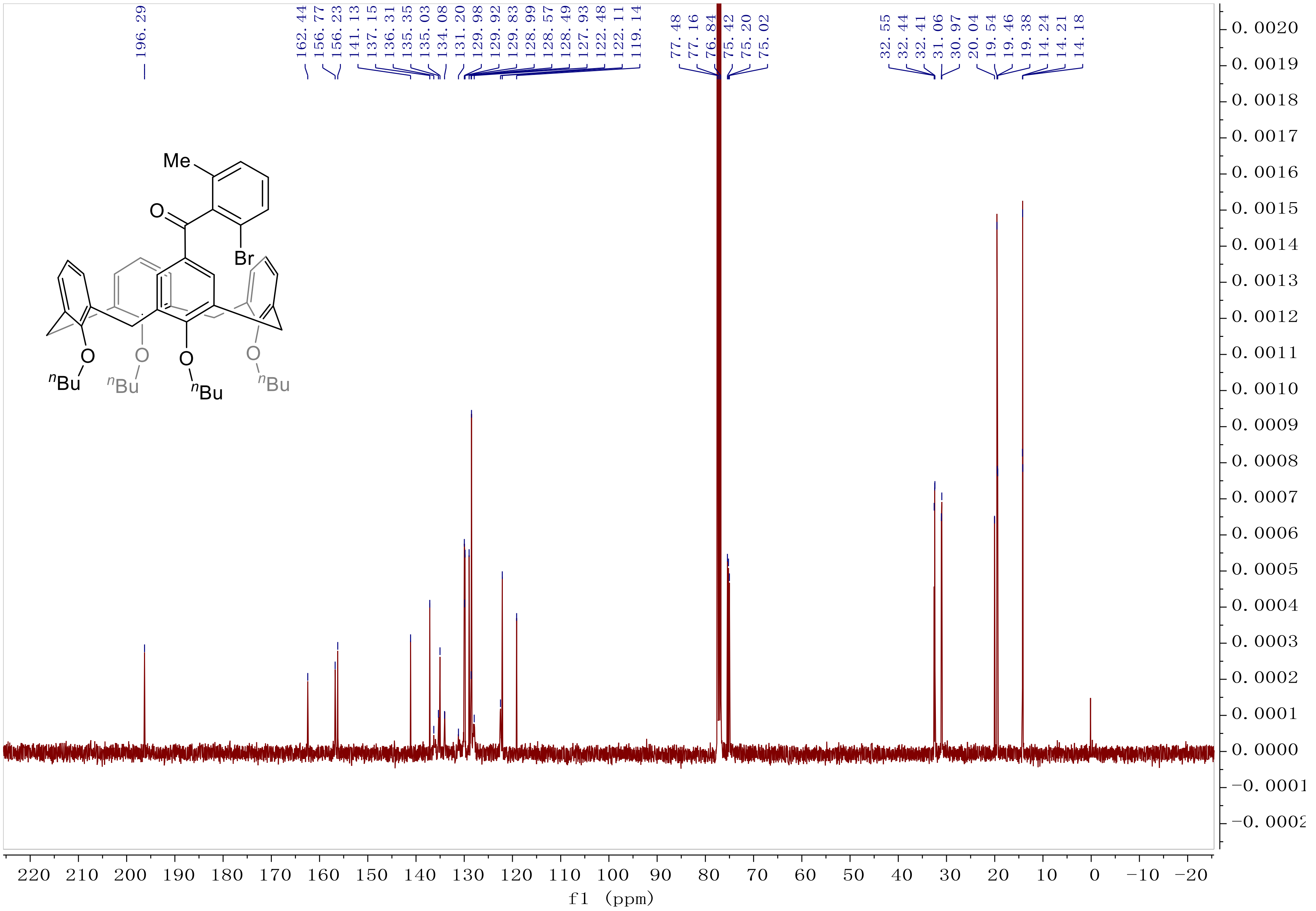

^{1}H NMR (400 MHz, 298 K) spectrum of **3a** in $CDCl_3$

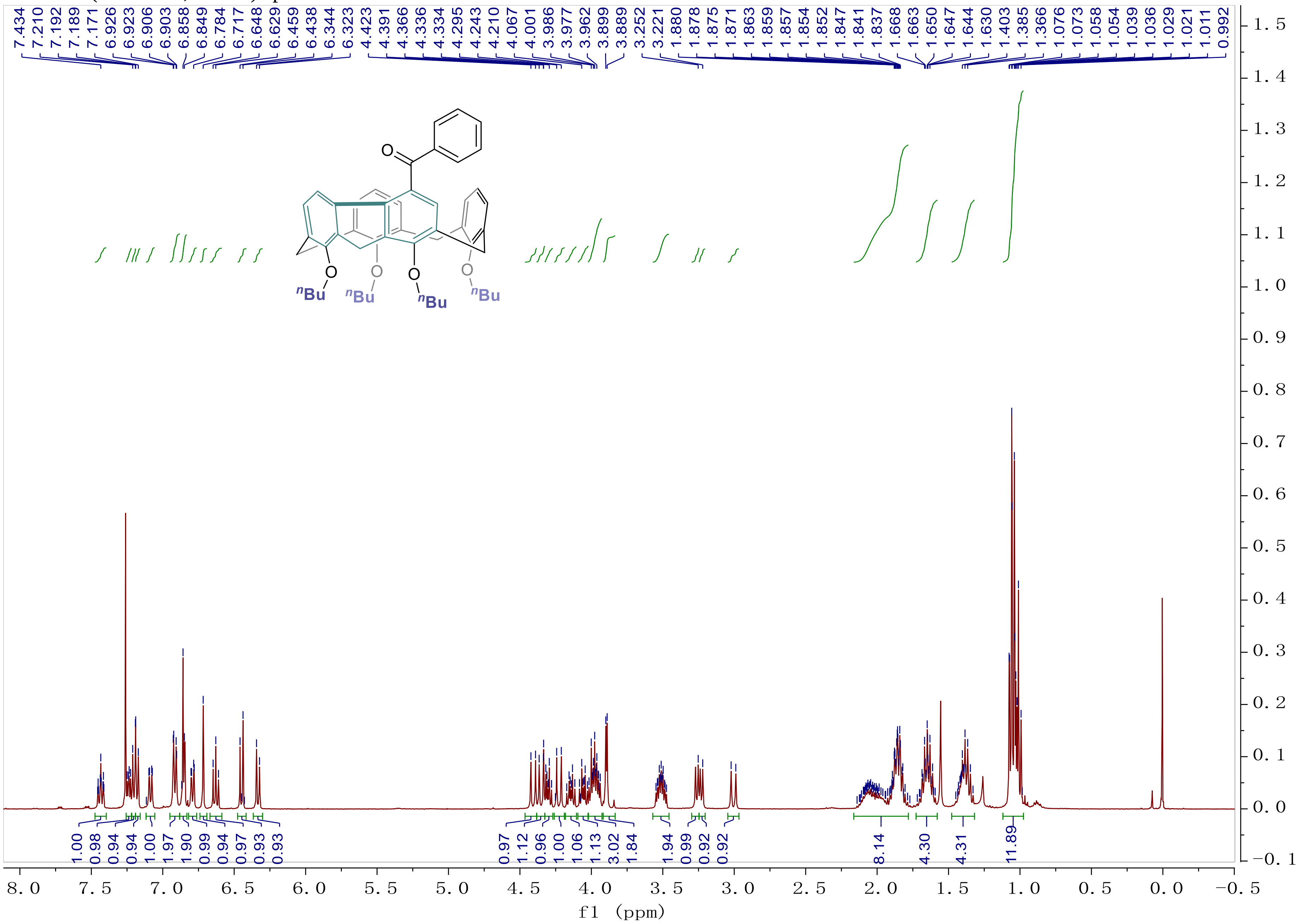

^{13}C NMR (100 MHz, 298 K) spectrum of **3a** in $CDCl_3$

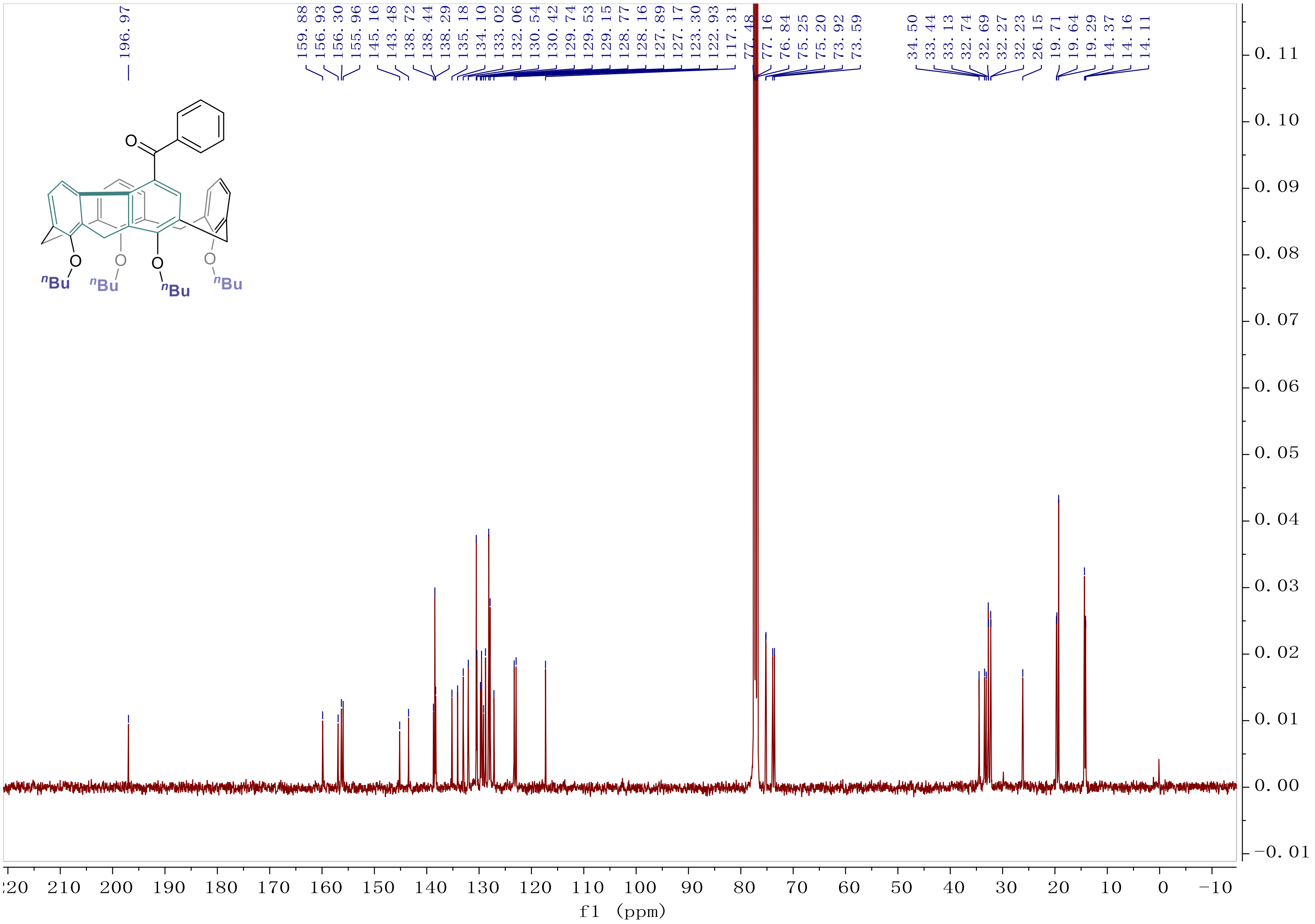

^{1}H NMR (400 MHz, 298 K) spectrum of **3b** in $CDCl_3$

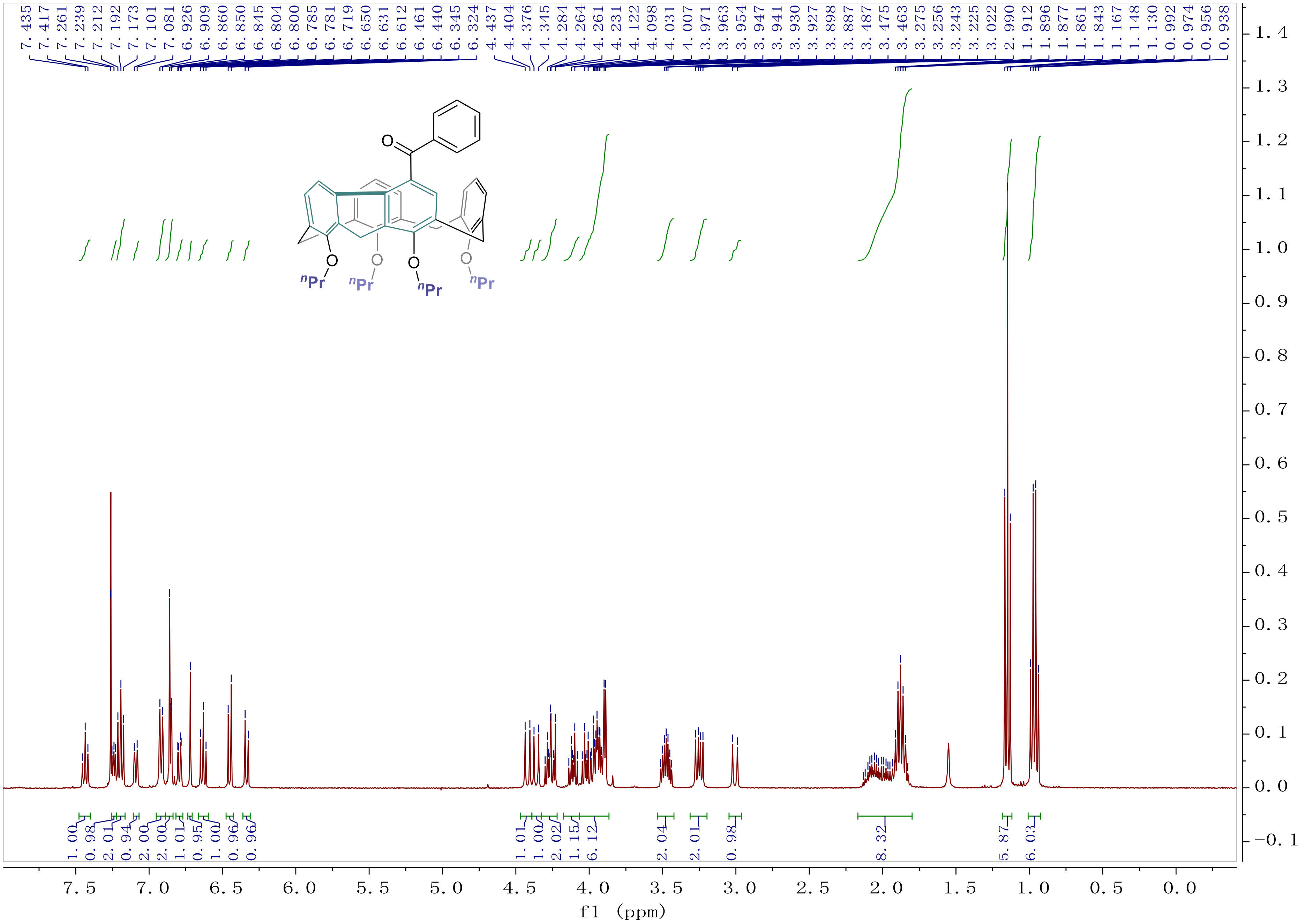

^{13}C NMR (100 MHz, 298 K) spectrum of **3b** in $CDCl_3$

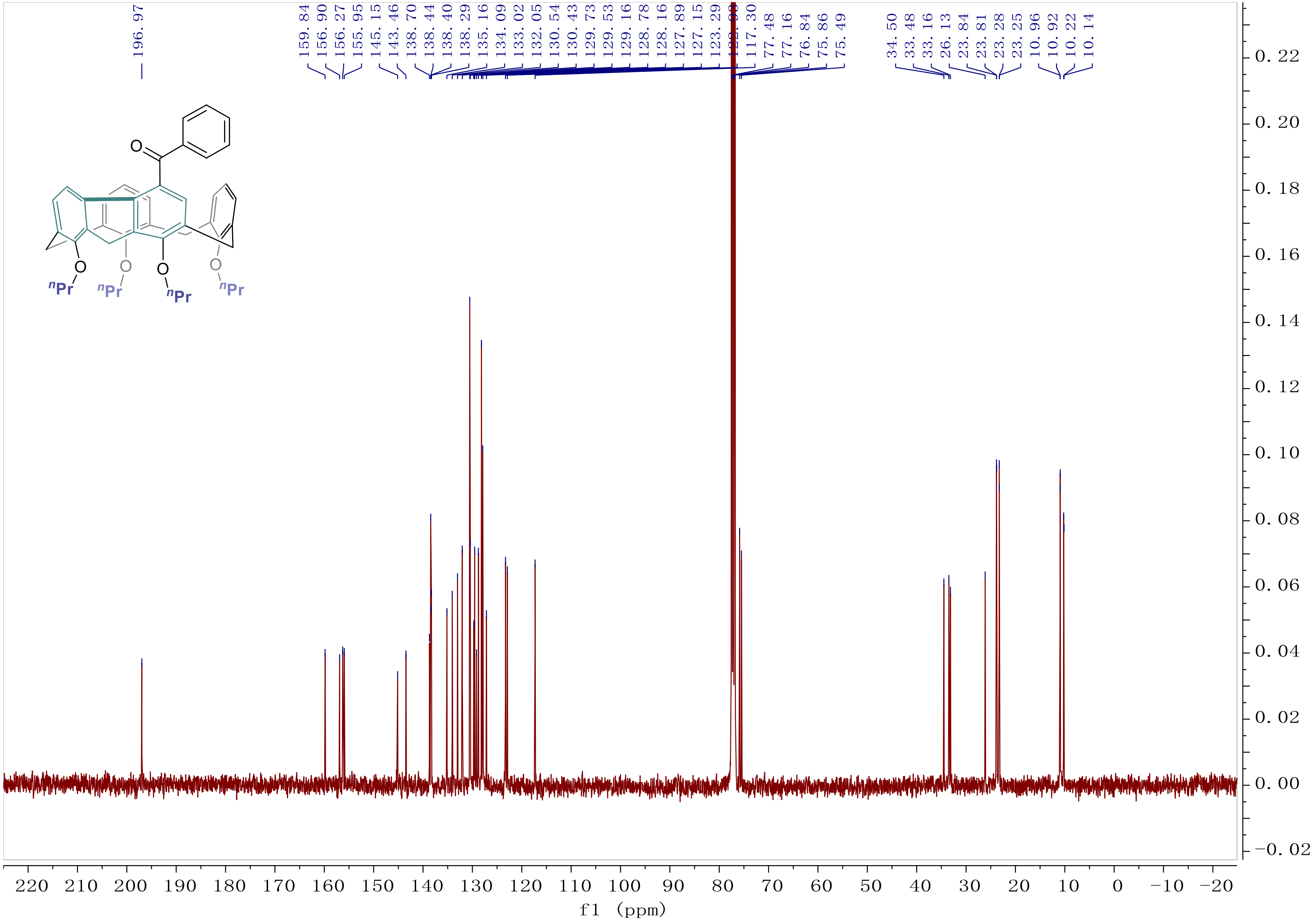

^{1}H NMR (400 MHz, 298 K) spectrum of **3c** in $CDCl_3$

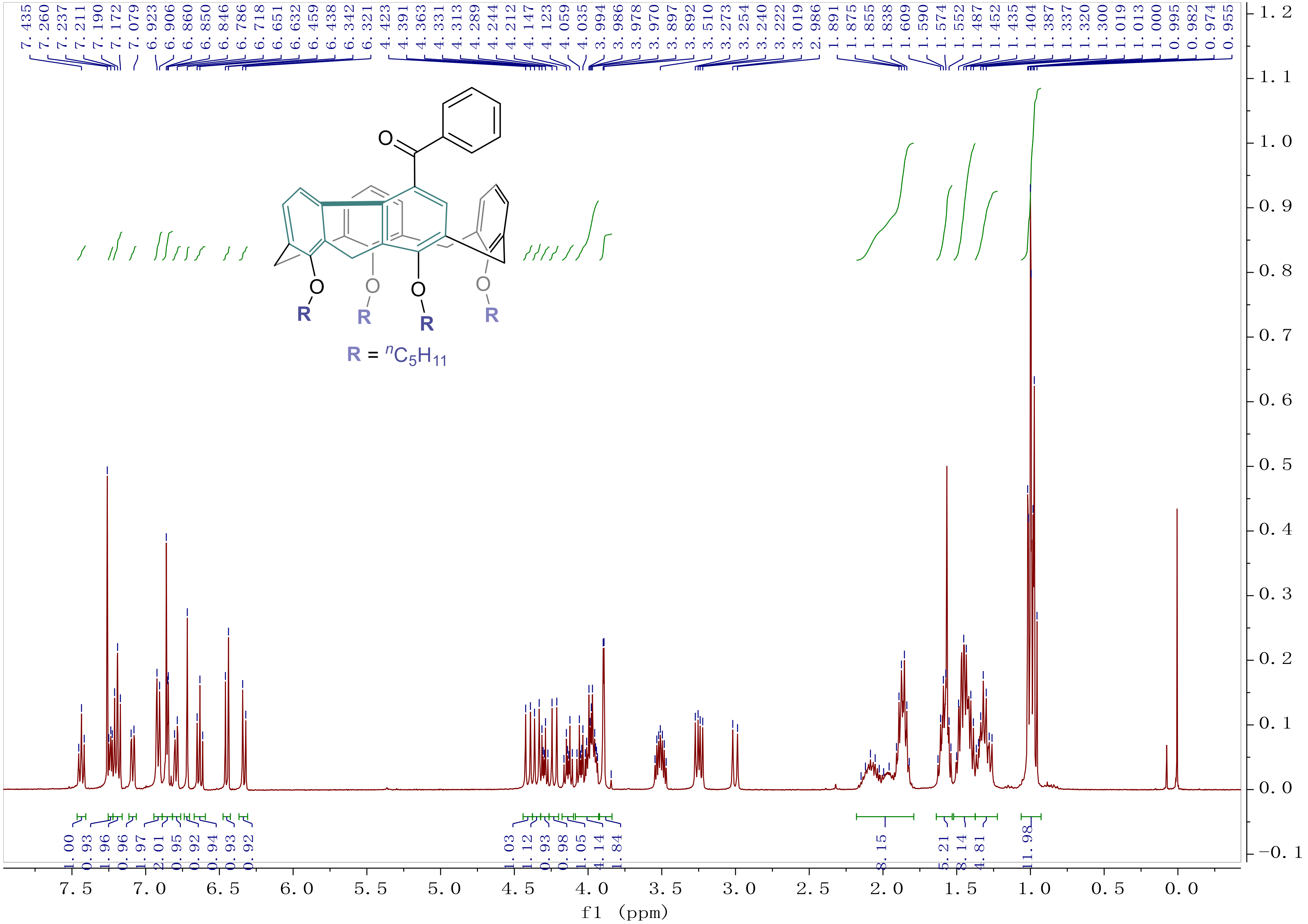

^{13}C NMR (100 MHz, 298 K) spectrum of **3c** in $CDCl_3$

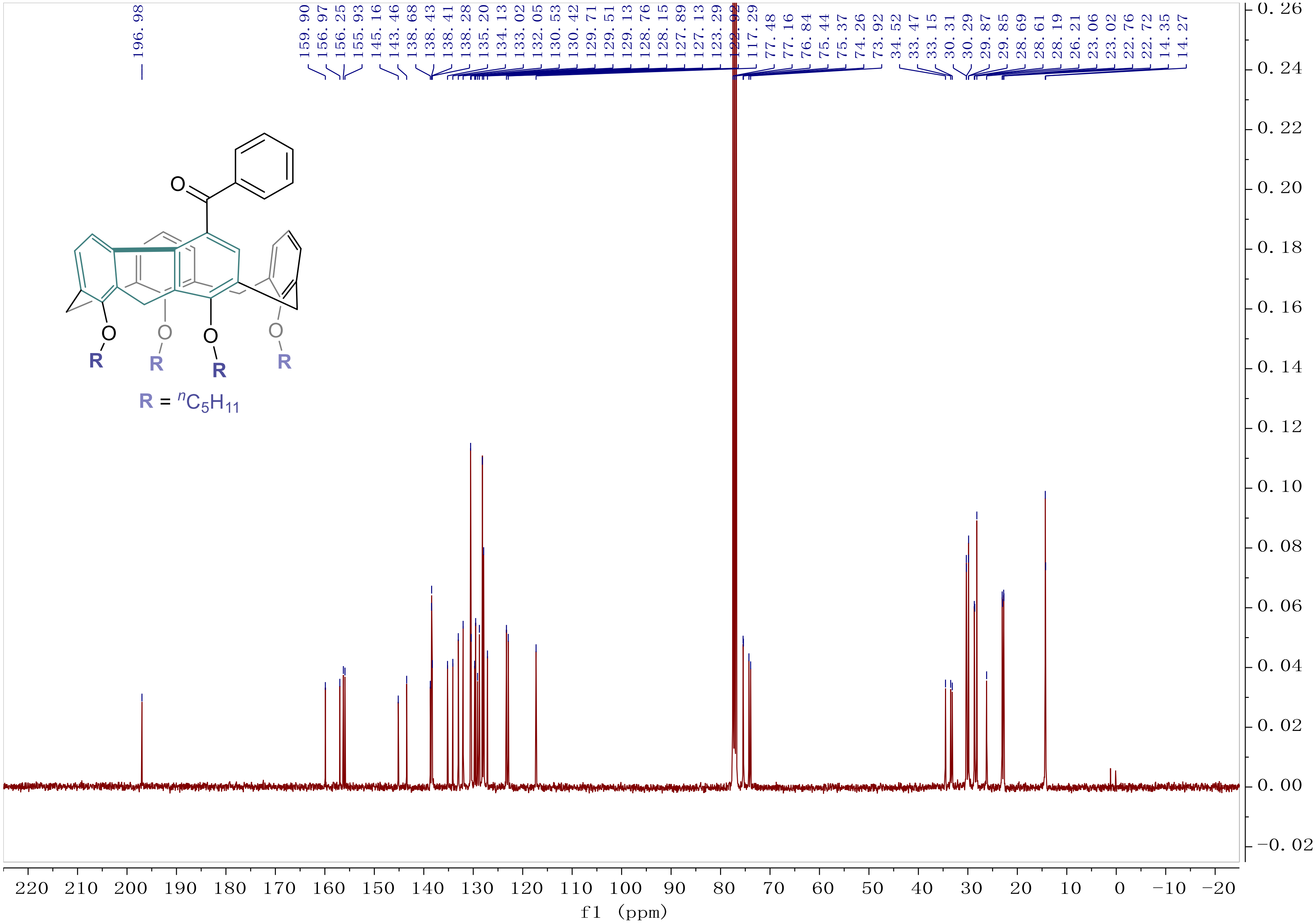

^{1}H NMR (400 MHz, 298 K) spectrum of **3d** in $CDCl_3$

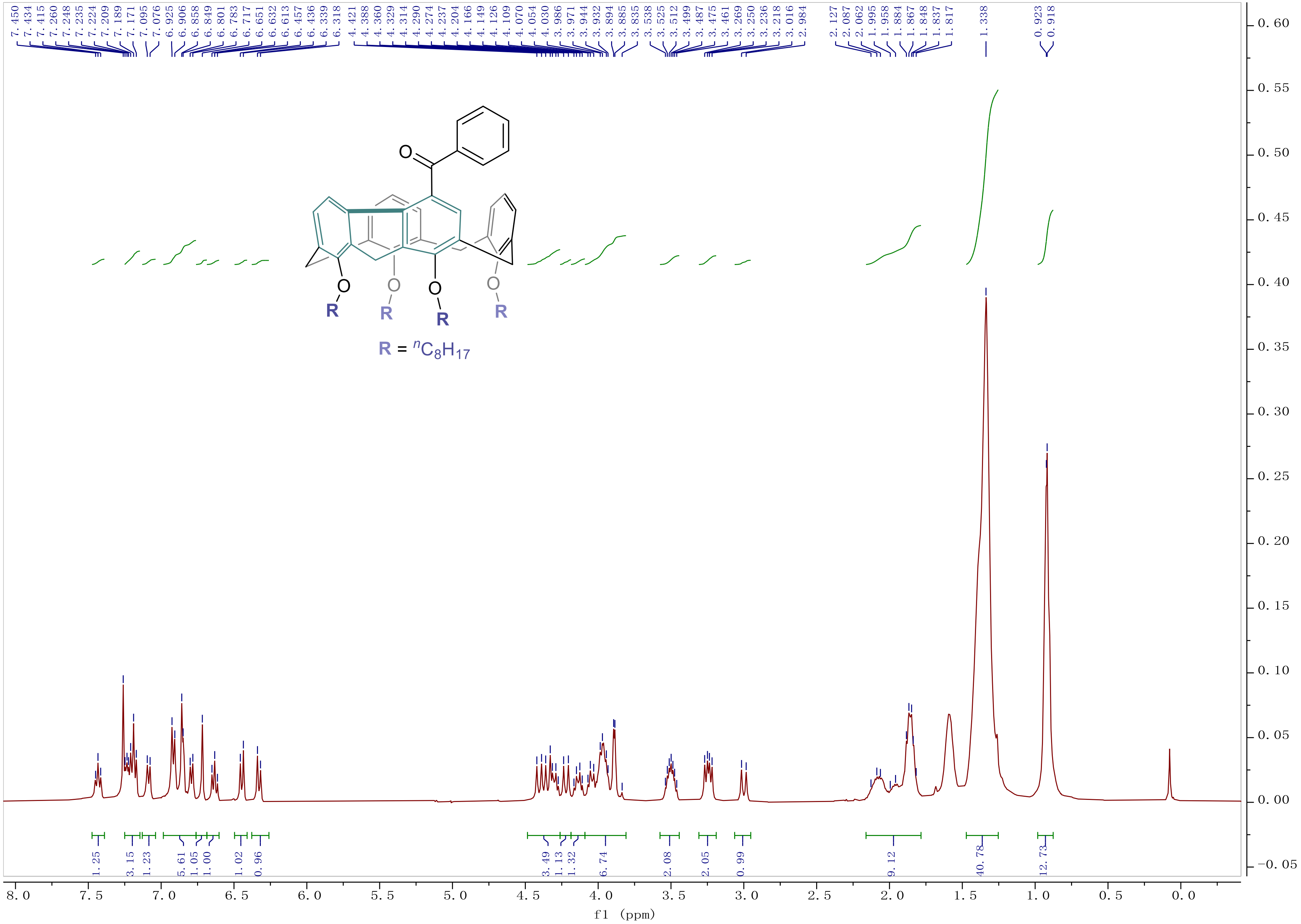

^{13}C NMR (100 MHz, 298 K) spectrum of **3d** in $CDCl_3$

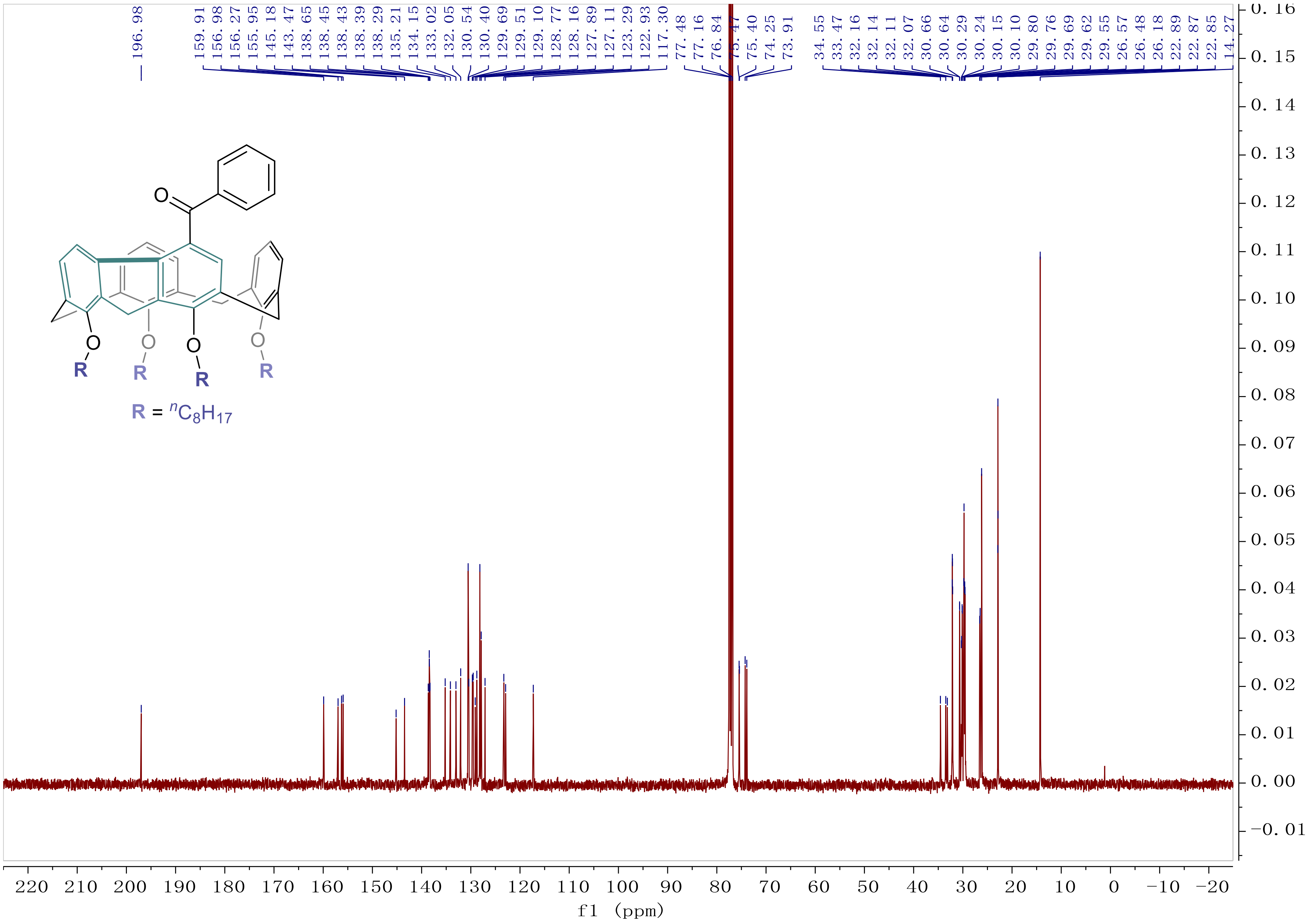

^{1}H NMR (400 MHz, 298 K) spectrum of **3e** in $CDCl_3$

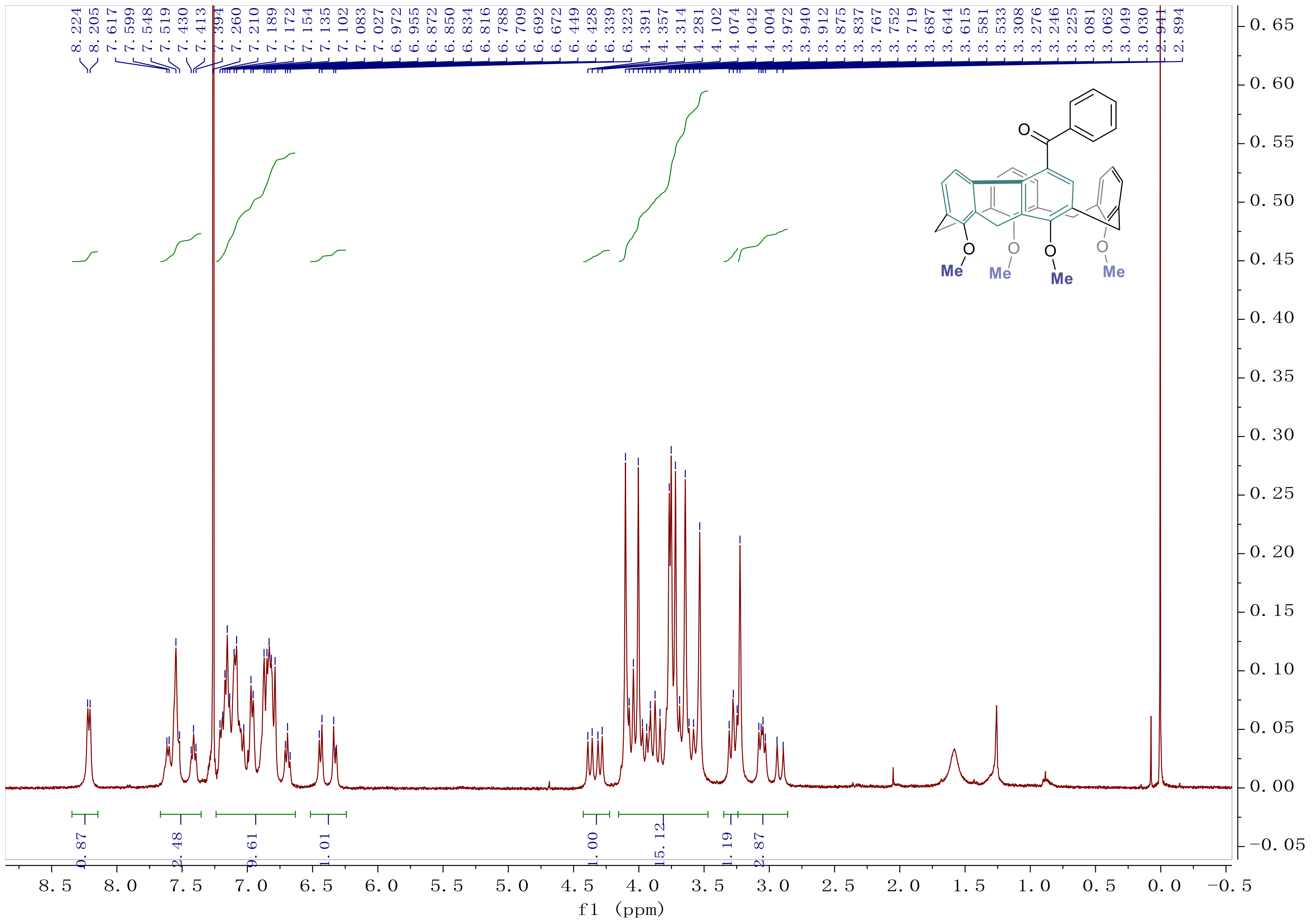

^{13}C NMR (100 MHz, 298 K) spectrum of **3e** in $CDCl_3$

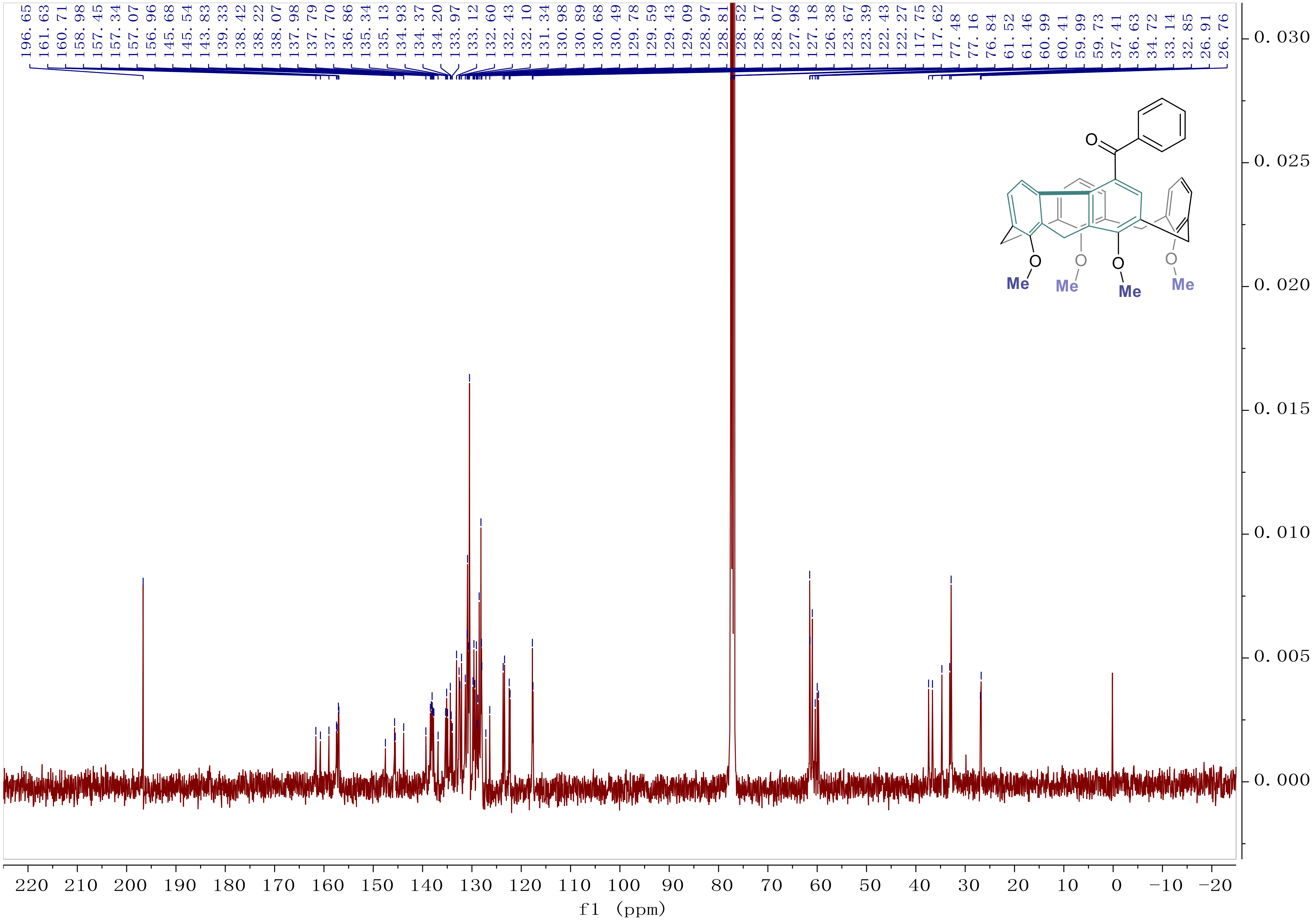

^{1}H NMR (400 MHz, 298 K) spectrum of **3f** in $CDCl_3$

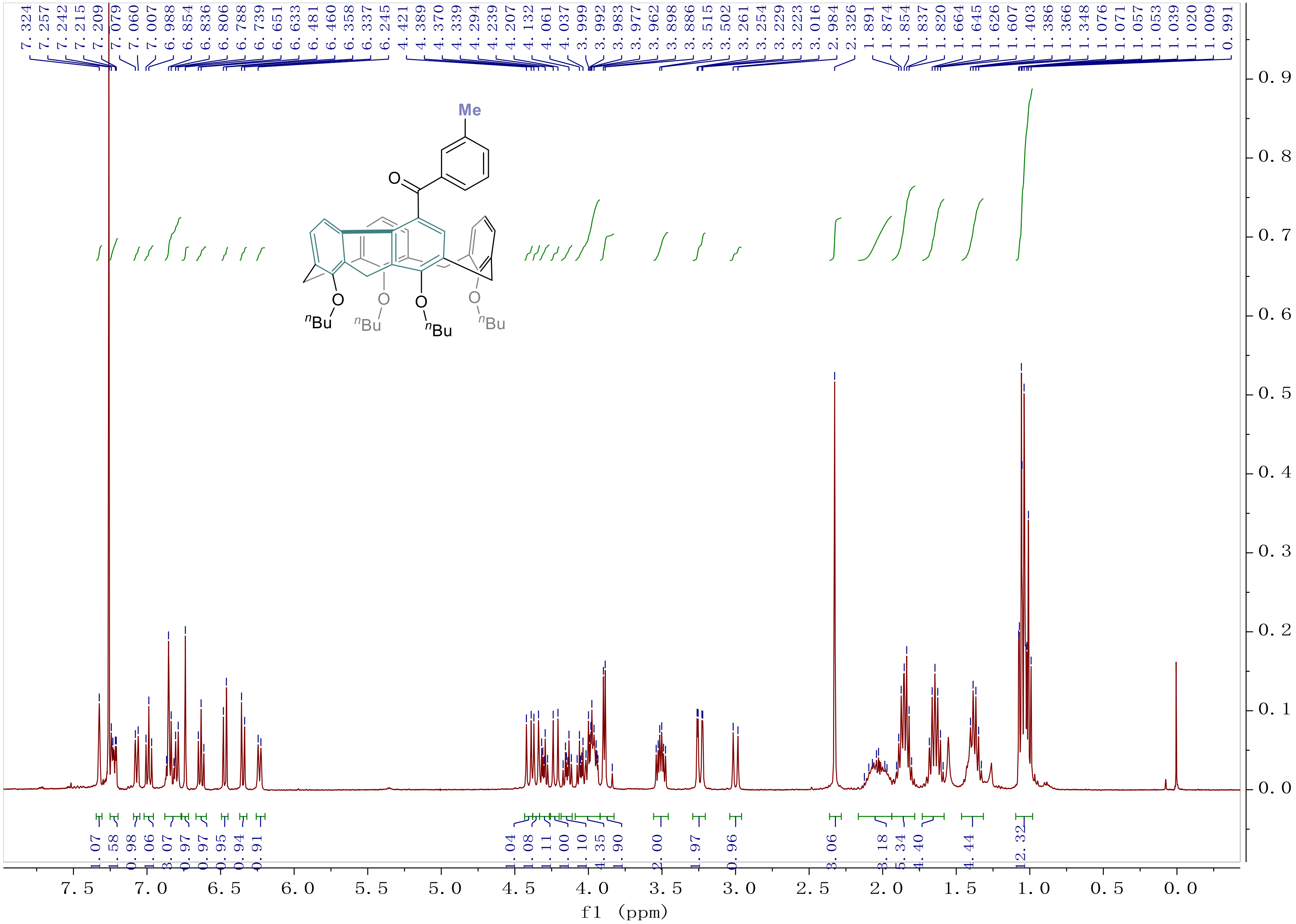

^{13}C NMR (100 MHz, 298 K) spectrum of **3f** in $CDCl_3$

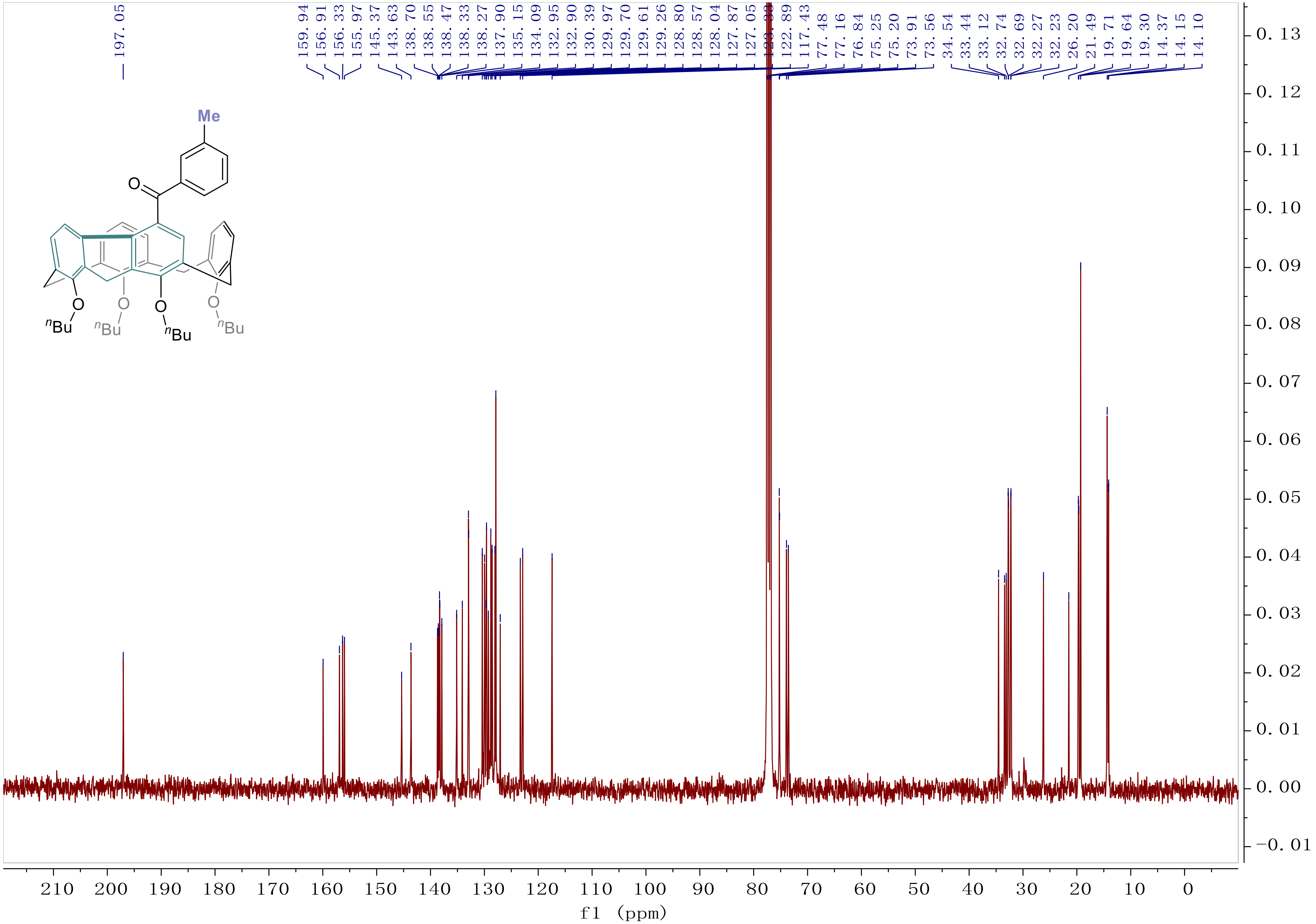

^{1}H NMR (400 MHz, 298 K) spectrum of **3g** in $CDCl_3$

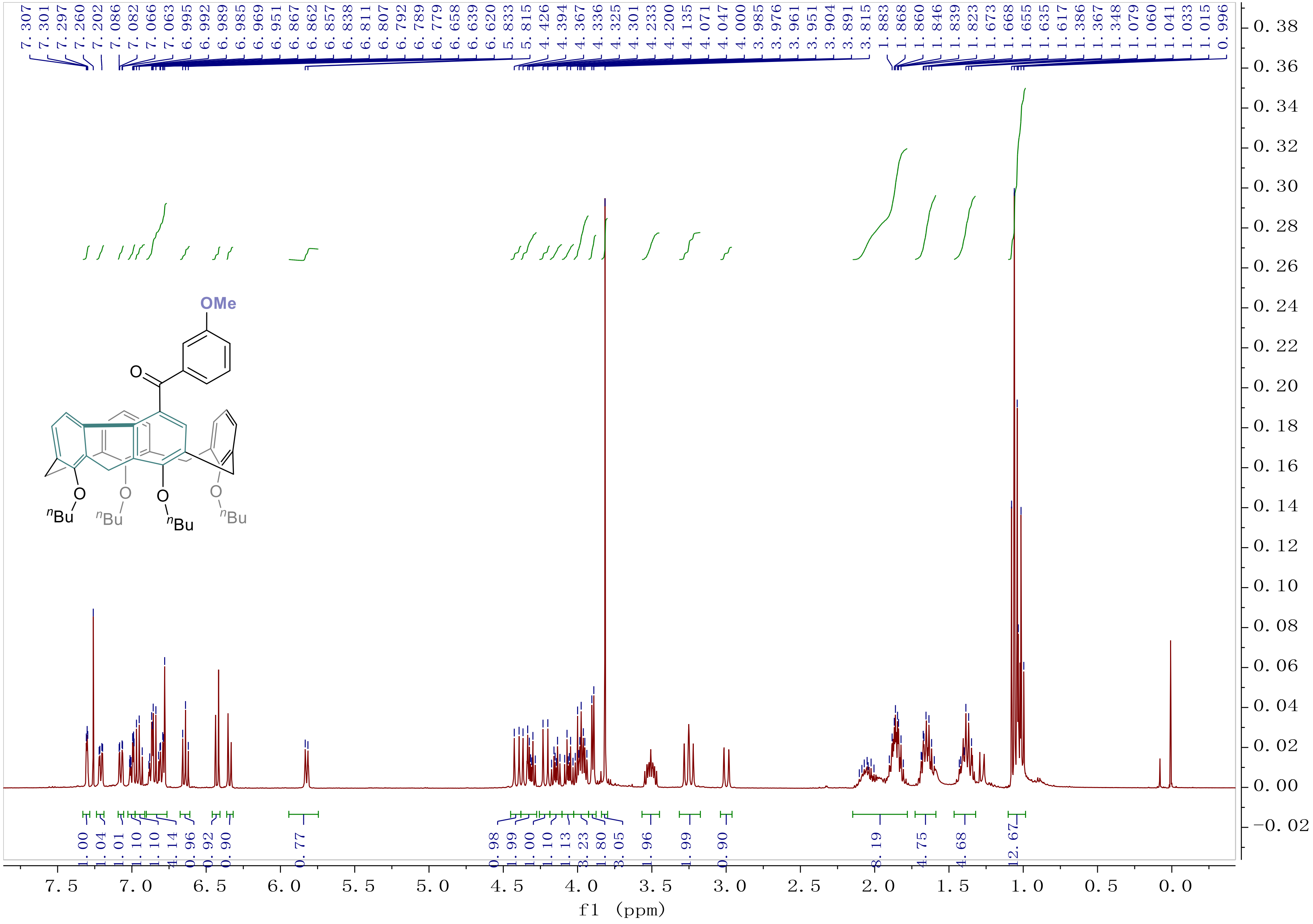

^{13}C NMR (100 MHz, 298 K) spectrum of **3g** in $CDCl_3$

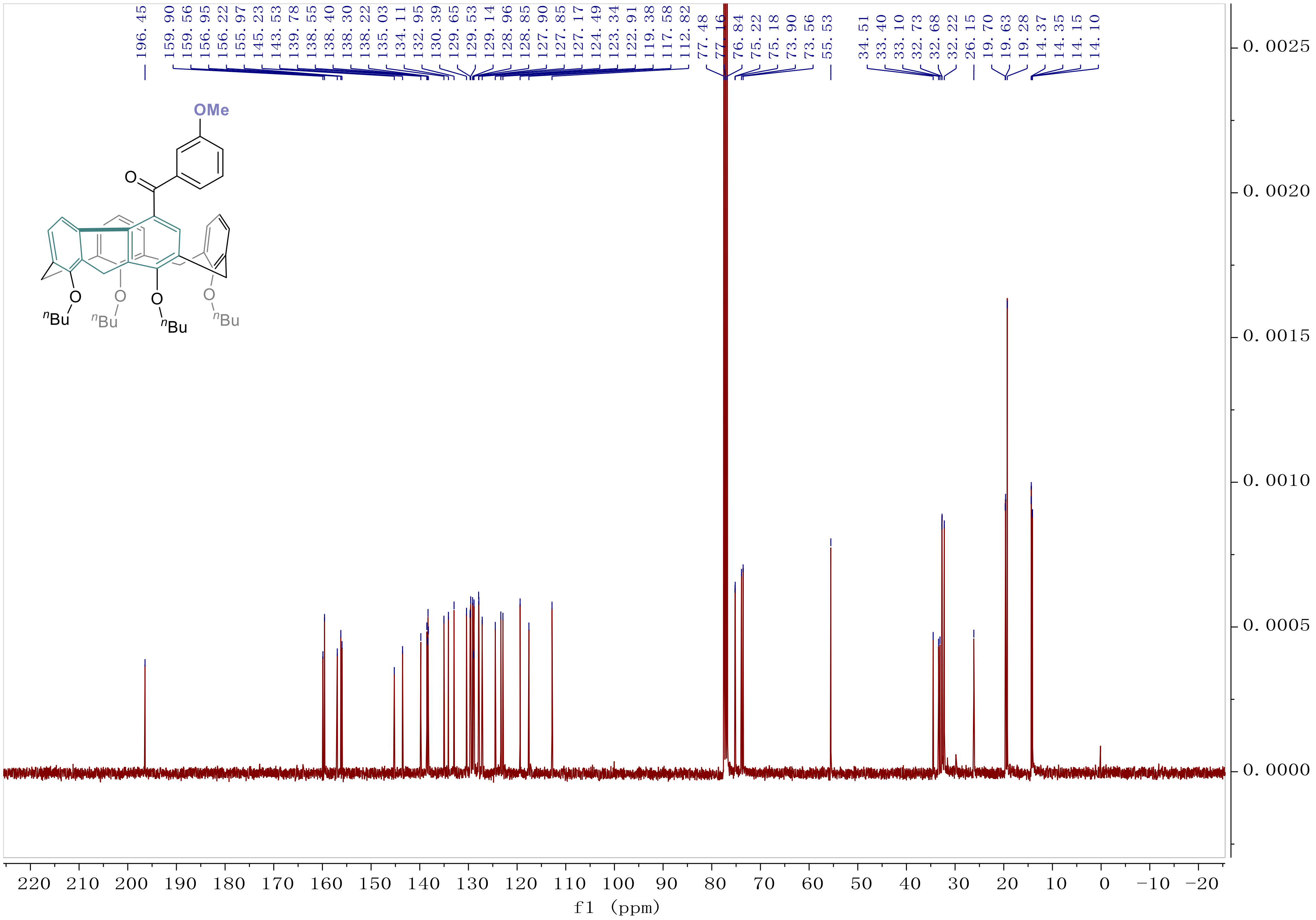

^{1}H NMR (400 MHz, 298 K) spectrum of **3h** in $CDCl_3$

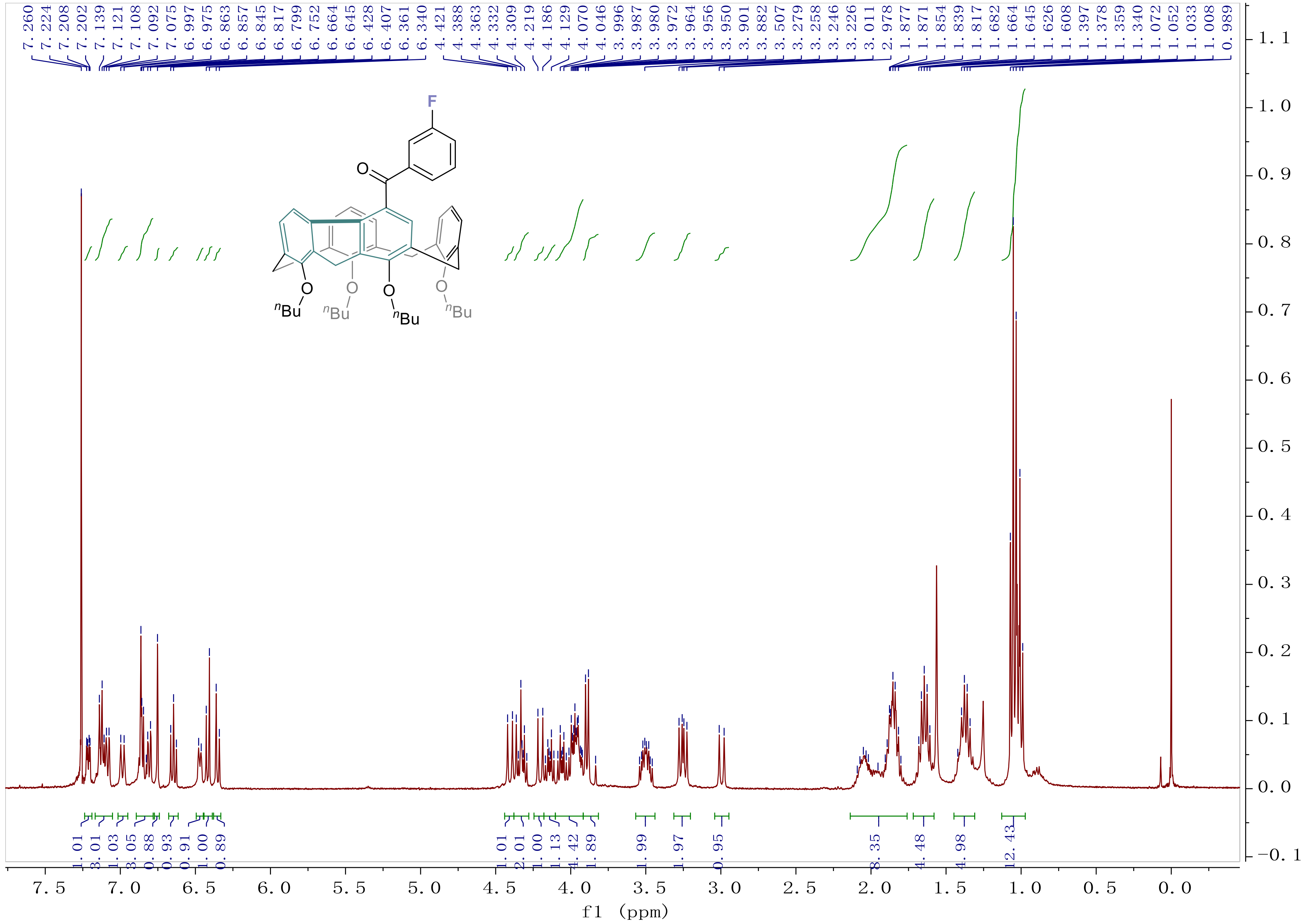

^{13}C NMR (100 MHz, 298 K) spectrum of **3h** in CDCl

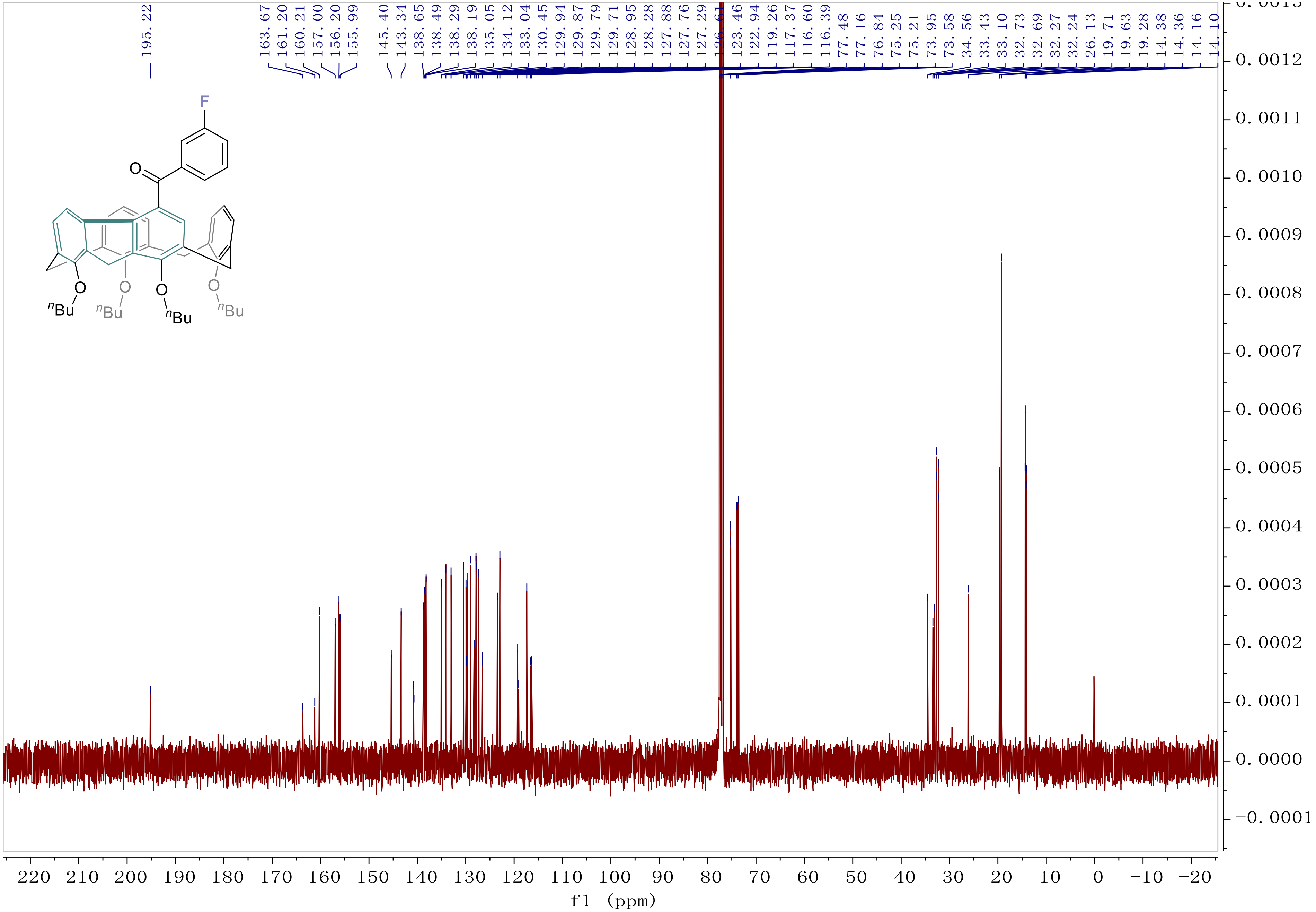

^{1}H NMR (400 MHz, 298 K) spectrum of **3i** in $CDCl_3$

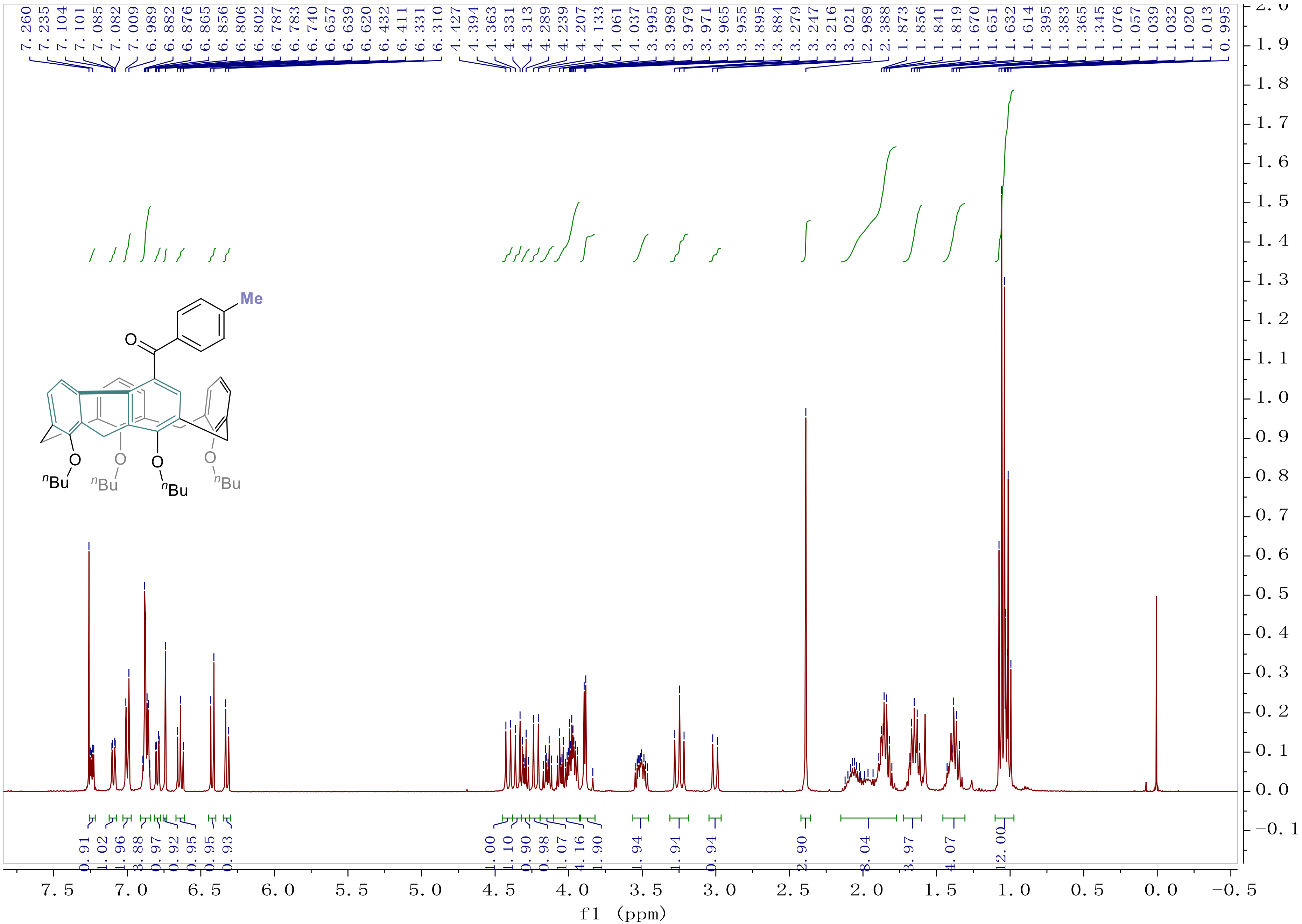

^{13}C NMR (100 MHz, 298 K) spectrum of **3i** in $CDCl_3$

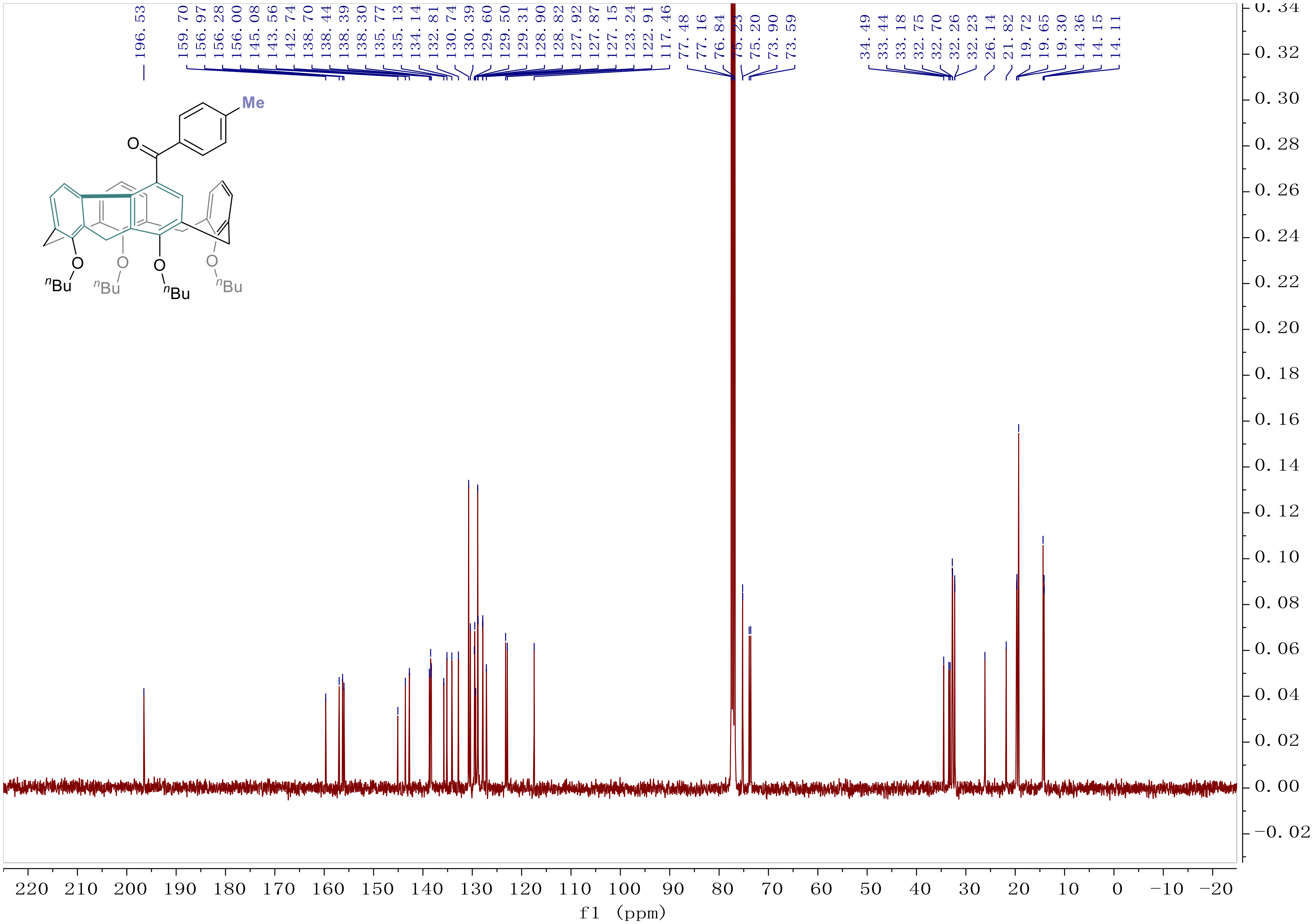

^{1}H NMR (400 MHz, 298 K) spectrum of **3j** in $CDCl_3$

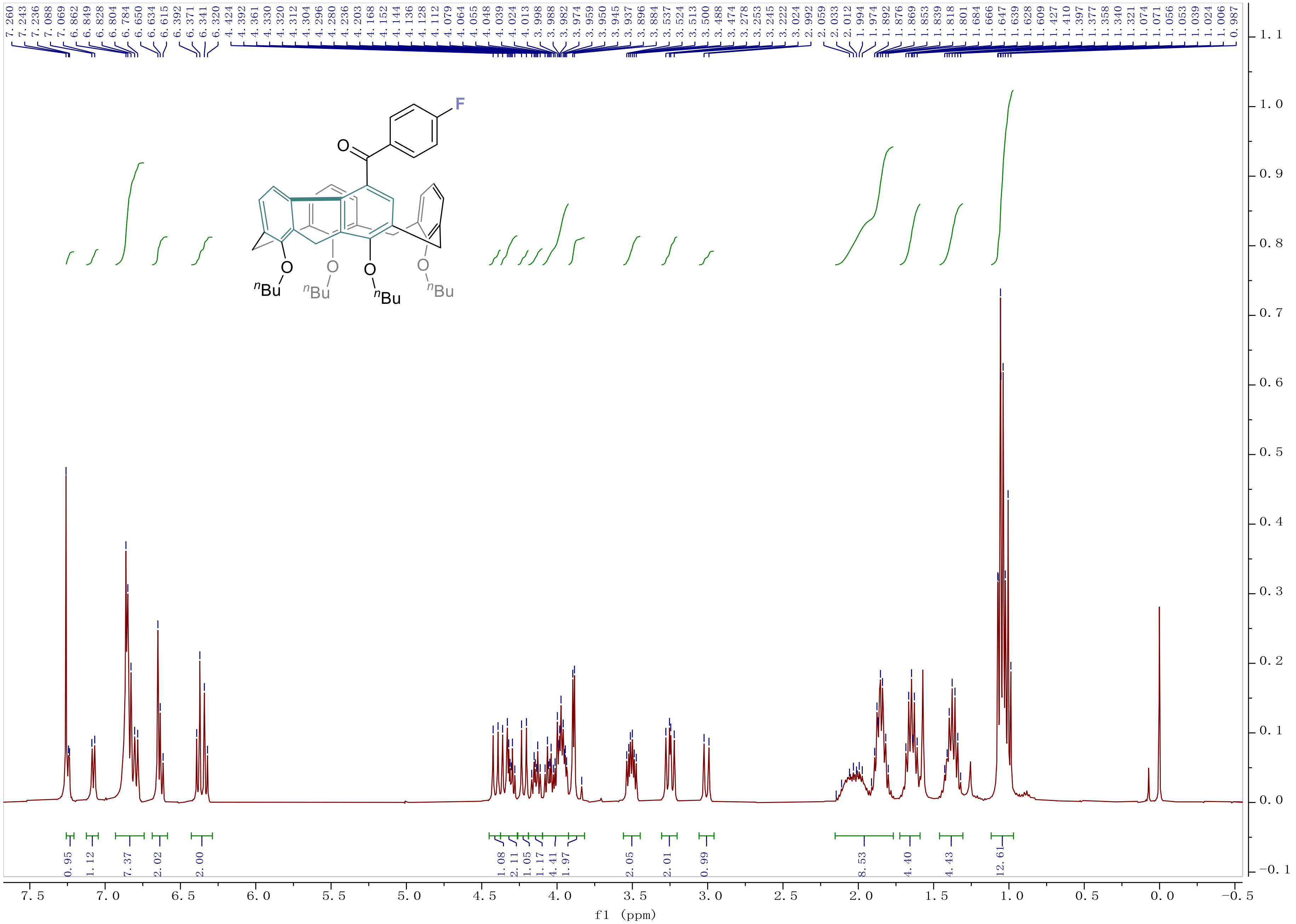

^{13}C NMR (100 MHz, 298 K) spectrum of **3j** in $CDCl_3$

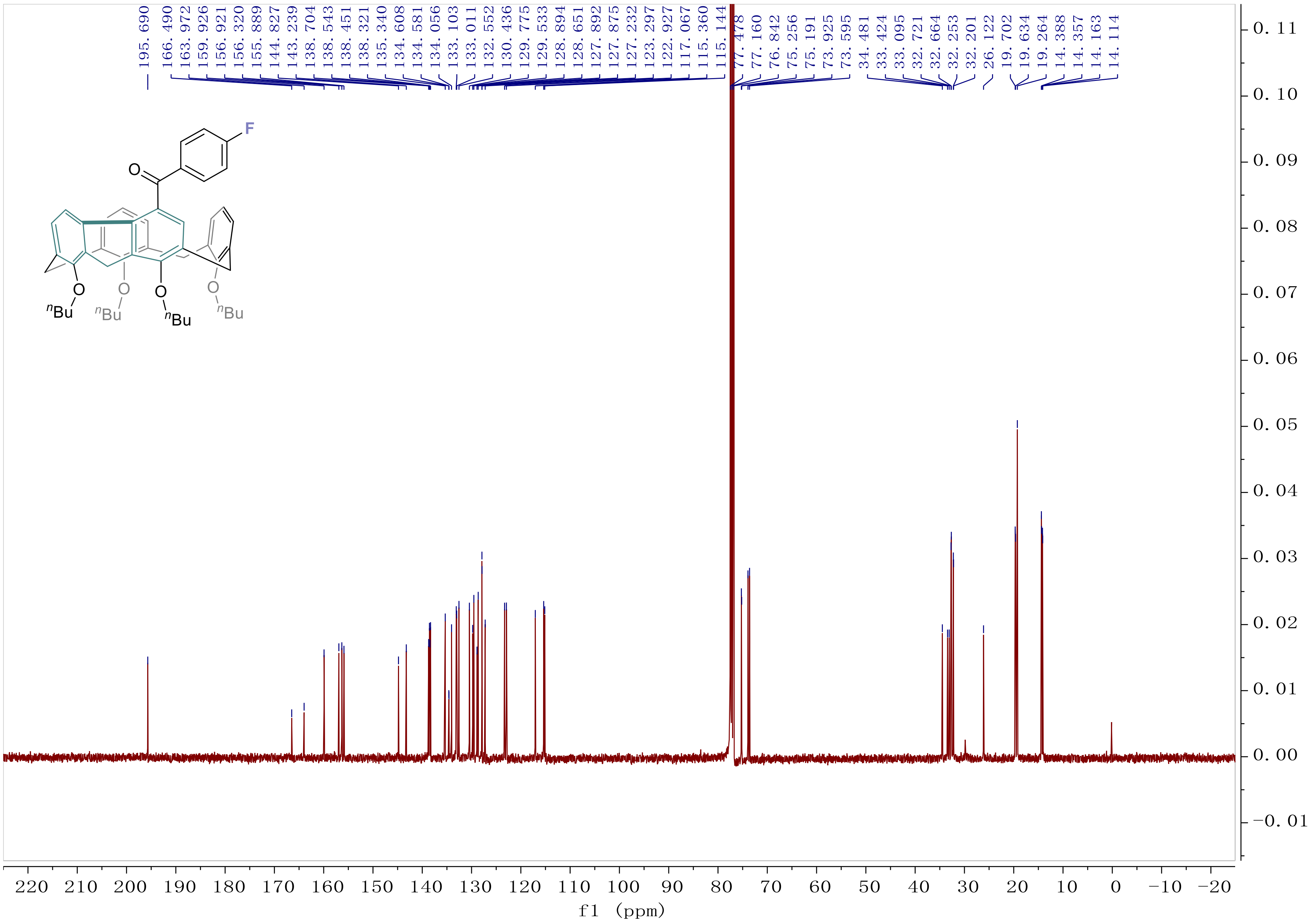

^{1}H NMR (400 MHz, 298 K) spectrum of **3k** in $CDCl_3$

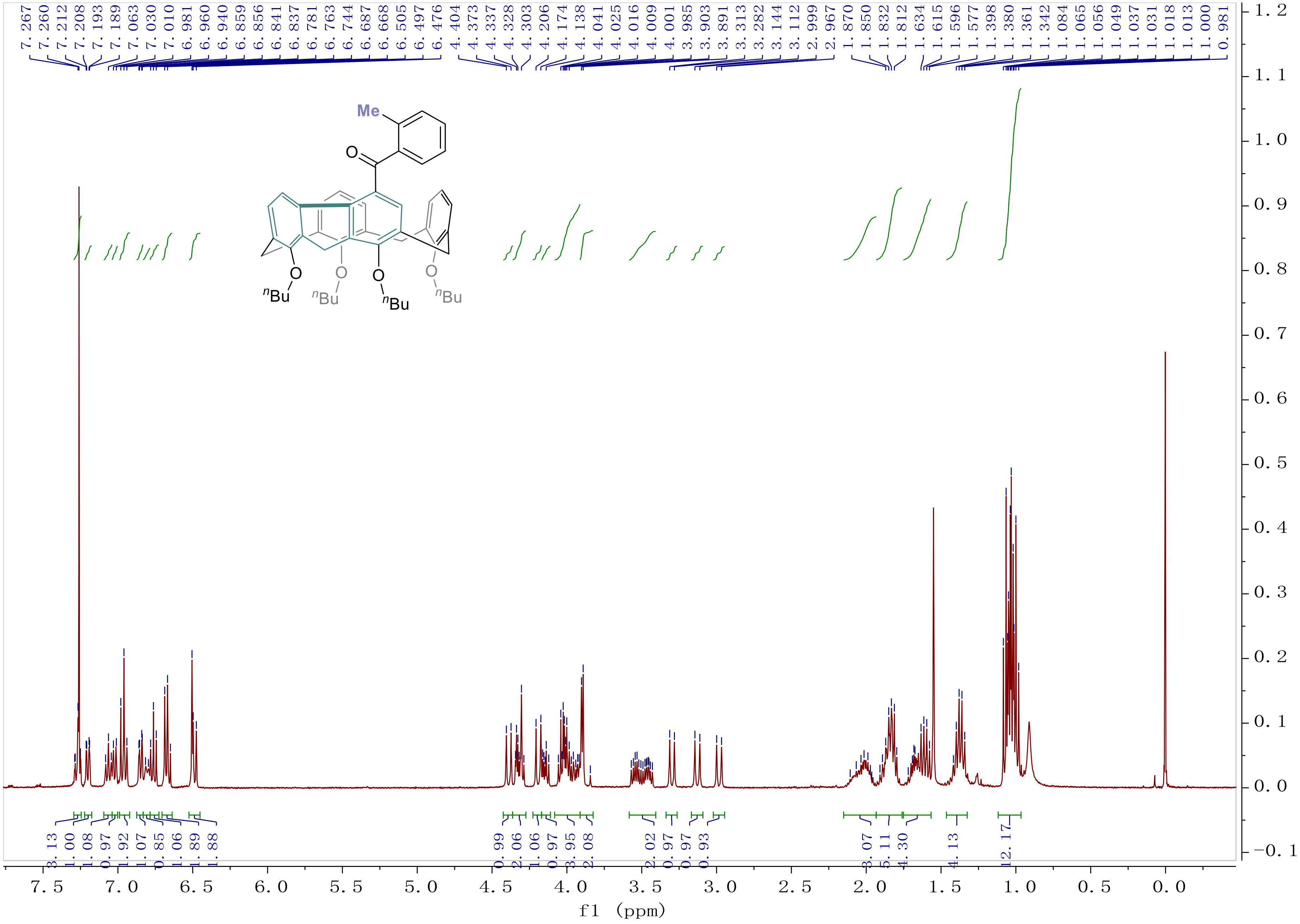

^{13}C NMR (100 MHz, 298 K) spectrum of **3k** in $CDCl_3$

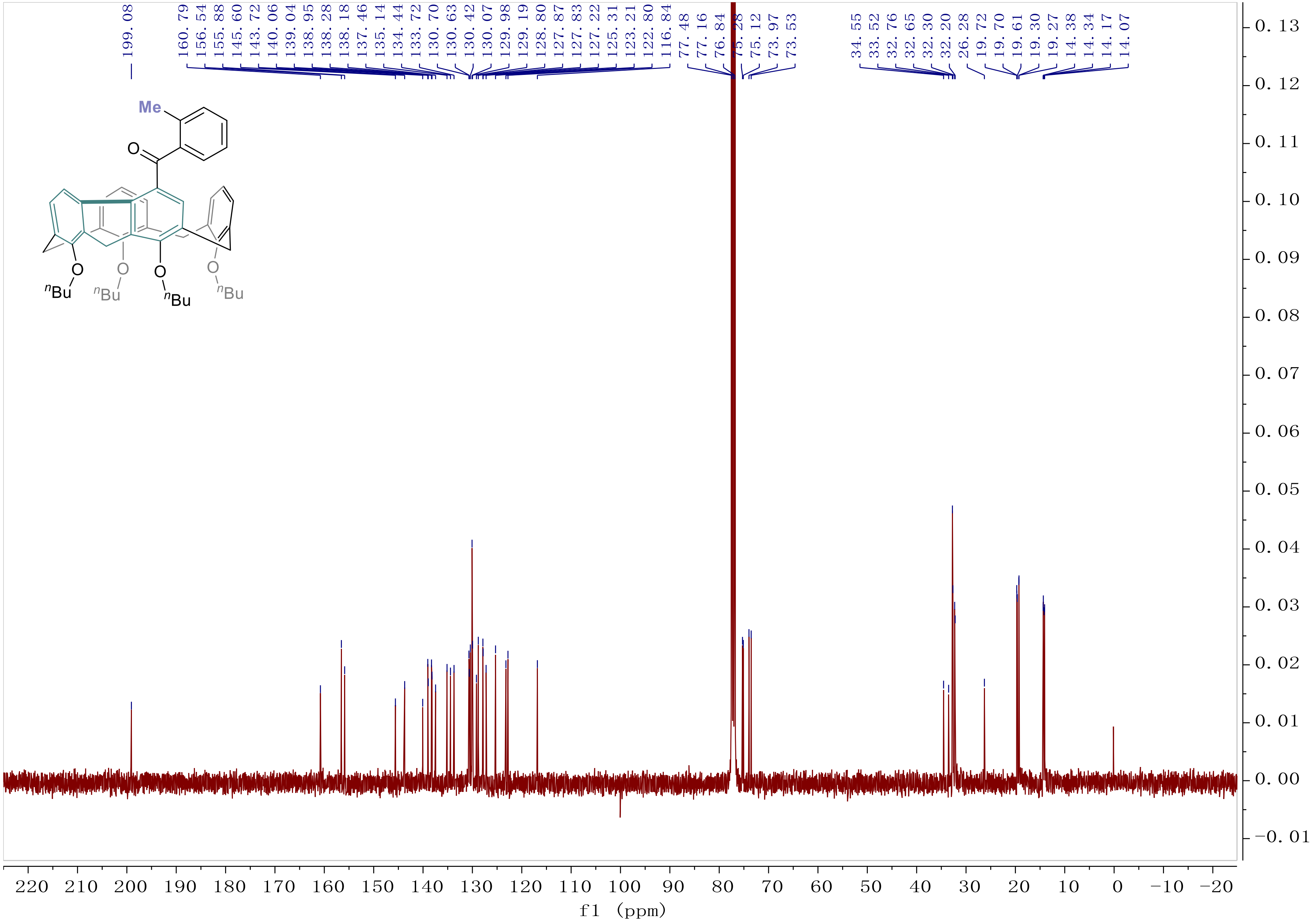

^{1}H NMR (400 MHz, 298 K) spectrum of **5** in $CDCl_3$

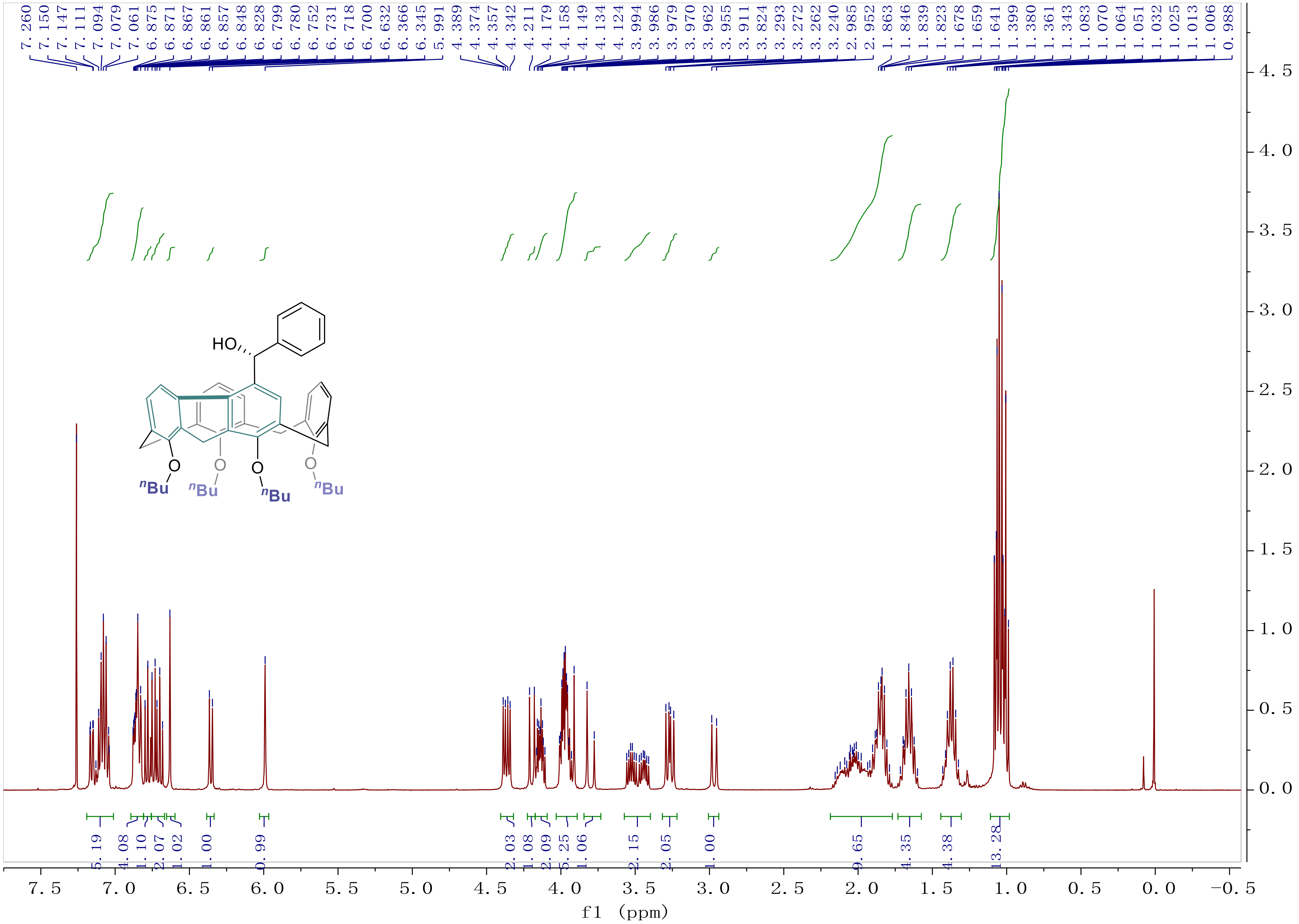

^{13}C NMR (100 MHz, 298 K) spectrum of **5** in $CDCl_3$

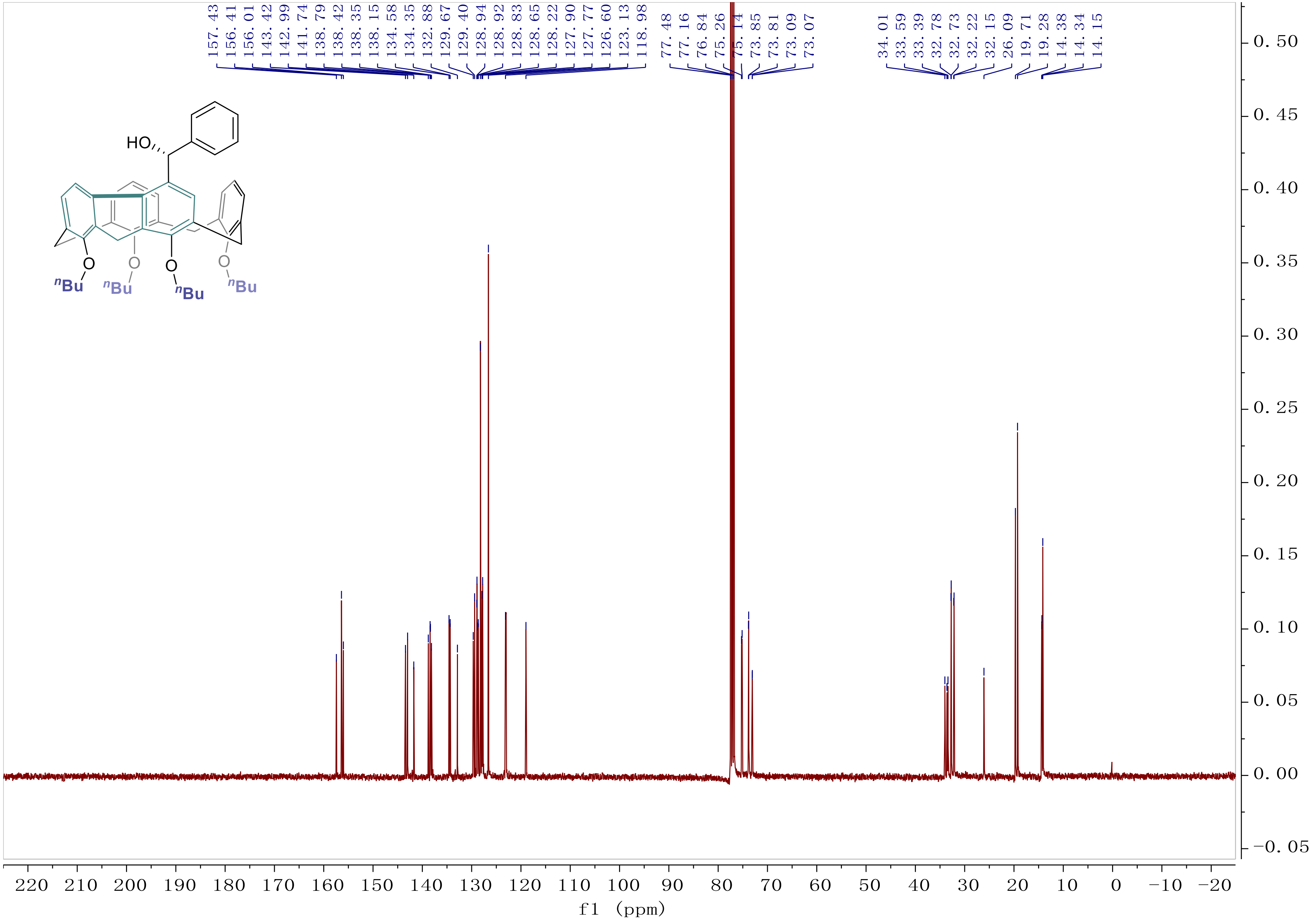

^{1}H NMR (400 MHz, 298 K) spectrum of **6** in $CDCl_3$

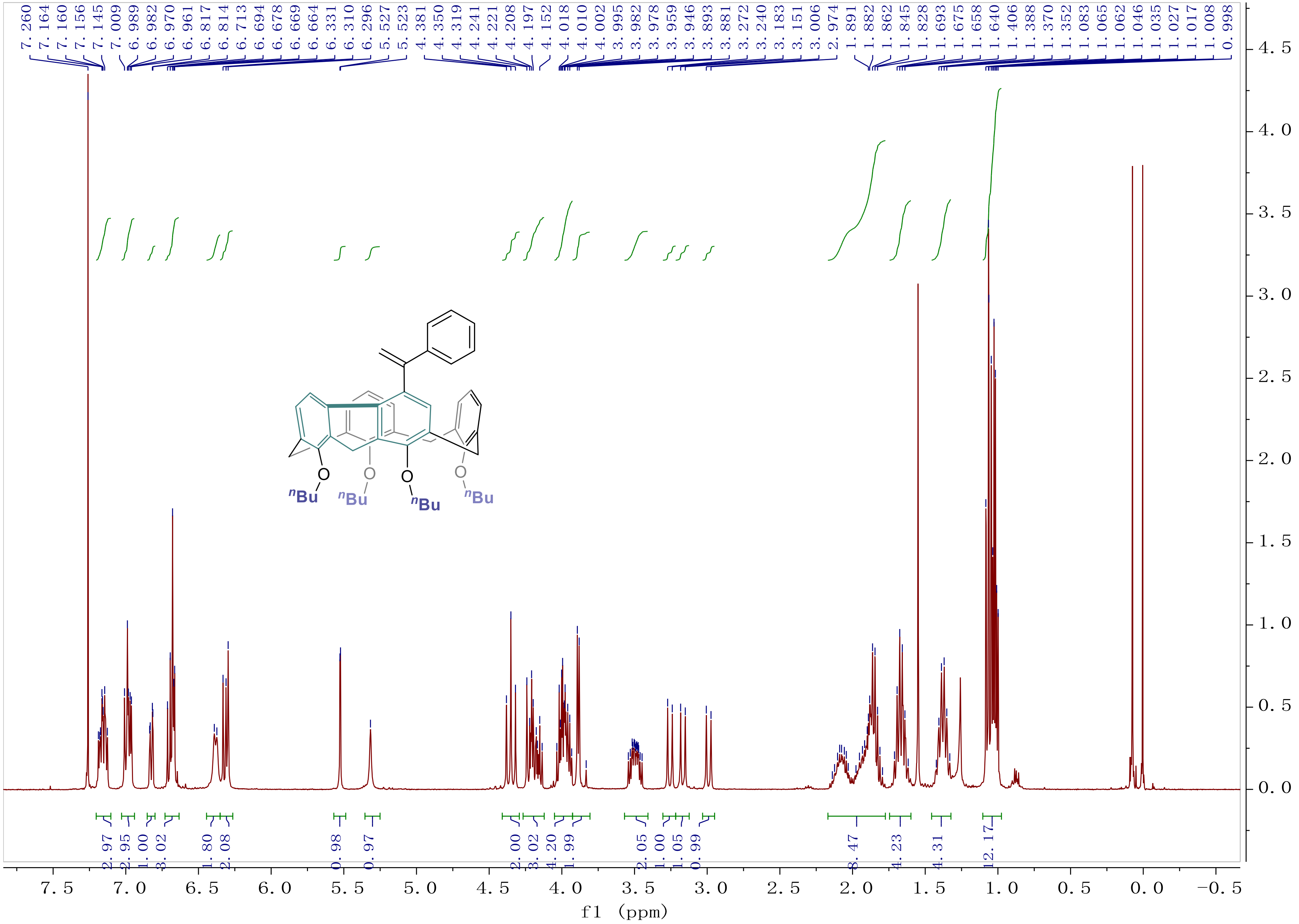

^{13}C NMR (100 MHz, 298 K) spectrum of **6** in $CDCl_3$

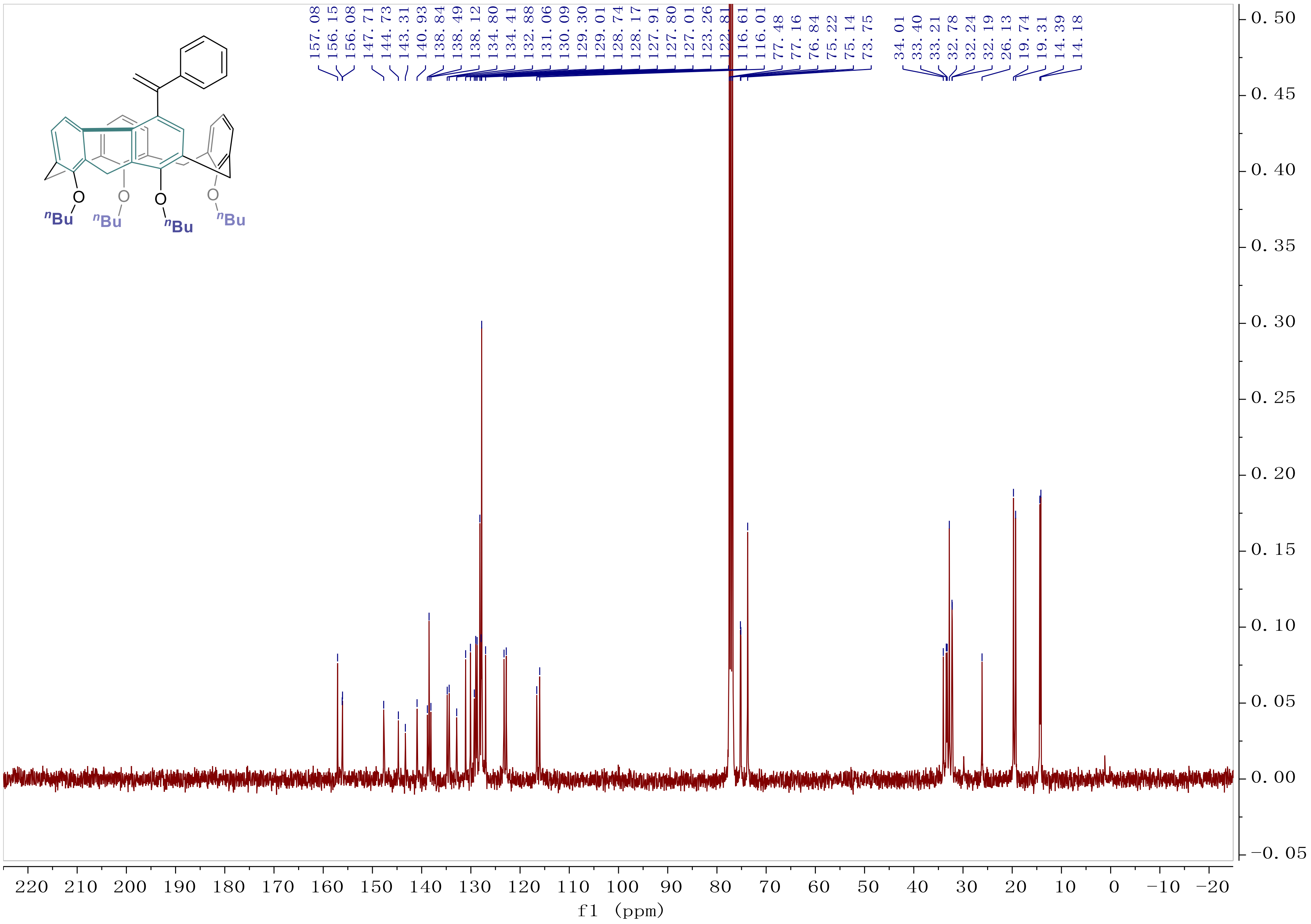

^1H NMR (400 MHz, 298 K) spectrum of **7** in $CDCl_3$

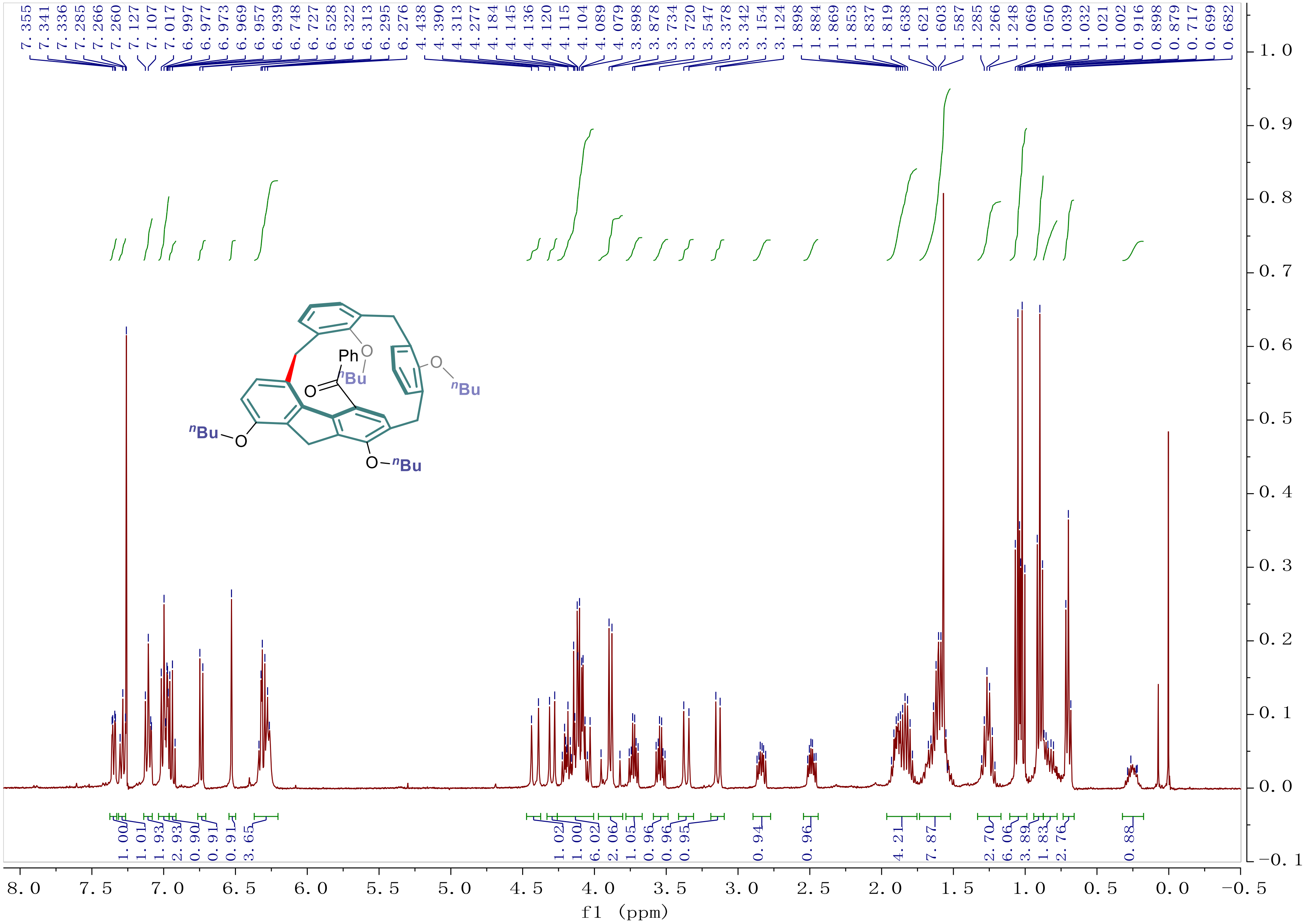

^{13}C NMR (100 MHz, 298 K) spectrum of **7** in $CDCl_3$

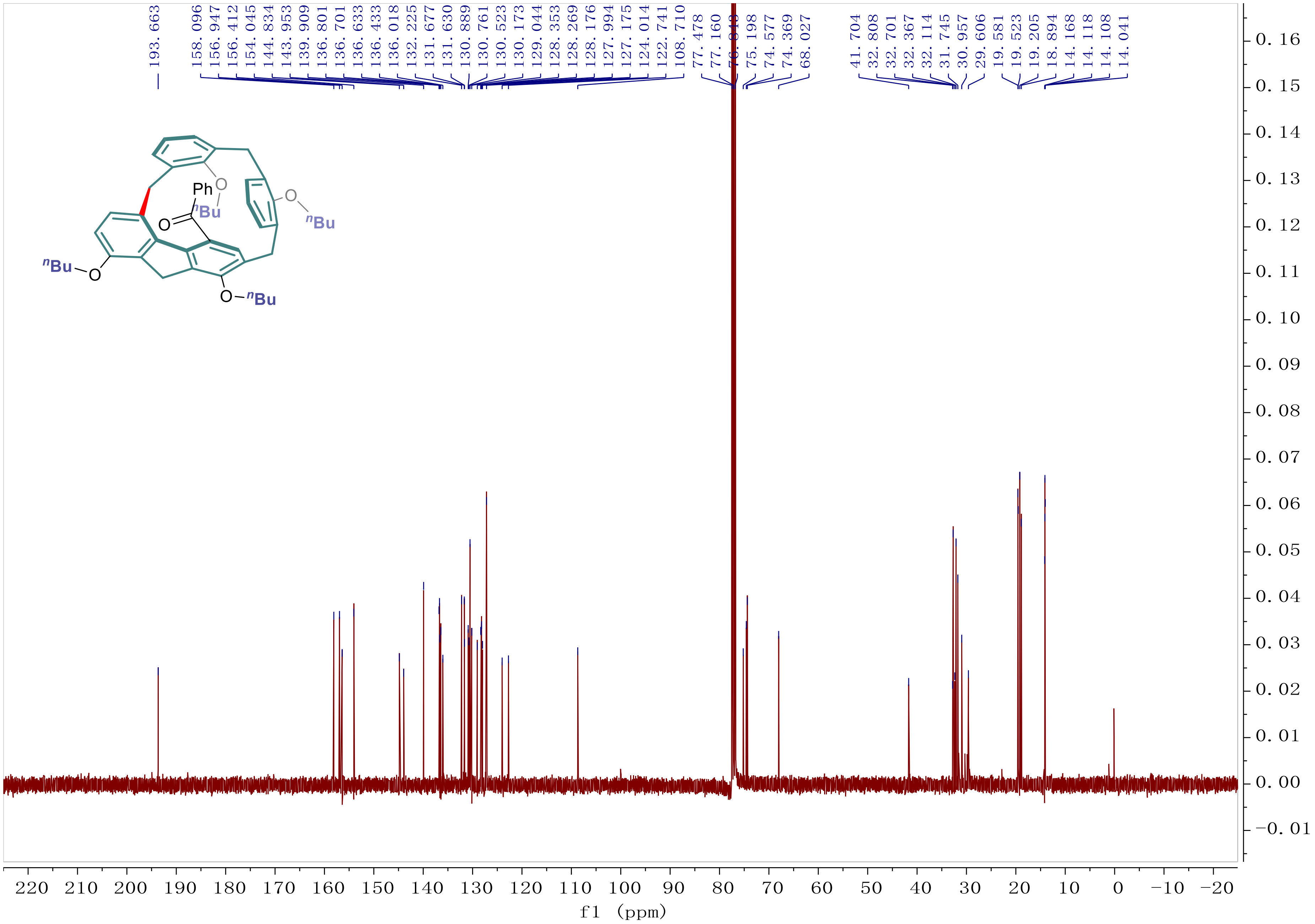

Supplement: SC-014-D2SC06234H-s001 [file SC-014-D2SC06234H-s001.pdf]
